# Can commonly prescribed drugs be repurposed for the prevention or treatment of Alzheimer's and other neurodegenerative diseases? Protocol for an observational cohort study in the UK Clinical Practice Research Datalink

**DOI:** 10.1136/bmjopen-2016-012044

**Published:** 2016-12-12

**Authors:** Venexia M Walker, Neil M Davies, Tim Jones, Patrick G Kehoe, Richard M Martin

**Affiliations:** 1 School of Social and Community Medicine, University of Bristol, Bristol, UK; 2 MRC University of Bristol Integrative Epidemiology Unit, Bristol, UK; 3 NIHR CLAHRC West, Bristol, UK; 4 Dementia Research Group, School of Clinical Sciences, University of Bristol, Bristol, UK

**Keywords:** EPIDEMIOLOGY, THERAPEUTICS

## Abstract

**Introduction:**

Current treatments for Alzheimer's and other neurodegenerative diseases have only limited effectiveness meaning that there is an urgent need for new medications that could influence disease incidence and progression. We will investigate the potential of a selection of commonly prescribed drugs, as a more efficient and cost-effective method of identifying new drugs for the prevention or treatment of Alzheimer's disease, non-Alzheimer's disease dementias, Parkinson's disease and amyotrophic lateral sclerosis. Our research will focus on drugs used for the treatment of hypertension, hypercholesterolaemia and type 2 diabetes, all of which have previously been identified as potentially cerebroprotective and have variable levels of preclinical evidence that suggest they may have beneficial effects for various aspects of dementia pathology.

**Methods and analysis:**

We will conduct a hypothesis testing observational cohort study using data from the Clinical Practice Research Datalink (CPRD). Our analysis will consider four statistical methods, which have different approaches for modelling confounding. These are multivariable adjusted Cox regression; propensity matched regression; instrumental variable analysis and marginal structural models. We will also use an intention-to-treat analysis, whereby we will define all exposures based on the first prescription observed in the database so that the target parameter is comparable to that estimated by a randomised controlled trial.

**Ethics and dissemination:**

This protocol has been approved by the CPRD's Independent Scientific Advisory Committee (ISAC). We will publish the results of the study as open-access peer-reviewed publications and disseminate findings through national and international conferences as are appropriate.

**Strengths and limitations of this study**

- This study will involve a large sample of data and has considerable power to detect even relatively small effects, even under highly conservative Bonferroni corrections. For example, the sample to assess the progression of dementia contains 105 471 patients and has a minimum detectable HR of 0.931.
- We plan to use four different statistical methods in our analysis, which have different approaches for modelling confounding. By doing this, we will be able to assess the merits of each method in the given situation in order to minimise confounding.
- Dementia is a heterogeneous outcome, and electronic codes used to define cases in primary care may not be as accurate as cases in clinical cohorts. We will undertake sensitivity analyses to explore how this may affect our results.

## Introduction

### Rationale

Alzheimer's disease (AD) is a progressive disease affecting brain function and independent living and eventually requires full-time care. There are only a few treatments that temporarily help symptoms such as memory loss; however, these eventually become ineffective as the underlying disease progresses unabated. Part of the difficulty of treating AD is that it involves the activation of many destructive processes in the brain, each of which is likely to need simultaneous treatment if the progression of the disease is to be halted.

For this reason, there is urgent need for new evidence about medications that could influence the incidence and progression of AD. One promising approach is to investigate drug repositioning,^1^ which offers a time-effective and cost-effective alternative to traditional drug development. A recent consensus study of dementia experts identified a short-list of individual and classes of prescribed drugs that may be repurposed as novel treatments for dementia.^2^ The short-list included compounds used to treat hypertension, hypercholesterolaemia and type 2 diabetes, all of which can be classed as having ‘cerebroprotective’ properties and have variable levels of preclinical evidence that suggest they may have beneficial effects for various aspects of dementia pathology. However as yet there is limited pharmacoepidemiological data to support their effects in human populations. Therefore, we plan to investigate whether these existing medications could be repurposed to prevent or treat AD.

Furthermore, the overlap of different forms of neurodegenerative disease would suggest that there may be scope to translate existing or newly identified interventions for testing in neurodegenerative diseases where similarities exist. Thus, we will start by examining the most common form of neurodegenerative disease—AD.^3^ ^4^ We will then investigate whether the drug candidates could also be repurposed to treat or prevent other neurodegenerative diseases—these will include other non-Alzheimer's disease dementias (NADD) (ie, the group for dementias in which AD is not thought to play a part), Parkinson's disease (PD) and amyotrophic lateral sclerosis (ALS). Collectively, these findings will allow the prioritisation of drugs to be tested as repurposed treatments in clinical trials of AD and other neurodegenerative diseases in the future.

### Objectives

To investigate whether commonly prescribed medications, previously identified as potentially cerebroprotective, are associated with the incidence or progression of neurodegenerative disease.

We will focus on the following medications:

- Treatments for hypertension.
- Treatments for hypercholesterolaemia.
- Treatments for type 2 diabetes.

Along with the following neurodegenerative diseases:

- Alzheimer's disease (AD).
- Non-Alzheimer's disease dementias (NADD).
- Parkinson's disease (PD).
- Amyotrophic lateral sclerosis (ALS).

## Methods and analysis

### Study design

We will conduct a hypothesis testing observational cohort study of neurodegenerative disease incidence and progression, using data from the Clinical Practice Research Datalink (CPRD).

### Participants

We will include men and women older than 40 years, with at least 12 consecutive months of records classified as ‘acceptable’ by the CPRD from all ‘up to standard’ practices.^5^ Patients registered at a practice <365 days before their 40th birthday, or those with a first record of one of the index drug classes of interest before their 40th birthday will be excluded. This will ensure high-quality assessment of baseline data and possible confounders.

### Sample selection

We will investigate the incidence and progression of AD, NADD, PD and ALS. Our investigations into the incidence will focus on treatments which are reported to be cerebroprotective and are currently prescribed for hypertension,^6^ hypercholesterolaemia^7^ ^8^ and type 2 diabetes.^9^ For each treatment, we will conduct two analyses. The first will compare treated to untreated individuals with similar indications—this is cohort A. The second will compare the outcomes of individuals given different subclasses of specific medications—this is cohort B. We will also investigate progression using a third cohort, cohort C. Progression will be defined as the postdiagnosis survival of a patient. For each neurodegenerative disease group of interest, we will determine the effects on progression of the three possible new treatments. In cohort C, exposure will be defined in two ways: (1) using patients being prescribed these drugs at the time of diagnosis of their neurodegenerative disease; and (2) using those issued new prescriptions postdiagnosis. The structure of these three cohorts is summarised in table 1 and further explained in the text that follows. All of the cohorts rely on defined diagnoses and exposures, which are recorded in the CPRD through the use of Read and product codes, respectively. Read codes uniquely identify clinical terms recorded by the general practitioner (GP) during a consultation, while product codes uniquely identify prescriptions issued. Both types of code are recorded with a date, and this will be used to determine the dates of diagnosis and exposure required to define the cohorts.

**Table 1:** Comparison of the three cohort types

	Cohort A	Cohort B	Cohort C
Purpose	To investigate incidence by comparing treated and untreated individuals.	To investigate incidence by comparing the different drug subclasses of each treatment.	To investigate progression by comparing treated and untreated individuals.
Number of cohorts required	There will be three cohorts of this type, one for each treatment of interest.	There will be three cohorts of this type, one for each treatment of interest.	There will be three cohorts of this type, one for each of dementia (AD or NADD), PD and ALS.
Exposures	Treatments for hypertension, hypercholesterolaemia and type 2 diabetes.	Treatments for hypertension, hypercholesterolaemia and type 2 diabetes.	Treatments for hypertension, hypercholesterolaemia, and type 2 diabetes.
Start of follow-up (index date)	Date at first risk of the condition the treatment is used for or date of first diagnosis of the condition itself if there was no preceding period ‘at risk’.	Date of first prescription of a treatment of interest.	Date of first diagnosis of neurodegenerative disease of interest.
Outcome	Diagnosis of neurodegenerative disease of interest.	Diagnosis of neurodegenerative disease of interest.	Death.
Exclusion criteria	Individuals with <12 consecutive months of records prior to cohort entry.	Individuals prescribed treatment and control medications at the same time or with <12 consecutive months of records prior to cohort entry.	Individuals with <12 consecutive months of records prior to cohort entry.
Statistical analysis	Conventional regression, propensity score regression, instrumental variable analysis and marginal structural models.	Conventional regression, propensity score regression, instrumental variable analysis and marginal structural models.	Conventional regression, propensity score regression and marginal structural models.

AD, Alzheimer's disease; ALS, amyotrophic lateral sclerosis; NADD, Non-Alzheimer's disease dementias; PD, Parkinson's disease.

#### Cohort A

**Figure 1:**
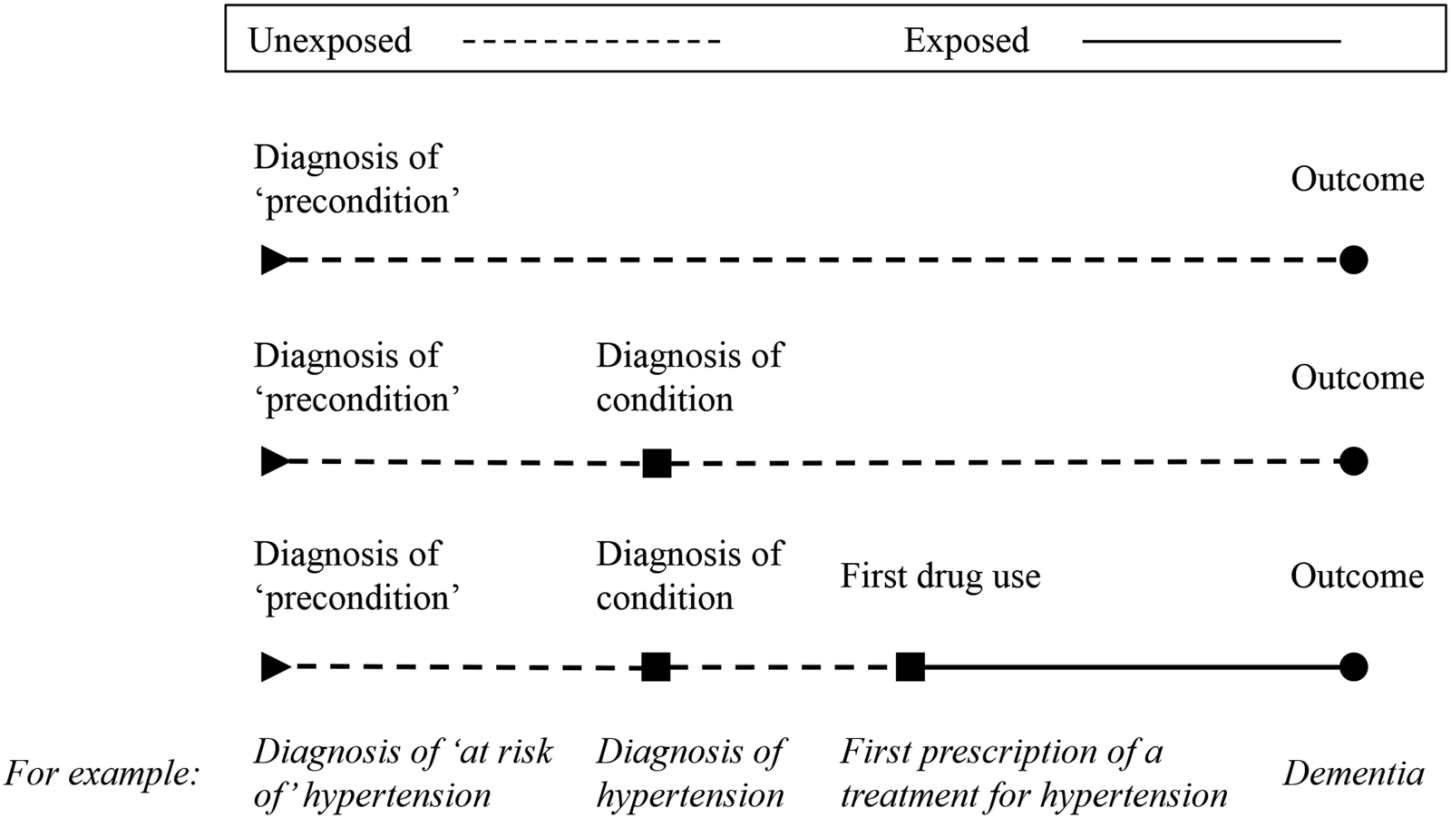
The cohort construction for cohort type A, designed to eliminate immortal time bias. A patient will enter the cohort for a given treatment when they first become ‘at risk’ of the condition the treatment is used for or, in the case of no period ‘at risk’, when they are first diagnosed with the condition (see table 2). For example, when they are diagnosed as at ‘at risk of’ hypertension or, in the case of no period ‘at risk’, when they receive a diagnosis of hypertension. We define cohort entry in this way to avoid excluded immortal time bias that can occur when cohort entry is determined by treatment variation over time.^33^ ^34^ Immortal time is the period during follow-up when the outcome cannot occur. Consider the hypertension example above, suppose we started following the treated patients in our cohort from the date of their first prescription of a treatment for hypertension and the untreated patients from a matched date. This would make it impossible for the treated patients to have an outcome, such as dementia, prior to their first prescription. Consequently, patients in this group would all have a period before treatment, when they could not be diagnosed with dementia, that is, they could not experience the outcome. This period is their immortal time, and it must be correctly attributed to the ‘unexposed’ group so that their outcome is not falsely attributed to their exposure. To do this, patients in the ‘exposed’ and ‘unexposed’ groups must be followed-up and compared from the same start date. In this cohort, we are minimising excluded immortal time by following patients from either a test result or diagnosis where possible. In order to capture all relevant patients, we will allow those receiving treatment without a recorded diagnosis to be included as it is assumed treatment suggests a diagnosis.

For each treatment, we will create a cohort of patients who are diagnosed with the condition the treatment is used for or ‘at risk’ of that condition. This will be determined by the indications and test results described in table 2. We will split each cohort into ‘exposed’ and ‘unexposed’ groups. The ‘exposed’ group will consist of patients who received a treatment of interest within 6 months of initial diagnosis of the condition for which the treatment is being given (the ‘indication’ for treatment). The ‘unexposed’ group will consist of all others in the cohort. Patients will be identified as having received a treatment of interest by product codes (see online supplementary files 1–3). Therefore, in this analysis, we will compare an ‘exposed’ group of individuals who were prescribed the treatment to an ‘unexposed’ group of individuals who received similar test results (ie, were ‘at risk’ of or diagnosed with the indication for treatment), but did not receive the treatment, as illustrated in figure 1. The aim of this approach is to address confounding by indication that could cause bias if the ‘unexposed’ group is drawn from the full population, whom are likely to be healthier. It will also allow us to minimise excluded immortal time bias as patients will be followed from a consistent index date (date first at risk or date of first diagnosis of the condition itself if there was no preceding period at risk).

**Table 2:** The cohorts of type A, one for each treatment of interest

Cohort	Entry criteria
Treatments for hypertension	Patients who are ‘at risk’ of hypertension as indicated by one of the following: Medical code indicating a diagnosis of ‘at risk’ of hypertension. Recorded systolic blood pressure test result between 120 and 139 mm Hg. Recorded diastolic blood pressure test result between 80 and 89 mm Hg. In the case of no period ‘at risk’, patients who have hypertension as indicated by one of the following: Medical code indicating a diagnosis of hypertension. Product code indicating treatment for hypertension. Recorded systolic blood pressure test result of 140 mm Hg or more. Recorded diastolic blood pressure test result of 90 mm Hg or more. See online supplementary file 1 for code lists relating to hypertension.
Treatments for hypercholesterolaemia	Patients who are ‘at risk’ of hypercholesterolaemia as indicated by one of the following: Medical code indicating a diagnosis of ‘at risk’ of hypercholesterolaemia. Recorded test result where total cholesterol level is between 4 and 5 mmol/L. Recorded test result where LDL cholesterol level is between 2 and 3 mmol/L. In the case of no period ‘at risk’, patients who have hypercholesterolaemia as indicated by one of the following: Medical code indicating a diagnosis of hypercholesterolaemia. Product code indicating treatment for hypercholesterolaemia. Recorded test result where total cholesterol level exceeds 5 mmol/L. Recorded test result where LDL cholesterol level exceeds 3 mmol/L. See online supplementary file 2 for code lists relating to hypercholesterolaemia.
Treatments for type 2 diabetes	Patients who are ‘at risk’ of type 2 diabetes as indicated by a medical code. In the case of no period ‘at risk’, patients who have type 2 diabetes as indicated by one of the following: Medical code indicating a diagnosis of type 2 diabetes. Product code indicating treatment for type 2 diabetes. Medical code indicating a diagnosis of unspecified diabetes, first received over the age of 40. Product code indicating treatment with insulin, first received over the age of 40. This assumes that patients treated with insulin or receiving a diagnosis of unspecified diabetes after the age of 40 have type 2 diabetes. Patients with a recorded diagnosis of type 1 diabetes at any age will be excluded. See online supplementary file 3 for code lists relating to type 2 diabetes.

LDL, low density lipoprotein.

10.1136/bmjopen-2016-012044.supp1 supplementary file

10.1136/bmjopen-2016-012044.supp2 supplementary file

10.1136/bmjopen-2016-012044.supp3 supplementary file

Follow-up for cohort A will start on the first date a patient is diagnosed with the condition the treatment is used for or ‘at risk’ of that condition (see table 2 and online supplementary files 1–3.) Follow-up will end with the earliest of a relevant neurodegenerative disease diagnosis, death or censoring due to the end of registration at a CPRD general practice.

#### Cohort B

In this analysis, we will compare different drug subclasses that exist for a given treatment (listed in box 1). To do this, we will create a cohort of all patients who were prescribed a treatment and categorise them by the drug subclass they initially received. This will allow us to compare the outcomes for each of the resulting subgroups. For each treatment, we have defined the most frequently prescribed subclass as the control (indicated in box 1). In the primary analysis of this cohort, we will exclude patients initially prescribed the control subclass if they receive another subclass of the same treatment at the same time, however we do not expect this to be a common. We will explore the outcomes of individuals prescribed multiple subclasses, other than that specified above, in a sensitivity analysis.

Box 1 **The drug subclasses of interest for each treatment group with the control treatments indicated**

Treatments for hypertension

- Beta-adrenoceptor blocking drugs (control)
- Angiotensin-converting enzyme inhibitors
- Thiazides and related diuretics
- Calcium channel blockers
- Loop diuretics
- Alpha-adrenoceptor blocking drugs
- Centrally acting antihypertensive drugs
- Angiotensin-II receptor antagonists
- Vasodilator antihypertensive drugs
- Potassium-sparing diuretics and aldosterone antagonists

Treatments for hypercholesterolaemia

- Statins (control)
- Fibrates
- Bile acid sequestrants
- Omega-3 fatty acid compounds
- Ezetimibe
- Nicotinic acid group

Treatments for type 2 diabetes

- Biguanides (control)
- Sulphonylureas
- Other antidiabetic drugs

Follow-up for cohort B will start on the date of first eligible prescription as determined by a product code (see the treatment code lists in online supplementary files 1–3). Follow-up will end with the earliest of a relevant neurodegenerative disease diagnosis, death or censoring due to the end of registration at a CPRD general practice.

#### Cohort C

For each neurodegenerative disease group of interest, we will create a cohort of individuals with a read code indicating this outcome (see online supplementary file 4) or a product code indicating they received treatment for the outcome (see online supplementary file 5). These cohorts will be used to investigate the associations of neurodegenerative disease progression with exposure to one of the three treatment groups of interest: treatments for hypertension, treatments for hypercholesterolaemia and treatments for type 2 diabetes. We will investigate both exposure before and after the diagnosis of a neurodegenerative disease. For all of the treatments we consider, each cohort will be split into ‘exposed’ and ‘unexposed’ groups. In the analysis, individuals in the ‘exposed’ group, that is, those who were prescribed the treatment under investigation, will be compared with individuals in the ‘unexposed’ groups, that is, those who did not receive the treatment.

10.1136/bmjopen-2016-012044.supp4 supplementary file

10.1136/bmjopen-2016-012044.supp5 supplementary file

Follow-up for cohort C will start with the first recorded read code indicating AD or other neurodegenerative disease (see online supplementary file 4) or, in the absence of a diagnosis, the first recorded product code indicating they received treatment for a neurodegenerative disease (see online supplementary file 5). Follow-up will end with the earliest of death or censoring due to the end of registration at a CPRD general practice.

### Variables

#### Exposures

For our primary analysis, we will define all exposures based on the first prescription observed in the database.^10^ This is so that the target parameter estimated in the observational study will be comparable to that estimated by a randomised controlled trial (RCT). Analogous to an intention-to-treat analysis in a RCT, patients initially prescribed an active treatment medication (exposed), but later stop that medication or switch to a control drug (unexposed), will be allocated to treatment (ie, classified as exposed, irrespective of what happens in the future) and vice versa. First time prescriptions of the medications of interest (treatment and control) will be defined as people who received at least one prescription of the product but who had no use of a related product during the 12 months before the start of follow-up. The prescriptions will be identified using product codes recorded in the therapy file in the CPRD, which details the date each prescription was issued, the quantity of drug prescribed and the dosage. Patients initially receiving more than one prescription of a drug of interest will be included in our analysis in a group representing the specific combination of drugs they receive. The effects of treatment switching and disease control will be explored using marginal structural models. We will also consider disease severity for the treatment indications through the use of instrumental variable analysis.

#### Outcomes

For incidence, we will identify the neurodegenerative disease outcomes (AD, NADD, PD and ALS) by the first record of a relevant read code (see online supplementary file 4) or the first record of a product code indicating treatment for the neurodegenerative disease (see online supplementary file 5). For incidence and progression, we will identify deaths using linked Office of National Statistics (ONS) data.

#### Covariates

We will match all covariates on date of birth, sex, year of index date and years of recorded history in the database before the index date. The covariates we will include in our analyses are body mass index, smoking status, alcohol consumption, a postcode-based measure of socioeconomic position, previous history of coronary-artery disease, previous coronary-bypass surgery and cerebrovascular disease including stroke. We will also control for other major chronic illness (including cancer, arthritis) using the Charlson Index^11^ ^12^ and for consultation rate, calculated by dividing the total number of clinic visits prior to the index date by the length of each patient's follow-up. If there are missing data in the covariates, we will consider using multiple imputation. Unfortunately not all covariates of interest, such as exercise, are recorded within the CPRD, and this may lead to unmeasured confounding. We hope to minimise this confounding by using methods, such as instrumental variable analysis, which can account for it to some degree.

### Data sources

The main data source for this project is the CPRD. Cause-specific mortality from linked ONS data is more accurate than CPRD data on mortality from general practices.^13^ Therefore, we will use the ONS data to identify date and cause of death and test our hypotheses relating to mortality using data from linked practices only. We will also be using the Index of Multiple Deprivation (IMD) in order to define socioeconomic position, which is listed as a covariate in our analysis.

### Study size

table 3 details the expected number of events for the event used to define the start of follow-up with the minimum sample size and detectable HR (α=0.05, β=0.80) for the Cox regression analysis of cohorts B and C. The minimum detectable HR is taken to be the detectable HR for the smallest exposed group tested against the control group. The minimum sample size is the sum of patients in the smallest exposed group and the control group. It is difficult to provide such details for cohort A due to its novel design that includes patients who are ‘at risk’ of the condition the treatment is used for. We can however consider the expected number of events listed for cohort B to be conservative estimates for the sample size of cohort A as all patients receiving treatment are included in cohort A by definition. This study will involve a large sample of data and has considerable power to detect even relatively small effects. Therefore, our statistical power will be more than adequate to test all of our proposed hypotheses, even under highly conservative Bonferroni corrections. Further details concerning the sample size and detectable HRs (α=0.05, β=0.80) for specific comparisons within cohorts B and C can be found in online supplementary files 6 and 7. Further details concerning the expected number of events, including at the drug subclass level, can be found in online supplementary file 8.

**Table 3:** The expected number of events for the event used to define the start of follow-up presented with the minimum sample size and detectable HR (α=0.05, β=0.80) for the Cox regression analysis of cohorts B and C

Cohort	Start of follow-up	Expected number of events	Minimum sample size	Minimum detectable HR
B	Treatment for hypertension	1 018 519	269 808	0.968
	Treatment for hypercholesterolaemia	808 687	788 479	0.844
	Treatment for type 2 diabetes	200 800	158 775	0.943
C	Diagnosis of dementia (AD and NADD)	105 471	105 471	0.931
	Diagnosis of PD	20 686	20 686	0.870
	Diagnosis of ALS	2227	2227	0.600

AD, Alzheimer's disease; ALS, amyotrophic lateral sclerosis; NADD, Non-Alzheimer's disease dementias.

10.1136/bmjopen-2016-012044.supp6 supplementary file

10.1136/bmjopen-2016-012044.supp7 supplementary file

10.1136/bmjopen-2016-012044.supp8 supplementary file

### Bias

There are three main sources of bias in our results: ascertainment bias, collider bias and immortal time bias. To overcome ascertainment bias, we will control for consultation rate in our analyses because people who have more chronic conditions such as hypertension, diabetes or renal insufficiency may have higher rates of consultation, which may also increase the opportunity for recording other diagnoses such as dementia. Collider bias could occur if we conditioned on events that happened as a result of the prescription the patient was issued. To prevent this form of bias from affecting our results, we will define each covariate using data inputted prior to the index date.^14^ Finally, we will minimise the risk of immortal time bias in two ways. First, to prevent misclassified immortal time, we will define exposures based on the first prescription observed in the database^10^ and use an intention-to-treat analysis. Second, to minimise immortal time, we will follow-up patients (exposed and unexposed) from a consistent index date. This will be a test result or a read code indicating diagnosis where possible;^15^ however, to capture all relevant patients, we will allow those receiving treatment without a recorded diagnosis to be included.

### Confounding

We have identified two key sources of confounding in our study: confounding by indication and time-dependent confounding. To address the former, we will use four different statistical methods, which have different approaches for modelling confounding. Through examination of these approaches, we hope to highlight any uncontrolled confounding that may be leading to false conclusions about drug effect. We will also account for confounding by indication in the construction of our cohorts. Consider cohort A, where we compare ‘exposed’ and ‘unexposed’ groups of individuals. Drawing the ‘unexposed’ group from the full population can introduce bias as they are likely to be in better health. We will avoid this by defining cohort entry on the basis of first test or recording of a relevant read code so the ‘exposed’ and ‘unexposed’ groups will have more in common. In addition to this, we will address time-dependent confounding in our study. This will be carried out using marginal structural models, which can allow for time-dependent confounding and treatment switching between products.

### Missing data

We will automatically use multiple imputation if there are missing data, as we are aware that a small amount of missing data across multiple variables could result in a large number of incomplete cases and consequently limit the sample for a complete case analysis. We will conduct sensitivity analyses to investigate the effect of imputing these missing data on our results.^16–18^

### Multiple testing

We will account for multiple testing using permutation analysis where appropriate. A portion of the data included in this project was previously used in a study investigating the association of α-adrenoceptor blocking drugs and angiotensin-II receptor antagonists with AD and non-AD dementias.^19^ We anticipate the overlap between our study and this previous study to be around 4% for each of cohorts A and B and 9% for cohort C. The authors acknowledge that this might be a concern for multiple testing, however wish to highlight that the analyses conducted in this study will differ from those used before.

### Statistical methods

We will use four statistical approaches in our study—they are multivariable adjusted Cox regression, propensity matched regression, instrumental variable analysis and marginal structural models. We are committed to presenting the findings from all our analyses, irrespective of the direction of findings and will handle discrepancies between them by assessing the merits of each method in the given situation. If we discover large main effects of specific medications, we may investigate interactions between covariates and will seek to replicate any interactions in other large samples.

We will be using an intention to treat analysis in our primary analysis. The reasons for this are twofold. First, while there are theoretical statistical models for estimating the effects of treatment switching such as marginal structural models, these methods require the strong assumption that there are no unmeasured confounders. Second, to the best of our knowledge there are no instrumental variable methods for estimating the effects of treatment switching. We will investigate treatment switching using marginal structural models in a secondary analysis. All of the methods we will use for this study are summarised below.

#### Multivariable adjusted Cox regression

In our first analyses, a conventional observational analysis, we will estimate the HRs of incidence and survival using Cox proportional hazards models. Both analyses will use the actual prescriptions issued to the patients.^20^ We will report these associations adjusted for basic confounders (age and gender) and results adjusted for all measured covariates described previously. We will also perform tests of the proportional hazards assumption.

#### Propensity matched regression

In our second analysis, we will construct a sample of patients balanced on covariates and risk factors using a propensity score.^21–24^ We will construct propensity scores using a logistic regression of the actual treatment received on the covariates described above. Therefore, each participant's propensity score will be their conditional probability (odds) of receiving treatment or not (cohort types A and C), or receiving treatment versus control therapy (cohort type B). We will match each patient receiving one treatment to another patient receiving the control treatment with the closest propensity score on a ratio of 1:1 using a nearest neighbour algorithm with no replacement, and matching will be restricted to the common support region. Patients outside the common support region are those prescribed the treatment therapy with propensity scores higher than any patient prescribed the control treatment and vice versa. We will estimate odds ratios and HRs of the outcomes using the propensity score matched sample using logistic and Cox regressions.

#### Instrumental variable analysis

In our third analysis, we will estimate the effects: (1) treatment or no treatment and (2) the specific subclass of medication prescribed, using physicians' prescribing preferences as instruments for the prescriptions the GPs issue to their patients. We cannot directly measure the physicians' preferences; therefore, we will use the prescriptions they issued to their previous patients as a proxy for their preferences. For example, if the instrument were based on just one previous prescription, physicians who previously prescribed the treatment therapy would be categorised as a ‘treatment prescriber’ otherwise they would be categorised as a ‘control prescriber’. As with our previous studies we will use seven prior prescriptions to improve the strength of the instruments.^25–27^ Using multiple prior prescriptions will maximise power. We will report risk differences in the outcomes using additive structural mean models estimated via the generalised method of moments.^28–30^ We will categorise each of the adverse event outcomes as occurring within 3, 6, 9, 12, 24 and 48 months of index date. Methods for estimating survival models using instrumental variables are not well developed. Therefore, we will explore potential instrumental variable survival models in a secondary analysis.^31^

#### Marginal structural models

In our fourth analysis, we will estimate the effects of each treatment using marginal structural models.^32^ We will use these models to account for time-dependent confounding and treatment switching. We will construct inverse probability weights for each treatment based on the patients' observed characteristics such as gender, age, comorbidities and concurrent treatments. We will use these models to estimate the odds and HRs of disease incidence and progression.

## Dissemination

We will publish the results of the study as open-access peer-reviewed publications. We will disseminate findings through national and international conferences as are appropriate. Depending on the findings, we would also explore additional options for focussed dissemination within appropriate communities, for example, via an Alzheimer's Society dissemination grant.
