# Supplementary material for: Can commonly prescribed drugs be repurposed for the prevention or treatment of Alzheimer's and other neurodegenerative diseases? Protocol for an observational cohort study in the UK Clinical Practice Research Datalink
Source: BMJ Open. 2016 Dec 12;6(12):e012044. doi: 10.1136/bmjopen-2016-012044 (PMC5168636; doi:10.1136/bmjopen-2016-012044)
Supplement: supplementary file [file bmjopen-2016-012044supp1.pdf]

Medical code list: 'at risk' of hypertension

Read Code	Read Code
1JD..00	Suspected hypertension
662Q.00	Borderline blood pressure
68B4.00	Risk factors present at hypertension screen
R1y2.00	[D]Raised blood pressure reading

Medical code list: hypertension

Read Code	Read Code
662O.00	On treatment for hypertension
7Q01.00	High cost hypertension drugs
7Q01y00	Other specified high cost hypertension drugs
G20..00	Essential hypertension
G20..11	High blood pressure
G20..12	Primary hypertension
G200.00	Malignant essential hypertension
G201.00	Benign essential hypertension
G202.00	Systolic hypertension
G203.00	Diastolic hypertension
G20z.00	Essential hypertension NOS
G20z.11	Hypertension NOS
G25..00	Stage 1 hypertension (NICE - Nat Ins for Hth Clin Excl 2011)
G25..11	Stage 1 hypertension
G26..00	Severe hypertension (Nat Inst for Health Clinical Ex 2011)
G26..11	Severe hypertension
G28..00	Stage 2 hypertension (NICE - Nat Ins for Hth Clin Excl 2011)

Product code list: treatments for hypertension

Product Code	Product Name
2	Bendroflumethiazide 2.5mg tablets
5	Atenolol 50mg tablets
6	Furosemide 40mg tablets
24	Atenolol 100mg tablets
26	Atenolol 25mg tablets
29	Amlodipine besilate 5mg tablets
55	Furosemide 20mg tablets
56	Co-amilofruse 5mg/40mg tablets
58	Bendroflumethiazide 5mg tablets
65	Lisinopril 10mg tablets
69	Lisinopril 20mg tablets
71	Amlodipine besilate 10mg tablets
78	Lisinopril 5mg tablets
80	Ramipril 5mg capsules
82	Ramipril 10mg capsules
97	Perindopril erbumine 4mg tablets
119	Doxazosin 1mg tablets
147	Ramipril 1.25mg capsules
193	Co-amilofruse 2.5mg/20mg tablets
196	Enalapril 5mg tablets
197	Atenolol 5mg/10ml solution for injection ampoules
211	Frumil 40mg+5mg Tablet (Helios Healthcare Ltd)
219	Diltiazem 120mg modified-release tablets
220	Propranolol 5mg/5ml oral solution
269	Nifedipine 5mg capsules
277	Lisinopril 2.5mg tablets
297	Propranolol 10mg tablets
338	Clonidine 25microgram tablets
348	Moduretic Tablet (Bristol-Myers Squibb Pharmaceuticals Ltd)
410	Nifedipine 10mg modified-release tablets
445	Prazosin 1mg tablets and Prazosin 500microgram tablets
448	Enalapril 2.5mg tablets
452	Nifedipine 10mg capsules
460	Flomax MR 400microgram capsules (Astellas Pharma Ltd)
472	Bisoprolol 5mg tablets
491	Felodipine 2.5mg modified-release tablets
493	Doxazosin 2mg tablets
501	Felodipine 5mg modified-release tablets
504	Hydralazine 20mg powder for solution for injection ampoules
517	Adizem sr 120mg Modified-release capsule (Napp Pharmaceuticals Ltd)
520	Losartan 25mg tablets
529	Candesartan 2mg tablets
531	Candesartan 4mg tablets

536	Tildiem la 200mg Modified-release capsule (Sanofi)
541	Adalat LA 20 tablets (Bayer Plc)
542	Hydrochlorothiazide 25mg tablets
562	Furosemide 10mg/ml Injection
568	Felodipine 10mg modified-release tablets
573	Hydralazine 25mg tablets
575	Valsartan 40mg capsules
581	Atenolol 50mg with Chlortalidone 12.5mg tablets
582	Doxazosin 4mg modified-release tablets
591	Prazosin 1mg tablets
593	Perindopril erbumine 2mg tablets
594	Bisoprolol 2.5mg tablets
599	Bisoprolol 1.25mg tablets
605	Chlortalidone 50mg tablets
624	Losartan 100mg tablets
633	Fosinopril 10mg tablets
634	Tamsulosin 400microgram modified-release capsules
636	Diltiazem 60mg modified-release capsules
654	Ramipril 2.5/5mg/10mg capsule
662	Adalat 5mg capsules (Bayer Plc)
692	Spironolactone 25mg tablets
700	Vera-Til SR 120mg tablets (Tillomed Laboratories Ltd)
707	Propranolol 40mg tablets
708	Spironolactone 50mg tablets
709	Ramipril 2.5mg capsules
726	Prazosin 2mg tablets
729	Amlodipine maleate 5mg tablets
737	Nifedipine 20mg modified-release capsules
739	Metoprolol 50mg tablets
749	Amlodipine 5mg tablets
751	Nebivolol 5mg tablets
753	Metoprolol 100mg tablets
755	Cardura XL 4mg tablets (Pfizer Ltd)
756	Ramipril 10mg tablets
761	Ramipril 1.25mg tablets
764	Co-Diovan 80mg/12.5mg tablets (Novartis Pharmaceuticals UK Ltd)
769	Propranolol 80mg modified-release capsules
786	Sotalol 40mg tablets
787	Spironolactone 100mg capsule
793	Adizem xl 240mg Capsule (Napp Pharmaceuticals Ltd)
814	Bumetanide 1mg tablets
817	Carvedilol 3.125mg tablets
822	Bisoprolol 1.5mg/5ml oral suspension
828	Irbesartan 75mg tablets
923	Co-amilozide 5mg/50mg tablets

924	Co-amilozide 2.5mg/25mg tablets
939	Tildiem Retard 90mg tablets (Sanofi)
940	Propranolol 80mg tablets
1006	Half Inderal LA 80mg capsules (AstraZeneca UK Ltd)
1021	Innozide 20mg/12.5mg tablets (Merck Sharp & Dohme Ltd)
1048	Inderal 80mg tablets (AstraZeneca UK Ltd)
1050	Inderal 40mg tablets (AstraZeneca UK Ltd)
1060	Amiloride 5mg tablets
1118	Verapamil 40mg tablets
1120	Verapamil 80mg tablets
1121	Captopril 12.5mg tablets
1124	Tenoretic 100mg/25mg tablets (AstraZeneca UK Ltd)
1125	Navidrex -k Tablet (Novartis Pharmaceuticals UK Ltd)
1130	Viazem XL 300mg capsules (Thornton & Ross Ltd)
1143	Captopril 25mg tablets
1144	Capoten 25mg tablets (Bristol-Myers Squibb Pharmaceuticals Ltd)
1170	Cyclopenthiazide 500microgram tablets
1209	Neo-Naclex 5mg tablets (Mercury Pharma Group Ltd)
1211	Bendroflumethiazide 2.5mg / Potassium chloride 630mg (potassium 8.4mmol) modified-release tablets
1213	Neo-Naclex-K modified-release tablets (Mercury Pharma Group Ltd)
1251	Moduret 25 tablets (Merck Sharp & Dohme Ltd)
1262	Nifedipine 12 20mg Modified-release tablet
1288	Tenoret 50mg/12.5mg tablets (AstraZeneca UK Ltd)
1289	Tildiem Retard 120mg tablets (Sanofi)
1290	Bisoprolol 10mg tablets
1292	Hypovase 1mg tablets (Pfizer Ltd)
1293	Irbesartan 150mg tablets
1294	Doxazosin 4mg tablets
1295	Labetalol 400mg tablets
1296	Hydralazine 50mg tablets
1297	Aldactide 50 tablets (Pfizer Ltd)
1298	Verapamil 240mg modified-release tablets
1299	Enalapril 10mg tablets
1300	Nifensar xl 20mg Modified-release tablet (Rhone-Poulenc Rorer Ltd)
1301	Frumil ls 20mg+2.5mg Tablet (Helios Healthcare Ltd)
1333	Oxprenolol 40mg tablets
1334	Oxprenolol 160mg modified-release tablets
1369	Furosemide with amiloride 40mg+5mg Tablet
1448	Propranolol 160mg modified-release capsules
1449	Nifedipine 24 30mg Modified-release tablet
1455	Prazosin 500microgram tablets
1520	Capozide 25mg/50mg tablets (Bristol-Myers Squibb Pharmaceuticals Ltd)
1529	Posicor 50mg Tablet (Roche Products Ltd)
1538	Diltiazem 60mg tablets

1572	Sotalol 80mg tablets
1574	Verapamil 120mg modified-release capsules
1597	Labetalol 100mg tablets
1684	Beta-Adalat modified-release capsules (Bayer Plc)
1686	Diltiazem 90mg modified-release capsules
1707	Methyldopa 250mg tablets
1721	Dyazide 50mg/25mg tablets (AMCo)
1747	Verapamil 120mg tablets
1748	Cordilox 120mg tablets (IVAX Pharmaceuticals UK Ltd)
1780	Losartan 50mg tablets
1788	Atenolol 100mg with Chlortalidone 25mg tablets
1807	Captopril 50mg tablets
1836	Diltiazem 60mg modified-release tablets
1854	Adalat la 30mg Tablet (Bayer Plc)
1904	Enalapril 20mg tablets
1995	Diltiazem 12hr 120mg modified-release capsules
2001	Aldactide 25 tablets (Pfizer Ltd)
2002	Amiloride 5mg / hydrochlorothiazide 50mg tablets
2046	Navidrex 500microgram tablets (AMCo)
2104	Rauwiloid 2mg Tablet (3M Health Care Ltd)
2117	Doralese Tiltab 20mg tablets (Chemidex Pharma Ltd)
2142	Spironolactone 100mg tablets
2179	Triamterene 50mg capsules
2255	Navispare 2.5mg/250microgram tablets (AMCo)
2280	Adalat retard 10mg tablets (Bayer Plc)
2343	Adalat retard 20mg tablets (Bayer Plc)
2345	Hytrin BPH tablets starter pack (AMCo)
2346	Hytrin 5mg Tablet (Abbott Laboratories Ltd)
2347	Hytrin 10mg Tablet (Abbott Laboratories Ltd)
2348	Hytrin bph 10mg Tablet (Amdipharm Plc)
2361	Trasicor 80mg Tablet (Novartis Pharmaceuticals UK Ltd)
2362	Apresoline 25mg tablets (AMCo)
2389	Aldactone 25mg tablets (Pfizer Ltd)
2414	Inderal 10mg tablets (AstraZeneca UK Ltd)
2432	Tenormin LS 50mg tablets (AstraZeneca UK Ltd)
2453	Diltiazem 60mg modified-release capsules
2493	Burinex A 5mg/1mg tablets (LEO Pharma)
2495	Bumetanide with Amiloride tablets
2499	Nadolol 80mg tablets
2521	Adalat 10mg capsules (Bayer Plc)
2528	Slozem 120mg capsules (Merck Serono Ltd)
2587	Tenormin 100mg tablets (AstraZeneca UK Ltd)
2590	Tenormin 25mg tablets (AstraZeneca UK Ltd)
2592	Viazem XL 120mg capsules (Thornton & Ross Ltd)
2605	Nifedipine 10mg modified-release capsules

2612	Indapamide 2.5mg tablets
2629	Carvedilol 12.5mg tablets
2663	Diltiazem 240mg modified-release capsules
2680	Apresoline 50mg Tablet (Sovereign Medical Ltd)
2686	Dilzem xl mr 240mg Modified-release capsule (Elan Pharma)
2746	Coracten SR 10mg capsules (UCB Pharma Ltd)
2772	Lasoride 5mg/40mg tablets (Sanofi)
2775	Labetalol 200mg tablets
2780	Oxprenolol 80mg tablets
2788	Burinex 1mg tablets (LEO Pharma)
2811	Adizem sr 180mg Modified-release capsule (Napp Pharmaceuticals Ltd)
2816	Indoramin 20mg tablets
2833	CYCLOPENTHIAZIDE -K tablets
2878	Clonidine 100microgram tablets
2888	Tildiem 60mg modified-release tablets (Sanofi)
2926	Nicardipine 20mg capsules
2961	Frusene 50mg/40mg tablets (Orion Pharma (UK) Ltd)
2967	Minoxidil 5mg tablets
2968	Minoxidil 10mg tablets
2970	Minoxidil 2.5mg tablets
2971	Irbesartan 300mg tablets
2979	Centyl k Tablet (Edwin Burgess Ltd)
2982	Zestoretic 20- 20mg+12.5mg Tablet (AstraZeneca UK Ltd)
3005	Inderal LA 160mg capsules (AstraZeneca UK Ltd)
3049	Methyldopa 125mg tablets
3050	Furosemide with triamterene 40mgwith50mg Tablet
3054	Hygroton 100mg Tablet (Alliance Pharmaceuticals Ltd)
3056	Natrilix SR 1.5mg tablets (Servier Laboratories Ltd)
3057	Securon 120mg tablets (Abbott Laboratories Ltd)
3061	Diltiazem 12hr 180mg modified-release capsules
3069	Acepril 25mg tablets (Bristol-Myers Squibb Pharmaceuticals Ltd)
3070	Methyldopa 500mg tablets
3087	Propranolol 40mg/5ml oral solution sugar free
3118	Adizem sr 90mg Modified-release capsule (Napp Pharmaceuticals Ltd)
3167	Propranolol 160mg tablets
3203	Capozide LS Tablet (E R Squibb and Sons Ltd)
3221	Lacidipine 4mg tablets
3222	Valsartan 80mg capsules
3248	Furosemide 500mg tablets
3287	Furosemide 1mg/ml Oral solution
3293	Moduretic Oral solution (Bristol-Myers Squibb Pharmaceuticals Ltd)
3302	Cardene SR 30mg capsules (Astellas Pharma Ltd)
3310	Capoten 12.5mg tablets (Bristol-Myers Squibb Pharmaceuticals Ltd)
3342	Securon SR 240mg tablets (Abbott Laboratories Ltd)
3343	Half Securon SR 120mg tablets (Abbott Laboratories Ltd)

3344	Betaloc 100mg tablets (AstraZeneca UK Ltd)
3370	Dilzem xl mr 120mg Modified-release capsule (Elan Pharma)
3470	Terazosin 1mg tablets
3474	Betaloc-SA 200mg tablets (AstraZeneca UK Ltd)
3516	Oxprenolol 20mg tablets
3517	Hydrochlorothiazide 50mg tablets
3526	Amiloride with atenolol with hydrochlorothiazide capsules
3548	Chlortalidone 100mg tablets
3588	Monocor 5mg tablets (Wyeth Pharmaceuticals)
3676	Dilzem xl mr 180mg Modified-release capsule (Elan Pharma)
3691	Sotalol 160mg with hydrochlorothiazide 25mg tablet
3701	Amiloride 2.5mg / hydrochlorothiazide 25mg tablets
3711	Adipine MR 20 tablets (Chiesi Ltd)
3712	Coracten XL 30mg capsules (UCB Pharma Ltd)
3715	Prazosin 5mg tablets
3720	Zestril 2.5mg tablets (AstraZeneca UK Ltd)
3748	Oxprenolol 160mg Tablet
3793	Co-amilofruse 10mg/80mg tablets
3827	Propanix 40mg Tablet (Ashbourne Pharmaceuticals Ltd)
3839	Capoten 50mg tablets (Bristol-Myers Squibb Pharmaceuticals Ltd)
3917	Istin 5mg tablets (Pfizer Ltd)
3923	Terazosin BPH starter pack 7x1mg with 14x2mg with 7x5mg
3924	Terazosin 5mg tablets
3929	Quinapril 10mg tablets
3930	Nifedipine 60mg modified-release tablets
3931	Posicor 100mg Tablet (Roche Products Ltd)
3943	Verapamil 240mg modified-release capsules
3997	Hygroton 50mg tablets (Alliance Pharmaceuticals Ltd)
4004	Sotacor 80mg tablets (Bristol-Myers Squibb Pharmaceuticals Ltd)
4025	Slow-Trasicor 160mg tablets (AMCo)
4034	Amiloride 5mg / hydrochlorothiazide 50mg/5ml solution
4044	Diurexan 20mg tablets (Meda Pharmaceuticals Ltd)
4068	Dytac 50mg capsules (AMCo)
4103	Trandolapril 1mg capsules
4111	Hypovase 500microgram tablets (Pfizer Ltd)
4155	Amias 2mg tablets (Takeda UK Ltd)
4161	Spiroctan 25mg Tablet (Roche Products Ltd)
4182	Lasix 5mg/5ml oral solution (Borg Medicare)
4211	Furosemide with amiloride 20mg+2.5mg Tablet
4215	Catapres 100microgram tablets (Boehringer Ingelheim Ltd)
4226	Cozaar 25mg tablets (Merck Sharp & Dohme Ltd)
4227	Adalat la 60mg Tablet (Bayer Plc)
4239	Adipine MR 10 tablets (Chiesi Ltd)
4258	Lasix 20mg/2ml solution for injection ampoules (Sanofi)
4265	Celectol 200mg Tablet (Pantheon Healthcare Ltd)

4308	Dilzem sr 90mg Capsule (Elan Pharma)
4332	Metolazone 5mg tablets
4334	Metolazone 500microgram low dose Tablet
4374	Debrisoquine 20mg tablets
4406	Decaserpyl plus Tablet (Roussel Laboratories Ltd)
4408	Slozem 240mg capsules (Merck Serono Ltd)
4410	Carvedilol 6.25mg tablets
4429	Trasidrex modified-release tablets (Mercury Pharma Group Ltd)
4449	Cardura 1mg tablets (Pfizer Ltd)
4540	Cozaar-Comp 50mg/12.5mg tablets (Merck Sharp & Dohme Ltd)
4542	Atenolol 50mg / Nifedipine 20mg modified-release capsules
4571	Staril 10mg tablets (Bristol-Myers Squibb Pharmaceuticals Ltd)
4588	Visken 5mg Tablet (Sovereign Medical Ltd)
4605	Moducren tablets (Merck Sharp & Dohme Ltd)
4635	Diltiazem 200mg modified-release capsules
4637	Terazosin 2mg tablets
4645	Valsartan 160mg capsules
4661	Spironolactone 50mg / Furosemide 20mg capsules
4685	Amias 4mg tablets (Takeda UK Ltd)
4694	Terazosin 2mg tablets and Terazosin 1mg tablets
4705	Furosemide 20mg/2ml Injection
4725	Labetalol 50mg tablets
4732	Diltiazem 90mg modified-release tablets
4741	Candesartan 16mg tablets
4771	Emcor LS 5mg tablets (Merck Serono Ltd)
4796	Inderetic 80mg/2.5mg capsules (AstraZeneca UK Ltd)
4802	Cardura 2mg tablets (Pfizer Ltd)
4808	Diltiazem 240mg modified-release capsules
4818	Candesartan 8mg tablets
4852	Adizem sr 120mg Modified-release tablet (Napp Pharmaceuticals Ltd)
4856	Coracten SR 20mg capsules (UCB Pharma Ltd)
4873	Fru-Co 5mg/40mg tablets (Teva UK Ltd)
4875	Terazosin 10mg tablets
4923	Diltiazem 24hr 180mg modified-release capsules
4939	Coracten XL 60mg capsules (UCB Pharma Ltd)
4960	Aldactone 50mg tablets (Pfizer Ltd)
4983	Atenolol with amiloride and hydrochlorothiazide capsules
4993	Moxonidine 200microgram tablets
5013	Amias 8mg tablets (Takeda UK Ltd)
5047	Trandolapril 2mg capsules
5054	Angitil SR 180 capsules (Chiesi Ltd)
5112	Indapamide 1.5mg modified-release tablets
5117	Amias 16mg tablets (Takeda UK Ltd)
5158	Lacidipine 2mg tablets
5159	Quinapril 20mg tablets

5162	Nifedipine 30mg modified-release capsules
5181	Angiopine MR 20mg tablets (Ashbourne Pharmaceuticals Ltd)
5183	Hypovase 2mg tablets (Pfizer Ltd)
5189	Enalapril 20mg / Hydrochlorothiazide 12.5mg tablets
5194	Dilzem sr 120mg Capsule (Elan Pharma)
5218	Bumetanide 1mg/5ml oral solution sugar free
5220	Furosemide with amiloride 80mg+10mg Tablet
5234	Slozem 180mg capsules (Merck Serono Ltd)
5249	Furosemide 50mg/5ml oral solution sugar free
5275	Tritace 2.5mg capsules (Sanofi)
5277	Fortipine LA 40 tablets (AMCo)
5284	Pindolol 5mg tablets
5289	Clonidine 250microgram modified-release capsules
5296	Tildiem la 300mg Modified-release capsule (Sanofi)
5326	Diltiazem 24hr 300mg modified-release capsules
5330	Corgaretic 40mg tablets (Sanofi-Synthelabo Ltd)
5337	Hytrin bph 5mg Tablet (Amdipharm Plc)
5348	Diltiazem 300mg modified-release capsules
5416	Co-triamterzide 50mg/25mg tablets
5477	Nicardipine 30mg modified-release capsules
5478	Propranolol 10mg/5ml oral solution sugar free
5496	Doxazosin 8mg modified-release tablets
5513	Dilzem sr 60mg Capsule (Elan Pharma)
5570	Zanidip 10mg tablets (Recordati Pharmaceuticals Ltd)
5593	Lercanidipine 10mg tablets
5612	Coversyl 2mg tablets (Servier Laboratories Ltd)
5618	Cardura XL 8mg tablets (Pfizer Ltd)
5713	Bisoprolol 7.5mg tablets
5721	Co-tenidone 100mg/25mg tablets
5723	Cozaar 50mg tablets (Merck Sharp & Dohme Ltd)
5727	Amiloride 2.5mg / Cyclopenthiazide 250microgram tablets
5728	Furosemide 40mg/5ml oral solution sugar free
5735	Tritace 5mg capsules (Sanofi)
5800	Coversyl 4mg tablets (Servier Laboratories Ltd)
5806	Tensipine MR 20 tablets (Thornton & Ross Ltd)
5815	Indoramin 25mg tablets
5858	Beta-Cardone 40mg tablets (Focus Pharmaceuticals Ltd)
5861	Fosinopril 20mg tablets
5868	Frusol 20mg/5ml oral solution (Rosemont Pharmaceuticals Ltd)
5914	Istin 10mg tablets (Pfizer Ltd)
5968	Monocor 10mg tablets (Wyeth Pharmaceuticals)
5988	Telmisartan 40mg tablets
6008	Omnic 400microgram Modified-release capsule (Paines & Byrne Ltd)
6066	Atenolol 25mg/5ml oral solution sugar free
6078	Perindopril erbumine 8mg tablets

6118	Furosemide 20mg/5ml oral solution sugar free
6200	Tritace titration pack capsules (Sanofi)
6207	Tadalafil 20mg tablets
6217	Olmesartan medoxomil 10mg tablets
6243	Telmisartan 20mg tablets
6261	Tritace 1.25mg tablets (Sanofi)
6285	Olmesartan medoxomil 20mg tablets
6288	Ramipril 5mg tablets
6309	Adizem xl 300mg Capsule (Napp Pharmaceuticals Ltd)
6314	Ramipril 2.5mg tablets
6351	Olmesartan medoxomil 40mg tablets
6359	Zestoretic 10- 10mg+12.5mg Tablet (AstraZeneca UK Ltd)
6362	Tritace 5mg tablets (Sanofi)
6364	Tritace 2.5mg tablets (Sanofi)
6408	Tanatril 5mg tablets (Chiesi Ltd)
6437	Losartan 50mg / Hydrochlorothiazide 12.5mg tablets
6468	Lisinopril 20mg / Hydrochlorothiazide 12.5mg tablets
6477	Amlodipine maleate 10mg tablets
6510	Univer 120mg modified-release capsules (Teva UK Ltd)
6518	Diovan 160mg capsules (Novartis Pharmaceuticals UK Ltd)
6694	Clonidine 300microgram tablets
6751	Beta-Cardone 80mg tablets (Focus Pharmaceuticals Ltd)
6765	Quinapril 5mg tablets
6786	Lisinopril 10mg / Hydrochlorothiazide 12.5mg tablets
6794	Perindopril erbumine 4mg / Indapamide 1.25mg tablets
6806	Zestril 10mg tablets (AstraZeneca UK Ltd)
6807	Zestril 5mg tablets (AstraZeneca UK Ltd)
6815	Spironolactone 50mg/5ml oral suspension sugar free
6816	Chlorothiazide 250mg/5ml oral suspension
6856	Amlodipine 10mg tablets
6877	Co-Diovan 160mg/12.5mg tablets (Novartis Pharmaceuticals UK Ltd)
6939	Eprosartan 300mg tablets
7043	Candesartan 32mg tablets
7049	Carvedilol 25mg tablets
7056	Tamsulosin 400microgram modified-release tablets
7066	Metoprolol 100mg / Hydrochlorothiazide 12.5mg tablets
7091	Bisoprolol 3.75mg tablets
7136	Dytide capsules (Mercury Pharma Group Ltd)
7174	Moxonidine 400microgram tablets
7280	Plendil 10mg modified-release tablets (AstraZeneca UK Ltd)
7314	Accupro 5mg tablets (Pfizer Ltd)
7338	Aprovel 75mg tablets (Sanofi)
7351	Bendroflumethiazide 2.5mg/5ml oral suspension
7398	Viazem XL 360mg capsules (Thornton & Ross Ltd)
7416	Aldomet 50mg/ml Injection (Merck Sharp & Dohme Ltd)

7419	Trandolapril 500microgram capsules
7429	Tenormin 5mg/10ml solution for injection ampoules (AstraZeneca UK Ltd)
7441	Lasilactone 20mg/50mg capsules (Sanofi)
7474	Trasicor 20mg Tablet (Novartis Pharmaceuticals UK Ltd)
7528	Nebilet 5mg tablets (A. Menarini Farmaceutica Internazionale SRL)
7541	Nifopress Retard 20mg tablets (AMCo)
7543	Kalten capsules (M & A Pharmachem Ltd)
7547	Doxadura 2mg tablets (Discovery Pharmaceuticals Ltd)
7549	Doxadura 1mg tablets (Discovery Pharmaceuticals Ltd)
7553	Bisoprolol 5mg/5ml oral suspension
7562	Cardene 30mg capsules (Astellas Pharma Ltd)
7606	Lasix 40mg tablets (Sanofi)
7618	Xipamide 20mg tablets
7620	Acebutolol 400mg tablets
7626	Aldomet 250mg Tablet (Merck Sharp & Dohme Ltd)
7641	Natrilix 2.5mg tablets (Servier Laboratories Ltd)
7642	Aldomet 500mg Tablet (Merck Sharp & Dohme Ltd)
7681	Synadrin 60mg Tablet (Hoechst UK Ltd)
7698	Aprinox 5mg tablets (Amdipharm Plc)
7709	Arelix 6mg Capsule (Hoechst Marion Roussel)
7740	Triamterene 50mg / Benzthiazide 25mg capsules
7759	Phenoxybenzamine 10mg capsules
7799	Lasix 20mg tablets (Borg Medicare)
7806	Bumetanide 5mg tablets
7852	Blocadren 10mg Tablet (Merck Sharp & Dohme Ltd)
7853	Timolol 10mg tablets
7911	Declinax 10mg Tablet (Roche Products Ltd)
7922	Ismelin 10mg Tablet (Sovereign Medical Ltd)
7923	Guanethidine 10mg Tablet
7952	Aldactone 100mg tablets (Pfizer Ltd)
7961	Spironolactone 50mg with hydroflumethiazide 50mg tablet
7974	Celiprolol 400mg tablets
7991	Spiroctan 100mg Capsule (Roche Products Ltd)
8023	Sectral 400mg tablets (Sanofi)
8025	Gopten 1mg capsules (Abbott Laboratories Ltd)
8026	Gopten 2mg capsules (Abbott Laboratories Ltd)
8033	Aldomet 125mg Tablet (Merck Sharp & Dohme Ltd)
8052	Torasemide 5mg tablets
8058	Normetic Tablet (Abbott Laboratories Ltd)
8061	Sotalol 80mg with hydrochlorothiazide 12.5mg tablet
8068	Metoprolol 200mg modified-release tablets
8071	Betaloc 50mg tablets (AstraZeneca UK Ltd)
8076	Hytrin tablets starter pack (AMCo)
8077	Hytrin 2mg Tablet (Abbott Laboratories Ltd)
8086	Cardura 4mg Tablet (Pfizer Ltd)

8105	Innovace 20mg tablets (Merck Sharp & Dohme Ltd)
8106	Innovace 2.5mg tablets (Merck Sharp & Dohme Ltd)
8113	Acebutolol 200mg capsules
8147	Lopresoretic Tablet (Novartis Pharmaceuticals UK Ltd)
8172	Acebutolol 100mg capsules
8189	Secadrex 200mg/12.5mg tablets (Sanofi)
8198	Hypovase 5mg Tablet (Pfizer Ltd)
8201	Nicardipine 30mg capsules
8213	Nifedipine 24 20mg Modified-release tablet
8257	Prescal 2.5mg tablets (Novartis Pharmaceuticals UK Ltd)
8262	Celiprolol 200mg tablets
8268	Zestril 20mg tablets (AstraZeneca UK Ltd)
8290	Trasicor 40mg Tablet (Novartis Pharmaceuticals UK Ltd)
8296	Catapres PL Perlongets 250microgram capsules (Boehringer Ingelheim Ltd)
8303	Tenavoid Tablet (Edwin Burgess Ltd)
8310	Isradipine 2.5mg tablets
8331	Inderal 160mg Tablet (AstraZeneca UK Ltd)
8342	Declinax 20mg Tablet (Roche Products Ltd)
8369	Inderex 160mg/5mg modified-release capsules (AstraZeneca UK Ltd)
8464	Meprobamate with bendroflumethiazide Tablet
8521	Spironolactone 25mg with hydroflumethiazide 25mg tablet
8524	Securon 40mg Tablet (Abbott Laboratories Ltd)
8526	Aprinox 2.5mg tablets (AMCo)
8555	Sectral 200mg capsules (Sanofi)
8558	Adizem xl 120mg Capsule (Napp Pharmaceuticals Ltd)
8602	Metenix 5mg tablets (Sanofi)
8623	Prestim Tablet (ICN Pharmaceuticals France S.A.)
8642	Tenif 50mg/20mg modified-release capsules (AstraZeneca UK Ltd)
8673	Oxprenolol with cyclopenthiazide 160mg+0.25mg Modified-release tablet
8707	Trandate 200mg tablets (Focus Pharmaceuticals Ltd)
8759	Verapamil hcl 120mg modified release tablets
8800	Innovace 5mg tablets (Merck Sharp & Dohme Ltd)
8807	Trandate 400mg tablets (Focus Pharmaceuticals Ltd)
8830	Innovace 10mg tablets (Merck Sharp & Dohme Ltd)
8836	Chlorothiazide 500mg tablets
8863	Hypovase benign prostatic hyperplasia 1mg Tablet (Pfizer Ltd)
8884	Cordilox 40mg tablets (IVAX Pharmaceuticals UK Ltd)
8891	Hygroton -k Tablet (Novartis Pharmaceuticals UK Ltd)
8897	Triam-Co 50mg/25mg tablets (IVAX Pharmaceuticals UK Ltd)
8935	Nadolol 40mg tablets
8942	Dibenyline 10mg capsules (Mercury Pharma Group Ltd)
8945	Univer 240mg modified-release capsules (Teva UK Ltd)
8975	Verapamil 180mg modified-release capsules
8978	Propanix 160mg Modified-release capsule (Ashbourne Pharmaceuticals Ltd)
8987	Propranolol 160mg modified-release / Bendroflumethiazide 5mg capsules

9016	Trandate 100mg tablets (Focus Pharmaceuticals Ltd)
9019	Indoramin 50mg Tablet
9143	Viskaldix tablets (AMCo)
9178	Atenolol 25mg / Bendroflumethiazide 1.25mg capsules
9185	Propranolol 80mg/5ml oral solution
9196	Aprovel 150mg tablets (Sanofi)
9223	Triamterene with hydrochlorothiazide 50mg + 25mg Tablet
9225	Methyldopa 250mg Capsule
9240	Adizem xl 180mg Capsule (Napp Pharmaceuticals Ltd)
9269	Nifedipine 40mg modified-release tablets
9273	Trandate 50mg tablets (Focus Pharmaceuticals Ltd)
9292	Sotalol 160mg tablets
9334	Plendil 2.5mg modified-release tablets (AstraZeneca UK Ltd)
9374	Adizem 60mg Modified-release tablet (Napp Pharmaceuticals Ltd)
9386	Nicardipine 45mg modified-release capsules
9410	Angitil SR 120 capsules (Chiesi Ltd)
9431	Frusemek 40mg+5mg Tablet (Approved Prescription Services Ltd)
9437	Plendil 5mg modified-release tablets (AstraZeneca UK Ltd)
9456	Amiloride 5mg / furosemide 40mg tablets
9463	Loniten 5mg tablets (Pfizer Ltd)
9485	Hypolar Retard 20 tablets (Sandoz Ltd)
9553	Slofedipine XL 60 tablets (Zentiva)
9569	Verapamil 120mg modified-release tablets
9573	Slofedipine XL 30mg tablets (Zentiva)
9646	Tritace 1.25mg capsules (Aventis Pharma)
9670	Motens 4mg tablets (GlaxoSmithKline UK Ltd)
9680	Frusol 40mg/5ml oral solution (Rosemont Pharmaceuticals Ltd)
9693	Tritace 10mg capsules (Sanofi)
9697	Loniten 2.5mg tablets (Pfizer Ltd)
9708	Diltiazem 24hr 120mg modified-release capsules
9723	Calcicard CR 90mg tablets (Teva UK Ltd)
9731	Quinapril 40mg tablets
9745	Teveten 300mg tablets (Abbott Healthcare Products Ltd)
9749	Physiotens 400microgram tablets (Abbott Healthcare Products Ltd)
9750	Nifedipine 60mg modified-release capsules
9764	Carace 20 Tablet (Bristol-Myers Squibb Pharmaceuticals Ltd)
9783	Co-tenidone 50mg/12.5mg tablets
9876	Physiotens 300microgram tablets (Abbott Healthcare Products Ltd)
9915	Tritace 10mg tablets (Sanofi)
9935	Amiloride 5mg/5ml oral solution sugar free
9948	Trandolapril 4mg capsules
10066	Torem 5mg tablets (Meda Pharmaceuticals Ltd)
10088	Doxadura 4mg tablets (Discovery Pharmaceuticals Ltd)
10134	Flomaxtra XL 400microgram tablets (Astellas Pharma Ltd)
10135	Nifedipress mr 10mg Modified-release tablet (Sandoz Ltd)

10136	Nifedipress MR 20 tablets (Dexcel-Pharma Ltd)
10153	Felendil xl 5mg Modified-release tablet (Ratiopharm UK Ltd)
10191	Atenix 50 tablets (Ashbourne Pharmaceuticals Ltd)
10214	Spironolactone 5mg/5ml oral suspension sugar free
10246	Adipine XL 60mg tablets (Chiesi Ltd)
10251	Eplerenone 25mg tablets
10253	Moxonidine 300microgram tablets
10267	Adizem-XL 200mg capsules (Napp Pharmaceuticals Ltd)
10316	CoAprovel 150mg/12.5mg tablets (Sanofi)
10323	Losartan 100mg / Hydrochlorothiazide 25mg tablets
10392	Lasix 500mg tablets (Sanofi)
10422	Lasix 50mg/5ml Injection (Hoechst UK Ltd)
10429	Lopresor 50mg Tablet (Novartis Pharmaceuticals UK Ltd)
10595	Nimotop 30mg tablets (Bayer Plc)
10627	Co-Betaloc tablets (Pfizer Ltd)
10688	Verapamil 160mg tablets
10713	Decaserpyl 5mg Tablet (Roussel Laboratories Ltd)
10714	Methoserpidine 5mg Tablet
10716	Corgard 80mg tablets (Sanofi)
10777	Trasicor 160mg Tablet (Novartis Pharmaceuticals UK Ltd)
10832	Securon 80mg Tablet (Abbott Laboratories Ltd)
10879	Bethanidine sulphate 10mg tablets
10882	Carace 2.5mg tablets (Bristol-Myers Squibb Pharmaceuticals Ltd)
10892	Emcor 10mg tablets (Merck Serono Ltd)
10902	Captopril 50mg with Hydrochlorothiazide 25mg tablets
11133	Hydrochlorothiazide with captopril 25mg with 50mg Tablet
11156	Spirolone 25mg Tablet (Berk Pharmaceuticals Ltd)
11177	Physiotens 200microgram tablets (Abbott Healthcare Products Ltd)
11197	Innovace melt 5mg Wafer (Merck Sharp & Dohme Ltd)
11223	Angitil SR 90 capsules (Chiesi Ltd)
11251	Diovan 40mg capsules (Novartis Pharmaceuticals UK Ltd)
11252	Diovan 80mg capsules (Novartis Pharmaceuticals UK Ltd)
11265	Triamterene 50mg / Furosemide 40mg tablets
11268	Torem 2.5mg tablets (Meda Pharmaceuticals Ltd)
11338	Bendroflumethiazide 5mg with Nadolol 40mg tablets
11348	Aprovel 300mg tablets (Sanofi)
11351	Co-zidocapt 25mg/50mg tablets
11380	Sotacor 160mg tablets (Bristol-Myers Squibb Pharmaceuticals Ltd)
11384	Co-flumactone 50mg/50mg tablets
11394	Baratol 25mg Tablet (Shire Pharmaceuticals Ltd)
11448	Irbesartan 150mg / Hydrochlorothiazide 12.5mg tablets
11469	Irbesartan 300mg / Hydrochlorothiazide 12.5mg tablets
11487	Torasemide 2.5mg tablets
11512	Nifedipress MR 10 tablets (Dexcel-Pharma Ltd)
11519	Spironolactone 25mg/5ml oral suspension sugar free

11526	CoAprovel 300mg/12.5mg tablets (Sanofi)
11547	Nimodipine 30mg tablets
11561	Co-zidocapt 12.5mg/25mg tablets
11567	Ramipril 5mg with felodipine 5mg modified-release tablet
11641	Captopril 25mg with Hydrochlorothiazide 12.5mg tablets
11711	Propranolol 50mg/5ml oral solution
11769	Calchan MR 20 tablets (Ranbaxy (UK) Ltd)
11770	Dilzem SR 60 capsules (Teva UK Ltd)
11777	Verapamil 40mg/5ml oral solution sugar free
11793	Metoprolol 50mg/5ml oral suspension
11864	Valsartan 160mg / Hydrochlorothiazide 12.5mg tablets
11922	Diltiazem 60mg/5ml oral suspension
11937	Ramipril 2.5mg/5ml oral suspension
11943	Cardene 20mg capsules (Astellas Pharma Ltd)
11965	Ramipril 2.5mg with felodipine 2.5mg modified-release tablet
11966	Motens 2mg tablets (GlaxoSmithKline UK Ltd)
11972	Vertab SR 240 tablets (Chiesi Ltd)
11973	Calcicard CR 120mg tablets (Teva UK Ltd)
11983	Perindopril erbumine 4mg/5ml oral suspension
11987	Lisinopril 5mg/5ml oral solution
12037	Betim 10mg Tablet (ICN Pharmaceuticals France S.A.)
12054	Propranolol 80mg / Bendroflumethiazide 2.5mg capsules
12104	Cordilox 160mg tablets (IVAX Pharmaceuticals UK Ltd)
12110	Hydroflumethiazide 50mg Tablet
12141	Betaxolol 20mg tablets
12226	Burinex 1mg/5ml Oral solution (LEO Pharma)
12294	Burinex 5mg tablets (LEO Pharma)
12296	Sectral 100mg capsules (Sanofi)
12313	Carace 20mg tablets (Bristol-Myers Squibb Pharmaceuticals Ltd)
12318	Lasix 250mg/25ml Injection (Hoechst Marion Roussel)
12354	Etacrynic 50mg tablets
12360	Nephril 1mg Tablet (Pfizer Ltd)
12367	Piretanide 6mg capsule
12392	Univer 180mg modified-release capsules (Teva UK Ltd)
12411	Cilazapril 500microgram tablets
12412	Cilazapril 2.5mg tablets
12440	Hydrosaluric 25mg tablets (Merck Sharp & Dohme Ltd)
12456	Sotazide Tablet (Bristol-Myers Squibb Pharmaceuticals Ltd)
12495	Berkolol 10mg Tablet (Berk Pharmaceuticals Ltd)
12517	Timolol maleate with bendroflumethiazide 20mg + 5mg Tablet
12518	Hypovase tablets B.D. starter pack (Pfizer Ltd)
12519	Kerlone 20mg tablets (Sanofi-Synthelabo Ltd)
12545	Phentolamine 10mg/1ml solution for injection ampoules
12546	Kalspare Tablet (Dominion Pharma)
12547	Triamterene 50mg / Chlortalidone 50mg tablets

12574	Cilazapril 1mg tablets
12606	Nifelease 20mg Modified-release tablet (Eastern Pharmaceuticals Ltd)
12613	Unipine xl 30mg Modified-release tablet (Genus Pharmaceuticals Ltd)
12639	Diltiazem HCl 90mg Modified-release tablet (Actavis UK Ltd)
12651	Timolol 10mg / Bendroflumethiazide 2.5mg tablets
12705	Angiozem CR 90mg tablets (Ashbourne Pharmaceuticals Ltd)
12815	Tanatril 10mg tablets (Chiesi Ltd)
12836	Eprosartan 600mg tablets
12858	Imidapril 10mg tablets
12874	Telmisartan 80mg tablets
12875	Cardene SR 45mg capsules (Astellas Pharma Ltd)
12926	Polythiazide 1mg tablets
12946	Spironolactone 10mg/5ml oral suspension sugar free
13026	Cilazapril 5mg tablets
13027	Viazem XL 240mg capsules (Thornton & Ross Ltd)
13033	Angitil XL 240 capsules (Chiesi Ltd)
13051	Sotalol 200mg tablets
13075	Dilzem XL 180 capsules (Teva UK Ltd)
13123	Eprosartan 400mg tablets
13127	Dilzem XL 240 capsules (Teva UK Ltd)
13139	Adipine XL 30mg tablets (Chiesi Ltd)
13240	Dilzem XL 120 capsules (Teva UK Ltd)
13243	Lercanidipine 20mg tablets
13246	Chlorothiazide 150mg/5ml oral suspension
13251	Vera-Til SR 240mg tablets (Tillomed Laboratories Ltd)
13264	Spironolactone 15mg/5ml oral suspension
13302	Dilzem SR 90 capsules (Teva UK Ltd)
13317	Apresoline 20mg powder for solution for injection ampoules (AMCo)
13352	Midamor 5mg Tablet (MSD Thomas Morson Pharmaceuticals)
13363	Esidrex 50mg Tablet (Novartis Pharmaceuticals UK Ltd)
13379	Ismelin 25mg Tablet (Sovereign Medical Ltd)
13394	Tenormin 25mg/5ml syrup (AstraZeneca UK Ltd)
13410	Angiozem 60mg modified-release tablets (Ashbourne Pharmaceuticals Ltd)
13415	Corgard 40mg tablets (Sanofi-Synthelabo Ltd)
13435	Frumil Forte 10mg/80mg tablets (Sanofi)
13487	Beta-Cardone 200mg tablets (Focus Pharmaceuticals Ltd)
13499	Lopresor 100mg Tablet (Novartis Pharmaceuticals UK Ltd)
13525	Hydrenox 50mg Tablet (Knoll Ltd)
13526	Atenix Co 100 tablets (Ashbourne Pharmaceuticals Ltd)
13589	Staril 20mg tablets (Bristol-Myers Squibb Pharmaceuticals Ltd)
13610	Alphavase 2 tablets (Ashbourne Pharmaceuticals Ltd)
13672	Angiopine MR 10mg tablets (Ashbourne Pharmaceuticals Ltd)
13699	Angiopine la 40mg Tablet (Ashbourne Pharmaceuticals Ltd)
13755	Enalapril 10mg wafer
13821	Micardis 40mg tablets (Boehringer Ingelheim Ltd)

13856	Verapress MR 240mg tablets (Actavis UK Ltd)
13871	Co-prenozide 160mg/0.25mg modified-release tablets
13926	Diltiazem 360mg modified-release capsules
13965	Cordilox MR 240mg tablets (Teva UK Ltd)
14030	Cardicor 2.5mg tablets (Merck Serono Ltd)
14057	Pindolol 10mg / Clopamide 5mg tablets
14058	Cardicor 1.25mg tablets (Merck Serono Ltd)
14109	Spironolactone 100mg/5ml oral solution sugar free
14117	Eucardic 3.125mg tablets (Roche Products Ltd)
14126	Acebutolol 200mg / Hydrochlorothiazide 12.5mg tablets
14144	Inspra 25mg tablets (Pfizer Ltd)
14146	Eucardic 6.25mg tablets (Roche Products Ltd)
14228	Coversyl Plus tablets (Servier Laboratories Ltd)
14283	Valsartan 160mg / Hydrochlorothiazide 25mg tablets
14300	Zanidip 20mg tablets (Recordati Pharmaceuticals Ltd)
14305	Vascalpha 10mg modified-release tablets (Actavis UK Ltd)
14387	Carace 5mg tablets (Bristol-Myers Squibb Pharmaceuticals Ltd)
14390	Aldomet 250mg/5ml Liquid (Merck Sharp & Dohme Ltd)
14438	Corgaretic 80mg tablets (Sanofi-Synthelabo Ltd)
14442	Esbatal 50mg Tablet (Wellcome Medical Division)
14477	Accupro 10mg tablets (Pfizer Ltd)
14478	Accupro 20mg tablets (Pfizer Ltd)
14495	Loniten 10mg tablets (Pfizer Ltd)
14502	Metoprolol 5mg/5ml solution for injection ampoules
14552	Propanix 10mg Tablet (Ashbourne Pharmaceuticals Ltd)
14587	Amiloride 5mg / Bumetanide 1mg tablets
14673	Pindolol 15mg tablets
14738	Hydrochlorothiazide with losartan 12.5mg with 50mg Tablet
14761	Frusid 40mg tablets (Dr Reddy's Laboratories (UK) Ltd)
14808	Bedranol SR 80mg capsules (Sandoz Ltd)
14837	Frusol 50mg/5ml oral solution (Rosemont Pharmaceuticals Ltd)
14861	Calchan MR 10 tablets (Ranbaxy (UK) Ltd)
14870	Telmisartan 40mg / Hydrochlorothiazide 12.5mg tablets
14932	Tabphyn MR 400microgram capsules (ProStrakan Ltd)
14943	Valsartan 40mg tablets
14960	Coversyl 8mg tablets (Servier Laboratories Ltd)
14965	Cozaar 100mg tablets (Merck Sharp & Dohme Ltd)
14983	Olmetec 10mg tablets (Daiichi Sankyo UK Ltd)
15031	Accuretic 12.5mg/10mg tablets (Pfizer Ltd)
15042	Tolerzide Tablet (Bristol-Myers Squibb Pharmaceuticals Ltd)
15052	Spiroctan 50mg Tablet (Roche Products Ltd)
15085	Innovace Titration pack (Merck Sharp & Dohme Ltd)
15096	Accupro 40mg tablets (Pfizer Ltd)
15108	Quinapril 10mg / Hydrochlorothiazide 12.5mg tablets
15117	Nifedipine with atenolol 20mg + 50mg Capsule

15121	Moexipril 7.5mg tablets
15127	Hydrochlorothiazide with triamterene 25mgwith50mg Tablet
15135	Hydrochlorothiazide with captopril 12.5mg with 25mg Tablet
15176	Totamol 50mg Tablet (C P Pharmaceuticals Ltd)
15221	Dilcardia xl 180mg Modified-release capsule (Generics (UK) Ltd)
15288	Angitil XL 300 capsules (Chiesi Ltd)
15341	Burinex 0.5mg/ml Injection (LEO Pharma)
15457	Baycaron 25mg Tablet (Bayer Plc)
15488	Metoprolol tartrate with chlortalidone Tablet
15493	Reserpine with hydrochlorothiazide tablet
15605	Cilazapril 250micrograms tablets
15619	Half-betadur cr 80mg Capsule (Monmouth Pharmaceuticals Ltd)
15652	Mibefradil 50mg Tablet
15715	Genalat retard 20mg Modified-release tablet (Wyeth Pharmaceuticals)
15730	Totamol 100mg Tablet (C P Pharmaceuticals Ltd)
15811	Co-flumactone 25mg/25mg tablets
15874	Amiloride 2.5mg / furosemide 20mg tablets
15958	Captopril 2mg tablets
16038	Dilzem SR 120 capsules (Teva UK Ltd)
16060	Valsartan 80mg / Hydrochlorothiazide 12.5mg tablets
16073	Nifedipress MR 10 tablets (Teva UK Ltd)
16161	Telmisartan 80mg / Hydrochlorothiazide 12.5mg tablets
16162	Amlodipine 5mg/5ml oral suspension
16196	Vascace 5mg tablets (Roche Products Ltd)
16197	Vascace 2.5mg tablets (Roche Products Ltd)
16198	Baratol 50mg Tablet (Shire Pharmaceuticals Ltd)
16201	Hytrin bph 2mg Tablet (Amdipharm Plc)
16206	Froop 40mg tablets (Ashbourne Pharmaceuticals Ltd)
16212	Vascace 1mg tablets (Roche Products Ltd)
16248	Clonidine 150micrograms/1ml solution for injection ampoules
16285	Teveten 400mg tablets (Abbott Healthcare Products Ltd)
16328	Verapress MR 240mg tablets (Dexcel-Pharma Ltd)
16371	Teveten 600mg tablets (Abbott Healthcare Products Ltd)
16498	Kalspare tablets (DHP Healthcare Ltd)
16531	Eplerenone 50mg tablets
16632	Hydrosaluric 50mg tablets (Merck Sharp & Dohme Ltd)
16645	Labrocol 400mg Tablet (Lagap)
16677	Cordilox 80mg tablets (IVAX Pharmaceuticals UK Ltd)
16701	Carace 10mg tablets (Bristol-Myers Squibb Pharmaceuticals Ltd)
16708	Enalapril titration pack
16710	Gopten 500microgram capsules (Abbott Laboratories Ltd)
16776	Celectol 400mg Tablet (Pantheon Healthcare Ltd)
16786	Chlortalidone 25mg with Atenolol 100mg tablets
16850	Angiozem CR 120mg tablets (Ashbourne Pharmaceuticals Ltd)
16924	Imidapril 5mg tablets

17006	Triapin 5mg/5mg modified-release tablets (Sanofi)
17082	Syprol 5mg/5ml oral solution (Rosemont Pharmaceuticals Ltd)
17120	Moexipril 15mg tablets
17122	Isoprenaline 200micrograms/1ml solution for injection ampoules
17143	Mefruside 25mg Tablet
17149	Monozide 10 tablets (Wyeth Pharmaceuticals)
17252	Esidrex 25mg Tablet (Novartis Pharmaceuticals UK Ltd)
17291	Guanethidine 25mg Tablet
17322	Atenix 25 tablets (Ashbourne Pharmaceuticals Ltd)
17325	Cardilate MR 10mg tablets (Teva UK Ltd)
17338	Nifedotard 20 mr 20mg Modified-release tablet (Galen Ltd)
17342	Nivaten retard 10mg Modified-release tablet (Actavis UK Ltd)
17406	Zemtard 180 XL capsules (Galen Ltd)
17425	Zemtard 120 XL capsules (Galen Ltd)
17448	Nifedipress mr 10mg Modified-release tablet (Sterwin Medicines)
17462	Bisoprolol 10mg / Hydrochlorothiazide 6.25mg tablets
17474	Felodipine 5mg modified-release / Ramipril 5mg tablets
17492	Zemtard 300 XL capsules (Galen Ltd)
17545	Micardis 80mg tablets (Boehringer Ingelheim Ltd)
17557	Felotens XL 5mg tablets (Thornton & Ross Ltd)
17561	Bendroflumethiazide 2.5mg / Potassium chloride 573mg (potassium 7.7mmol) modified-release tablets
17566	Felotens XL 10mg tablets (Thornton & Ross Ltd)
17586	Slozem 300mg capsules (Merck Serono Ltd)
17599	Verapress MR 240mg tablets (Sandoz Ltd)
17615	Cardicor 5mg tablets (Merck Serono Ltd)
17624	Captopril 5mg/5ml oral suspension
17633	Captopril 3mg/5ml oral solution
17640	Amlostin 5mg tablets (Discovery Pharmaceuticals Ltd)
17655	Carace 10 Tablet (Bristol-Myers Squibb Pharmaceuticals Ltd)
17666	Viazem XL 180mg capsules (Thornton & Ross Ltd)
17679	Sotalol 10mg/ml injection
17686	Micardis 20mg tablets (Boehringer Ingelheim Ltd)
17689	MicardisPlus 80mg/12.5mg tablets (Boehringer Ingelheim Ltd)
17720	Saluric 500mg Tablet (Merck Sharp & Dohme Ltd)
17783	Spiroprop Tablet (Pharmacia Ltd)
17902	Spirolone 100mg Tablet (Berk Pharmaceuticals Ltd)
17950	Spirolone 50mg Tablet (Berk Pharmaceuticals Ltd)
18038	Nisoldipine 20mg modified-release tablets
18096	Torasemide 10mg tablets
18185	Cardicor 7.5mg tablets (Merck Serono Ltd)
18200	Olmesartan medoxomil 20mg / Hydrochlorothiazide 12.5mg tablets
18202	MicardisPlus 40mg/12.5mg tablets (Boehringer Ingelheim Ltd)
18219	Imidapril 20mg tablets
18223	Trandolapril with verapamil 2mg + 180mg Modified-release capsule

18247	Esbatal 10mg Tablet (Wellcome Medical Division)
18252	Metalpha 250mg Tablet (Ashbourne Pharmaceuticals Ltd)
18263	Acezide 25mg/50mg tablets (Bristol-Myers Squibb Pharmaceuticals Ltd)
18267	Enduron 5mg Tablet (Abbott Laboratories Ltd)
18269	Acepril 12.5mg tablets (Bristol-Myers Squibb Pharmaceuticals Ltd)
18287	Co-Betaloc SA tablets (Pfizer Ltd)
18325	Acepril 50mg tablets (Bristol-Myers Squibb Pharmaceuticals Ltd)
18332	Aridil 20mg+2.5mg Tablet (C P Pharmaceuticals Ltd)
18361	Amilmaxco 5mg/50mg tablets (Ashbourne Pharmaceuticals Ltd)
18379	Dilcardia SR 90mg capsules (Generics (UK) Ltd)
18403	Diltiazem HCl 180mg Modified-release capsule (Hillcross Pharmaceuticals Ltd)
18404	Diltiazem 60mg modified-release capsules (A A H Pharmaceuticals Ltd)
18414	Eucardic 12.5mg tablets (Roche Products Ltd)
18497	Amiloride 10mg / furosemide 80mg tablets
18606	Diltiazem and hydrochlorothiazide 150mg+12.5mg modified-release capsules
18650	Edecrin 50mg Tablet (Merck Sharp & Dohme Ltd)
18716	Dryptal 10mg/ml Injection (Berk Pharmaceuticals Ltd)
18726	Triamaxco 50mg/25mg tablets (Ashbourne Pharmaceuticals Ltd)
18733	Co-amilozide 5mg with 50mg/ml oral solution
18743	Tenben 25mg/1.25mg capsules (Galen Ltd)
18830	Disogram SR 90mg capsules (Ranbaxy (UK) Ltd)
18834	Disogram SR 60mg capsules (Ranbaxy (UK) Ltd)
18852	Disogram SR 120mg capsules (Ranbaxy (UK) Ltd)
18861	Hydralazine 10mg/5ml oral suspension
18874	Disogram SR 180mg capsules (Ranbaxy (UK) Ltd)
18903	Olmesartan medoxomil 20mg / Hydrochlorothiazide 25mg tablets
18910	Olmetec 20mg tablets (Daiichi Sankyo UK Ltd)
18950	Totamol 25mg Tablet (C P Pharmaceuticals Ltd)
18973	Centyl 2.5mg Tablet (Edwin Burgess Ltd)
18975	Calcicard 60mg Tablet (3M Health Care Ltd)
18983	Brinaldix k Effervescent tablet (Berk Pharmaceuticals Ltd)
19013	Clinium 120mg Tablet (LEO Pharma)
19055	Chlortalidone 12.5mg with Atenolol 50mg tablets
19056	Furosemide 50mg/5ml sugar free Oral solution (Rosemont Pharmaceuticals Ltd)
19129	Syscor MR 10 tablets (Forest Laboratories UK Ltd)
19142	Bendroflumethiazide 2.5mg with Timolol maleate 10mg tablets
19170	Tensipine MR 10 tablets (Thornton & Ross Ltd)
19172	Atenolol 25mg tablets (IVAX Pharmaceuticals UK Ltd)
19175	Verapamil 40mg tablets (IVAX Pharmaceuticals UK Ltd)
19178	Bisoprolol 10mg tablets (Ranbaxy (UK) Ltd)
19182	Atenolol 50mg tablets (IVAX Pharmaceuticals UK Ltd)
19191	Atenolol 100mg tablets (Teva UK Ltd)
19192	Furosemide 40mg Tablet (M & A Pharmachem Ltd)
19193	Doxazosin 2mg tablets (Teva UK Ltd)
19194	Furosemide 20mg tablets (Teva UK Ltd)

19195	Spironolactone 50mg Tablet (Wyeth Pharmaceuticals)
19198	Lisinopril 20mg tablets (Teva UK Ltd)
19200	Bisoprolol 5mg tablets (IVAX Pharmaceuticals UK Ltd)
19202	Carvedilol 6.25mg tablets (Teva UK Ltd)
19204	Lisinopril 5mg tablets (Teva UK Ltd)
19208	Enalapril 10mg tablets (Actavis UK Ltd)
19216	Doxazosin 4mg tablets (IVAX Pharmaceuticals UK Ltd)
19223	Lisinopril 10mg tablets (Teva UK Ltd)
19258	Furosemide 50mg/5ml solution for injection ampoules
19300	Bumetanide 2mg/4ml solution for injection ampoules
19325	Cordilox 2.5mg/ml Injection (IVAX Pharmaceuticals UK Ltd)
19352	Xuret 0.5mg Tablet (Galen Ltd)
19426	Disogram SR 240mg capsules (Ranbaxy (UK) Ltd)
19437	Eucardic 25mg tablets (Roche Products Ltd)
19440	Disogram SR 300mg capsules (Ranbaxy (UK) Ltd)
19457	Ranvera MR 240mg tablets (Ranbaxy (UK) Ltd)
19459	Verapamil 240mg modified-release tablets (A A H Pharmaceuticals Ltd)
19690	Verapamil 180mg modified-release / Trandolapril 2mg capsules
19823	Alphavase 5 tablets (Ashbourne Pharmaceuticals Ltd)
19853	Cardicor 3.75mg tablets (Merck Serono Ltd)
19858	Cardicor 10mg tablets (Merck Serono Ltd)
19890	Hydrochlorothiazide with amiloride 25mgwith2.5mg Tablet
19892	Serpasil -esidrex Tablet (Novartis Pharmaceuticals UK Ltd)
19998	Trandate 100mg/20ml solution for injection ampoules (Focus Pharmaceuticals Ltd)
20012	Visken 15mg Tablet (Sovereign Medical Ltd)
20057	Methyclothiazide 5mg Tablet
20066	Amil-Co 5mg/50mg tablets (IVAX Pharmaceuticals UK Ltd)
20082	Lopresor SR 200mg tablets (Recordati Pharmaceuticals Ltd)
20093	Metoprolol 200mg modified-release / Hydrochlorothiazide 25mg tablets
20117	Olmetec 40mg tablets (Daiichi Sankyo UK Ltd)
20169	Practolol 2mg/ml injection
20188	Enalapril 2.5mg wafer
20257	Cardilate MR 20mg tablets (IVAX Pharmaceuticals UK Ltd)
20311	Nifedipress mr 20mg Modified-release tablet (Generics (UK) Ltd)
20369	Doxazosin 1mg/5ml oral suspension
20426	Centyl k 2.5mg+7.7mmol Tablet (Edwin Burgess Ltd)
20431	Centyl K modified-release tablets (LEO Pharma)
20459	Felendil xl 10mg Modified-release tablet (Ratiopharm UK Ltd)
20468	Half Beta-Prograne 80mg modified-release capsules (Tillomed Laboratories Ltd)
20502	Atenix 100 tablets (Ashbourne Pharmaceuticals Ltd)
20538	Frumax 40mg Tablet (Ashbourne Pharmaceuticals Ltd)
20579	Tarka modified-release capsules (Abbott Laboratories Ltd)
20591	Nifedipress MR 20 tablets (Teva UK Ltd)
20642	Bi-carzem sr 60mg Modified-release capsule (Tillomed Laboratories Ltd)
20656	Serpasil 250microgram Tablet (Novartis Pharmaceuticals UK Ltd)

20690	Reserpine 250micrograms tablet
20728	Atenamin 25mg Tablet (OPD Pharm)
20849	Tensopril 12.5mg tablets (Teva UK Ltd)
20878	Angiopine 10 capsules (Ashbourne Pharmaceuticals Ltd)
20890	Zemtard 240 XL capsules (Galen Ltd)
20975	Lisinopril 7.5mg/5ml oral suspension
21025	Prestim forte Tablet (LEO Pharma)
21053	Vascace 500microgram tablets (Roche Products Ltd)
21133	Atenamin 50mg Tablet (OPD Pharm)
21145	Dilcardia SR 60mg capsules (Generics (UK) Ltd)
21162	Felodipine 2.5mg modified-release / Ramipril 2.5mg tablets
21182	Hydrochlorothiazide with timolol and amiloride 25mg with 10mg with 2.5mg Tablet
21216	Hypolar Retard 10mg tablets (Sandoz Ltd)
21231	Caralpha 20mg/12.5mg tablets (Actavis UK Ltd)
21245	Nifedipress mr 10mg Modified-release tablet (Actavis UK Ltd)
21346	Hydromet Tablet (MSD Thomas Morson Pharmaceuticals)
21423	Cozaar-Comp 100mg/25mg tablets (Merck Sharp & Dohme Ltd)
21502	Serpasil 100microgram Tablet (Novartis Pharmaceuticals UK Ltd)
21763	Diltiazem 60mg modified-release tablets (A A H Pharmaceuticals Ltd)
21773	Diltiazem HCl 60mg Tablet (Generics (UK) Ltd)
21778	Diltiazem 60mg modified-release tablets (Teva UK Ltd)
21795	Retalzem 60 modified-release tablets (Kent Pharmaceuticals Ltd)
21803	Berkozide 2.5mg Tablet (Berk Pharmaceuticals Ltd)
21838	Propanix 80mg Tablet (Ashbourne Pharmaceuticals Ltd)
21839	Berkolol 80mg Tablet (Berk Pharmaceuticals Ltd)
21849	Dryptal 40mg Tablet (Berk Pharmaceuticals Ltd)
21866	Berkolol 40mg Tablet (Berk Pharmaceuticals Ltd)
21867	Berkozide 5mg Tablet (Berk Pharmaceuticals Ltd)
21872	Angiopine 5mg Capsule (Ashbourne Pharmaceuticals Ltd)
21873	Atenix Co 50 tablets (Ashbourne Pharmaceuticals Ltd)
21885	Oxyprenix SR 160mg tablets
21886	Nifedipress MR 20 tablets (Actavis UK Ltd)
21905	Bipranix 10mg tablets (Ashbourne Pharmaceuticals Ltd)
21911	Spirospare 25mg Tablet (Ashbourne Pharmaceuticals Ltd)
21918	Optil 60mg modified-release tablets (Opus Pharmaceuticals Ltd)
21938	Froop Co 5mg/40mg tablets (Ashbourne Pharmaceuticals Ltd)
21943	Kaplon 12.5mg tablets (Teva UK Ltd)
21966	Bipranix 5mg tablets (Ashbourne Pharmaceuticals Ltd)
22019	Calanif 10mg Capsule (Berk Pharmaceuticals Ltd)
22142	Calcilat 10mg Capsule (Eastern Pharmaceuticals Ltd)
22208	Half propanix la 80mg Modified-release capsule (Ashbourne Pharmaceuticals Ltd)
22217	Nimodrel 10mg modified-release tablet (Opus Pharmaceuticals Ltd)
22241	Mibefradil 100mg Tablet
22439	Ednyt 20mg Tablet (Dominion Pharma)
22454	Demser 250mg Capsule (Merck Sharp & Dohme Ltd)

22619	Britiazim 60mg Modified-release tablet (Thames Laboratories Ltd)
22658	Torem 10mg tablets (Meda Pharmaceuticals Ltd)
22696	Slofedipine 20mg tablets (Sterwin Medicines)
22708	Enalapril 5mg wafer
22793	Labrocol 200mg Tablet (Lagap)
22826	Securon 160mg Tablet (Abbott Laboratories Ltd)
22839	Clopamide with Potassium effervescent tablets
22853	Reserpine 100micrograms tablet
22912	Bendroflumethiazide 2.5mg with Propanolol 80mg capsules
22923	Hydrochlorothiazide with amiloride 50mg with 5mg Tablet
23010	Rogitine 10mg/1ml solution for injection ampoules (Alliance Pharmaceuticals Ltd)
23091	Spirospare 100 tablets (Ashbourne Pharmaceuticals Ltd)
23131	Bendroflumethiazide 5mg with Propanolol 160mg modified-release capsules
23134	Nadolol 40mg / Bendroflumethiazide 5mg tablets
23233	Bi-carzem sr 90mg Modified-release capsule (Tillomed Laboratories Ltd)
23252	Pralenal 10 tablets (Opus Pharmaceuticals Ltd)
23326	Betadur cr 160mg Modified-release capsule (Monmouth Pharmaceuticals Ltd)
23345	Hypercal 2mg Tablet (Carlton Laboratories)
23380	Catapres 300microgram tablets (Boehringer Ingelheim Ltd)
23427	Bendroflumethiazide 5mg tablets (A A H Pharmaceuticals Ltd)
23456	Hydrochlorothiazide with valsartan 25mg with 160mg Tablet
23459	Hypovase benign prostatic hyperplasia 2mg Tablet (Pfizer Ltd)
23478	Tensopril 50mg tablets (Teva UK Ltd)
23505	Adizem xl plus 150mg+12.5mg Modified-release capsule (Napp Pharmaceuticals Ltd)
23587	Sloprolol 160mg Capsule (C P Pharmaceuticals Ltd)
23642	Vascace 0.25mg Tablet (Roche Products Ltd)
23733	Optil sr 90mg Modified-release capsule (Opus Pharmaceuticals Ltd)
23736	Hypolar XL 30 tablets (Sandoz Ltd)
23761	Methyldopa 250mg/5ml oral suspension
23805	Nisoldipine 10mg modified-release tablets
23823	Nisoldipine 30mg modified-release tablets
23872	Berkatens 40mg Tablet (Berk Pharmaceuticals Ltd)
24008	Vasetic Tablet (Shire Pharmaceuticals Ltd)
24041	Enalapril 20mg wafer
24083	Bisoprolol 5mg tablets (Teva UK Ltd)
24094	Trasicor 40mg tablets (Amdipharm Plc)
24189	Neo-bendromax 2.5mg Tablet (Ashbourne Pharmaceuticals Ltd)
24190	Neo-bendromax 5mg Tablet (Ashbourne Pharmaceuticals Ltd)
24191	Antipressan 50mg tablets (Teva UK Ltd)
24195	Antipressan 100mg tablets (Teva UK Ltd)
24196	Dopamet 250mg Tablet (Berk Pharmaceuticals Ltd)
24218	Berkolol 160mg Tablet (Berk Pharmaceuticals Ltd)
24228	Nimodrel 20mg modified-release tablet (Opus Pharmaceuticals Ltd)
24268	Hydrochlorothiazide with valsartan 12.5mg with 80mg Tablet
24280	Totaretic 100mg+25mg Tablet (C P Pharmaceuticals Ltd)

24359	Diovan 40mg tablets (Novartis Pharmaceuticals UK Ltd)
24365	Cardioplen XL 5mg tablets (Chiesi Ltd)
24366	Cardioplen XL 10mg tablets (Chiesi Ltd)
24369	Petyme 400microgram MR capsules (Teva UK Ltd)
24461	Betaloc I.V. 5mg/5ml solution for injection ampoules (AstraZeneca UK Ltd)
24482	Captomex 50mg tablets (Actavis UK Ltd)
24484	Hydrochlorothiazide with valsartan 12.5mg with 160mg Tablet
24632	Hydrochlorothiazide with losartan 25mg with 100mg Tablet
24635	Sotacor 10mg/ml Injection (Bristol-Myers Squibb Pharmaceuticals Ltd)
24832	Lasipressin Tablet (Hoechst UK Ltd)
24835	Min-i-jet furosemide 10mg/ml Injection (Celltech Pharma Europe Ltd)
24893	Amilospare Tablet (Ashbourne Pharmaceuticals Ltd)
25047	Hypovase benign prostatic hyperplasia 500microgram Tablet (Pfizer Ltd)
25059	Berkatens 80mg Tablet (Berk Pharmaceuticals Ltd)
25132	Nifopress MR 20mg tablets (Teva UK Ltd)
25275	Metalpha 500mg Tablet (Ashbourne Pharmaceuticals Ltd)
25289	Dopamet 500mg Tablet (Berk Pharmaceuticals Ltd)
25334	Furosemide 500mg tablets (A A H Pharmaceuticals Ltd)
25359	Rapranol SR 160mg capsules (Ranbaxy (UK) Ltd)
25363	Prestim tablets (Meda Pharmaceuticals Ltd)
25367	Rapranol SR 80mg capsules (Ranbaxy (UK) Ltd)
25382	Co-Diovan 160mg/25mg tablets (Novartis Pharmaceuticals UK Ltd)
25393	Decaserpyl 10mg Tablet (Roussel Laboratories Ltd)
25462	Clopamide 5mg with Pindolol 10mg tablets
25487	Cascor 2mg tablets (Ranbaxy (UK) Ltd)
25494	Diatensec 50mg Tablet (Pharmacia Ltd)
25500	Hypertane 50 Tablet (Schwarz Pharma Ltd)
25505	Spiro-co 50mg+50mg Tablet (IVAX Pharmaceuticals UK Ltd)
25551	Cascor 4mg tablets (Ranbaxy (UK) Ltd)
25572	Felogen XL 5mg tablets (Generics (UK) Ltd)
25644	Apsolox 80mg Tablet (Approved Prescription Services Ltd)
25645	Seominal Tablet (Sterling-Winthrop)
25646	Nivaten retard 20mg Modified-release tablet (Actavis UK Ltd)
25717	Furosemide 40mg tablets (Generics (UK) Ltd)
25730	Timolol maleate with amiloride and hydrochlorothiazide Tablet
25777	Dilcardia SR 120mg capsules (Generics (UK) Ltd)
25919	Nifedipine 20mg modified-release tablets (A A H Pharmaceuticals Ltd)
25965	Co-amilofruse 2.5mg/20mg tablets (Wockhardt UK Ltd)
25998	Captomex 12.5mg tablets (Actavis UK Ltd)
26211	Antipressan 25mg tablets (Teva UK Ltd)
26217	Berkamil 5mg Tablet (Berk Pharmaceuticals Ltd)
26219	Zida-co 5mg+50mg Tablet (Opus Pharmaceuticals Ltd)
26220	Delvas Tablet (Berk Pharmaceuticals Ltd)
26228	Propanix LA 160mg Modified-release capsule (Ashbourne Pharmaceuticals Ltd)
26229	Beta-Prograne 160mg modified-release capsules (Tillomed Laboratories Ltd)

26237	Alphavase 500microgram Tablet (Ashbourne Pharmaceuticals Ltd)
26238	Alphavase 1 tablets (Ashbourne Pharmaceuticals Ltd)
26248	Tenchlor 100mg/25mg tablets (Teva UK Ltd)
26252	Berkatens 160mg Tablet (Berk Pharmaceuticals Ltd)
26255	Lopranol la 160mg Capsule (Opus Pharmaceuticals Ltd)
26256	Opumide 2.5mg Tablet (Opus Pharmaceuticals Ltd)
26265	Calanif 5mg Capsule (Berk Pharmaceuticals Ltd)
26267	Optil sr 120mg Modified-release capsule (Opus Pharmaceuticals Ltd)
26269	Optil sr 180mg Modified-release capsule (Opus Pharmaceuticals Ltd)
26270	Optil xl 300mg Modified-release capsule (Opus Pharmaceuticals Ltd)
26275	Nindaxa 2.5 tablets (Ashbourne Pharmaceuticals Ltd)
26292	Diuresal 40mg Tablet (Lagap)
26309	Optil xl 240mg Modified-release capsule (Opus Pharmaceuticals Ltd)
26337	Cabren 10mg modified-release tablets (Teva UK Ltd)
26460	Dilcardia xl 240mg Modified-release capsule (Generics (UK) Ltd)
26463	Zemret xl 240mg Capsule (Neo Laboratories Ltd)
26529	Furosemide with penbutolol Tablet
26674	Verapamil 5mg/2ml solution for injection ampoules
26693	Hypovase benign prostatic hyperplasia bd BD Starter pack (Pfizer Ltd)
26741	Totaretic 50mg+12.5mg Tablet (C P Pharmaceuticals Ltd)
26759	Zildil SR 60mg capsules (Chanelle Medical UK Ltd)
26774	Nifedipine 10mg/5ml Oral suspension
26895	Syprol 10mg/5ml oral solution (Rosemont Pharmaceuticals Ltd)
26919	Methyldopa 50mg/ml Injection
26995	Kaplon 25mg tablets (Teva UK Ltd)
27135	Diltiazem sr 90mg Capsule (Hillcross Pharmaceuticals Ltd)
27136	Diltiazem 90mg modified-release tablets (A A H Pharmaceuticals Ltd)
27137	Sildenafil 20mg tablets
27256	Bendroflumethiazide 2.5mg tablets (Wockhardt UK Ltd)
27295	Securon IV 5mg/2ml solution for injection ampoules (Abbott Laboratories Ltd)
27357	Oxprenolol 40mg Tablet (Actavis UK Ltd)
27401	Kenzem SR 90mg capsules (Kent Pharmaceuticals Ltd)
27403	Omnic MR 400microgram capsules (Astellas Pharma Ltd)
27447	Furosemide 40mg tablets (Wockhardt UK Ltd)
27520	Olmetec Plus 20mg/25mg tablets (Daiichi Sankyo UK Ltd)
27685	Diltiazem HCl 300mg Capsule (PLIVA Pharma Ltd)
27689	Bendroflumethiazide 2.5mg tablets (IVAX Pharmaceuticals UK Ltd)
27690	Furosemide 40mg tablets (A A H Pharmaceuticals Ltd)
27696	Furosemide 40mg tablets (Kent Pharmaceuticals Ltd)
27700	Propranolol 40mg tablets (Actavis UK Ltd)
27719	Metoros ls 95mg Tablet (Geigy Pharmaceuticals)
27727	Sotalol 2mg/ml injection
27871	Innovace melt 10mg Wafer (Merck Sharp & Dohme Ltd)
27926	Furosemide 20mg tablets (Generics (UK) Ltd)
27946	Nadolol 80mg / Bendroflumethiazide 5mg tablets

27957	Natramid 2.5mg Tablet (Trinity Pharmaceuticals Ltd)
27964	Apsolol 40mg Tablet (Approved Prescription Services Ltd)
28048	Angilol 10mg Tablet (DDSA Pharmaceuticals Ltd)
28127	Enalapril 2.5mg tablets (Teva UK Ltd)
28128	Propranolol 80mg Modified-release capsule (Actavis UK Ltd)
28129	Co-amilofruse 5mg/40mg tablets (Teva UK Ltd)
28157	Kalspare ls Tablet (Dominion Pharma)
28177	Hydrochlorothiazide with atenolol and amiloride Capsule
28438	Triapin 2.5mg/2.5mg modified-release tablets (Sanofi)
28441	Pamsvax XL 400microgram capsules (Actavis UK Ltd)
28486	Captopril 6.25mg/5ml oral suspension
28586	Lopace 5mg capsules (Discovery Pharmaceuticals Ltd)
28676	Bethanidine sulphate 50mg tablets
28688	Nifedipine 10mg modified-release tablets (A A H Pharmaceuticals Ltd)
28700	Cartrol 10mg Tablet (Novartis Consumer Health UK Ltd)
28721	Neofel XL 5mg tablets (Kent Pharmaceuticals Ltd)
28724	Perdix 7.5mg tablets (UCB Pharma Ltd)
28725	Perdix 15mg tablets (UCB Pharma Ltd)
28738	Methyldopa with hydrochlorothiazide Tablet
28788	Half propatard la 80mg Modified-release capsule (Galen Ltd)
28820	Captomex 25mg tablets (Actavis UK Ltd)
28843	Verapamil hc 80mg Tablet (Celltech Pharma Europe Ltd)
28844	Berkatens 120mg Tablet (Berk Pharmaceuticals Ltd)
28902	Odrik 2mg capsules (Aventis Pharma)
28949	Bi-carzem sr 120mg Modified-release capsule (Tillomed Laboratories Ltd)
28996	Bedranol SR 160mg capsules (Sandoz Ltd)
29044	Neofel XL 10mg tablets (Kent Pharmaceuticals Ltd)
29130	Gopten 4mg capsules (Abbott Laboratories Ltd)
29145	Felendil xl 2.5mg Modified-release tablet (Ratiopharm UK Ltd)
29180	Trasicor 80mg tablets (Amdipharm Plc)
29187	Methoserpidine 10mg Tablet
29230	Slow-pren 160mg Tablet (IVAX Pharmaceuticals UK Ltd)
29368	Atenolol 25mg tablets (Teva UK Ltd)
29397	Spiretic 100mg Tablet (DDSA Pharmaceuticals Ltd)
29398	Atenamin 100mg Tablet (OPD Pharm)
29427	Hydrochlorothiazide with metoprolol tartrate 12.5mg with 100mg tablet
29443	Bendogen 10mg Tablet (Lagap)
29529	Hydroflumethiazide with spironolactone 25mg+25mg Tablet
29530	Innovace melt 2.5mg Wafer (Merck Sharp & Dohme Ltd)
29560	Bosentan 62.5mg tablets
29561	Bosentan 125mg tablets
29570	Dopamet 125mg Tablet (Berk Pharmaceuticals Ltd)
29610	Betim 10mg tablets (Meda Pharmaceuticals Ltd)
29627	Lopace 2.5mg capsules (Discovery Pharmaceuticals Ltd)
29634	Olmetec Plus 20mg/12.5mg tablets (Daiichi Sankyo UK Ltd)

29637	Verapress MR 240mg tablets (Teva UK Ltd)
29676	Calazem 60mg Modified-release tablet (Berk Pharmaceuticals Ltd)
29694	Inspra 50mg tablets (Pfizer Ltd)
29696	Methoserpidine with benzthiazide Tablet
29762	Mepranix 50mg Tablet (Ashbourne Pharmaceuticals Ltd)
29763	Propanix 160mg Tablet (Ashbourne Pharmaceuticals Ltd)
29780	Furosemide 20mg Tablet (C P Pharmaceuticals Ltd)
29827	Carteolol HCl 10mg tablets
29991	Centyl 5mg Tablet (Edwin Burgess Ltd)
29998	Metoros 190mg Tablet (Novartis Pharmaceuticals UK Ltd)
30039	Tensopril 25mg tablets (Teva UK Ltd)
30127	Ismelin 10mg/ml Injection (Sovereign Medical Ltd)
30129	Abicol Tablet (Knoll Ltd)
30197	Diltiazem 120mg modified-release capsules
30199	Nifedipine 30mg modified-release tablets
30242	Diltiazem 180mg modified-release capsules
30272	Benthiazide with Triamterene capsules
30293	Catapres 150micrograms/1ml solution for injection ampoules (Boehringer Ingelheim Ltd)
30400	Mepranix 100mg Tablet (Ashbourne Pharmaceuticals Ltd)
30462	Ethimil MR 240mg tablets (Genus Pharmaceuticals Ltd)
30473	Coroday MR 20mg tablets (Generics (UK) Ltd)
30519	Amiloride with timolol with hydrochlorothiazide tablets
30557	Felogen XL 10mg tablets (Generics (UK) Ltd)
30625	Furosemide 20mg tablets (A A H Pharmaceuticals Ltd)
30636	Vasaten 50mg Tablet (Shire Pharmaceuticals Ltd)
30691	Hypercal b Tablet (Carlton Laboratories)
30758	Lidoflazine 120mg Tablet
30770	Labetalol 200mg tablets (A A H Pharmaceuticals Ltd)
30773	Co-amilofruse 5mg+40mg Tablet (Berk Pharmaceuticals Ltd)
30875	Furosemide 250mg/25ml solution for injection ampoules
30913	Betinex 1mg Tablet (Berk Pharmaceuticals Ltd)
30915	Cabren 2.5mg modified-release tablets (Teva UK Ltd)
30921	Lisinopril 2.5mg tablets (Teva UK Ltd)
30967	Revatio 20mg tablets (Pfizer Ltd)
30991	Cabren 5mg modified-release tablets (Teva UK Ltd)
31072	Amias 32mg tablets (Takeda UK Ltd)
31080	Guanethidine 10mg/1ml solution for injection ampoules
31109	Prosurin XL 400microgram capsules (Generics (UK) Ltd)
31131	Spiro-co 25mg+25mg Tablet (IVAX Pharmaceuticals UK Ltd)
31150	Co-amilozide 5mg/50mg tablets (IVAX Pharmaceuticals UK Ltd)
31214	Propranolol 80mg tablets (Generics (UK) Ltd)
31219	Spironolactone 100mg tablets (A A H Pharmaceuticals Ltd)
31220	Hydralazine 25mg tablets (A A H Pharmaceuticals Ltd)
31307	Odrik 500microgram capsules (Aventis Pharma)
31336	Syscor MR 30 tablets (Forest Laboratories UK Ltd)

31337	Syscor MR 20 tablets (Forest Laboratories UK Ltd)
31375	Amilamont 5mg/5ml oral solution sugar free (Rosemont Pharmaceuticals Ltd)
31470	Tenchlor 50mg/12.5mg tablets (Teva UK Ltd)
31489	Bi-carzem xl 240mg Capsule (Tillomed Laboratories Ltd)
31490	Zolvera 40mg/5ml oral solution (Rosemont Pharmaceuticals Ltd)
31529	Spironolactone 25mg tablets (Teva UK Ltd)
31536	Atenolol 25mg tablets (Kent Pharmaceuticals Ltd)
31548	Furosemide 20mg tablets (Actavis UK Ltd)
31587	Innovace melt 20mg Wafer (Merck Sharp & Dohme Ltd)
31670	Bendroflumethiazide 2.5mg tablets (Teva UK Ltd)
31676	Diltiazem HCl 120mg Modified-release tablet (Actavis UK Ltd)
31708	Co-tenidone 50mg/12.5mg tablets (Actavis UK Ltd)
31711	Verapamil 80mg tablets (A A H Pharmaceuticals Ltd)
31716	Enalapril 20mg tablets (Actavis UK Ltd)
31737	Zildil SR 120mg capsules (Chanelle Medical UK Ltd)
31761	Amlostin 10mg tablets (Discovery Pharmaceuticals Ltd)
31773	Co-amilofruse 5mg/40mg tablets (Wockhardt UK Ltd)
31776	Propranolol 40mg tablets (Generics (UK) Ltd)
31810	Odrik 1mg capsules (Aventis Pharma)
31820	Bendroflumethiazide 5mg tablets (Wockhardt UK Ltd)
31833	Angilol 80mg Tablet (DDSA Pharmaceuticals Ltd)
31932	Bumetanide 1mg tablets (C P Pharmaceuticals Ltd)
31934	Atenolol 100mg tablets (IVAX Pharmaceuticals UK Ltd)
32002	Etacrynic 50mg/vial injection
32048	Kaplon 50mg tablets (Teva UK Ltd)
32089	Diltiazem HCl 120mg Modified-release capsule (Hillcross Pharmaceuticals Ltd)
32091	Bumetanide 1mg tablets (A A H Pharmaceuticals Ltd)
32094	Co-tenidone 50mg/12.5mg tablets (A A H Pharmaceuticals Ltd)
32114	Bisoprolol 5mg tablets (Generics (UK) Ltd)
32162	Propranolol 80mg Modified-release capsule (Lagap)
32166	Capto-co 25mg+50mg Tablet (IVAX Pharmaceuticals UK Ltd)
32241	Enalapril 10mg tablets (A A H Pharmaceuticals Ltd)
32262	Diltiazem HCl 60mg Tablet (C P Pharmaceuticals Ltd)
32267	Diazoxide 300mg/20ml solution for injection ampoules
32277	Furosemide 80mg/8ml solution for injection pre-filled syringes
32514	Ecopace 25mg tablets (AMCo)
32552	Congescor 2.5mg tablets (Tillomed Laboratories Ltd)
32560	Tanatril 20mg tablets (Chiesi Ltd)
32590	Verapamil 40mg tablets (Generics (UK) Ltd)
32595	Amlodipine 5mg tablets (A A H Pharmaceuticals Ltd)
32597	Lisinopril 10mg tablets (Sandoz Ltd)
32630	Vivacor 10mg tablets (Lexon (UK) Ltd)
32658	Dilcardia xl 120mg Modified-release capsule (Generics (UK) Ltd)
32787	Visken 15mg tablets (AMCo)
32836	Metoprolol 50mg tablets (Generics (UK) Ltd)

32837	Spironolactone 50mg tablets (Teva UK Ltd)
32857	Ramipril 1.25mg capsules (Teva UK Ltd)
32870	Diltiazem 60mg modified-release tablets (Sterwin Medicines)
32896	Furosemide 40mg tablets (Ranbaxy (UK) Ltd)
32913	Methyldopa 250mg tablets (Actavis UK Ltd)
32917	Amlodipine 5mg tablets (IVAX Pharmaceuticals UK Ltd)
32918	Furosemide 20mg tablets (Sandoz Ltd)
32922	Felodipine 10mg Modified-release tablet (Sandoz Ltd)
32934	Lopace 10mg capsules (Discovery Pharmaceuticals Ltd)
33025	Nimodrel XL 30mg tablets (Zurich Pharmaceuticals)
33057	Ednyt 5mg Tablet (Dominion Pharma)
33078	Enalapril 20mg tablets (A A H Pharmaceuticals Ltd)
33079	Atenolol 100mg tablets (Generics (UK) Ltd)
33083	Indapamide 2.5mg tablets (Teva UK Ltd)
33085	Atenolol 100mg tablets (A A H Pharmaceuticals Ltd)
33091	Felodipine 10mg modified-release tablets (A A H Pharmaceuticals Ltd)
33092	Atenolol 50mg tablets (A A H Pharmaceuticals Ltd)
33093	Clonidine 25microgram tablets (Sandoz Ltd)
33094	Doxazosin 2mg tablets (Generics (UK) Ltd)
33095	Perindopril erbumine 4mg tablets (A A H Pharmaceuticals Ltd)
33184	Atenolol 100mg tablets (Wockhardt UK Ltd)
33322	Moxonidine 200microgram tablets (Sandoz Ltd)
33336	Captopril 5mg/5ml Oral suspension (Eldon Laboratories)
33353	Lisinopril 20mg / Hydrochlorothiazide 12.5mg tablets (Teva UK Ltd)
33374	Carvedilol 12.5mg tablets (Genus Pharmaceuticals Ltd)
33376	Probeta LA 160mg Capsule (Trinity Pharmaceuticals Ltd)
33415	Bendroflumethiazide 2.5mg tablets (Generics (UK) Ltd)
33471	Verapamil 40mg tablets (Actavis UK Ltd)
33527	Co-amilofruse 5mg/40mg tablets (Generics (UK) Ltd)
33569	Oxprenolol sr 160mg Modified-release tablet (Hillcross Pharmaceuticals Ltd)
33602	Slo-Pro 160mg capsules (Generics (UK) Ltd)
33644	Propranolol 80mg tablets (A A H Pharmaceuticals Ltd)
33646	Captopril 12.5mg Tablet (Generics (UK) Ltd)
33650	Atenolol 50mg tablets (Generics (UK) Ltd)
33651	Bendroflumethiazide 2.5mg tablets (A A H Pharmaceuticals Ltd)
33657	Atenolol 25mg tablets (A A H Pharmaceuticals Ltd)
33658	Co-amilofruse 5mg/40mg tablets (A A H Pharmaceuticals Ltd)
33659	Hydrochlorothiazide with metoprolol tartrate 25mg with 200mg Modified-release tablet
33724	Diuril 250mg/5ml oral suspension (Imported (United States))
33788	Metirosine 250mg Capsule
33811	Ramipril 2.5mg capsules (Ranbaxy (UK) Ltd)
33836	Apsolol 160mg Tablet (Approved Prescription Services Ltd)
33837	Amiloride 5mg tablets (A A H Pharmaceuticals Ltd)
33839	Bisoprolol 10mg tablets (Actavis UK Ltd)
33850	Atenolol 50mg tablets (Actavis UK Ltd)

33894	Ramipril 10mg capsules (Teva UK Ltd)
33909	Congescor 1.25mg tablets (Tillomed Laboratories Ltd)
33932	Parmid XL 5mg tablets (Sandoz Ltd)
33977	Lisinopril 10mg tablets (Generics (UK) Ltd)
34006	Furosemide 40mg tablets (Actavis UK Ltd)
34012	Co-tenidone 100mg/25mg tablets (IVAX Pharmaceuticals UK Ltd)
34034	Co-tenidone 50mg/12.5mg tablets (IVAX Pharmaceuticals UK Ltd)
34059	Bendroflumethiazide 2.5mg tablets (Actavis UK Ltd)
34080	Stronazon 400microgram MR capsules (Actavis UK Ltd)
34092	Metoprolol 100mg tablets (Teva UK Ltd)
34093	Amlodipine 10mg tablets (A A H Pharmaceuticals Ltd)
34094	Metoprolol 50mg tablets (A A H Pharmaceuticals Ltd)
34101	Nifedipine mr 20mg Modified-release tablet (IVAX Pharmaceuticals UK Ltd)
34115	Nifedipine 60mg Modified-release tablet
34124	Bendroflumethiazide 5mg tablets (Actavis UK Ltd)
34125	Metoprolol 100mg tablets (A A H Pharmaceuticals Ltd)
34146	Nifedipine mr 10mg Modified-release tablet (IVAX Pharmaceuticals UK Ltd)
34171	Labetalol 100mg Tablet (C P Pharmaceuticals Ltd)
34177	Labetalol 100mg tablets (A A H Pharmaceuticals Ltd)
34185	Propranolol LA 80mg Modified-release capsule (Approved Prescription Services Ltd)
34187	Nifedipine 10mg Modified-release tablet (Generics (UK) Ltd)
34188	Labetalol 200mg Tablet (Celltech Pharma Europe Ltd)
34208	Propranolol SR 160mg Modified-release capsule (C P Pharmaceuticals Ltd)
34214	Propranolol 160mg tablets (Actavis UK Ltd)
34247	Nifedipine 10mg Capsule (Berk Pharmaceuticals Ltd)
34265	Atenolol 50mg tablets (Sandoz Ltd)
34280	Co-amilofruse 2.5mg/20mg tablets (Sandoz Ltd)
34296	Spironolactone 25mg tablets (A A H Pharmaceuticals Ltd)
34324	Amiloride 5mg tablets (Teva UK Ltd)
34342	Doxazosin 1mg tablets (Teva UK Ltd)
34347	Spironolactone 25mg tablets (Actavis UK Ltd)
34357	Ramipril 10mg capsules (Genus Pharmaceuticals Ltd)
34365	Atenolol 50mg tablets (Teva UK Ltd)
34367	Co-amilozide 2.5mg/25mg tablets (Wockhardt UK Ltd)
34371	Sotalol 40mg tablets (A A H Pharmaceuticals Ltd)
34374	Furosemide 40mg tablets (Teva UK Ltd)
34377	Diltiazem HCl 90mg Modified-release capsule (Hillcross Pharmaceuticals Ltd)
34378	Propranolol 10mg tablets (A A H Pharmaceuticals Ltd)
34382	Ramipril 5mg capsules (Zentiva)
34390	Ramipril 5mg capsules (Genus Pharmaceuticals Ltd)
34400	Enalapril 5mg Tablet (Dowelhurst Ltd)
34407	Metoprolol 50mg tablets (Teva UK Ltd)
34412	Ramipril 5mg capsules (Teva UK Ltd)
34429	Ramipril 5mg capsules (Generics (UK) Ltd)
34430	Metoprolol 50mg tablets (Actavis UK Ltd)

34431	Ramipril 2.5mg capsules (Zentiva)
34432	Ramipril 2.5mg capsules (Genus Pharmaceuticals Ltd)
34443	Atenolol 50mg tablets (Wockhardt UK Ltd)
34449	Co-tenidone 50mg/12.5mg tablets (Generics (UK) Ltd)
34453	Enalapril 20mg tablets (Generics (UK) Ltd)
34471	Lisinopril 5mg tablets (Generics (UK) Ltd)
34475	Diltiazem HCl 90mg Modified-release tablet (IVAX Pharmaceuticals UK Ltd)
34490	Ramipril 2.5mg capsules (Teva UK Ltd)
34492	Atenolol 25mg tablets (Generics (UK) Ltd)
34501	Carvedilol 12.5mg tablets (Actavis UK Ltd)
34505	Ramipril 2.5mg capsules (Sandoz Ltd)
34509	Metoprolol 100mg tablets (Generics (UK) Ltd)
34520	Sotalol 80mg tablets (Generics (UK) Ltd)
34522	Nifedipine 5mg capsules (A A H Pharmaceuticals Ltd)
34528	Ramipril 2.5mg capsules (A A H Pharmaceuticals Ltd)
34539	Ramipril 5mg capsules (Sandoz Ltd)
34540	Ramipril 5mg capsules (A A H Pharmaceuticals Ltd)
34544	Captopril 12.5mg Tablet (IVAX Pharmaceuticals UK Ltd)
34551	Indapamide 2.5mg tablets (Generics (UK) Ltd)
34553	Doxazosin 4mg tablets (Generics (UK) Ltd)
34557	Furosemide 40mg tablets (IVAX Pharmaceuticals UK Ltd)
34562	Captopril 25mg Tablet (IVAX Pharmaceuticals UK Ltd)
34567	Ramipril 2.5mg capsules (Generics (UK) Ltd)
34575	Atenolol 25mg tablets (Wockhardt UK Ltd)
34581	Diltiazem HCl 60mg Modified-release tablet (Kent Pharmaceuticals Ltd)
34583	Ramipril 10mg Capsule (Dexcel-Pharma Ltd)
34584	Metoprolol 50mg tablets (IVAX Pharmaceuticals UK Ltd)
34585	Atenolol 25mg tablets (Sandoz Ltd)
34589	Ramipril 5mg Capsule (Dexcel-Pharma Ltd)
34600	Sotalol 40mg tablets (Teva UK Ltd)
34601	Doxazosin 1mg tablets (Generics (UK) Ltd)
34602	Bendroflumethiazide 2.5mg tablets (Sovereign Medical Ltd)
34607	Nifedipine 5mg capsules (IVAX Pharmaceuticals UK Ltd)
34613	Bumetanide 5mg tablets (Teva UK Ltd)
34622	Co-amilofruse 10mg/80mg tablets (Wockhardt UK Ltd)
34625	Doxazosin 2mg tablets (A A H Pharmaceuticals Ltd)
34640	Sotalol 40mg Tablet (Tillomed Laboratories Ltd)
34651	Ramipril 10mg capsules (Generics (UK) Ltd)
34652	Ramipril 5mg Capsule (Sovereign Medical Ltd)
34657	Ramipril 10mg capsules (Zentiva)
34690	Sotalol 80mg tablets (Sandoz Ltd)
34695	Atenolol 50mg tablets (Kent Pharmaceuticals Ltd)
34696	Lisinopril 20mg tablets (Generics (UK) Ltd)
34698	Ramipril 1.25mg capsules (Zentiva)
34710	Ramipril 10mg capsules (Sandoz Ltd)

34712	Enalapril 20mg tablets (Kent Pharmaceuticals Ltd)
34715	Doxazosin 1mg tablets (A A H Pharmaceuticals Ltd)
34719	Captopril 50mg Tablet (Generics (UK) Ltd)
34732	Ramipril 2.5mg Capsule (Dexcel-Pharma Ltd)
34740	Carvedilol 6.25mg tablets (Actavis UK Ltd)
34741	Carvedilol 3.125mg tablets (IVAX Pharmaceuticals UK Ltd)
34750	Amiloride 5mg tablets (Actavis UK Ltd)
34754	Atenolol 100mg tablets (Sandoz Ltd)
34768	Enalapril 20mg tablets (IVAX Pharmaceuticals UK Ltd)
34783	Propranolol 10mg tablets (Actavis UK Ltd)
34798	Enalapril 20mg tablets (Sandoz Ltd)
34799	Lisinopril 20mg tablets (Zentiva)
34803	Bendroflumethiazide 2.5mg Tablet (Regent Laboratories Ltd)
34804	Propranolol 10mg tablets (Teva UK Ltd)
34821	Bisoprolol 10mg tablets (Generics (UK) Ltd)
34824	Diltiazem HCl 120mg Modified-release tablet (IVAX Pharmaceuticals UK Ltd)
34825	Co-tenidone 50mg/12.5mg tablets (Teva UK Ltd)
34854	Metoprolol 100mg tablets (Actavis UK Ltd)
34867	Propranolol 80mg Capsule (IVAX Pharmaceuticals UK Ltd)
34868	Propranolol 40mg tablets (Teva UK Ltd)
34877	Ramipril 10mg Capsule (Sovereign Medical Ltd)
34882	Atenolol 50mg Tablet (Berk Pharmaceuticals Ltd)
34884	Propranolol 160mg Modified-release capsule (Sandoz Ltd)
34890	Metoprolol 50mg Tablet (Berk Pharmaceuticals Ltd)
34893	Ramipril 10mg Capsule (IVAX Pharmaceuticals UK Ltd)
34899	Co-tenidone 100mg/25mg tablets (A A H Pharmaceuticals Ltd)
34908	Spironolactone 25mg tablets (IVAX Pharmaceuticals UK Ltd)
34925	Metoprolol 50mg tablets (Sandoz Ltd)
34934	Bumetanide 1mg tablets (Generics (UK) Ltd)
34936	Captopril 25mg Tablet (Lagap)
34937	Captopril 50mg Tablet (IVAX Pharmaceuticals UK Ltd)
34943	Ramipril 10mg capsules (A A H Pharmaceuticals Ltd)
34945	Propranolol 160mg Modified-release capsule (Lagap)
34949	Propranolol 160mg Modified-release capsule (Actavis UK Ltd)
34952	Enalapril 10mg tablets (Generics (UK) Ltd)
34953	Enalapril 20mg tablets (Zentiva)
34959	Verapamil 40mg tablets (A A H Pharmaceuticals Ltd)
34963	Bisoprolol 5mg tablets (Actavis UK Ltd)
34975	Nifedipine 5mg capsules (Teva UK Ltd)
34976	Atenolol 25mg tablets (Tillomed Laboratories Ltd)
35007	Ramipril 10mg/5ml oral suspension
35054	Celectol 200mg tablets (Zentiva)
35058	Diffundox XL 400microgram capsules (Zentiva)
35062	Trasicor 20mg tablets (Amdipharm Plc)
35084	Vascalpha 5mg modified-release tablets (Actavis UK Ltd)

35096	Exforge 10mg/160mg tablets (Novartis Pharmaceuticals UK Ltd)
35162	Furosemide 20mg/2ml solution for injection ampoules
35173	Valsartan 160mg with amlodipine 5mg tablets
35174	Valsartan 80mg with amlodipine 5mg tablets
35189	Amlodipine 10mg / Valsartan 160mg tablets
35192	Sildenafil 10mg/5ml oral solution
35196	CoAprovel 300mg/25mg tablets (Sanofi)
35272	Doxadura XL 4mg tablets (Discovery Pharmaceuticals Ltd)
35302	Captopril 12.5mg/5ml oral suspension
35304	Valsartan 160mg with amlodipine 10mg tablets
35312	Bazetham MR 400microgram capsules (Teva UK Ltd)
35317	Exforge 5mg/80mg tablets (Novartis Pharmaceuticals UK Ltd)
35329	Amlodipine 5mg / Valsartan 80mg tablets
35343	Amlodipine 5mg / Valsartan 160mg tablets
35380	Hydrochlorothiazide with olmesartan medoxomil 12.5mg with 20mg tablet
35466	Alphacard MR 400microgram capsules (Ratiopharm UK Ltd)
35481	Irbesartan 300mg / Hydrochlorothiazide 25mg tablets
35592	Cardioplen XL 2.5mg tablets (Chiesi Ltd)
35603	Doxazosin 4mg/5ml oral suspension
35646	Neozipine XL 60mg tablets (Kent Pharmaceuticals Ltd)
35695	Visken 5mg tablets (AMCo)
35696	Kenzem SR 120mg capsules (Kent Pharmaceuticals Ltd)
35697	Exforge 5mg/160mg tablets (Novartis Pharmaceuticals UK Ltd)
35710	Sotalol 25mg/5ml oral suspension
35729	Verapamil 80mg tablets (Teva UK Ltd)
35731	Perindopril erbumine 8mg tablets (A A H Pharmaceuticals Ltd)
35778	Labrocol 100mg Tablet (Lagap)
35789	Spironolactone 25mg Tablet (Celltech Pharma Europe Ltd)
35794	Enalapril 5mg tablets (A A H Pharmaceuticals Ltd)
35925	Contiflo XL 400microgram capsules (Ranbaxy (UK) Ltd)
35938	Propranolol 80mg modified-release capsules (A A H Pharmaceuticals Ltd)
35940	Celectol 400mg tablets (Zentiva)
36023	Cardozin xl 4mg Tablet (Hillcross Pharmaceuticals Ltd)
36190	Furosemide 5mg/5ml oral solution sugar free
36202	Amlodipine 10mg tablets (Actavis UK Ltd)
36261	Atenolol 50mg tablets (Tillomed Laboratories Ltd)
36282	Morvesin XL 400microgram capsules (Sandoz Ltd)
36519	Esidrex -k Tablet (Novartis Pharmaceuticals UK Ltd)
36576	Propranolol 10mg tablets (Generics (UK) Ltd)
36583	Zemret xl 180mg Capsule (Neo Laboratories Ltd)
36603	Propranolol SR 160mg Modified-release capsule (Hillcross Pharmaceuticals Ltd)
36612	Sodium nitroprusside 50mg powder for solution for infusion vials
36620	Parmid XL 10mg tablets (Sandoz Ltd)
36629	Aliskiren 150mg tablets
36649	Hytrin 2mg tablets (AMCo)

36664	Zemret xl 300mg Capsule (Neo Laboratories Ltd)
36740	Slocinx XL 4mg tablets (Zentiva)
36742	Captopril 2mg/5ml oral suspension
36753	Ednyt 10mg Tablet (Dominion Pharma)
36767	Bumetanide 1mg tablets (IVAX Pharmaceuticals UK Ltd)
36780	Hytrin 5mg tablets (AMCo)
36878	Rasilez 150mg tablets (Novartis Pharmaceuticals UK Ltd)
36879	Rasilez 300mg tablets (Novartis Pharmaceuticals UK Ltd)
36909	Aliskiren 300mg tablets
36939	Irbesartan 300mg/5ml oral suspension
37025	Nifedipine 20mg modified-release tablets
37080	Enalapril 5mg/5ml oral solution
37085	Sitaxentan 100mg tablets
37087	Enalapril 5mg/5ml oral suspension
37118	Bisoprolol 2.5mg tablets (A A H Pharmaceuticals Ltd)
37184	Valni XL 30mg tablets (Zentiva)
37243	Cardozin xl 4mg Tablet (Teva UK Ltd)
37294	Triamterene with chlortalidone 50mg + 25mg Tablet
37428	Hytrin 10mg tablets (AMCo)
37530	Neozipine XL 30mg tablets (Kent Pharmaceuticals Ltd)
37573	Valsartan 320mg tablets
37650	Losartan 100mg / Hydrochlorothiazide 12.5mg tablets
37655	Captopril 25mg tablets (Teva UK Ltd)
37710	Lisinopril 10mg / Hydrochlorothiazide 12.5mg tablets (Teva UK Ltd)
37725	Co-tenidone 100mg/25mg tablets (Generics (UK) Ltd)
37726	Nifedipine 100mg/5ml oral suspension
37747	Cozaar-Comp 100mg/12.5mg tablets (Merck Sharp & Dohme Ltd)
37774	Kenzem SR 60mg capsules (Kent Pharmaceuticals Ltd)
37778	Lisinopril 5mg/5ml oral suspension
37837	Bisoprolol 2.5mg Tablet (Teva UK Ltd)
37897	Felotens XL 2.5mg tablets (Thornton & Ross Ltd)
37908	Coversyl Arginine Plus 5mg/1.25mg tablets (Servier Laboratories Ltd)
37930	Perindopril arginine 5mg tablets
37964	Perindopril arginine 2.5mg tablets
37965	Coversyl Arginine 5mg tablets (Servier Laboratories Ltd)
37971	Perindopril arginine 10mg tablets
37978	Perindopril arginine 5mg / Indapamide 1.25mg tablets
38026	Coversyl Arginine 10mg tablets (Servier Laboratories Ltd)
38034	Coversyl Arginine 2.5mg tablets (Servier Laboratories Ltd)
38066	Diltiazem HCl 60mg Modified-release tablet (Lagap)
38107	Nifedipine sr 30mg Tablet (Hillcross Pharmaceuticals Ltd)
38285	Perindopril erbumine 4mg tablets (Teva UK Ltd)
38308	Ramipril 2.5/5mg/10mg tablet
38367	Hydrochlorothiazide with losartan 12.5mg with 100mg Tablet
38370	Labetalol 100mg/20ml solution for injection ampoules

38395	Valsartan 80mg tablets
38433	Propranolol 50mg/5ml Oral solution (Rosemont Pharmaceuticals Ltd)
38434	Keloc SR 10mg tablets (Teva UK Ltd)
38459	Telmisartan 80mg / Hydrochlorothiazide 25mg tablets
38461	Cardozin XL 4mg tablets (Arrow Generics Ltd)
38510	Perindopril erbumine 4mg tablets (Apotex UK Ltd)
38545	Tildiem LA 200 capsules (Sanofi)
38632	Adizem-SR 90mg capsules (Napp Pharmaceuticals Ltd)
38634	Adizem-XL 300mg capsules (Napp Pharmaceuticals Ltd)
38818	Adizem-SR 120mg capsules (Napp Pharmaceuticals Ltd)
38831	Adizem-SR 180mg capsules (Napp Pharmaceuticals Ltd)
38854	Quinapril 20mg/5ml oral solution
38855	Adizem-XL 180mg capsules (Napp Pharmaceuticals Ltd)
38865	Adizem-XL 120mg capsules (Napp Pharmaceuticals Ltd)
38876	Tildiem LA 300 capsules (Sanofi)
38882	Adizem-XL 240mg capsules (Napp Pharmaceuticals Ltd)
38889	MicardisPlus 80mg/25mg tablets (Boehringer Ingelheim Ltd)
38899	Quinil 10mg tablets (Tillomed Laboratories Ltd)
38901	Frumil LS 20mg/2.5mg tablets (Sanofi)
38964	Adizem-SR 120mg tablets (Napp Pharmaceuticals Ltd)
38991	Bisoprolol 7.5mg tablets (A A H Pharmaceuticals Ltd)
38995	Zestoretic 20 tablets (AstraZeneca UK Ltd)
39009	Verapamil 40mg tablets (Teva UK Ltd)
39021	Hydrochlorothiazide with olmesartan medoxomil 25mg with 20mg tablet
39137	Zestoretic 10 tablets (AstraZeneca UK Ltd)
39147	Carace 20 Plus tablets (Merck Sharp & Dohme Ltd)
39171	Bi-Carzem SR 60mg capsules (Tillomed Laboratories Ltd)
39199	Diovan 320mg tablets (Novartis Pharmaceuticals UK Ltd)
39227	Capozide LS 12.5mg/25mg tablets (Bristol-Myers Squibb Pharmaceuticals Ltd)
39233	Propranolol 80mg modified-release capsules (Teva UK Ltd)
39242	Carace 10 Plus tablets (Merck Sharp & Dohme Ltd)
39298	Bi-Carzem SR 90mg capsules (Tillomed Laboratories Ltd)
39355	Tritace 10mg Tablet (Sterwin Medicines)
39357	Neofel XL 2.5mg tablets (Kent Pharmaceuticals Ltd)
39421	Tritace titration pack tablets (Sanofi)
39423	Sotalol 80mg tablets (A A H Pharmaceuticals Ltd)
39447	Varbim XL 1.5mg tablets (Teva UK Ltd)
39512	Captopril 25mg/5ml oral suspension
39602	Bumetanide 1mg tablets (Actavis UK Ltd)
39646	Bisoprolol 0.625mg/5ml oral solution
39786	Olmesartan medoxomil 10mg/5ml oral suspension
39800	Valni XL 60mg tablets (Zentiva)
39804	Amlodipine 5mg tablets (Dr Reddy's Laboratories (UK) Ltd)
39807	Frumil 40mg/5mg tablets (Sanofi)
39846	Vivacor 5mg tablets (Lexon (UK) Ltd)

39914	Amlodipine 5mg tablets (Teva UK Ltd)
39944	Losartan 12.5mg tablets
39984	Sevikar 20mg/5mg tablets (Daiichi Sankyo UK Ltd)
40074	Nifedipine 20mg Capsule
40149	Bendroflumethiazide 5mg tablets (IVAX Pharmaceuticals UK Ltd)
40167	Metoprolol 100mg tablets (IVAX Pharmaceuticals UK Ltd)
40190	Torasemide iv 20mg/4ml Intravenous injection
40240	Labetalol 400mg tablets (A A H Pharmaceuticals Ltd)
40241	Propranolol LA 160mg Capsule (Approved Prescription Services Ltd)
40247	Furosemide 10mg/ml Injection (Martindale Pharmaceuticals Ltd)
40256	Baratol 25mg tablets (Amdipharm Plc)
40310	Moxonidine 200microgram tablets (Teva UK Ltd)
40316	Olmesartan medoxomil 20mg / Amlodipine 5mg tablets
40355	Quinil 5mg tablets (Tillomed Laboratories Ltd)
40384	Ramipril 10mg tablets (A A H Pharmaceuticals Ltd)
40405	Verapamil 120mg tablets (Teva UK Ltd)
40421	Bendogen 50mg Tablet (Lagap)
40527	Ambrisentan 10mg tablets
40528	Ambrisentan 5mg tablets
40571	Cozaar 12.5mg tablets (Merck Sharp & Dohme Ltd)
40633	Vascalpha 5mg modified-release tablets (Almus Pharmaceuticals Ltd)
40639	Olmesartan medoxomil 40mg / Amlodipine 5mg tablets
40668	Olmesartan medoxomil 40mg / Amlodipine 10mg tablets
40678	Doxazosin 4mg tablets (Teva UK Ltd)
40711	Losartan 2.5mg/ml oral suspension sugar free
40738	Torem iv 10mg/2ml Intravenous injection (Boehringer Mannheim UK Ltd)
40761	Nebivolol 2.5mg tablets
40810	Phenoxybenzamine 10mg/5ml oral suspension
40886	Bendroflumethiazide 2.5mg tablets (Almus Pharmaceuticals Ltd)
40891	Doxazosin 2mg tablets (IVAX Pharmaceuticals UK Ltd)
40898	Torasemide 5mg tablets (A A H Pharmaceuticals Ltd)
40899	Thelin 100mg tablets (Pfizer Ltd)
40907	Indapamide 2.5mg tablets (Genus Pharmaceuticals Ltd)
41074	Spironolactone 25mg tablets (Almus Pharmaceuticals Ltd)
41203	Sevikar 40mg/10mg tablets (Daiichi Sankyo UK Ltd)
41205	Sevikar 40mg/5mg tablets (Daiichi Sankyo UK Ltd)
41232	Cozaar 2.5mg/ml oral suspension (Merck Sharp & Dohme Ltd)
41292	Furosemide 20mg tablets (Wockhardt UK Ltd)
41405	Furosemide 500mg tablets (Teva UK Ltd)
41417	Enalapril 2.5mg tablets (A A H Pharmaceuticals Ltd)
41489	Bi-Carzem SR 120mg capsules (Tillomed Laboratories Ltd)
41517	Bendroflumethiazide 5mg tablets (Teva UK Ltd)
41522	Lisopress 20mg tablets (Teva UK Ltd)
41532	Lisopress 5mg tablets (Teva UK Ltd)
41533	Co-amilofruse 2.5mg/20mg tablets (Teva UK Ltd)

41538	Lisopress 2.5mg tablets (Teva UK Ltd)
41543	Doxazosin 1mg tablets (IVAX Pharmaceuticals UK Ltd)
41555	Propranolol 40mg tablets (A A H Pharmaceuticals Ltd)
41556	Co-amilozide 5mg/50mg tablets (Teva UK Ltd)
41572	Co-tenidone 100mg/25mg tablets (Teva UK Ltd)
41573	Lisopress 10mg tablets (Teva UK Ltd)
41586	Verapamil 80mg tablets (Actavis UK Ltd)
41591	Bisoprolol 10mg tablets (Teva UK Ltd)
41592	Spironolactone 100mg tablets (Actavis UK Ltd)
41617	Captopril 25mg tablets (Actavis UK Ltd)
41630	Amiloride 5mg Tablet (IVAX Pharmaceuticals UK Ltd)
41633	Captopril 12.5mg tablets (Actavis UK Ltd)
41635	Diltiazem 60mg modified-release tablets (IVAX Pharmaceuticals UK Ltd)
41639	Hydralazine 50mg tablets (Actavis UK Ltd)
41651	Prazosin 500microgram Tablet (Approved Prescription Services Ltd)
41652	Prazosin 500microgram tablets (A A H Pharmaceuticals Ltd)
41660	Spironolactone 100mg tablets (Teva UK Ltd)
41661	Methyldopa 250mg Tablet (C P Pharmaceuticals Ltd)
41679	Verapamil 80mg tablets (IVAX Pharmaceuticals UK Ltd)
41693	Verapamil 120mg tablets (Generics (UK) Ltd)
41694	Enalapril 2.5mg tablets (IVAX Pharmaceuticals UK Ltd)
41706	Spironolactone 50mg tablets (IVAX Pharmaceuticals UK Ltd)
41719	Co-amilofruse 5mg/40mg tablets (Actavis UK Ltd)
41721	Prazosin 1mg tablets (A A H Pharmaceuticals Ltd)
41740	Celiprolol 200mg tablets (Teva UK Ltd)
41743	Captopril 50mg tablets (Teva UK Ltd)
41746	Enalapril 10mg tablets (Sandoz Ltd)
41827	Labetalol 100mg tablets (Generics (UK) Ltd)
41828	Furosemide 500mg tablets (Actavis UK Ltd)
41861	Tensaid XL 1.5mg tablets (Generics (UK) Ltd)
41885	Ethibide XL 1.5mg tablets (Genus Pharmaceuticals Ltd)
41979	Adipine la 30mg Modified-release tablet (Chiesi Ltd)
42081	Tritace 1.25mg Tablet (Sterwin Medicines)
42142	Moduretic 5mg/50mg tablets (Merck Sharp & Dohme Ltd)
42152	Syprol 50mg/5ml oral solution (Rosemont Pharmaceuticals Ltd)
42210	Amlodipine 10mg tablets (Zentiva)
42285	Quinil 40mg tablets (Tillomed Laboratories Ltd)
42388	Furosemide 40mg/5ml oral solution sugar free (Focus Pharmaceuticals Ltd)
42462	Kirtacap mr 400microgram Capsule (Consilient Health Ltd)
42488	Furosemide 40mg/5ml oral solution sugar free (A A H Pharmaceuticals Ltd)
42625	Vera-Til SR 120mg tablets (Actavis UK Ltd)
42723	Pralenal 5 tablets (Opus Pharmaceuticals Ltd)
42731	Diltiazem sr 120mg Capsule (Hillcross Pharmaceuticals Ltd)
42795	Celiprolol 200mg tablets (Generics (UK) Ltd)
42804	Diltiazem HCl 180mg Capsule (PLIVA Pharma Ltd)

42819	Diltiazem xl 240mg Capsule (Hillcross Pharmaceuticals Ltd)
42894	Enalapril 10mg tablets (Teva UK Ltd)
42901	Enalapril 5mg tablets (Teva UK Ltd)
42902	Enalapril 20mg tablets (Teva UK Ltd)
42906	Indapamide 2.5mg tablets (Niche Generics Ltd)
42908	Enalapril 5mg tablets (IVAX Pharmaceuticals UK Ltd)
42912	Nifedipine 10mg capsules (Teva UK Ltd)
42936	Flomax Relief MR 400microgram capsules (Boehringer Ingelheim Self-Medication Division)
43012	Perindopril erbumine oral solution
43184	Mapemid XL 1.5mg tablets (Teva UK Ltd)
43222	Valni 20 Retard tablets (Tillomed Laboratories Ltd)
43251	Bisoprolol 1.25mg tablets (Generics (UK) Ltd)
43322	Olmesartan medoxomil 40mg / Hydrochlorothiazide 12.5mg tablets
43394	Pinefeld XL 10mg tablets (Tillomed Laboratories Ltd)
43410	Nifedipine extra 60mg Modified-release tablet
43411	Enalapril 5mg tablets (Sandoz Ltd)
43412	Lisinopril 2.5mg tablets (A A H Pharmaceuticals Ltd)
43413	Lisinopril 20mg tablets (A A H Pharmaceuticals Ltd)
43416	Lisinopril 10mg tablets (A A H Pharmaceuticals Ltd)
43418	Lisinopril 5mg tablets (A A H Pharmaceuticals Ltd)
43430	Diltiazem 120mg modified-release tablets (A A H Pharmaceuticals Ltd)
43432	Captopril 6.25mg tablets
43470	Amlodipine 5mg tablets (Wockhardt UK Ltd)
43500	Hydralazine 25mg tablets (Actavis UK Ltd)
43507	Captopril 25mg Tablet (Generics (UK) Ltd)
43508	Co-amilofruse 5mg/40mg tablets (Sandoz Ltd)
43511	Nifedipine 10mg capsules (A A H Pharmaceuticals Ltd)
43512	Felodipine 5mg modified-release tablets (A A H Pharmaceuticals Ltd)
43514	Spironolactone 50mg tablets (A A H Pharmaceuticals Ltd)
43515	Nifedipine 10mg capsules (Actavis UK Ltd)
43516	Indapamide 2.5mg tablets (Actavis UK Ltd)
43523	Amiloride 5mg tablets (Generics (UK) Ltd)
43525	Propranolol 10mg tablets (IVAX Pharmaceuticals UK Ltd)
43531	Moxonidine 400microgram tablets (Sandoz Ltd)
43547	Prazosin 500microgram tablets (IVAX Pharmaceuticals UK Ltd)
43549	Sotalol 40mg tablets (IVAX Pharmaceuticals UK Ltd)
43563	Enalapril 2.5mg tablets (Zentiva)
43564	Bisoprolol 5mg Tablet (PLIVA Pharma Ltd)
43566	Lisinopril 2.5mg tablets (Sandoz Ltd)
43649	Captopril 25mg tablets (A A H Pharmaceuticals Ltd)
43695	Colixil XL 4mg tablets (Sandoz Ltd)
43753	Adalat LA 30 tablets (Bayer Plc)
43790	Vascalpha 10mg modified-release tablets (Almus Pharmaceuticals Ltd)
43813	Perindopril erbumine 2mg tablets (Actavis UK Ltd)
43818	Adalat LA 60 tablets (Bayer Plc)

43879	Vera-Til SR 240mg tablets (Actavis UK Ltd)
43880	Amlodipine 5mg tablets (Almus Pharmaceuticals Ltd)
43915	Olmetec Plus 40mg/12.5mg tablets (Daiichi Sankyo UK Ltd)
43988	Aldomet 250mg tablets (Aspen Pharma Trading Ltd)
43989	Aldomet 500mg tablets (Aspen Pharma Trading Ltd)
44000	Bisoprolol 2.5mg/5ml oral suspension
44083	Labetalol 200mg tablets (Actavis UK Ltd)
44168	Indipam XL 1.5mg tablets (Actavis UK Ltd)
44192	Zemret 240 XL capsules (Tillomed Laboratories Ltd)
44254	Amiloride 5.67mg tablets
44527	Captopril 5mg/ml oral solution sugar free
44553	Pinexel PR 400microgram capsules (Wockhardt UK Ltd)
44657	Ednyt 2.5mg Tablet (Dominion Pharma)
44778	Valsartan 160mg tablets
44808	Nebivolol 2.5mg tablets (A A H Pharmaceuticals Ltd)
44858	Atenolol 25mg tablets (Actavis UK Ltd)
44859	Felodipine sr 5mg Tablet (Approved Prescription Services Ltd)
44887	Bi-carzem xl 300mg Capsule (Tillomed Laboratories Ltd)
45040	Larbex XL 4mg tablets (Teva UK Ltd)
45051	Verapamil hc 240mg Modified-release tablet (Actavis UK Ltd)
45070	Amlodipine 10mg/5ml oral suspension
45078	Spironolactone 25mg/5ml Oral solution sugar free (Rosemont Pharmaceuticals Ltd)
45217	Enalapril 5mg tablets (Kent Pharmaceuticals Ltd)
45228	Captopril capsules
45250	Labetalol 400mg tablets (Sandoz Ltd)
45264	Ramipril 1.25mg capsules (Actavis UK Ltd)
45265	Doxazosin sr 4mg Tablet (Generics (UK) Ltd)
45279	Amlodipine 5mg tablets (Sandoz Ltd)
45289	Metoprolol tartrate Oral solution
45292	Nicardipine 30mg capsules (A A H Pharmaceuticals Ltd)
45297	Propranolol 40mg tablets (IVAX Pharmaceuticals UK Ltd)
45300	Lisinopril 10mg tablets (Actavis UK Ltd)
45305	Bumetanide 1mg tablets (Teva UK Ltd)
45308	Verapamil 240mg modified-release tablets (Generics (UK) Ltd)
45309	Acebutolol 400mg tablets (A A H Pharmaceuticals Ltd)
45319	Perindopril erbumine 2mg tablets (A A H Pharmaceuticals Ltd)
45324	Lisinopril 20mg tablets (Actavis UK Ltd)
45328	Doxazosin 1mg tablets (Sandoz Ltd)
45337	Lisinopril 5mg tablets (Actavis UK Ltd)
45340	Ramipril 10mg Capsule (Actavis UK Ltd)
45342	Doxazosin 4mg tablets (Sandoz Ltd)
45343	Propranolol SR 80mg Modified-release capsule (C P Pharmaceuticals Ltd)
45494	Propranolol 10mg tablets (Almus Pharmaceuticals Ltd)
45554	Ramipril 5mg/5ml oral solution
45578	Clonidine 25microgram tablets (A A H Pharmaceuticals Ltd)

45583	Doxazosin 2mg tablets (Dexcel-Pharma Ltd)
45584	Phenoxybenzamine 100mg/2ml solution for infusion ampoules
45600	Diovan 160mg Tablet (Novartis Pharmaceuticals UK Ltd)
45641	Sildenafil 40mg/50ml solution for injection vials
45685	Adanif XL 30mg tablets (Focus Pharmaceuticals Ltd)
45759	Diltiazem HCl 240mg Capsule (PLIVA Pharma Ltd)
45765	Syprol 40mg/5ml oral solution (Rosemont Pharmaceuticals Ltd)
45816	Lisinopril 5mg tablets (Almus Pharmaceuticals Ltd)
45877	Beta-Prograne 160mg modified-release capsules (Teva UK Ltd)
45916	Hydroflumethiazide with spironolactone 50mg+50mg Tablet
45938	Perindopril erbumine 8mg tablets (Teva UK Ltd)
46009	Verapamil 120mg tablets (Kent Pharmaceuticals Ltd)
46066	Cardozin XL 4mg tablets (Almus Pharmaceuticals Ltd)
46116	Furosemide 10mg/ml Injection (Antigen Pharmaceuticals)
46206	Pamsvax XL 400microgram capsules (Almus Pharmaceuticals Ltd)
46233	Amlodipine Oral solution
46249	Revatio 10mg/12.5ml solution for injection vials (Pfizer Ltd)
46302	Neo-Naclex 2.5mg tablets (AMCo)
46355	Sevikar HCT 20mg/5mg/12.5mg tablets (Daiichi Sankyo UK Ltd)
46363	Half Beta-Prograne 80mg modified-release capsules (Teva UK Ltd)
46365	Quinil 20mg tablets (Tillomed Laboratories Ltd)
46445	Nifedipine 10mg capsules (IVAX Pharmaceuticals UK Ltd)
46525	Torasemide 5mg tablets (Teva UK Ltd)
46526	Raporsin XL 4mg tablets (Actavis UK Ltd)
46614	Lopresor 50mg tablets (Recordati Pharmaceuticals Ltd)
46674	Spironolactone 50mg/5ml Oral suspension sugar free (Rosemont Pharmaceuticals Ltd)
46675	Indapamide 1.5mg modified-release tablets (A A H Pharmaceuticals Ltd)
46687	Olmesartan medoxomil with amlodipine and hydrochlorothiazide 20mg + 5mg + 12.5mg Tablet
46699	Furosemide 40mg tablets (Almus Pharmaceuticals Ltd)
46715	Olmesartan medoxomil with amlodipine and hydrochlorothiazide 40mg + 10mg + 12.5mg Tablet
46724	Amlodipine 5mg/5ml oral solution
46740	Lopresor 100mg tablets (Recordati Pharmaceuticals Ltd)
46792	Olmesartan medoxomil with amlodipine and hydrochlorothiazide 40mg + 5mg + 12.5mg Tablet
46795	Sildenafil 10mg/12.5ml solution for injection vials
46851	Captopril 5mg/5ml oral solution
46884	Verapamil hc 240mg Modified-release tablet (Sandoz Ltd)
46887	Adanif XL 60mg tablets (Focus Pharmaceuticals Ltd)
46890	Ramipril 5mg/5ml oral suspension
46908	Atenolol 100mg tablets (Kent Pharmaceuticals Ltd)
46916	Co-amilozide 5mg/50mg tablets (A A H Pharmaceuticals Ltd)
46922	Prazosin 1mg tablets (IVAX Pharmaceuticals UK Ltd)
46930	Amiloride 5mg tablets (Wockhardt UK Ltd)
46931	Atenolol 100mg tablets (Actavis UK Ltd)

46935	Carvedilol 3.125mg tablets (Actavis UK Ltd)
46936	Carvedilol 3.125mg tablets (A A H Pharmaceuticals Ltd)
46937	Diltiazem 60mg modified-release tablets (Actavis UK Ltd)
46948	Furosemide 40mg tablets (Arrow Generics Ltd)
46951	Captopril 12.5mg tablets (A A H Pharmaceuticals Ltd)
46952	Co-tenidone 100mg/25mg tablets (Actavis UK Ltd)
46955	Verapamil 80mg tablets (Generics (UK) Ltd)
46957	Captopril 12.5mg tablets (Tillomed Laboratories Ltd)
46974	Enalapril 5mg tablets (Generics (UK) Ltd)
46975	Lisinopril 5mg tablets (Sandoz Ltd)
46979	Lisinopril 20mg tablets (Sandoz Ltd)
46990	Spironolactone 50mg/5ml oral suspension
47002	Amlodipine 10mg/5ml sugar free Oral suspension
47006	Losartan 100mg tablets (Teva UK Ltd)
47018	Spironolactone 25mg/5ml oral suspension
47021	Ramipril 2.5mg/5ml oral solution sugar free
47027	Nifedipine 10mg Modified-release tablet (Kent Pharmaceuticals Ltd)
47041	Bisoprolol 2.5mg tablets (Generics (UK) Ltd)
47107	Carvedilol 5mg/5ml oral suspension
47159	Lisinopril 10mg tablets (Almus Pharmaceuticals Ltd)
47217	Adipine la 60mg Modified-release tablet (Chiesi Ltd)
47222	Verapamil 120mg modified-release tablets (A A H Pharmaceuticals Ltd)
47230	Verapamil 240mg modified-release tablets (Teva UK Ltd)
47264	Adcirca 20mg tablets (Eli Lilly and Company Ltd)
47285	Nifedipine xl 60mg Tablet (Hillcross Pharmaceuticals Ltd)
47300	Nebivolol 2.5mg tablets (Glenmark Generics (Europe) Ltd)
47331	Lercanidipine 10mg tablets (Generics (UK) Ltd)
47415	Diltiazem sr 60mg Capsule (Hillcross Pharmaceuticals Ltd)
47467	Olmesartan medoxomil with amlodipine and hydrochlorothiazide 40mg + 5mg + 25mg Tablet
47529	Nifedipine 20mg/ml oral drops
47530	Horizem SR 60mg capsules (Horizon lifecare)
47536	Metoprolol tartrate 12.5mg/5ml Oral suspension
47543	Half Beta-Prograne 80mg modified-release capsules (Actavis UK Ltd)
47573	Sevikar HCT 40mg/5mg/12.5mg tablets (Daiichi Sankyo UK Ltd)
47608	Zemret 300 XL capsules (Tillomed Laboratories Ltd)
47614	Nifedipine 30mg modified-release tablets (A A H Pharmaceuticals Ltd)
47616	Sevikar HCT 40mg/10mg/12.5mg tablets (Daiichi Sankyo UK Ltd)
47647	Co-amilofruse oral liquid
47654	Tracleer 62.5mg tablets (Actelion Pharmaceuticals UK Ltd)
47673	Labetalol 400mg Tablet (Approved Prescription Services Ltd)
47674	Labetalol 200mg Tablet (C P Pharmaceuticals Ltd)
47687	Spiretic 25mg Tablet (DDSA Pharmaceuticals Ltd)
47707	Nifedipine Oral solution
47724	Bi-Carzem XL 240mg capsules (Tillomed Laboratories Ltd)

47727	Sevikar HCT 40mg/5mg/25mg tablets (Daiichi Sankyo UK Ltd)
47732	Zemret 180 XL capsules (Tillomed Laboratories Ltd)
47804	Co-triamterzide 50mg/25mg tablets (A A H Pharmaceuticals Ltd)
47807	Doxazosin xl 4mg Tablet (Hillcross Pharmaceuticals Ltd)
47815	Furosemide 20mg Tablet (Celltech Pharma Europe Ltd)
47833	Bedranol SR 80mg capsules (Almus Pharmaceuticals Ltd)
47844	Bendroflumethiazide 2.5mg tablets (Kent Pharmaceuticals Ltd)
47870	Atenolol 25mg tablets (Almus Pharmaceuticals Ltd)
47887	Nimodrel XL 60mg tablets (Zurich Pharmaceuticals)
47907	Bedranol SR 160mg capsules (Almus Pharmaceuticals Ltd)
47998	Ramipril 2.5mg capsules (Actavis UK Ltd)
48008	Ramipril 5mg capsules (Actavis UK Ltd)
48009	Felodipine 5mg Modified-release tablet (Sandoz Ltd)
48039	Losartan 100mg / Hydrochlorothiazide 12.5mg tablets (Teva UK Ltd)
48049	Perindopril erbumine 2mg tablets (Generics (UK) Ltd)
48053	Ramipril 2.5mg capsules (Almus Pharmaceuticals Ltd)
48079	Indapamide 2.5mg tablets (Zentiva)
48098	Perindopril arginine 4mg with Indapamide 1.25mg tablet
48099	Indapamide 2.5mg tablets (A A H Pharmaceuticals Ltd)
48132	Hydrochlorothiazide Capsule
48150	Doxazosin 1mg tablets (Actavis UK Ltd)
48180	Perindopril erbumine 4mg tablets (Sandoz Ltd)
48189	Ismelin 10mg/1ml solution for injection ampoules (Amdipharm Plc)
48214	Perindopril erbumine 4mg tablets (Actavis UK Ltd)
48272	Diltiazem 60mg modified-release capsules (Alliance Healthcare (Distribution) Ltd)
48282	Diltiazem 90mg modified-release capsules (A A H Pharmaceuticals Ltd)
48288	Diltiazem 120mg modified-release capsules (A A H Pharmaceuticals Ltd)
48398	Losartan 25mg tablets (Dexcel-Pharma Ltd)
48457	Diltiazem 90mg modified-release capsules (Alliance Healthcare (Distribution) Ltd)
48682	Propranolol 50mg/5ml oral solution sugar free
48745	Timolol 10mg / Amiloride 2.5mg / Hydrochlorothiazide 25mg tablets
48870	Adizem-SR 90mg capsules (DE Pharmaceuticals)
49001	Diltiazem 120mg modified-release tablets (Alliance Healthcare (Distribution) Ltd)
49142	Carvedilol 3.125mg/5ml oral suspension
49164	Ramipril 10mg capsules (Actavis UK Ltd)
49268	Furosemide 50mg/5ml oral suspension
49289	Diltiazem 120mg modified-release capsules (Alliance Healthcare (Distribution) Ltd)
49338	Nifedipine 20mg modified-release tablets (Alliance Healthcare (Distribution) Ltd)
49388	Spironolactone 100mg/5ml oral suspension
49390	Diltiazem 90mg modified-release tablets (Alliance Healthcare (Distribution) Ltd)
49411	Sildenafil 10mg/5ml oral suspension
49491	Perindopril erbumine 2mg tablets (Consilient Health Ltd)
49492	Losartan 25mg tablets (Generics (UK) Ltd)
49529	Indapamide 2.5mg tablets (Phoenix Healthcare Distribution Ltd)
49588	Losartan 100mg tablets (A A H Pharmaceuticals Ltd)

49636	Amlodipine 10mg tablets (DE Pharmaceuticals)
49752	Metolazone 2.5mg/5ml oral solution
49762	Nifedipine 10mg modified-release tablets (Alliance Healthcare (Distribution) Ltd)
49863	Propranolol 5mg/5ml oral solution sugar free
49953	Atenolol 25mg tablets (Bristol Laboratories Ltd)
50079	Spironolactone 10mg/5ml oral suspension
50185	Candesartan 8mg tablets (Teva UK Ltd)
50224	Congescor 2.5mg tablets (Teva UK Ltd)
50300	Congescor 1.25mg tablets (Teva UK Ltd)
50334	Enalapril 4mg/5ml oral suspension
50347	Coversyl Arginine 5mg tablets (Waymade Healthcare Plc)
50362	Sildenafil 12.5mg/5ml oral solution
50370	Spironolactone 5mg/5ml oral suspension
50402	Perindopril 2mg Tablet (Servier Laboratories Ltd)
50403	Bisoprolol 1.25mg Tablet (Teva UK Ltd)
50467	Doxazosin 2mg tablets (Alliance Healthcare (Distribution) Ltd)
50509	Ramipril 10mg/5ml oral solution
50514	Bisoprolol 2.5mg tablets (Chanelle Medical UK Ltd)
50607	Perindopril arginine 2mg with Indapamide 625 micrograms tablet
50702	Atenolol 25mg tablets (Alliance Healthcare (Distribution) Ltd)
50780	Enalapril 2mg/5ml oral solution
50863	Enalapril 5mg/5ml oral solution (Drug Tariff Special Order)
50971	Losartan 25mg tablets (A A H Pharmaceuticals Ltd)
51117	Candesartan 8mg tablets (DE Pharmaceuticals)
51186	Losartan 25mg tablets (Arrow Generics Ltd)
51258	Coversyl Arginine Plus 5mg/1.25mg tablets (DE Pharmaceuticals)
51261	Tildiem Retard 120mg tablets (Mawdsley-Brooks & Company Ltd)
51328	Iloprost 50micrograms/0.5ml solution for infusion ampoules
51368	Azilsartan medoxomil 80mg tablets
51433	Lisinopril 20mg tablets (Tillomed Laboratories Ltd)
51447	Metoprolol 12.5mg/5ml oral suspension
51461	Securon SR 240mg tablets (Waymade Healthcare Plc)
51492	Sotalol 25mg/5ml oral solution
51519	Candesartan 8mg tablets (A A H Pharmaceuticals Ltd)
51528	Bisoprolol 1.25mg tablets (Actavis UK Ltd)
51601	Losartan 50mg tablets (Actavis UK Ltd)
51643	Atenolol 25mg/5ml oral solution sugar free (Alliance Healthcare (Distribution) Ltd)
51647	Candesartan 4mg tablets (Mawdsley-Brooks & Company Ltd)
51652	Spironolactone 25mg tablets (DE Pharmaceuticals)
51665	Tamurex 400microgram modified-release capsules (Somex Pharma)
51685	Doxazosin 4mg tablets (Actavis UK Ltd)
51701	Ramipril 5mg capsules (Bristol Laboratories Ltd)
51714	Ramipril 2.5mg capsules (Alliance Healthcare (Distribution) Ltd)
51720	Spironolactone 25mg/5ml oral solution
51807	Coversyl Arginine 5mg tablets (DE Pharmaceuticals)

51897	Edarbi 20mg tablets (Takeda UK Ltd)
51917	Adalat LA 60 tablets (Sigma Pharmaceuticals Plc)
51933	Spironolactone 50mg/5ml oral solution
51983	Furosemide 5mg/5ml oral suspension
51998	Atenolol 25mg tablets (Co-Pharma Ltd)
52010	Enalapril 10mg tablets (Alliance Healthcare (Distribution) Ltd)
52017	Adalat LA 30 tablets (Mawdsley-Brooks & Company Ltd)
52045	Furosemide 250mg/5ml solution for injection vials
52055	Tamsulosin 400microgram oral powder sachets
52088	Lisinopril 5mg tablets (Phoenix Healthcare Distribution Ltd)
52136	Bedranol sr 160mg Capsule (Lagap)
52145	Cyclopenthiazide 0.25mg with oxprenolol 160mg modified-release tablets
52159	Tamsulosin 400microgram modified-release capsules (Focus Pharmaceuticals Ltd)
52189	Losartan 100mg / Hydrochlorothiazide 25mg tablets (A A H Pharmaceuticals Ltd)
52197	Ramipril 5mg capsules (Sigma Pharmaceuticals Plc)
52208	Candesartan 16mg tablets (A A H Pharmaceuticals Ltd)
52276	Adizem-XL 180mg capsules (DE Pharmaceuticals)
52293	Captopril 2mg capsules
52310	Atenolol 25mg tablets (Crescent Pharma Ltd)
52366	Spironolactone 5mg/5ml oral solution
52399	Ramipril 1.25mg capsules (Kent Pharmaceuticals Ltd)
52407	Ramipril 10mg capsules (Kent Pharmaceuticals Ltd)
52427	Cozaar 100mg tablets (Necessity Supplies Ltd)
52440	Amlodipine 10mg/5ml oral solution
52499	Captopril 25mg/5ml oral solution
52500	Atenolol 50mg tablets (Almus Pharmaceuticals Ltd)
52548	Bisoprolol 1.25mg tablets (Almus Pharmaceuticals Ltd)
52555	Clonidine 50micrograms/5ml oral solution
52559	Candesartan 8mg tablets (Zentiva)
52609	Inderal LA 160mg capsules (Sigma Pharmaceuticals Plc)
52611	Bisoprolol 10mg/5ml oral solution
52635	Bisoprolol 5mg tablets (Alliance Healthcare (Distribution) Ltd)
52658	Losartan 100mg/5ml oral suspension
52659	Losartan 50mg/5ml oral solution
52686	Bisoprolol 2.5mg/5ml oral solution
52701	Tildiem LA 200 capsules (Mawdsley-Brooks & Company Ltd)
52728	Beta-Adalat modified-release capsules (Lexon (UK) Ltd)
52777	Propranolol 40mg tablets (Kent Pharmaceuticals Ltd)
52858	Co-Diovan 80mg/12.5mg tablets (Sigma Pharmaceuticals Plc)
52882	Enalapril 5mg/5ml oral suspension sugar free
52886	Losartan 12.5mg tablets (A A H Pharmaceuticals Ltd)
52887	Furosemide 20mg/2ml solution for injection ampoules (A A H Pharmaceuticals Ltd)
52900	Furosemide 80mg/8ml solution for injection Minijet pre-filled syringes (UCB Pharma Ltd)
52970	Spironolactone 10mg/5ml oral solution
52972	Irbesartan 300mg tablets (Sigma Pharmaceuticals Plc)

53033	Doxzogen XL 4mg tablets (Generics (UK) Ltd)
53058	Perindopril erbumine 8mg tablets (Sandoz Ltd)
53084	Cositam XL 400microgram tablets (Consilient Health Ltd)
53142	Clonidine 50micrograms/5ml oral suspension
53177	Propranolol oral solution
53204	Atenolol 50mg tablets (Alliance Healthcare (Distribution) Ltd)
53215	Atenolol 50mg tablets (Bristol Laboratories Ltd)
53220	Sevikar HCT 40mg/10mg/25mg tablets (Daiichi Sankyo UK Ltd)
53253	Spironolactone 50mg/5ml oral suspension (Drug Tariff Special Order)
53271	Lisinopril 10mg tablets (Alliance Healthcare (Distribution) Ltd)
53278	Adalat LA 30 tablets (Necessity Supplies Ltd)
53322	Doxazosin 4mg tablets (Bristol Laboratories Ltd)
53334	Bisoprolol 10mg tablets (A A H Pharmaceuticals Ltd)
53357	Nifedipine 10mg/5ml oral suspension
53414	Atenolol 50mg tablets (Accord Healthcare Ltd)
53500	Adalat LA 30 tablets (DE Pharmaceuticals)
53508	Spironolactone 5mg/5ml / Chlorothiazide 50mg/5ml oral suspension
53551	Lisinopril 20mg tablets (Phoenix Healthcare Distribution Ltd)
53612	Ramipril 10mg tablets (Alliance Healthcare (Distribution) Ltd)
53621	Ramipril 2.5mg capsules (Bristol Laboratories Ltd)
53629	Adalat retard 20mg tablets (Lexon (UK) Ltd)
53664	Bisoprolol 2.5mg tablets (Sandoz Ltd)
53674	Metolazone 2.5mg tablets
53680	Candesartan 16mg tablets (Teva UK Ltd)
53719	Enalapril 20mg tablets (Alliance Healthcare (Distribution) Ltd)
53755	Candesartan 4mg tablets (Teva UK Ltd)
53802	Atenolol 25mg tablets (Sigma Pharmaceuticals Plc)
53812	Bendroflumethiazide oral solution
53820	Lisinopril 5mg tablets (Arrow Generics Ltd)
53826	Atenolol 25mg tablets (Boston Healthcare Ltd)
53833	Valsartan 160mg capsules (Generics (UK) Ltd)
53868	Amlodipine 5mg tablets (Actavis UK Ltd)
53885	Bisoprolol 1.25mg tablets (A A H Pharmaceuticals Ltd)
53896	Iloprost 100micrograms/1ml solution for infusion ampoules
53915	Enalapril 5mg tablets (Dexcel-Pharma Ltd)
53916	Bisoprolol 2.5mg tablets (Almus Pharmaceuticals Ltd)
53964	Tamsulosin 400microgram modified-release capsules (A A H Pharmaceuticals Ltd)
53967	Furosemide 20mg tablets (Bristol Laboratories Ltd)
53990	Nifedipine 5mg/5ml oral suspension
54037	Lisinopril 10mg tablets (Relonchem Ltd)
54049	Losartan 50mg tablets (Accord Healthcare Ltd)
54057	Losartan 50mg tablets (Teva UK Ltd)
54106	Carvedilol 1.5mg/5ml oral suspension
54120	Spironolactone 4mg/5ml oral suspension
54201	Lisinopril 20mg / Hydrochlorothiazide 12.5mg tablets (Almus Pharmaceuticals Ltd)

54283	Lisinopril 5mg/5ml oral suspension (Drug Tariff Special Order)
54288	Lisinopril 10mg tablets (Arrow Generics Ltd)
54297	Propranolol 50mg/5ml oral solution
54298	Ramipril 2.5mg capsules (Arrow Generics Ltd)
54316	Indapamide 2.5mg tablets (Alliance Healthcare (Distribution) Ltd)
54326	Candesartan 32mg tablets (Teva UK Ltd)
54329	Metolazone 5mg/5ml oral suspension
54341	Chlorothiazide 5mg/5ml oral suspension
54345	Trandolapril 4mg capsules (Arrow Generics Ltd)
54404	Losartan 100mg tablets (Actavis UK Ltd)
54414	Candesartan 16mg tablets (Consilient Health Ltd)
54479	Bisoprolol 1.25mg tablets (Alliance Healthcare (Distribution) Ltd)
54487	Nebivolol 2.5mg tablets (Sigma Pharmaceuticals Plc)
54497	Galebon 400microgram modified-release capsules (Consilient Health Ltd)
54512	Lisinopril Oral solution
54515	Amlodipine 10mg tablets (Alliance Healthcare (Distribution) Ltd)
54542	Atenolol 25mg tablets (Zanza Laboratories Ltd)
54544	Captopril 25mg/5ml oral suspension
54620	Ramipril 2.5mg capsules (Sigma Pharmaceuticals Plc)
54623	Beta-Prograne 160mg modified-release capsules (Actavis UK Ltd)
54633	Amlodipine 5mg tablets (Bristol Laboratories Ltd)
54643	Metolazone Oral solution
54654	Amlodipine 10mg tablets (Almus Pharmaceuticals Ltd)
54679	Chlorothiazide 250mg tablets
54696	Amlodipine 10mg tablets (Sandoz Ltd)
54726	Valsartan 40mg capsules (Teva UK Ltd)
54733	Perindopril erbumine 8mg tablets (Consilient Health Ltd)
54735	Losartan 50mg tablets (Alliance Healthcare (Distribution) Ltd)
54740	Losartan 25mg tablets (Actavis UK Ltd)
54752	Atenolol 50mg tablets (Co-Pharma Ltd)
54785	Doxazosin 4mg tablets (Medreich Plc)
54799	Tildiem LA 300 capsules (Mawdsley-Brooks & Company Ltd)
54825	Furosemide 20mg tablets (Sigma Pharmaceuticals Plc)
54843	Losartan 50mg tablets (Dexcel-Pharma Ltd)
54899	Perindopril erbumine 2mg tablets (Teva UK Ltd)
54918	Phenoxybenzamine oral liquid
54928	Lisinopril 10mg tablets (Bristol Laboratories Ltd)
54941	Ramipril 5mg capsules (Alliance Healthcare (Distribution) Ltd)
54942	Perindopril erbumine 8mg tablets (Generics (UK) Ltd)
54983	Amlodipine 2.5mg/5ml oral suspension
54986	Perindopril erbumine 8mg/5ml oral suspension
55002	Lisinopril 20mg tablets (Accord Healthcare Ltd)
55017	Irbesartan 300mg tablets (Accord Healthcare Ltd)
55160	Cozaar-Comp 50mg/12.5mg tablets (Sigma Pharmaceuticals Plc)
55187	Valsartan 160mg capsules (Arrow Generics Ltd)

55228	Propranolol 40mg tablets (Boston Healthcare Ltd)
55257	Diltiazem 60mg/5ml oral solution
55259	Indapamide 2.5mg tablets (Kent Pharmaceuticals Ltd)
55296	Losartan 50mg tablets (Generics (UK) Ltd)
55298	Bisoprolol 10mg tablets (Sigma Pharmaceuticals Plc)
55299	Ramipril 1.25mg capsules (A A H Pharmaceuticals Ltd)
55306	Folpik XL 5mg tablets (Teva UK Ltd)
55358	Olmesartan medoxomil with amlodipine and hydrochlorothiazide 40mg + 10mg + 25mg Tablet
55368	Sildenafil 10mg/ml oral suspension sugar free
55399	Lisinopril 20mg / Hydrochlorothiazide 12.5mg tablets (A A H Pharmaceuticals Ltd)
55416	Propranolol 40mg tablets (Almus Pharmaceuticals Ltd)
55446	Losartan 100mg tablets (Bristol Laboratories Ltd)
55455	Nifedipine 10mg capsules (Co-Pharma Ltd)
55456	Lisinopril 5mg tablets (Alliance Healthcare (Distribution) Ltd)
55548	Bumetanide 1mg tablets (Alliance Healthcare (Distribution) Ltd)
55588	Lisinopril 20mg tablets (Sigma Pharmaceuticals Plc)
55639	Lisinopril 10mg tablets (Accord Healthcare Ltd)
55718	Losartan 25mg tablets (Phoenix Healthcare Distribution Ltd)
55738	Furosemide 50mg/5ml solution for injection ampoules (Hameln Pharmaceuticals Ltd)
55740	Neofel XL 2.5mg tablets (Actavis UK Ltd)
55777	Metolazone 5mg/5ml oral solution
55778	Atenolol 50mg tablets (Phoenix Healthcare Distribution Ltd)
55791	Bisoprolol 3.75mg tablets (Actavis UK Ltd)
55797	Clonidine 25micrograms/5ml oral solution
55798	Ramipril 5mg capsules (Waymade Healthcare Plc)
55821	Valsartan 160mg capsules (Teva UK Ltd)
55824	Nifedipine 20mg Modified-release tablet (Berk Pharmaceuticals Ltd)
55826	Prazosin 5mg tablets (A A H Pharmaceuticals Ltd)
55849	Propranolol 160mg tablets (Generics (UK) Ltd)
55853	Pindolol 15mg Tablet (Hillcross Pharmaceuticals Ltd)
55889	Chlorothiazide oral solution
55896	Lisinopril 2.5mg tablets (Actavis UK Ltd)
55903	Enalapril 10mg tablets (Dexcel-Pharma Ltd)
55906	Doxazosin 1mg tablets (Dexcel-Pharma Ltd)
55916	Doxazosin 1mg tablets (Alliance Healthcare (Distribution) Ltd)
55929	Bisoprolol 5mg tablets (Accord Healthcare Ltd)
55949	Propranolol 40mg/5ml oral solution
55979	Metoprolol 25mg/5ml oral suspension
56013	Ramipril 2.5mg capsules (Waymade Healthcare Plc)
56038	Ramipril 10mg tablets (Pfizer Ltd)
56051	Furosemide 20mg tablets (Kent Pharmaceuticals Ltd)
56067	Spironolactone 4mg/5ml oral solution
56079	Perindopril tosilate 10mg tablets
56104	Losartan 50mg tablets (A A H Pharmaceuticals Ltd)

56129	Ramipril 5mg capsules (Kent Pharmaceuticals Ltd)
56145	Doxazosin 2mg tablets (Actavis UK Ltd)
56147	Amlodipine 10mg tablets (Accord Healthcare Ltd)
56148	Ramipril 1.25mg tablets (Kent Pharmaceuticals Ltd)
56157	Perindopril tosilate 5mg / Indapamide 1.25mg tablets
56162	Perindopril erbumine 4mg tablets (Consilient Health Ltd)
56169	Ramipril 10mg capsules (Arrow Generics Ltd)
56173	Half Beta-Prograne 80mg modified-release capsules (Actavis UK Ltd)
56204	Losartan 50mg / Hydrochlorothiazide 12.5mg tablets (Actavis UK Ltd)
56240	Bisoprolol 3.75mg tablets (Sandoz Ltd)
56244	Lisinopril 20mg / Hydrochlorothiazide 12.5mg tablets (Tillomed Laboratories Ltd)
56274	Spironolactone 4.5mg/5ml oral suspension
56279	Lisinopril 2.5mg/5ml oral solution
56296	Indapamide 2.5mg tablets (Boston Healthcare Ltd)
56334	Amlodipine 10mg tablets (Bristol Laboratories Ltd)
56356	Ramipril 10mg capsules (Alliance Healthcare (Distribution) Ltd)
56375	Furosemide 40mg tablets (Accord Healthcare Ltd)
56445	Atenolol 25mg/5ml oral solution sugar free (A A H Pharmaceuticals Ltd)
56459	Bisoprolol 2.5mg tablets (Accord Healthcare Ltd)
56467	Tildiem 60mg modified-release tablets (DE Pharmaceuticals)
56469	Adalat LA 60 tablets (Necessity Supplies Ltd)
56472	Perindopril erbumine 4mg tablets (Kent Pharmaceuticals Ltd)
56473	Perindopril erbumine 2mg tablets (Sigma Pharmaceuticals Plc)
56485	Celectol 200mg tablets (Waymade Healthcare Plc)
56486	Monocor 10mg tablets (Dowelhurst Ltd)
56505	Zestril 5mg tablets (Lexon (UK) Ltd)
56506	Coversyl 2mg tablets (Dowelhurst Ltd)
56508	Coversyl 4mg tablets (Dowelhurst Ltd)
56509	Capoten 12.5mg tablets (Dowelhurst Ltd)
56510	Zestril 20mg tablets (Sigma Pharmaceuticals Plc)
56516	Perindopril erbumine 2mg tablets (Sandoz Ltd)
56536	Spironolactone 100mg/5ml oral solution
56606	Azilsartan medoxomil 40mg tablets
56704	Ramipril 1.25mg capsules (Alliance Healthcare (Distribution) Ltd)
56758	Diltiazem 90mg modified-release capsules (Cubic Pharmaceuticals Ltd)
56760	Indapamide 2.5mg tablets (Co-Pharma Ltd)
56763	Ramipril 10mg capsules (Phoenix Healthcare Distribution Ltd)
56764	Propranolol 40mg tablets (Waymade Healthcare Plc)
56767	Lercanidipine 20mg tablets (Generics (UK) Ltd)
56768	Bisoprolol 2.5mg tablets (Niche Generics Ltd)
56804	Chlorothiazide 25mg/5ml oral suspension
56850	Ecopace 12.5mg tablets (AMCo)
56855	Ramipril 10mg capsules (Sigma Pharmaceuticals Plc)
56970	Losartan 100mg tablets (Pfizer Ltd)
56975	Losartan 50mg / Hydrochlorothiazide 12.5mg tablets (A A H Pharmaceuticals Ltd)

56994	Lacidipine 4mg tablets (Teva UK Ltd)
57023	Bisoprolol 2.5mg tablets (Almus Pharmaceuticals Ltd)
57026	Candesartan 8mg tablets (Waymade Healthcare Plc)
57028	Losartan 100mg tablets (Generics (UK) Ltd)
57048	Lisinopril 10mg tablets (Zentiva)
57063	Bedranol SR 80mg capsules (Almus Pharmaceuticals Ltd)
57073	Ramipril 1.25mg capsules (Waymade Healthcare Plc)
57074	Doxazosin 2mg tablets (Sigma Pharmaceuticals Plc)
57104	Spironolactone 200mg/5ml oral suspension
57145	Terazosin 2mg tablets (A A H Pharmaceuticals Ltd)
57176	Bisoprolol 10mg tablets (Accord Healthcare Ltd)
57208	Diltiazem 120mg modified-release capsules (Cubic Pharmaceuticals Ltd)
57235	Ramipril 1.25mg tablets (Sandoz Ltd)
57240	Metoprolol 50mg/5ml oral suspension (Drug Tariff Special Order)
57266	Candesartan 2mg tablets (Actavis UK Ltd)
57273	Candesartan 8mg tablets (Actavis UK Ltd)
57333	Perindopril tosilate 5mg tablets
57342	Propranolol 40mg tablets (Phoenix Healthcare Distribution Ltd)
57346	Ramipril 10mg capsules (Waymade Healthcare Plc)
57378	Enalapril 2mg/5ml oral suspension
57444	Lercanidipine 10mg tablets (Aptil Pharma Ltd)
57448	Doxazosin 4mg tablets (A A H Pharmaceuticals Ltd)
57488	Hydrochlorothiazide Oral solution
57531	Adalat LA 60 tablets (Waymade Healthcare Plc)
57539	Zestoretic 10 tablets (Sigma Pharmaceuticals Plc)
57556	Spironolactone 12mg/5ml oral solution
57567	Propranolol 10mg/5ml oral suspension
57573	Celectol 200mg tablets (Dowelhurst Ltd)
57578	Cardicor 2.5mg tablets (Necessity Supplies Ltd)
57588	Zestril 2.5mg tablets (Mawdsley-Brooks & Company Ltd)
57594	Tildiem 60mg modified-release tablets (Waymade Healthcare Plc)
57600	Furosemide 20mg/2ml solution for injection ampoules (Alliance Healthcare (Distribution) Ltd)
57610	Furosemide 20mg/5ml oral solution sugar free (Focus Pharmaceuticals Ltd)
57626	Bisoprolol 1.25mg/5ml oral solution
57653	Adalat LA 20 tablets (Sigma Pharmaceuticals Plc)
57658	Ramipril 1.25mg tablets (A A H Pharmaceuticals Ltd)
57680	Lacidipine 4mg tablets (A A H Pharmaceuticals Ltd)
57701	Perindopril erbumine 8mg tablets (Actavis UK Ltd)
57784	Doxazosin 2mg/5ml oral suspension
57796	Cozaar-Comp 50mg/12.5mg tablets (DE Pharmaceuticals)
57801	Perindopril erbumine 4mg tablets (Glenmark Generics (Europe) Ltd)
57817	Atenolol 50mg tablets (Zentiva)
57859	Diltiazem 90mg modified-release tablets (Cubic Pharmaceuticals Ltd)
57864	Ramipril 5mg tablets (Sigma Pharmaceuticals Plc)

57882	Enalapril 2.5mg/5ml oral suspension
57908	Co-amilofruse 5mg/40mg tablets (Kent Pharmaceuticals Ltd)
57933	Spironolactone 40mg/5ml oral suspension
57934	Bisoprolol 5mg tablets (Sandoz Ltd)
57944	Perindopril tosilate 2.5mg tablets
57977	Candesartan 16mg tablets (Alliance Healthcare (Distribution) Ltd)
58077	Spironolactone 8mg/5ml oral suspension
58078	Furosemide 8mg/5ml oral solution
58090	Clonidine 75micrograms/5ml oral solution
58108	Irbesartan 150mg tablets (A A H Pharmaceuticals Ltd)
58109	Bisoprolol 1.25mg/5ml oral suspension
58151	Sildenafil 10mg/5ml oral solution
58195	Captopril 12.5mg/5ml oral solution
58201	Irbesartan 150mg tablets (Actavis UK Ltd)
58224	Furosemide 10mg/5ml oral solution
58225	Spironolactone 2.5mg/5ml oral suspension
58258	Lisinopril 2.5mg/5ml oral suspension
58274	Losartan 25mg tablets (Accord Healthcare Ltd)
58276	Doxazosin 2mg tablets (Medreich Plc)
58294	Lisinopril 5mg tablets (Accord Healthcare Ltd)
58297	Propranolol 10mg tablets (Kent Pharmaceuticals Ltd)
58325	Doxazosin 4mg tablets (Phoenix Healthcare Distribution Ltd)
58339	Neofel XL 2.5mg tablets (Almus Pharmaceuticals Ltd)
58407	Propranolol 80mg tablets (Teva UK Ltd)
58451	Lisinopril 2.5mg tablets (Almus Pharmaceuticals Ltd)
58455	Bisoprolol 7.5mg tablets (Sandoz Ltd)
58461	Lisinopril 2.5mg tablets (Kent Pharmaceuticals Ltd)
58491	Propranolol 40mg tablets (Alliance Healthcare (Distribution) Ltd)
58498	Bisoprolol 2.5mg tablets (Medreich Plc)
58511	Bisoprolol 1.25mg tablets (Sandoz Ltd)
58517	Faramsil 400microgram modified-release tablets (Sandoz Ltd)
58557	Adalat LA 20 tablets (Necessity Supplies Ltd)
58580	Amlodipine 10mg tablets (APC Pharmaceuticals & Chemicals (Europe) Ltd)
58632	Tracleer 125mg tablets (Actelion Pharmaceuticals UK Ltd)
58646	Candesartan 4mg tablets (Actavis UK Ltd)
58649	Losartan 25mg tablets (Bristol Laboratories Ltd)
58669	Valsartan 40mg capsules (Teva UK Ltd)
58682	Lisinopril 2.5mg tablets (Generics (UK) Ltd)
58751	Enalapril 1.25mg/5ml oral suspension
58757	Spironolactone 20mg/5ml oral suspension
58763	Bisoprolol 2.5mg tablets (Waymade Healthcare Plc)
58843	Perindopril erbumine 2mg tablets (Kent Pharmaceuticals Ltd)
58863	Lisinopril 10mg tablets (Phoenix Healthcare Distribution Ltd)
58871	Lisinopril 10mg tablets (Waymade Healthcare Plc)
58874	Perindopril erbumine 2mg tablets (Somex Pharma)

58910	Valsartan 80mg capsules (Sigma Pharmaceuticals Plc)
58967	Losartan 12.5mg tablets (Alliance Healthcare (Distribution) Ltd)
58973	Bisoprolol 10mg tablets (Niche Generics Ltd)
58974	Bisoprolol 2.5mg tablets (Alliance Healthcare (Distribution) Ltd)
58982	Bisoprolol 10mg tablets (Medreich Plc)
58990	Nifedipine 10mg modified-release tablets (Cubic Pharmaceuticals Ltd)
59001	Amlodipine 10mg tablets (Generics (UK) Ltd)
59029	Valsartan 3mg/1ml oral solution
59030	Furosemide 20mg/5ml oral solution
59037	Bisoprolol 5mg tablets (A A H Pharmaceuticals Ltd)
59086	Losartan 25mg tablets (Wockhardt UK Ltd)
59098	Dilzem XL 180 capsules (Lexon (UK) Ltd)
59109	Lisinopril 5mg tablets (Tillomed Laboratories Ltd)
59111	Lisinopril 20mg tablets (Alliance Healthcare (Distribution) Ltd)
59148	Bisoprolol 2.5mg tablets (Zentiva)
59163	Nifedipine 20mg modified-release tablets (Cubic Pharmaceuticals Ltd)
59209	Doxazosin 1mg tablets (Kent Pharmaceuticals Ltd)
59222	Labetalol 100mg/20ml solution for injection ampoules (Focus Pharmaceuticals Ltd)
59233	Lercanidipine 20mg tablets (Actavis UK Ltd)
59264	Securon SR 240mg tablets (DE Pharmaceuticals)
59271	Losartan 25mg tablets (Sandoz Ltd)
59290	Furosemide 20mg tablets (Alliance Healthcare (Distribution) Ltd)
59340	Losartan 12.5mg tablets (Dexcel-Pharma Ltd)
59351	Losartan 50mg tablets (Pfizer Ltd)
59393	Irbesartan 300mg tablets (Sandoz Ltd)
59412	Co-amilofruse 5mg/40mg tablets (Waymade Healthcare Plc)
59415	Propranolol 40mg tablets (Accord Healthcare Ltd)
59448	Valsartan 80mg capsules (A A H Pharmaceuticals Ltd)
59495	Bisoprolol 1.25mg tablets (Teva UK Ltd)
59512	Hydralazine 50mg/5ml oral solution
59549	Carvedilol 5mg/5ml oral suspension
59557	Ramipril 2.5mg capsules (Kent Pharmaceuticals Ltd)
59585	Uard 120XL capsules (Ennogen Healthcare Ltd)
59597	Propranolol 160mg modified-release capsules (A A H Pharmaceuticals Ltd)
59603	Ramipril 2.5mg capsules (Phoenix Healthcare Distribution Ltd)
59616	Rawel XL 1.5mg tablets (Consilient Health Ltd)
59690	Candesartan 8mg tablets (Consilient Health Ltd)
59695	Atenolol 50mg tablets (Boston Healthcare Ltd)
59699	Captopril 5mg/5ml oral solution sugar free
59750	Losartan 50mg tablets (Aptil Pharma Ltd)
59762	Amlodipine 10mg tablets (Teva UK Ltd)
59770	Perindopril erbumine 4mg tablets (Aurobindo Pharma Ltd)
59771	Sildenafil 25mg/5ml oral suspension
59788	Ramipril 10mg capsules (Bristol Laboratories Ltd)
59790	Perindopril erbumine 8mg tablets (Accord Healthcare Ltd)

59802	Candesartan 2mg tablets (Teva UK Ltd)
59834	Chlorothiazide 250mg/5ml oral solution
59862	Doxazosin 4mg tablets (Dexcel-Pharma Ltd)
59863	Dilzem XL 240 capsules (Lexon (UK) Ltd)
59884	Furosemide 20mg tablets (Phoenix Healthcare Distribution Ltd)
59903	Losartan 50mg/5ml oral suspension
59911	Furosemide 40mg tablets (Alliance Healthcare (Distribution) Ltd)
59915	Captopril 25mg/5ml oral solution sugar free
59939	Furosemide 20mg/5ml oral suspension
59961	Nebivolol 10mg tablets
59969	Bisoprolol 5mg tablets (Almus Pharmaceuticals Ltd)
59972	Perindopril erbumine 2mg tablets (Alliance Healthcare (Distribution) Ltd)
59982	Atenolol 25mg tablets (Accord Healthcare Ltd)
59996	Enalapril 20mg tablets (Milpharm Ltd)
60007	Generic Sevikar HCT 40mg/10mg/12.5mg tablets
60010	Lisinopril 10mg tablets (Kent Pharmaceuticals Ltd)
60020	Indapamide 1.5mg modified-release tablets (Waymade Healthcare Plc)
60065	Perindopril erbumine 4mg tablets (Sigma Pharmaceuticals Plc)
60067	Perindopril erbumine 4mg / Amlodipine 5mg tablets
60076	Valsartan 160mg capsules (Waymade Healthcare Plc)
60089	Clonidine 25microgram tablets (Waymade Healthcare Plc)
60097	Lisinopril 2.5mg tablets (Zentiva)
60136	Clonidine 100micrograms/5ml oral solution
60143	Enalapril 5mg tablets (Medreich Plc)
60149	Amiloride 5mg/5ml oral suspension
60200	Doxazosin 4mg tablets (DE Pharmaceuticals)
60232	Lisinopril 5mg tablets (Zentiva)
60244	Amlodipine 10mg tablets (Phoenix Healthcare Distribution Ltd)
60258	Co-amilofruse 2.5mg/20mg tablets (Aurobindo Pharma Ltd)
60291	Furosemide 40mg tablets (AMCo)
60309	Lisinopril 5mg tablets (Relonchem Ltd)
60316	Prazosin 1mg Tablet (Approved Prescription Services Ltd)
60319	Doxazosin 1mg tablets (Bristol Laboratories Ltd)
60343	Spironolactone 25mg tablets (Kent Pharmaceuticals Ltd)
60349	Noyada 25mg/5ml oral solution (Martindale Pharmaceuticals Ltd)
60354	Co-amilozide 2.5mg/25mg tablets (Kent Pharmaceuticals Ltd)
60415	Dilzem XL 180 capsules (Sigma Pharmaceuticals Plc)
60465	Furosemide 5mg/5ml oral solution
60502	Bisoprolol 3.75mg tablets (DE Pharmaceuticals)
60506	Losartan 100mg tablets (Dexcel-Pharma Ltd)
60565	Propranolol 40mg tablets (Ranbaxy (UK) Ltd)
60569	Felodipine 2.5mg modified-release tablets (Waymade Healthcare Plc)
60597	Irbesartan 150mg tablets (Teva UK Ltd)
60603	Chlorothiazide 50mg/5ml oral suspension
60620	Adizem-XL 240mg capsules (Waymade Healthcare Plc)

60652	Parmid XL 2.5mg tablets (Sandoz Ltd)
60660	Spironolactone 3mg/5ml oral suspension
60684	Perindopril erbumine 4mg / Amlodipine 10mg tablets
60699	Lacidipine 2mg tablets (Sigma Pharmaceuticals Plc)
60730	Ramipril 5mg capsules (Phoenix Healthcare Distribution Ltd)
60744	Perindopril erbumine 8mg / Amlodipine 5mg tablets
60757	Trandolapril 500microgram capsules (Teva UK Ltd)
60761	Bisoprolol 1.25mg tablets (Medreich Plc)
60780	Generic Sevikar HCT 20mg/5mg/12.5mg tablets
60823	Noyada 5mg/5ml oral solution (Martindale Pharmaceuticals Ltd)
60856	Nifedipine 10mg modified-release tablets (Sigma Pharmaceuticals Plc)
60884	Felodipine 2.5mg modified-release tablets (Phoenix Healthcare Distribution Ltd)
60896	Bisoprolol 5mg tablets (Medreich Plc)
60898	Moxonidine 200microgram tablets (Generics (UK) Ltd)
60934	Propranolol 80mg modified-release capsules (Kent Pharmaceuticals Ltd)
61010	Diltiazem 120mg modified-release tablets (Cubic Pharmaceuticals Ltd)
61025	Spironolactone 20mg/5ml oral solution
61036	Physiotens 300microgram tablets (Actavis UK Ltd)
61053	Losartan 100mg tablets (Alliance Healthcare (Distribution) Ltd)
61066	Doxazosin 2mg tablets (Bristol Laboratories Ltd)
61067	Ramipril 5mg capsules (Almus Pharmaceuticals Ltd)
61115	Bisoprolol 5mg/5ml oral solution
61116	Hydralazine 50mg/5ml oral suspension
61117	Perindopril erbumine 4mg/5ml oral solution
61123	Terazosin 2mg tablets and Terazosin 1mg tablets
61133	Enalapril 10mg tablets (Phoenix Healthcare Distribution Ltd)
61177	Telmisartan 20mg tablets (Sigma Pharmaceuticals Plc)
61230	Perhexiline maleate 100mg tablets
61245	Diltiazem 60mg modified-release capsules (Sigma Pharmaceuticals Plc)
61256	Clonidine 5micrograms/5ml oral suspension
61262	Lisinopril 20mg tablets (Bristol Laboratories Ltd)
61270	Perindopril erbumine 4mg tablets (Accord Healthcare Ltd)
61283	Doxazosin 4mg tablets (Alliance Healthcare (Distribution) Ltd)
61288	Losartan 100mg tablets (Accord Healthcare Ltd)
61292	Quinapril 40mg tablets (Generics (UK) Ltd)
61339	Ramipril 10mg capsules (Almus Pharmaceuticals Ltd)
61340	Bisoprolol 5mg tablets (DE Pharmaceuticals)
61365	Furosemide 40mg/5ml oral suspension
61374	Amlodipine 4mg/5ml oral suspension
61422	Amlodipine 5mg tablets (Accord Healthcare Ltd)
61442	Valsartan 160mg capsules (Teva UK Ltd)
61475	Furosemide 20mg tablets (DE Pharmaceuticals)
61495	Losartan 25mg tablets (Aptil Pharma Ltd)
61499	Ramipril 2.5mg tablets (Actavis UK Ltd)
61532	Diltiazem 120mg modified-release capsules (Sigma Pharmaceuticals Plc)

61564	Bisoprolol 3.75mg tablets (Waymade Healthcare Plc)
61573	Atenolol 25mg/5ml oral solution
61611	Lercanidipine 10mg tablets (DE Pharmaceuticals)
61651	Bisoprolol 7.5mg tablets (Almus Pharmaceuticals Ltd)
61663	Carvedilol 3.125mg tablets (Teva UK Ltd)
61693	Perindopril erbumine 8mg tablets (Aurobindo Pharma Ltd)
61694	Ramipril 5mg tablets (Zentiva)
61710	Clonidine 25microgram tablets (Teva UK Ltd)
61719	Beta-Adalat modified-release capsules (Waymade Healthcare Plc)
61727	Propranolol 10mg tablets (Accord Healthcare Ltd)
61754	Losartan 25mg/5ml oral suspension
61781	Irbesartan 300mg tablets (Teva UK Ltd)
61846	Zaroxolyn 2.5mg tablets (IDIS)
61985	Ramipril 1.25mg tablets (Teva UK Ltd)
62019	Doxazosin 1mg tablets (Almus Pharmaceuticals Ltd)
62024	Bumetanide 5mg tablets (A A H Pharmaceuticals Ltd)
62035	Candesartan 16mg tablets (Waymade Healthcare Plc)
62036	Ramipril 5mg tablets (Waymade Healthcare Plc)
62039	Ramipril 1.25mg tablets (Zentiva)
62064	Diltiazem 120mg modified-release tablets (Mawdsley-Brooks & Company Ltd)
62065	Diltiazem 90mg modified-release tablets (Colorama Pharmaceuticals Ltd)
62066	Cardide SR 1.5mg tablets (Teva UK Ltd)
62140	Candesartan 4mg tablets (Sandoz Ltd)
62158	Doxazosin 4mg tablets (Almus Pharmaceuticals Ltd)
62207	Adizem-SR 120mg capsules (Waymade Healthcare Plc)
62249	Co-amilozide 5mg/50mg tablets (Alliance Healthcare (Distribution) Ltd)
62300	Phenoxybenzamine 10mg capsules (AMCo)
62325	Atenolol 25mg tablets (Waymade Healthcare Plc)
62337	Irbesartan 300mg / Hydrochlorothiazide 12.5mg tablets (Actavis UK Ltd)
62351	Doxazosin 2mg tablets (Phoenix Healthcare Distribution Ltd)
62361	Bisoprolol 1.25mg tablets (Chanelle Medical UK Ltd)
62376	Actelsar HCT 80mg/12.5mg tablets (Actavis UK Ltd)
62388	Losartan 12.5mg tablets (DE Pharmaceuticals)
62407	Bisoprolol oral solution
62415	Irbesartan 300mg tablets (A A H Pharmaceuticals Ltd)
62513	Methyldopa 250mg tablets (Sovereign Medical Ltd)
62516	Hydrochlorothiazide 12.5mg tablets
62537	Co-tenidone 100mg/25mg tablets (DE Pharmaceuticals)
62552	Verapamil 80mg tablets (Alliance Healthcare (Distribution) Ltd)
62553	Tamsulosin 400microgram modified-release capsules (Alliance Healthcare (Distribution) Ltd)
62564	Lisinopril 10mg/5ml oral solution
62638	Labetalol 100mg tablets (Actavis UK Ltd)
62659	Sildenafil 12.5mg/5ml oral suspension
