# Supplementary material for: Can commonly prescribed drugs be repurposed for the prevention or treatment of Alzheimer's and other neurodegenerative diseases? Protocol for an observational cohort study in the UK Clinical Practice Research Datalink
Source: BMJ Open. 2016 Dec 12;6(12):e012044. doi: 10.1136/bmjopen-2016-012044 (PMC5168636; doi:10.1136/bmjopen-2016-012044)
Supplement: supplementary file [file bmjopen-2016-012044supp2.pdf]

Medical code list: 'at risk' of hypercholesterolaemia

Read Code	Read Code
1W1..00	Possible familial hypercholesterolaemia
44P2.00	Serum cholesterol borderline
44P3.00	Serum cholesterol raised

Medical code list: hypercholesterolaemia

Read Code	Read Code
1W2..00	Probable familial hypercholesterolaemia
44P4.00	Serum cholesterol very high
C320.00	Pure hypercholesterolaemia
C320.11	Familial hypercholesterolaemia
C320000	Familial hypercholesterolaemia
C320600	Polygenic hypercholesterolaemia
C320y00	Other specified pure hypercholesterolaemia
C320z00	Pure hypercholesterolaemia NOS
C329.00	Hypercholesterolaemia

Product code list: treatments for hypercholesterolaemia

Product Code	Product Name
25	Simvastatin 20mg tablets
28	Atorvastatin 10mg tablets
42	Simvastatin 10mg tablets
51	Simvastatin 40mg tablets
75	Atorvastatin 20mg tablets
184	Bezafibrate 200mg tablets
379	Fluvastatin 20mg capsules
420	Cerivastatin 100microgram tablets
490	Pravastatin 10mg tablets
602	Bezafibrate 400mg modified-release tablets
644	Colestyramine 4g oral powder sachets
653	Ezetimibe 10mg tablets
713	Rosuvastatin 10mg tablets
730	Pravastatin 20mg tablets
745	Atorvastatin 40mg tablets
802	Simvador 40mg tablets (Discovery Pharmaceuticals Ltd)
818	Simvastatin 20mg/5ml oral solution sugar free
1212	Colestipol 5g granules sachets sugar free
1214	Bezalip 400mg Tablet (Roche Products Ltd)
1215	Fenofibrate 100mg Capsule
1217	Lipantil micro 200 200mg Capsule (Fournier Pharmaceuticals Ltd)
1219	Pravastatin 40mg tablets
1221	Lipostat 10mg tablets (Bristol-Myers Squibb Pharmaceuticals Ltd)
1223	Lipostat 40mg tablets (Bristol-Myers Squibb Pharmaceuticals Ltd)
1322	Clofibrate 500mg capsules
1324	Bezalip 200mg Tablet (Roche Products Ltd)
1477	Atromid -s 500mg Capsule (AstraZeneca UK Ltd)
1716	Questran 4g oral powder sachets (Bristol-Myers Squibb Pharmaceuticals Ltd)
1764	Questran Light 4g oral powder sachets (Bristol-Myers Squibb Pharmaceuticals Ltd)
2137	Fluvastatin 40mg capsules
2215	Lopid 300mg capsules (Pfizer Ltd)
2435	Lipantil 100mg Capsule (Fournier Pharmaceuticals Ltd)
2662	MaxEPA 1g capsules (Seven Seas Ltd)
2718	Zocor 10mg tablets (Merck Sharp & Dohme Ltd)
2955	Lipitor 40mg tablets (Pfizer Ltd)
3089	Ciprofibrate 100mg tablets
3159	Fenofibrate 200mg capsules
3204	MaxEPA liquid (Seven Seas Ltd)
3318	Gemfibrozil 300mg capsules
3411	Lipitor 10mg tablets (Pfizer Ltd)
3690	Lipostat 20mg tablets (Bristol-Myers Squibb Pharmaceuticals Ltd)
4062	Lopid 600mg tablets (Pfizer Ltd)
4067	Olbetam 250mg capsules (Pfizer Ltd)

4920	Fenofibrate micronised 200mg capsules
4928	Lipantil Micro 200 capsules (Abbott Healthcare Products Ltd)
4961	Lipobay 300microgram Tablet (Bayer Plc)
5009	Cerivastatin 200microgram tablets
5148	Simvastatin 80mg tablets
5216	Bezalip mono 400mg Modified-release tablet (Roche Products Ltd)
5251	Cerivastatin 300microgram tablets
5278	Cerivastatin 400microgram tablets
5390	Fenofibrate micronised 267mg capsules
5564	Colestid Orange sachets (Pharmacia Ltd)
5775	Atorvastatin 80mg tablets
5985	Lescol XL 80mg tablets (Novartis Pharmaceuticals UK Ltd)
6024	Eicosapentaenoic acid 460mg / Docosahexaenoic acid 380mg capsules
6120	Ezetrol 10mg tablets (Merck Sharp & Dohme Ltd)
6155	Colestyramine with aspartame 4g sugar free powder
6168	Zocor 40mg tablets (Merck Sharp & Dohme Ltd)
6213	Rosuvastatin 20mg tablets
6365	Colestid 5g granules sachets plain (Pfizer Ltd)
6572	Omacor capsules (Abbott Healthcare Products Ltd)
7196	Zocor 20mg tablets (Merck Sharp & Dohme Ltd)
7347	Crestor 10mg tablets (AstraZeneca UK Ltd)
7374	Lipitor 20mg tablets (Pfizer Ltd)
7540	Lipantil Micro 267 capsules (Abbott Healthcare Products Ltd)
7544	Niaspan 750mg modified-release tablets (Abbott Laboratories Ltd)
7551	Niaspan 1g modified-release tablets (Abbott Laboratories Ltd)
7552	Simvastatin 20mg / Ezetimibe 10mg tablets
7554	Rosuvastatin 5mg tablets
8082	Gemfibrozil 600mg tablets
8104	Acipimox 250mg capsules
8380	Lescol 20mg capsules (Novartis Pharmaceuticals UK Ltd)
8706	Modalim 100mg tablets (Sanofi)
9153	Lescol 40mg capsules (Novartis Pharmaceuticals UK Ltd)
9315	Lipobay 100microgram Tablet (Bayer Plc)
9316	Lipobay 200microgram Tablet (Bayer Plc)
9491	Fenofibrate micronised 67mg capsules
9639	Fenofibrate micronised 160mg tablets
9716	Supralip 160mg tablets (Abbott Healthcare Products Ltd)
9897	Rosuvastatin 40mg tablets
9920	Simvador 20mg tablets (Discovery Pharmaceuticals Ltd)
9930	Crestor 40mg tablets (AstraZeneca UK Ltd)
10094	Niaspan titration pack (Abbott Laboratories Ltd)
10172	Simvastatin 40mg / Ezetimibe 10mg tablets
10183	Simvastatin 40mg with ezetimibe 10mg tablet
10206	Simvastatin 80mg with ezetimibe 10mg tablet
11627	Fluvastatin 80mg modified-release tablets

11785	Colestyramine 4g oral powder sachets sugar free
11815	Simvastatin 20mg with ezetimibe 10mg tablet
11976	Niaspan 500mg modified-release tablets (Abbott Laboratories Ltd)
12211	Nicotinic acid 50mg tablets
13041	Simvador 10mg tablets (Discovery Pharmaceuticals Ltd)
14037	Eicosapentaenoic acid 170mg / Docosahexaenoic acid 115mg capsules
14209	Eicosapentaenoic acid 170mg/g / Docosahexaenoic acid 115mg/g oral liquid
14219	Simvastatin 80mg / Ezetimibe 10mg tablets
14379	Lipantil Micro 67 capsules (Abbott Healthcare Products Ltd)
14963	Nicotinic acid 500mg modified-release tablets
15209	Crampex tablets (Thornton & Ross Ltd)
15252	Crestor 20mg tablets (AstraZeneca UK Ltd)
16186	Inegy 10mg/80mg tablets (Merck Sharp & Dohme Ltd)
17059	Inegy 10mg/40mg tablets (Merck Sharp & Dohme Ltd)
17614	Zimbacol XL 400mg tablets (Archimedes Pharma UK Ltd)
17683	Lipitor 80mg tablets (Pfizer Ltd)
17688	Crestor 5mg tablets (AstraZeneca UK Ltd)
17813	Nicotinic acid 100mg Tablet
17824	Nicotinic acid 25mg Tablet
18081	Colestid Orange 5g granules sachets (Pfizer Ltd)
18098	Nicotinic acid 375mg + 500mg + 750mg Modified-release tablet
18126	Nicotinic acid 1g modified-release tablets
18442	Lipobay 400microgram Tablet (Bayer Plc)
19938	Colestipol with aspartame granules
21020	Inegy 10mg/20mg tablets (Merck Sharp & Dohme Ltd)
21924	Acetomenapthone 7mg / Nicotinic acid 25mg tablets
22579	Zocor 80mg tablets (Merck Sharp & Dohme Ltd)
23153	Liparol 400 XL tablets (Ashbourne Pharmaceuticals Ltd)
23956	Maxepa Liquid (Seven Seas Ltd)
24084	Colestyramine 4g oral powder sachets sugar free (PLIVA Pharma Ltd)
24583	Nicotinic acid 750mg modified-release tablets
29213	Bezagen XL 400mg tablets (Generics (UK) Ltd)
29328	Bezafibrate 200mg tablets (A A H Pharmaceuticals Ltd)
31221	Bezafibrate 200mg tablets (Generics (UK) Ltd)
31658	Cerivastatin 800microgram tablets
31783	Fenogal 200mg capsules (Genus Pharmaceuticals Ltd)
31930	Zocor heart-pro 10mg Tablet (McNeil Products Ltd)
32110	Colestyramine 4g Sachets (Dominion Pharma)
32909	Simvastatin 80mg tablets (A A H Pharmaceuticals Ltd)
32921	Pravastatin 10mg Tablet (Dr Reddy's Laboratories (UK) Ltd)
33082	Simvastatin 20mg tablets (A A H Pharmaceuticals Ltd)
33603	Fibrazate XL 400mg tablets (Sandoz Ltd)
33944	Bezafibrate 200mg tablets (Teva UK Ltd)
34181	Bezafibrate 400mg Modified-release tablet (Hillcross Pharmaceuticals Ltd)
34201	Colestyramine 4g oral powder sachets sugar free (Actavis UK Ltd)

34277	Gemfibrozil 600mg tablets (Teva UK Ltd)
34312	Simvastatin 20mg tablets (Generics (UK) Ltd)
34316	Simvastatin 20mg tablets (Teva UK Ltd)
34353	Simvastatin 40mg tablets (Generics (UK) Ltd)
34366	Simvastatin 20mg tablets (IVAX Pharmaceuticals UK Ltd)
34376	Simvastatin 40mg tablets (Teva UK Ltd)
34381	Simvastatin 40mg tablets (IVAX Pharmaceuticals UK Ltd)
34476	Simvastatin 20mg Tablet (Ratiopharm UK Ltd)
34481	Simvastatin 10mg tablets (IVAX Pharmaceuticals UK Ltd)
34502	Simvastatin 40mg tablets (A A H Pharmaceuticals Ltd)
34535	Simvastatin 10mg tablets (Generics (UK) Ltd)
34545	Simvastatin 40mg Tablet (Ratiopharm UK Ltd)
34560	Simvastatin 10mg Tablet (Ratiopharm UK Ltd)
34746	Simvastatin 20mg Tablet (Niche Generics Ltd)
34814	Simvastatin 20mg tablets (Wockhardt UK Ltd)
34820	Pravastatin 40mg tablets (A A H Pharmaceuticals Ltd)
34879	Simvastatin 40mg Tablet (Niche Generics Ltd)
34891	Simvastatin 20mg tablets (Kent Pharmaceuticals Ltd)
34907	Simvastatin 40mg tablets (Wockhardt UK Ltd)
34955	Simvastatin 10mg tablets (A A H Pharmaceuticals Ltd)
34969	Simvastatin 40mg tablets (Actavis UK Ltd)
36377	Pravastatin 20mg tablets (Teva UK Ltd)
37266	Colesevelam 625mg tablets
37434	Simvastatin 40mg tablets (Sandoz Ltd)
37953	Cholestagel 625mg tablets (Sanofi)
39060	Simvastatin 20mg tablets (Dexcel-Pharma Ltd)
39420	Bezalip Mono 400mg modified-release tablets (Actavis UK Ltd)
39576	Bezalip 200mg tablets (Actavis UK Ltd)
39652	Simvastatin 40mg/5ml oral solution sugar free
39675	Simvastatin 20mg/5ml Oral suspension (Martindale Pharmaceuticals Ltd)
39870	Simvador 80mg tablets (Discovery Pharmaceuticals Ltd)
40340	Simvastatin 10mg tablets (Teva UK Ltd)
40382	Pravastatin 20mg tablets (A A H Pharmaceuticals Ltd)
40601	Simvastatin 20mg tablets (Ranbaxy (UK) Ltd)
40729	Tredaptive 1000mg/20mg modified-release tablets (Merck Sharp & Dohme Ltd)
40885	Nicotinic acid 1g / Laropiprant 20mg modified-release tablets
41396	Fenofibrate micronised 200mg capsules (A A H Pharmaceuticals Ltd)
41657	Simvastatin 80mg tablets (Teva UK Ltd)
42801	Bezafibrate xl 400mg Modified-release tablet (Generics (UK) Ltd)
43218	Pravastatin 10mg tablets (Teva UK Ltd)
44528	Simvastatin 20mg/5ml oral suspension sugar free (Rosemont Pharmaceuticals Ltd)
44650	Simvastatin 40mg tablets (Dexcel-Pharma Ltd)
44878	Ranzolont 10mg tablets (Ranbaxy (UK) Ltd)
45219	Simvastatin 40mg tablets (Kent Pharmaceuticals Ltd)
45235	Simvastatin 20mg tablets (Sandoz Ltd)

45245	Simvastatin 20mg tablets (Actavis UK Ltd)
45346	Simvastatin 40mg tablets (Arrow Generics Ltd)
46878	Simvastatin 40mg tablets (Almus Pharmaceuticals Ltd)
46956	Simvastatin 80mg tablets (Arrow Generics Ltd)
47065	Atorvastatin 20mg chewable tablets sugar free
47090	Atorvastatin 10mg chewable tablets sugar free
47630	Lipitor 20mg chewable tablets (Pfizer Ltd)
47721	Lipitor 10mg chewable tablets (Pfizer Ltd)
47774	Simvastatin 10mg tablets (Arrow Generics Ltd)
47935	Fenofibrate 200mg Capsule (Teva UK Ltd)
47948	Simvastatin 10mg tablets (Tillomed Laboratories Ltd)
47988	Pravastatin 40mg tablets (Generics (UK) Ltd)
48018	Simvastatin 20mg tablets (Arrow Generics Ltd)
48051	Simvastatin 10mg tablets (Kent Pharmaceuticals Ltd)
48058	Simvastatin 10mg tablets (Ranbaxy (UK) Ltd)
48078	Simvastatin 10mg tablets (Actavis UK Ltd)
48097	Pravastatin 40mg tablets (Teva UK Ltd)
48221	Simvastatin 20mg/5ml oral suspension sugar free
48346	Atorvastatin 60mg tablets
48431	Simvastatin 40mg/5ml oral suspension sugar free
48518	Atorvastatin 10mg/5ml oral solution
48867	Simvastatin 40mg tablets (Alliance Healthcare (Distribution) Ltd)
48973	Atorvastatin 30mg tablets
49061	Simvastatin 40mg tablets (Bristol Laboratories Ltd)
49062	Simvastatin 20mg tablets (Alliance Healthcare (Distribution) Ltd)
49558	Atorvastatin 20mg tablets (A A H Pharmaceuticals Ltd)
49587	Simvastatin 80mg tablets (Almus Pharmaceuticals Ltd)
49609	Bezafibrate 400mg Modified-release tablet (Sandoz Ltd)
49751	Atorvastatin 40mg tablets (Alliance Healthcare (Distribution) Ltd)
50071	Fenofibrate 160mg Tablet (Teva UK Ltd)
50236	Atorvastatin 10mg tablets (Zentiva)
50272	Atorvastatin 40mg tablets (Pfizer Ltd)
50483	Simvastatin 40mg tablets (Relonchem Ltd)
50564	Simvastatin 20mg tablets (Relonchem Ltd)
50670	Simvastatin 40mg tablets (Aurobindo Pharma Ltd)
50703	Simvastatin 40mg tablets (Accord Healthcare Ltd)
50754	Simvastatin 20mg tablets (Medreich Plc)
50788	Atorvastatin 20mg tablets (Pfizer Ltd)
50790	Atorvastatin 20mg tablets (Dexcel-Pharma Ltd)
50882	Simvastatin 40mg tablets (Somex Pharma)
50925	Pravastatin 10mg tablets (Sigma Pharmaceuticals Plc)
50963	Atorvastatin 40mg tablets (Teva UK Ltd)
51085	Simvastatin 10mg tablets (Medreich Plc)
51134	Atorvastatin 10mg tablets (A A H Pharmaceuticals Ltd)
51166	Simvastatin 40mg tablets (Medreich Plc)

51200	Atorvastatin 40mg tablets (Arrow Generics Ltd)
51233	Simvastatin 10mg tablets (Alliance Healthcare (Distribution) Ltd)
51359	Atorvastatin 20mg tablets (Arrow Generics Ltd)
51483	Simvastatin 20mg tablets (Aurobindo Pharma Ltd)
51622	Atorvastatin 20mg tablets (Consilient Health Ltd)
51676	Pravastatin 40mg tablets (Medreich Plc)
51715	Simvastatin 10mg tablets (Sigma Pharmaceuticals Plc)
51876	Atorvastatin 40mg tablets (Consilient Health Ltd)
51890	Pravastatin 20mg tablets (Medreich Plc)
52097	Atorvastatin 40mg tablets (Wockhardt UK Ltd)
52098	Simvastatin 40mg tablets (Ranbaxy (UK) Ltd)
52168	Atorvastatin 20mg tablets (Aspire Pharma Ltd)
52211	Atorvastatin 20mg tablets (Actavis UK Ltd)
52257	Simvastatin 20mg tablets (Accord Healthcare Ltd)
52397	Atorvastatin 40mg tablets (Dr Reddy's Laboratories (UK) Ltd)
52398	Atorvastatin 40mg tablets (A A H Pharmaceuticals Ltd)
52459	Atorvastatin 80mg tablets (Actavis UK Ltd)
52460	Atorvastatin 40mg tablets (Aspire Pharma Ltd)
52625	Simvastatin 10mg tablets (Wockhardt UK Ltd)
52676	Simvastatin 10mg/5ml oral suspension
52755	Pravastatin 20mg tablets (Alliance Healthcare (Distribution) Ltd)
52812	Simvastatin 20mg tablets (Sigma Pharmaceuticals Plc)
52814	Bezafibrate 400mg modified-release tablets (Alliance Healthcare (Distribution) Ltd)
52821	Atorvastatin 80mg tablets (Dr Reddy's Laboratories (UK) Ltd)
52953	Simvastatin 20mg tablets (Bristol Laboratories Ltd)
52962	Simvastatin 80mg tablets (Medreich Plc)
53087	Simvastatin 20mg tablets (Somex Pharma)
53250	Modalim 100mg tablets (Lexon (UK) Ltd)
53340	Zocor 40mg tablets (Lexon (UK) Ltd)
53415	Simvastatin 10mg tablets (Aurobindo Pharma Ltd)
53460	Crestor 10mg tablets (DE Pharmaceuticals)
53594	Lipitor 80mg tablets (Mawdsley-Brooks & Company Ltd)
53676	Simvastatin 20mg tablets (Tillomed Laboratories Ltd)
53770	Fluvastatin 40mg capsules (A A H Pharmaceuticals Ltd)
53772	Atorvastatin 80mg tablets (Alliance Healthcare (Distribution) Ltd)
53822	Simvastatin 10mg tablets (Bristol Laboratories Ltd)
53887	Atorvastatin 40mg tablets (Actavis UK Ltd)
53890	Atorvastatin 80mg tablets (Pfizer Ltd)
53908	Simvastatin 10mg tablets (Dexcel-Pharma Ltd)
53966	Simvastatin 40mg tablets (Phoenix Healthcare Distribution Ltd)
54240	Simvastatin 40mg tablets (Sigma Pharmaceuticals Plc)
54266	Simvastatin 20mg/5ml oral suspension
54435	Pravastatin 40mg tablets (Almus Pharmaceuticals Ltd)
54493	Simvastatin 10mg tablets (Relonchem Ltd)
54535	Atorvastatin 10mg tablets (Pfizer Ltd)

54606	Simvastatin 20mg/5ml oral suspension sugar free (A A H Pharmaceuticals Ltd)
54607	Pravastatin 20mg tablets (Almus Pharmaceuticals Ltd)
54655	Simvastatin 10mg tablets (Accord Healthcare Ltd)
54819	Simvastatin 40mg/5ml oral suspension sugar free (Rosemont Pharmaceuticals Ltd)
54947	Simvastatin 20mg tablets (Almus Pharmaceuticals Ltd)
54976	Simvastatin 10mg tablets (Somex Pharma)
54985	Simvastatin 40mg/5ml oral suspension
54992	Atorvastatin 10mg/5ml oral suspension
55032	Atorvastatin 10mg tablets (Dexcel-Pharma Ltd)
55034	Atorvastatin 40mg/5ml oral suspension
55444	Atorvastatin 40mg tablets (Zentiva)
55452	Simvastatin 20mg tablets (Phoenix Healthcare Distribution Ltd)
55727	Atorvastatin 10mg tablets (Actavis UK Ltd)
55912	Pravastatin 40mg tablets (Alliance Healthcare (Distribution) Ltd)
55971	Prestylon 1g capsules (Teva UK Ltd)
56016	Lipitor 20mg chewable tablets (Pfizer Ltd)
56065	Simvastatin 20mg/5ml oral suspension sugar free (Waymade Healthcare Plc)
56097	Atorvastatin 10mg chewable tablets sugar free
56146	Pravastatin 10mg tablets (Waymade Healthcare Plc)
56165	Atorvastatin 20mg chewable tablets sugar free
56182	Atorvastatin 80mg tablets (Zentiva)
56248	Atorvastatin 20mg tablets (Sigma Pharmaceuticals Plc)
56426	Teromeg 1000mg capsules (AMCo)
56481	Zocor 10mg tablets (Sigma Pharmaceuticals Plc)
56494	Zocor 20mg tablets (Sigma Pharmaceuticals Plc)
56564	Atorvastatin 20mg tablets (Almus Pharmaceuticals Ltd)
56607	Pravastatin 20mg tablets (Waymade Healthcare Plc)
56735	Pravastatin 20mg tablets (Generics (UK) Ltd)
56841	Atorvastatin 40mg tablets (Dexcel-Pharma Ltd)
56893	Pravastatin 40mg tablets (Accord Healthcare Ltd)
56916	Pravastatin 40mg tablets (PLIVA Pharma Ltd)
57108	Pravastatin 40mg tablets (Waymade Healthcare Plc)
57117	Atorvastatin 80mg tablets (Waymade Healthcare Plc)
57137	Pravastatin 10mg tablets (Almus Pharmaceuticals Ltd)
57219	Fenofibrate micronised 200mg capsules (Sandoz Ltd)
57234	Omacor capsules (DE Pharmaceuticals)
57296	Pravastatin 20mg tablets (Phoenix Healthcare Distribution Ltd)
57329	Simvastatin 25mg/5ml oral suspension
57348	Atorvastatin 10mg tablets (Consilient Health Ltd)
57397	Pravastatin 10mg tablets (Accord Healthcare Ltd)
57489	Ciprofibrate 100mg tablets (Zentiva)
57568	Zocor 10mg tablets (Lexon (UK) Ltd)
57583	Eicosapentaenoic acid 460mg / Docosahexaenoic acid 380mg capsules (Huxley Europe Ltd)
57763	Rosuvastatin 10mg tablets (Waymade Healthcare Plc)
57834	Atorvastatin 40mg tablets (DE Pharmaceuticals)

57836	Atorvastatin 80mg tablets (Teva UK Ltd)
57999	Crestor 40mg tablets (Lexon (UK) Ltd)
58041	Atorvastatin 20mg tablets (Teva UK Ltd)
58110	Atorvastatin 20mg tablets (Zentiva)
58315	Simvastatin 20mg tablets (Waymade Healthcare Plc)
58394	Atorvastatin 20mg tablets (Alliance Healthcare (Distribution) Ltd)
58418	Atorvastatin 80mg tablets (A A H Pharmaceuticals Ltd)
58617	Rosuvastatin 20mg/5ml oral suspension
58635	Bezalip Mono 400mg modified-release tablets (DE Pharmaceuticals)
58655	Omega 3-acid-ethyl esters 1000mg capsules (Glenmark Generics (Europe) Ltd)
58742	Atorvastatin 80mg tablets (Arrow Generics Ltd)
58755	Simvastatin 10mg tablets (Phoenix Healthcare Distribution Ltd)
58834	Atorvastatin 10mg tablets (DE Pharmaceuticals)
58868	Atorvastatin 10mg tablets (Sigma Pharmaceuticals Plc)
59002	Bezafibrate 400mg modified-release tablets (DE Pharmaceuticals)
59272	Atorvastatin 20mg tablets (Waymade Healthcare Plc)
59278	Fluvastatin 20mg capsules (Zentiva)
59331	Lipitor 10mg tablets (DE Pharmaceuticals)
59357	Atorvastatin 10mg tablets (Ranbaxy (UK) Ltd)
59446	Atorvastatin 40mg tablets (Almus Pharmaceuticals Ltd)
59447	Crestor 20mg tablets (Waymade Healthcare Plc)
59452	Rosuvastatin 5mg tablets (Waymade Healthcare Plc)
59508	Pravastatin 20mg tablets (Accord Healthcare Ltd)
59776	Atorvastatin 80mg tablets (Aspire Pharma Ltd)
59859	Atorvastatin 10mg tablets (Teva UK Ltd)
60101	Colestyramine 4g oral powder sachets sugar free (Teva UK Ltd)
60160	Rosuvastatin 5mg tablets (Mawdsley-Brooks & Company Ltd)
60251	Pravastatin 10mg tablets (Sandoz Ltd)
60385	Bezalip Mono 400mg modified-release tablets (Lexon (UK) Ltd)
60464	Atorvastatin 20mg/5ml oral suspension
60511	Atorvastatin 40mg tablets (Ranbaxy (UK) Ltd)
60607	Atorvastatin 80mg tablets (DE Pharmaceuticals)
60788	Fenofibrate micronised 267mg capsules (Zentiva)
60989	Atorvastatin 80mg tablets (Phoenix Healthcare Distribution Ltd)
61087	Questran Light 4g oral powder sachets (Mawdsley-Brooks & Company Ltd)
61134	Pravastatin 20mg tablets (Sigma Pharmaceuticals Plc)
61149	Atorvastatin 10mg tablets (Waymade Healthcare Plc)
61155	Simvastatin 40mg/5ml oral suspension sugar free (A A H Pharmaceuticals Ltd)
61269	Nebbaro 1000mg capsules (Zentiva)
61321	Simvastatin 10mg tablets (Sandoz Ltd)
61360	Simvastatin 10mg tablets (Almus Pharmaceuticals Ltd)
61523	Omega 3 1000mg capsules (Alissa Healthcare Research Ltd)
61665	Simvastatin 10mg tablets (Waymade Healthcare Plc)
62137	Simvastatin 40mg tablets (Waymade Healthcare Plc)
62148	Fluvastatin 20mg capsules (Actavis UK Ltd)

62219	Atorvastatin 20mg tablets (DE Pharmaceuticals)
62429	Atorvastatin 20mg tablets (DE Pharmaceuticals)
62476	Atorvastatin 80mg tablets (Almus Pharmaceuticals Ltd)
