# Supplementary material for: Can commonly prescribed drugs be repurposed for the prevention or treatment of Alzheimer's and other neurodegenerative diseases? Protocol for an observational cohort study in the UK Clinical Practice Research Datalink
Source: BMJ Open. 2016 Dec 12;6(12):e012044. doi: 10.1136/bmjopen-2016-012044 (PMC5168636; doi:10.1136/bmjopen-2016-012044)
Supplement: supplementary file [file bmjopen-2016-012044supp3.pdf]

Medical code list: 'at risk' of diabetes

Read Code	Read Term
R10D.00	[D]Elevated blood glucose level
R102.00	[D]Glucose tolerance test abnormal
R105700	[D]Glucose, blood level abnormal
R10D011	[D]Impaired fasting glucose
R10D000	[D]Impaired fasting glycaemia
R10E.00	[D]Impaired glucose tolerance
R102.12	[D]Impaired glucose tolerance test
R102.11	[D]Prediabetes
14O8.00	At risk of diabetes mellitus
44U9.00	Blood glucose abnormal
44Uz.00	Blood glucose raised NOS
C313500	Glucose intolerance
44V2.00	Glucose tol. test impaired
14O8000	High risk of diabetes mellitus
C11y300	Impaired fasting glycaemia
C11y400	Impaired glucose regulation
C11y200	Impaired glucose tolerance
C11y500	Pre-diabetes

Medical code list: type 2 diabetes

Read Code	Read Term
C106100	Diabetes mellitus, adult onset, + neurological manifestation
C105100	Diabetes mellitus, adult onset, + ophthalmic manifestation
C10z100	Diabetes mellitus, adult onset, + unspecified complication
C100100	Diabetes mellitus, adult onset, no mention of complication
C102100	Diabetes mellitus, adult onset, with hyperosmolar coma
C101100	Diabetes mellitus, adult onset, with ketoacidosis
C103100	Diabetes mellitus, adult onset, with ketoacidotic coma
C104100	Diabetes mellitus, adult onset, with renal manifestation
C107200	Diabetes mellitus, adult with gangrene
C10y100	Diabetes mellitus, adult, + other specified manifestation
C107100	Diabetes mellitus, adult, + peripheral circulatory disorder
44V3.00	Glucose tol. test diabetic
C10FK00	Hyperosmolar non-ketotic state in type 2 diabetes mellitus
C109K00	Hyperosmolar non-ketotic state in type 2 diabetes mellitus
C10FK11	Hyperosmolar non-ketotic state in type II diabetes mellitus
C109J11	Insulin treated non-insulin dependent diabetes mellitus
C10FJ00	Insulin treated Type 2 diabetes mellitus
C109J00	Insulin treated Type 2 diabetes mellitus
C109J12	Insulin treated Type II diabetes mellitus
C10FJ11	Insulin treated Type II diabetes mellitus
C109.11	NIDDM - Non-insulin dependent diabetes mellitus
C109E00	Non-insulin depend diabetes mellitus with diabetic cataract
C100112	Non-insulin dependent diabetes mellitus
C109.00	Non-insulin dependent diabetes mellitus
C109700	Non-insulin dependent diabetes mellitus - poor control
C109G00	Non-insulin dependent diabetes mellitus with arthropathy
C109500	Non-insulin dependent diabetes mellitus with gangrene
C109D00	Non-insulin dependent diabetes mellitus with hypoglyca coma
C109A00	Non-insulin dependent diabetes mellitus with mononeuropathy
C109C00	Non-insulin dependent diabetes mellitus with nephropathy
C109B00	Non-insulin dependent diabetes mellitus with polyneuropathy
C109400	Non-insulin dependent diabetes mellitus with ulcer
C109300	Non-insulin-dependent diabetes mellitus with multiple comps
C109200	Non-insulin-dependent diabetes mellitus with neuro comps
C109100	Non-insulin-dependent diabetes mellitus with ophthalm comps
C109000	Non-insulin-dependent diabetes mellitus with renal comps
C109600	Non-insulin-dependent diabetes mellitus with retinopathy
C109900	Non-insulin-dependent diabetes mellitus without complication
L180600	Pre-existing diabetes mellitus, non-insulin-dependent
C10F.00	Type 2 diabetes mellitus
C109.12	Type 2 diabetes mellitus
C10F700	Type 2 diabetes mellitus - poor control
C109712	Type 2 diabetes mellitus - poor control
C109G12	Type 2 diabetes mellitus with arthropathy
C10FG00	Type 2 diabetes mellitus with arthropathy
C109E12	Type 2 diabetes mellitus with diabetic cataract

C10FE00	Type 2 diabetes mellitus with diabetic cataract
C10FQ00	Type 2 diabetes mellitus with exudative maculopathy
C10F500	Type 2 diabetes mellitus with gangrene
C109512	Type 2 diabetes mellitus with gangrene
C10FR00	Type 2 diabetes mellitus with gastroparesis
C10FD00	Type 2 diabetes mellitus with hypoglycaemic coma
C109D12	Type 2 diabetes mellitus with hypoglycaemic coma
C10FN00	Type 2 diabetes mellitus with ketoacidosis
C10FP00	Type 2 diabetes mellitus with ketoacidotic coma
C10FA00	Type 2 diabetes mellitus with mononeuropathy
C10F300	Type 2 diabetes mellitus with multiple complications
C109312	Type 2 diabetes mellitus with multiple complications
C10FC00	Type 2 diabetes mellitus with nephropathy
C109C12	Type 2 diabetes mellitus with nephropathy
C10F200	Type 2 diabetes mellitus with neurological complications
C109212	Type 2 diabetes mellitus with neurological complications
C10FH00	Type 2 diabetes mellitus with neuropathic arthropathy
C109H12	Type 2 diabetes mellitus with neuropathic arthropathy
C10F100	Type 2 diabetes mellitus with ophthalmic complications
C109112	Type 2 diabetes mellitus with ophthalmic complications
C10FF00	Type 2 diabetes mellitus with peripheral angiopathy
C109F12	Type 2 diabetes mellitus with peripheral angiopathy
C10FM00	Type 2 diabetes mellitus with persistent microalbuminuria
C10FL00	Type 2 diabetes mellitus with persistent proteinuria
C10FB00	Type 2 diabetes mellitus with polyneuropathy
C109012	Type 2 diabetes mellitus with renal complications
C10F000	Type 2 diabetes mellitus with renal complications
C10F600	Type 2 diabetes mellitus with retinopathy
C109612	Type 2 diabetes mellitus with retinopathy
C10F400	Type 2 diabetes mellitus with ulcer
C109412	Type 2 diabetes mellitus with ulcer
C10F900	Type 2 diabetes mellitus without complication
C109912	Type 2 diabetes mellitus without complication
C109.13	Type II diabetes mellitus
C10F.11	Type II diabetes mellitus
C109711	Type II diabetes mellitus - poor control
C10F711	Type II diabetes mellitus - poor control
C109G11	Type II diabetes mellitus with arthropathy
C10FG11	Type II diabetes mellitus with arthropathy
C109E11	Type II diabetes mellitus with diabetic cataract
C10FE11	Type II diabetes mellitus with diabetic cataract
C109511	Type II diabetes mellitus with gangrene
C10F511	Type II diabetes mellitus with gangrene
C109D11	Type II diabetes mellitus with hypoglycaemic coma
C10FD11	Type II diabetes mellitus with hypoglycaemic coma
C10FN11	Type II diabetes mellitus with ketoacidosis
C10FP11	Type II diabetes mellitus with ketoacidotic coma
C109A11	Type II diabetes mellitus with mononeuropathy

C10FA11	Type II diabetes mellitus with mononeuropathy
C10F311	Type II diabetes mellitus with multiple complications
C109C11	Type II diabetes mellitus with nephropathy
C10FC11	Type II diabetes mellitus with nephropathy
C109211	Type II diabetes mellitus with neurological complications
C10F211	Type II diabetes mellitus with neurological complications
C109H11	Type II diabetes mellitus with neuropathic arthropathy
C10FH11	Type II diabetes mellitus with neuropathic arthropathy
C109111	Type II diabetes mellitus with ophthalmic complications
C10F111	Type II diabetes mellitus with ophthalmic complications
C109F11	Type II diabetes mellitus with peripheral angiopathy
C10FF11	Type II diabetes mellitus with peripheral angiopathy
C10FM11	Type II diabetes mellitus with persistent microalbuminuria
C10FL11	Type II diabetes mellitus with persistent proteinuria
C109B11	Type II diabetes mellitus with polyneuropathy
C10FB11	Type II diabetes mellitus with polyneuropathy
C109011	Type II diabetes mellitus with renal complications
C10F011	Type II diabetes mellitus with renal complications
C10F611	Type II diabetes mellitus with retinopathy
C109611	Type II diabetes mellitus with retinopathy
C109411	Type II diabetes mellitus with ulcer
C10F411	Type II diabetes mellitus with ulcer
C10F911	Type II diabetes mellitus without complication
C109911	Type II diabetes mellitus without complication

Medical code list: unspecified diabetes

Read Code	Read Term
R054200	[D]Gangrene of toe in diabetic
R054300	[D]Widespread diabetic foot gangrene
Cyu2.00	[X]Diabetes mellitus
Kyu0300	[X]Glomerular disorders in diabetes mellitus
Cyu2000	[X]Other specified diabetes mellitus
Cyu2300	[X]Unspecified diabetes mellitus with renal complications
F372000	Acute painful diabetic neuropathy
F420300	Advanced diabetic maculopathy
F420500	Advanced diabetic retinal disease
F372200	Asymptomatic diabetic neuropathy
F171100	Autonomic neuropathy due to diabetes
F420000	Background diabetic retinopathy
M037200	Cellulitis in diabetic foot
F372100	Chronic painful diabetic neuropathy
K08yA11	Clinical diabetic nephropathy
C10..00	Diabetes mellitus
C10P.00	Diabetes mellitus in remission
C102z00	Diabetes mellitus NOS with hyperosmolar coma
C101z00	Diabetes mellitus NOS with ketoacidosis
C103z00	Diabetes mellitus NOS with ketoacidotic coma
C106z00	Diabetes mellitus NOS with neurological manifestation
C100z00	Diabetes mellitus NOS with no mention of complication
C105z00	Diabetes mellitus NOS with ophthalmic manifestation
C10yz00	Diabetes mellitus NOS with other specified manifestation
C107z00	Diabetes mellitus NOS with peripheral circulatory disorder
C10zz00	Diabetes mellitus NOS with unspecified complication
C107.11	Diabetes mellitus with gangrene
C102.00	Diabetes mellitus with hyperosmolar coma
C101.00	Diabetes mellitus with ketoacidosis
C103.00	Diabetes mellitus with ketoacidotic coma
C104z00	Diabetes mellitus with nephropathy NOS
C106.00	Diabetes mellitus with neurological manifestation
C106.12	Diabetes mellitus with neuropathy
C100.00	Diabetes mellitus with no mention of complication
C105.00	Diabetes mellitus with ophthalmic manifestation
C10y.00	Diabetes mellitus with other specified manifestation
C107.00	Diabetes mellitus with peripheral circulatory disorder
C106.13	Diabetes mellitus with polyneuropathy
C104.00	Diabetes mellitus with renal manifestation
C10z.00	Diabetes mellitus with unspecified complication
C107.12	Diabetes with gangrene
66AI.00	Diabetic - good control
66AJ.00	Diabetic - poor control
66AJz00	Diabetic - poor control NOS
F381311	Diabetic amyotrophy
C106.11	Diabetic amyotrophy

F464000	Diabetic cataract
N030100	Diabetic Charcot arthropathy
N030000	Diabetic cheiroarthropathy
N030011	Diabetic cheiropathy
F440700	Diabetic iritis
F420400	Diabetic maculopathy
F345000	Diabetic mononeuritis multiplex
F35z000	Diabetic mononeuritis NOS
F3y0.00	Diabetic mononeuropathy
C104.11	Diabetic nephropathy
F372.12	Diabetic neuropathy
G73y000	Diabetic peripheral angiopathy
F372.11	Diabetic polyneuropathy
F420.00	Diabetic retinopathy
F420z00	Diabetic retinopathy NOS
8A13.00	Diabetic stabilisation
K27y700	Erectile dysfunction due to diabetes mellitus
2G5C.00	Foot abnormality - diabetes related
2G51000	Foot abnormality - diabetes related
F420800	High risk non proliferative diabetic retinopathy
F420700	High risk proliferative diabetic retinopathy
2BBr.00	Impaired vision due to diabetic retinopathy
M271000	Ischaemic ulcer diabetic foot
C10ER00	Latent autoimmune diabetes mellitus in adult
C10M.00	Lipoatrophic diabetes mellitus
M271200	Mixed diabetic ulcer - foot
F381300	Myasthenic syndrome due to diabetic amyotrophy
K01x100	Nephrotic syndrome in diabetes mellitus
M271100	Neuropathic diabetic ulcer - foot
F420600	Non proliferative diabetic retinopathy
C103y00	Other specified diabetes mellitus with coma
C101y00	Other specified diabetes mellitus with ketoacidosis
C108y00	Other specified diabetes mellitus with multiple comps
C106y00	Other specified diabetes mellitus with neurological comps
C105y00	Other specified diabetes mellitus with ophthalmic complicatn
C10yy00	Other specified diabetes mellitus with other spec comps
C104y00	Other specified diabetes mellitus with renal complications
C10zy00	Other specified diabetes mellitus with unspecified comps
8BL2.00	Patient on maximal tolerated therapy for diabetes
F372.00	Polyneuropathy in diabetes
L180X00	Pre-existing diabetes mellitus, unspecified
F420200	Preproliferative diabetic retinopathy
F420100	Proliferative diabetic retinopathy
K08yA00	Proteinuric diabetic nephropathy
C314.11	Renal diabetes
2BBF.00	Retinal abnormality - diabetes related
C108z00	Unspecified diabetes mellitus with multiple complications
66AJ.11	Unstable diabetes

C108.11	IDDM-Insulin dependent diabetes mellitus
C108900	Insulin dependent diabetes maturity onset
C10E912	Insulin dependent diabetes maturity onset
C100011	Insulin dependent diabetes mellitus
C108.00	Insulin dependent diabetes mellitus
C10E.12	Insulin dependent diabetes mellitus
C108800	Insulin dependent diabetes mellitus - poor control
C10E812	Insulin dependent diabetes mellitus - poor control
C108H00	Insulin dependent diabetes mellitus with arthropathy
C108F00	Insulin dependent diabetes mellitus with diabetic cataract
C10EF12	Insulin dependent diabetes mellitus with diabetic cataract
C108600	Insulin dependent diabetes mellitus with gangrene
C10E612	Insulin dependent diabetes mellitus with gangrene
C108E00	Insulin dependent diabetes mellitus with hypoglycaemic coma
C10EE12	Insulin dependent diabetes mellitus with hypoglycaemic coma
C108B00	Insulin dependent diabetes mellitus with mononeuropathy
C10E312	Insulin dependent diabetes mellitus with multiple complicat
C108300	Insulin dependent diabetes mellitus with multiple complicatn
C108D00	Insulin dependent diabetes mellitus with nephropathy
C10ED12	Insulin dependent diabetes mellitus with nephropathy
C108C00	Insulin dependent diabetes mellitus with polyneuropathy
C10EC12	Insulin dependent diabetes mellitus with polyneuropathy
C108700	Insulin dependent diabetes mellitus with retinopathy
C10E712	Insulin dependent diabetes mellitus with retinopathy
C108500	Insulin dependent diabetes mellitus with ulcer
C10E512	Insulin dependent diabetes mellitus with ulcer
C108200	Insulin-dependent diabetes mellitus with neurological comps
C10E212	Insulin-dependent diabetes mellitus with neurological comps
C108100	Insulin-dependent diabetes mellitus with ophthalmic comps
C10E112	Insulin-dependent diabetes mellitus with ophthalmic comps
C108000	Insulin-dependent diabetes mellitus with renal complications
C10E012	Insulin-dependent diabetes mellitus with renal complications
C108A00	Insulin-dependent diabetes without complication
C10EA12	Insulin-dependent diabetes without complication
ZRbH.00	Perceived control of insulin-dependent diabetes
L180500	Pre-existing diabetes mellitus, insulin-dependent
C108400	Unstable insulin dependent diabetes mellitus
C10E412	Unstable insulin dependent diabetes mellitus

Medical code list: type 1 diabetes

Read Code	Read Term
C107000	Diabetes mellitus, juvenile +peripheral circulatory disorder
C105000	Diabetes mellitus, juvenile type, + ophthalmic manifestation
C10z000	Diabetes mellitus, juvenile type, + unspecified complication
C100000	Diabetes mellitus, juvenile type, no mention of complication
C102000	Diabetes mellitus, juvenile type, with hyperosmolar coma
C101000	Diabetes mellitus, juvenile type, with ketoacidosis
C103000	Diabetes mellitus, juvenile type, with ketoacidotic coma
C104000	Diabetes mellitus, juvenile type, with renal manifestation
C106000	Diabetes mellitus, juvenile, + neurological manifestation
C10E.00	Type 1 diabetes mellitus
C108.12	Type 1 diabetes mellitus
C10E800	Type 1 diabetes mellitus - poor control
C108812	Type 1 diabetes mellitus - poor control
C10E900	Type 1 diabetes mellitus maturity onset
C108912	Type 1 diabetes mellitus maturity onset
C10EH00	Type 1 diabetes mellitus with arthropathy
C10EF00	Type 1 diabetes mellitus with diabetic cataract
C10EP00	Type 1 diabetes mellitus with exudative maculopathy
C10E600	Type 1 diabetes mellitus with gangrene
C10EQ00	Type 1 diabetes mellitus with gastroparesis
C10EE00	Type 1 diabetes mellitus with hypoglycaemic coma
C108E12	Type 1 diabetes mellitus with hypoglycaemic coma
C10EM00	Type 1 diabetes mellitus with ketoacidosis
C10EN00	Type 1 diabetes mellitus with ketoacidotic coma
C10EB00	Type 1 diabetes mellitus with mononeuropathy
C10E300	Type 1 diabetes mellitus with multiple complications
C10ED00	Type 1 diabetes mellitus with nephropathy
C10E200	Type 1 diabetes mellitus with neurological complications
C108212	Type 1 diabetes mellitus with neurological complications
C108J12	Type 1 diabetes mellitus with neuropathic arthropathy
C10EJ00	Type 1 diabetes mellitus with neuropathic arthropathy
C10E100	Type 1 diabetes mellitus with ophthalmic complications
C108112	Type 1 diabetes mellitus with ophthalmic complications
C10EG00	Type 1 diabetes mellitus with peripheral angiopathy
C10EL00	Type 1 diabetes mellitus with persistent microalbuminuria
C10EK00	Type 1 diabetes mellitus with persistent proteinuria
C10EC00	Type 1 diabetes mellitus with polyneuropathy
C108012	Type 1 diabetes mellitus with renal complications
C10E000	Type 1 diabetes mellitus with renal complications
C10E700	Type 1 diabetes mellitus with retinopathy
C108712	Type 1 diabetes mellitus with retinopathy
C10E500	Type 1 diabetes mellitus with ulcer
C108512	Type 1 diabetes mellitus with ulcer
C10EA00	Type 1 diabetes mellitus without complication
C10E.11	Type I diabetes mellitus
C108.13	Type I diabetes mellitus

C108811	Type I diabetes mellitus - poor control
C10E811	Type I diabetes mellitus - poor control
C10P000	Type I diabetes mellitus in remission
C108911	Type I diabetes mellitus maturity onset
C10E911	Type I diabetes mellitus maturity onset
C108H11	Type I diabetes mellitus with arthropathy
C108F11	Type I diabetes mellitus with diabetic cataract
C10EP11	Type I diabetes mellitus with exudative maculopathy
C10E611	Type I diabetes mellitus with gangrene
C10EQ11	Type I diabetes mellitus with gastroparesis
C108E11	Type I diabetes mellitus with hypoglycaemic coma
C10EM11	Type I diabetes mellitus with ketoacidosis
C10EN11	Type I diabetes mellitus with ketoacidotic coma
C108B11	Type I diabetes mellitus with mononeuropathy
C10E311	Type I diabetes mellitus with multiple complications
C108311	Type I diabetes mellitus with multiple complications
C108D11	Type I diabetes mellitus with nephropathy
C108211	Type I diabetes mellitus with neurological complications
C108J11	Type I diabetes mellitus with neuropathic arthropathy
C10E111	Type I diabetes mellitus with ophthalmic complications
C10EL11	Type I diabetes mellitus with persistent microalbuminuria
C10EC11	Type I diabetes mellitus with polyneuropathy
C108011	Type I diabetes mellitus with renal complications
C108711	Type I diabetes mellitus with retinopathy
C10E711	Type I diabetes mellitus with retinopathy
C108511	Type I diabetes mellitus with ulcer
C10E511	Type I diabetes mellitus with ulcer
C10EA11	Type I diabetes mellitus without complication
C108A11	Type I diabetes mellitus without complication
C10E400	Unstable type 1 diabetes mellitus
C108412	Unstable type 1 diabetes mellitus
C10E411	Unstable type I diabetes mellitus
C108411	Unstable type I diabetes mellitus

Product code list: treatments for type 2 diabetes

Product Code	Product Name
4163	Rapitard MC 100unit/ml Injection (Novo Nordisk Ltd)
13416	Insulin biphasic 100 units/ml Injection
16142	Insulin aspart 100units/ml solution for injection 3ml cartridges
53251	NovoRapid Penfill 100units/ml solution for injection 3ml cartridges (DE Pharmaceuticals)
10067	Insulin biphasic aspart human pyr 30:70; 100 units/ml Injection
5892	NovoRapid FlexPen 100units/ml solution for injection 3ml pre-filled pen (Novo Nordisk Ltd)
11337	NovoRapid Novolet 100units/ml solution for injection (Novo Nordisk Ltd)
6061	Novomix 30 30/70 100units/ml Injection (Novo Nordisk Ltd)
53118	NovoRapid FlexPen 100units/ml solution for injection 3ml pre-filled pen (Mawdsley-Brooks & Company Ltd)
29567	Insulin aspart 100units/ml solution for injection 10ml vials
6209	NovoRapid 100units/ml solution for injection 10ml vials (Novo Nordisk Ltd)
6447	Insulin aspart human pyr 100 iu/ml Injection
59533	NovoRapid FlexPen 100units/ml solution for injection 3ml pre-filled pen (Sigma Pharmaceuticals Plc)
51743	NovoRapid Penfill 100units/ml solution for injection 3ml cartridges (Sigma Pharmaceuticals Plc)
49108	NovoRapid Penfill 100units/ml solution for injection 3ml cartridges (Necessity Supplies Ltd)
46666	NovoRapid FlexTouch 100units/ml solution for injection 3ml pre-filled pen (Novo Nordisk Ltd)
19877	Insulin aspart 100units/ml solution for injection 3ml pre-filled disposable devices
5021	NovoRapid Penfill 100units/ml solution for injection 3ml cartridges (Novo Nordisk Ltd)
56489	NovoMix 30 Penfill 100units/ml suspension for injection 3ml cartridges (Waymade Healthcare Plc)
7267	NovoMix 30 Penfill 100units/ml suspension for injection 3ml cartridges (Novo Nordisk Ltd)
23099	Insulin aspart biphasic 30/70 100units/ml suspension for injection 3ml pre-filled disposable devices
7228	NovoMix 30 FlexPen 100units/ml suspension for injection 3ml pre-filled pen (Novo Nordisk Ltd)
24795	Insulin aspart biphasic 30/70 100units/ml suspension for injection 3ml cartridges
55462	Tresiba FlexTouch 100units/ml solution for injection 3ml pre-filled pen (Novo Nordisk Ltd)
55910	Tresiba Penfill 100units/ml solution for injection 3ml cartridges (Novo Nordisk Ltd)
55234	Tresiba FlexTouch 200units/ml solution for injection 3ml pre-filled pen (Novo Nordisk Ltd)
55907	Insulin degludec 100units/ml solution for injection 3ml cartridges
6958	Levemir FlexPen 100units/ml solution for injection 3ml pre-filled pen (Novo Nordisk Ltd)
55618	Levemir FlexPen 100units/ml solution for injection 3ml pre-filled pen (Waymade Healthcare Plc)
35260	Levemir InnoLet 100units/ml solution for injection 3ml pre-filled pen (Novo Nordisk Ltd)
6965	Levemir Penfill 100units/ml solution for injection 3ml cartridges (Novo Nordisk Ltd)
14301	Insulin detemir 100units/ml solution for injection 3ml cartridges

14330	Insulin detemir 100units/ml solution for injection 3ml pre-filled disposable devices
10184	Insulin detemir 100 iu/ml Solution for injection
7400	Insulin glargine 100units/ml solution for injection 3ml pre-filled disposable devices
5953	Insulin glargine 100iu/ml Injection
10225	Lantus 100units/ml solution for injection 3ml OptiClik cartridges (Sanofi)
36853	Lantus 100units/ml solution for injection 3ml pre-filled SoloStar pen (Sanofi)
7266	Lantus 100units/ml solution for injection 3ml cartridges (Sanofi)
10259	Insulin glargine 100units/ml solution for injection 10ml vials
7393	Insulin glargine 100units/ml solution for injection 3ml cartridges
50633	Lantus 100units/ml solution for injection 3ml cartridges (Necessity Supplies Ltd)
6057	Lantus 100iu/ml Injection (Aventis Pharma)
56495	Lantus 100units/ml solution for injection 3ml pre-filled OptiSet pen (Waymade Healthcare Plc)
7237	Lantus 100units/ml solution for injection 3ml pre-filled OptiSet pen (Sanofi)
7402	Lantus 100units/ml solution for injection 10ml vials (Sanofi)
49831	Lantus 100units/ml solution for injection 3ml pre-filled SoloStar pen (Necessity Supplies Ltd)
29953	Apidra 100units/ml solution for injection 3ml OptiClik cartridges (Sanofi)
21583	Apidra 100units/ml solution for injection 3ml pre-filled OptiSet pen (Sanofi)
28101	Insulin glulisine 100units/ml solution for injection 10ml vials
28442	Insulin glulisine 100unit/ml Solution for injection
14345	Apidra 100units/ml solution for injection 3ml cartridges (Sanofi)
21590	Insulin glulisine 100units/ml solution for injection 3ml pre-filled disposable devices
36920	Apidra 100units/ml solution for injection 3ml pre-filled SoloStar pen (Sanofi)
19491	Apidra 100units/ml solution for injection 10ml vials (Sanofi)
14299	Insulin glulisine 100units/ml solution for injection 3ml cartridges
53710	Insulin human 500units/ml solution for injection 20ml vials
36356	Insulin human 3mg inhalation powder blisters
31465	Exubera 1mg inhalation powder blisters (Pfizer Ltd)
62276	Humulin R 500units/ml solution for injection 20ml vials (Imported (United States))
36355	Insulin human 1mg inhalation powder blisters
31467	Exubera 3mg inhalation powder blisters (Pfizer Ltd)
28588	Hypurin Bovine Isophane 100units/ml suspension for injection 3ml cartridges (Wockhardt UK Ltd)
30236	Isophane insulin 100iu/ml Injection
36066	Insulin isophane bovine 100units/ml suspension for injection 3ml cartridges
15484	Insulin isophane bovine 100units/ml suspension for injection 1.5ml cartridges
18590	Insulin isophane bovine 100units/ml suspension for injection 10ml vials
47856	Neuphane 100unit/ml Injection (Wellcome Medical Division)
14340	Hypurin Bovine Isophane 100units/ml suspension for injection 10ml vials (Wockhardt UK Ltd)
13516	Hypurin bovine isophane 100unit/ml Injection (C P Pharmaceuticals Ltd)
38422	Isophane 100iu/ml Injection (Celltech Pharma Europe Ltd)
43950	Humulin I KwikPen 100units/ml suspension for injection 3ml pre-filled pen (Eli Lilly and Company Ltd)
10207	Insulin isophane human 100units/ml suspension for injection 3ml cartridges
14928	Insulatard 100units/ml suspension for injection 10ml vials (Novo Nordisk Ltd)
8118	Humaject i 100iu/ml Pen (Eli Lilly and Company Ltd)

35468	Insuman Basal 100units/ml suspension for injection 5ml vials (Sanofi)
9737	Insulatard innolet 100iu/ml Injection (Novo Nordisk Ltd)
27461	Insuman Basal 100units/ml suspension for injection 3ml cartridges (Sanofi)
33966	Insulatard 100unit/ml Injection (Novo Nordisk Ltd)
4760	Humulin i 100unit/ml Injection (Eli Lilly and Company Ltd)
14918	Humulin I 100units/ml suspension for injection 10ml vials (Eli Lilly and Company Ltd)
13729	Insulin isophane human emp 100unit/ml Injection
1593	Insulatard penfill 100 100iu/ml Penfill (Novo Nordisk Ltd)
23992	Insuman Basal 100units/ml suspension for injection 3ml pre-filled OptiSet pen (Sanofi)
14925	Insulin isophane human vial 100unit/ml Sterile suspension injection
55517	Insulin isophane human 100units/ml suspension for injection 10ml vials
7771	Human protaphane penfill 100 100unit/ml Penfill (Novo Nordisk Ltd)
52748	Insulatard Penfill 100units/ml suspension for injection 3ml cartridges (Waymade Healthcare Plc)
11080	Insulin isophane human prb 100iu/ml Injection
5501	Insuman basal 100iu/ml Injection (Aventis Pharma)
10175	Insulin isophane human 100units/ml suspension for injection 1.5ml cartridges
59500	Insulin isophane human 100units/ml suspension for injection 5ml vials
7772	Human protaphane 100unit/ml Injection (Novo Nordisk Ltd)
1595	Insulatard NovoLet 100units/ml suspension for injection (Novo Nordisk Ltd)
1886	Insulatard 100iu/ml GE injection (Novo Nordisk Ltd)
46001	Insuman Basal 100units/ml suspension for injection 3ml pre-filled SoloStar pen (Sanofi)
5891	Insulatard FlexPen 100units/ml suspension for injection (Novo Nordisk Ltd)
14357	Humulin I 100units/ml suspension for injection 3ml cartridges (Eli Lilly and Company Ltd)
10208	Insulatard InnoLet 100units/ml suspension for injection 3ml pre-filled pen (Novo Nordisk Ltd)
15961	Insulin isophane human crb 100iu/ml Injection
14290	Insulatard Penfill 100units/ml suspension for injection 3ml cartridges (Novo Nordisk Ltd)
25812	Insulin isophane human 100units/ml suspension for injection 3ml pre-filled disposable devices
10229	Humulin I Pen 100units/ml suspension for injection 3ml pre-filled pen (Eli Lilly and Company Ltd)
20422	Insuman comb 15 100iu/ml Injection (Aventis Pharma)
1843	Pork Insulatard 100units/ml suspension for injection 10ml vials (Novo Nordisk Ltd)
7350	Insulin isophane porcine 100units/ml suspension for injection 10ml vials
13819	Hypurin Porcine Isophane 100units/ml suspension for injection 1.5ml cartridges (C P Pharmaceuticals Ltd)
28183	Hypurin Porcine Isophane 100units/ml suspension for injection 10ml vials (Wockhardt UK Ltd)
30686	Insulin isophane porcine 100units/ml suspension for injection 3ml cartridges
8895	Initard 50/50 100unit/ml Injection (Novo Nordisk Ltd)
4247	Insulin isophane porcine 100units/ml suspension for injection 1.5ml cartridges
14933	Hypurin Porcine Isophane 100units/ml suspension for injection 3ml cartridges (Wockhardt UK Ltd)
18224	Humalog 100units/ml solution for injection 10ml vials (Eli Lilly and Company Ltd)
57564	Humalog KwikPen 100units/ml solution for injection 3ml pre-filled pen (Waymade Healthcare Plc)

7318	Humalog 100units/ml solution for injection 3ml cartridges (Eli Lilly and Company Ltd)
57529	Humalog 100units/ml solution for injection 10ml vials (Dowelhurst Ltd)
26060	Insulin lispro 100units/ml solution for injection 10ml vials
5214	Insulin lispro 100units/ml solution for injection 1.5ml cartridges
5250	Insulin biphasic lispro human prb 25:75; 100 units/ml Injection
14313	Insulin lispro 100units/ml solution for injection 3ml cartridges
322	Humalog 100units/ml solution for injection 1.5ml cartridges (Eli Lilly and Company Ltd)
14362	Insulin lispro 100units/ml solution for injection 3ml pre-filled disposable devices
38986	Humalog KwikPen 100units/ml solution for injection 3ml pre-filled pen (Eli Lilly and Company Ltd)
4715	Humalog mix 25 25/75 100units/ml Injection (Eli Lilly and Company Ltd)
27177	Insulin biphasic lispro human prb 50:50; 100 units/ml Injection
10264	Humalog Pen 100units/ml solution for injection 3ml pre-filled pen (Eli Lilly and Company Ltd)
55603	Humalog KwikPen 100units/ml solution for injection 3ml pre-filled pen (DE Pharmaceuticals)
18593	Humalog Mix50 100units/ml suspension for injection 3ml cartridges (Eli Lilly and Company Ltd)
43953	Insulin lispro biphasic 25/75 100units/ml suspension for injection 10ml vials
42395	Humalog Mix25 100units/ml suspension for injection 10ml vials (Eli Lilly and Company Ltd)
36146	Insulin lispro biphasic 50/50 100units/ml suspension for injection 3ml cartridges
57622	Humalog Mix50 KwikPen 100units/ml suspension for injection 3ml pre-filled pen (Waymade Healthcare Plc)
52522	Humalog Mix50 KwikPen 100units/ml suspension for injection 3ml pre-filled pen (DE Pharmaceuticals)
35701	Insulin lispro biphasic 50/50 100units/ml suspension for injection 3ml pre-filled disposable devices
10001	Humalog Mix50 Pen 100units/ml suspension for injection 3ml pre-filled pen (Eli Lilly and Company Ltd)
39086	Humalog Mix50 KwikPen 100units/ml suspension for injection 3ml pre-filled pen (Eli Lilly and Company Ltd)
31258	Insulin lispro biphasic 25/75 100units/ml suspension for injection 3ml pre-filled disposable devices
10243	Humalog Mix25 100units/ml suspension for injection 3ml cartridges (Eli Lilly and Company Ltd)
28185	Insulin lispro biphasic 25/75 100units/ml suspension for injection 3ml cartridges
14270	Humalog Mix25 Pen 100units/ml suspension for injection 3ml pre-filled pen (Eli Lilly and Company Ltd)
39006	Humalog Mix25 KwikPen 100units/ml suspension for injection 3ml pre-filled pen (Eli Lilly and Company Ltd)
14505	Insulin protamine zinc bovine 100units/ml suspension for injection 10ml vials
9503	Hypurin Bovine Protamine Zinc 100units/ml suspension for injection 10ml vials (Wockhardt UK Ltd)
18592	Insulin soluble bovine 100units/ml solution for injection 10ml vials
14938	Insulin soluble bovine cartridge 100unit/ml Solution for injection
14339	Hypurin Bovine Neutral 100units/ml solution for injection 10ml vials (Wockhardt UK Ltd)

23231	Hypurin Bovine Neutral 100units/ml solution for injection 3ml cartridges (Wockhardt UK Ltd)
10572	Insulin soluble bovine 100unit/ml Injection
24593	Neutral insulin bovine 100unit/ml Injection
12297	Hypurin bovine neutral 100unit/ml Injection (C P Pharmaceuticals Ltd)
47360	Neutral insulin 100unit/ml Injection (Celltech Pharma Europe Ltd)
1592	Actrapid penfill 100 100iu/ml Penfill (Novo Nordisk Ltd)
56115	Human Actrapid Penfill 100units/ml solution for injection 1.5ml cartridges (Novo Nordisk Ltd)
22945	Insuman rapid 100iu/ml Injection (Aventis Pharma)
10910	Humaject m2 100iu/ml M2 pen (Eli Lilly and Company Ltd)
1588	Actrapid 100iu/ml Injection (Novo Nordisk Ltd)
4199	Humulin m1 100unit/ml M1 injection (Eli Lilly and Company Ltd)
10915	Humaject m1 100iu/ml M1 pen (Eli Lilly and Company Ltd)
11107	Humulin m4 100unit/ml M4 injection (Eli Lilly and Company Ltd)
10887	Penmix 40/60 100iu/ml Penfill (Novo Nordisk Ltd)
3551	Mixtard 20 penfill 100 100iu/ml Penfill (Novo Nordisk Ltd)
22155	Humaject m5 100iu/ml M5 pen (Eli Lilly and Company Ltd)
11056	Insulin biphasic isophane human pyr 30:70; 100 units/ml Injection
7349	Actrapid 100units/ml solution for injection 10ml vials (Novo Nordisk Ltd)
1649	Human actraphane 100iu/ml Injection (Novo Nordisk Ltd)
22058	Pur-in mix 15/85 Injection (C P Pharmaceuticals Ltd)
14649	Insulin biphasic isophane human pyr 10:90; 100 units/ml Injection
2929	Mixtard 30 100iu/ml GE injection (Novo Nordisk Ltd)
2454	Mixtard 30 penfill 100 100iu/ml Penfill (Novo Nordisk Ltd)
1806	Penmix 30/70 100iu/ml Penfill (Novo Nordisk Ltd)
26403	Pur-in mix 25/75 Injection (C P Pharmaceuticals Ltd)
10484	Penmix 20/80 Penfill (Novo Nordisk Ltd)
3550	Mixtard 40 penfill 100 100iu/ml Penfill (Novo Nordisk Ltd)
54462	Insulin biphasic isophane human emp 25:75; 100 units/ml Injection
4198	Humulin m3 100unit/ml M3 injection (Eli Lilly and Company Ltd)
17809	Humaject m4 100iu/ml M4 pen (Eli Lilly and Company Ltd)
41959	Penject 100unit/ml Injection device (Hypoguard Ltd)
14644	Insulin biphasic isophane human prb 20:80; 100 units/ml Injection
21110	Insulin biphasic isophane human prb 50:50; 100 units/ml Injection
24846	Pur-in neutral 100unit/ml Injection (C P Pharmaceuticals Ltd)
34097	Human initard 50/50 100unit/ml Injection (Novo Nordisk Ltd)
12654	Insulin soluble human prb 100unit/ml Injection
17336	Novopen 100unit/ml Injection device (Novo Nordisk Ltd)
9341	Insulin biphasic isophane human prb 30:70; 100 units/ml Injection
16129	Insulin soluble human 100units/ml solution for injection 3ml cartridges
33167	Insulin biphasic isophane human crb 25:75; 100 units/ml Injection
3439	Penmix 10/90 Pen (Novo Nordisk Ltd)
11055	Insulin biphasic isophane human pyr 20:80; 100 units/ml Injection
1840	Humulin s 100unit/ml Injection (Eli Lilly and Company Ltd)
21347	Penmix 40/60 100iu/ml Injection (Novo Nordisk Ltd)
13837	Insulin biphasic isophane human prb 10:90; 100 units/ml Injection
5255	Mixtard 10 penfill 100 100iu/ml Penfill (Novo Nordisk Ltd)

15199	Insuman comb 25 100iu/ml Injection (Aventis Pharma)
15710	Insulin soluble human emp 100unit/ml Injection
21395	Insulin biphasic isophane human pyr 40:60; 100 units/ml Injection
21374	Insulin biphasic isophane human prb 40:60; 100 units/ml Injection
1594	Actrapid NovoLet 100units/ml solution for injection (Novo Nordisk Ltd)
22983	Insuman Rapid 100units/ml solution for injection 3ml cartridges (Sanofi)
26621	Insulin soluble human crb 100iu/ml Injection
12638	Insulin soluble human pyr 100unit/ml Injection
23993	Insuman Rapid 100units/ml solution for injection 3ml pre-filled OptiSet pen (Sanofi)
4706	Velosulin 100units/ml solution for injection 10ml vials (Novo Nordisk Ltd)
14944	Humulin S 100units/ml solution for injection 3ml cartridges (Eli Lilly and Company Ltd)
27614	Penmix 30/70 100iu/ml Injection (Novo Nordisk Ltd)
27402	Insulin soluble human 100units/ml solution for injection 10ml vials
36430	Insulin soluble human 100units/ml solution for injection 3ml pre-filled disposable devices
56502	Actrapid Penfill 100units/ml solution for injection 3ml cartridges (Novo Nordisk Ltd)
9565	HumaJect S Pen 100units/ml solution for injection (Eli Lilly and Company Ltd)
3396	Penmix 10/90 Penfill (Novo Nordisk Ltd)
21235	Humulin S 100units/ml solution for injection 10ml vials (Eli Lilly and Company Ltd)
2220	Penmix 20/80 Pen (Novo Nordisk Ltd)
29837	Insulin biphasic isophane human prb 25:75; 100 units/ml Injection
1805	Mixtard 30/70 100unit/ml Injection (Novo Nordisk Ltd)
41120	Insulin isophane biphasic human 50/50 100units/ml suspension for injection 3ml pre-filled disposable devices
10277	Humulin M3 100units/ml suspension for injection 3ml cartridges (Eli Lilly and Company Ltd)
2221	Mixtard 30 NovoLet 100units/ml suspension for injection (Novo Nordisk Ltd)
8203	Penmix 50/50 100iu/ml Penfill (Novo Nordisk Ltd)
31205	Insuman Comb 50 100units/ml suspension for injection 3ml pre-filled OptiSet pen (Sanofi)
22697	Insulin isophane biphasic human 50/50 100units/ml suspension for injection 1.5ml cartridges
24002	Insuman Comb 25 100units/ml suspension for injection 5ml vials (Sanofi)
21554	Insuman comb 50 100iu/ml Injection (Aventis Pharma)
60938	Mixtard 30 100units/ml suspension for injection 10ml vials (Waymade Healthcare Plc)
5933	Mixtard 50 NovoLet 100units/ml suspension for injection (Novo Nordisk Ltd)
60933	Humulin M3 100units/ml suspension for injection 10ml vials (Sigma Pharmaceuticals Plc)
50691	Human Mixtard 20 Penfill 100units/ml suspension for injection 1.5ml cartridges (Novo Nordisk Ltd)
16160	Humulin M3 Pen 100units/ml suspension for injection 3ml pre-filled pen (Eli Lilly and Company Ltd)
56857	Insulin isophane biphasic human 15/85 100units/ml suspension for injection 3ml cartridges
17731	Penmix 50/50 100iu/ml Injection (Novo Nordisk Ltd)
2812	Mixtard 40 NovoLet 100units/ml suspension for injection (Novo Nordisk Ltd)
25736	Insulin isophane biphasic human 10/90 100units/ml suspension for injection 3ml cartridges

16152	Insulin isophane biphasic human 30/70 100units/ml suspension for injection 3ml cartridges
52722	Human Mixtard 30 Penfill 100units/ml suspension for injection 1.5ml cartridges (Novo Nordisk Ltd)
12818	Human Mixtard 50 100units/ml suspension for injection 10ml vials (Novo Nordisk Ltd)
21422	Insulin isophane biphasic human 40/60 100units/ml suspension for injection 3ml cartridges
35253	Insuman Comb 50 100units/ml suspension for injection 3ml cartridges (Sanofi)
10245	Mixtard 10 Penfill 100units/ml suspension for injection 3ml cartridges (Novo Nordisk Ltd)
43991	Humulin M3 KwikPen 100units/ml suspension for injection 3ml pre-filled pen (Eli Lilly and Company Ltd)
36194	Insulin isophane biphasic human 25/75 100units/ml suspension for injection 3ml cartridges
21232	Insulin isophane biphasic human 30/70 100units/ml suspension for injection 10ml vials
13277	Mixtard 50 Penfill 100units/ml suspension for injection 3ml cartridges (Novo Nordisk Ltd)
7319	Mixtard 20 Penfill 100units/ml suspension for injection 3ml cartridges (Novo Nordisk Ltd)
25735	Insulin isophane biphasic human 20/80 100units/ml suspension for injection 3ml cartridges
24993	Insuman Comb 25 100units/ml suspension for injection 3ml cartridges (Sanofi)
28096	Insulin isophane biphasic human 50/50 100units/ml suspension for injection 3ml cartridges
44480	Insuman Comb 25 100units/ml suspension for injection 3ml pre-filled SoloStar pen (Sanofi)
7231	Mixtard 30 Penfill 100units/ml suspension for injection 3ml cartridges (Novo Nordisk Ltd)
33232	Insulin isophane biphasic human 50/50 100units/ml suspension for injection 5ml vials
30819	Insuman Comb 15 100units/ml suspension for injection 3ml pre-filled OptiSet pen (Sanofi)
10244	Mixtard 40 Penfill 100units/ml suspension for injection 3ml cartridges (Novo Nordisk Ltd)
45158	Insuman Comb 15 100units/ml suspension for injection 3ml cartridges (Sanofi)
7300	Mixtard 30 100units/ml suspension for injection 10ml vials (Novo Nordisk Ltd)
7793	HumaJect M3 Pen 100units/ml suspension for injection (Eli Lilly and Company Ltd)
8841	Humulin M5 100units/ml suspension for injection 10ml vials (Eli Lilly and Company Ltd)
4093	Humulin M2 100units/ml suspension for injection 3ml cartridges (Eli Lilly and Company Ltd)
25133	Insuman Comb 25 100units/ml suspension for injection 3ml pre-filled OptiSet pen (Sanofi)
5845	Mixtard 30 InnoLet 100units/ml suspension for injection 3ml pre-filled pen (Novo Nordisk Ltd)
2456	Mixtard 10 NovoLet 100units/ml suspension for injection (Novo Nordisk Ltd)
44378	Insulin isophane biphasic human 25/75 100units/ml suspension for injection 3ml pre-filled disposable devices
42954	Insulin isophane biphasic human 25/75 100units/ml suspension for injection 5ml vials

57620	Humulin M3 100units/ml suspension for injection 10ml vials (Mawdsley-Brooks & Company Ltd)
19513	Humulin M3 100units/ml suspension for injection 10ml vials (Eli Lilly and Company Ltd)
2455	Mixtard 20 NovoLet 100units/ml suspension for injection (Novo Nordisk Ltd)
4790	Mixtard 50 penfill 100 100iu/ml Penfill (Novo Nordisk Ltd)
19878	Insulin isophane biphasic human 30/70 100units/ml suspension for injection 3ml pre-filled disposable devices
13622	Hypurin porcine neutral 100unit/ml Injection (C P Pharmaceuticals Ltd)
27396	Insulin soluble porcine 100units/ml solution for injection 10ml vials
26098	Hypurin Porcine Neutral 100units/ml solution for injection 10ml vials (Wockhardt UK Ltd)
14930	Hypurin Porcine Neutral 100units/ml solution for injection 3ml cartridges (Wockhardt UK Ltd)
25479	Insulin soluble porcine 100units/ml solution for injection 3ml cartridges
1842	Pork velosulin 100unit/ml Injection (Novo Nordisk Ltd)
36513	Velosulin cartridge 100unit/ml Injection (Novo Nordisk Ltd)
4129	Insulin soluble porcine 100units/ml solution for injection 1.5ml cartridges
30209	Actrapid mc 100unit/ml Injection (Arun Products Ltd)
9521	Pork Actrapid 100units/ml solution for injection 10ml vials (Novo Nordisk Ltd)
36031	Insulin isophane biphasic porcine 30/70 100units/ml suspension for injection 3ml cartridges
14619	Insulin isophane biphasic porcine 30/70 100units/ml suspension for injection 1.5ml cartridges
20995	Hypurin Porcine 30/70 Mix 100units/ml suspension for injection 3ml cartridges (Wockhardt UK Ltd)
2459	Pork Mixtard 30 100units/ml suspension for injection 10ml vials (Novo Nordisk Ltd)
24800	Hypurin Porcine 30/70 Mix 100units/ml suspension for injection 10ml vials (Wockhardt UK Ltd)
27280	Insulin isophane biphasic porcine 30/70 100units/ml suspension for injection 10ml vials
9618	Hypurin Porcine 30/70 Mix 100units/ml suspension for injection 1.5ml cartridges (C P Pharmaceuticals Ltd)
9376	Insulin zinc suspension crystalline human pyr 100unit/ml long acting Injection
18931	Insulin zinc crystalline human 100units/ml suspension for injection 10ml vials
8322	Insulin zinc suspension mixed human pyr 100unit/ml Injection
7537	Humulin Zn 100units/ml suspension for injection 10ml vials (Eli Lilly and Company Ltd)
1844	Ultratard 100units/ml suspension for injection 10ml vials (Novo Nordisk Ltd)
16682	Tempulin 100unit/ml Injection (Knoll Ltd)
16700	Insulin zinc mixed bovine vial 100unit/ml Sterile suspension injection
12035	Insulin zinc mixed bovine 100units/ml suspension for injection 10ml vials
41834	Insulin zinc suspension lente 100iu/ml Injection (Celltech Pharma Europe Ltd)
17712	Hypurin Bovine Lente 100units/ml suspension for injection 10ml vials (Wockhardt UK Ltd)
10547	Humulin Lente 100units/ml suspension for injection 10ml vials (Eli Lilly and Company Ltd)
1587	Monotard 100units/ml suspension for injection 10ml vials (Novo Nordisk Ltd)
18461	Insulin zinc mixed human 100units/ml suspension for injection 10ml vials
44251	Insulin zinc suspension mixed porcine 100unit/ml Injection
34031	Monotard mc 100unit/ml Injection (Novo Nordisk Ltd)

26498	Insulin zinc suspension mixed bovine and porcine 100unit/ml Injection
4784	Lentard mc 100unit/ml Injection (Novo Nordisk Ltd)
12299	Semitard mc 100unit/ml Injection (Novo Nordisk Ltd)
34713	Insulin 1 ml syringe
2321	Insulin 0.5ml disposable syringe
23636	Insulin 1ml disposable syringe
40085	Insulin 2ml syringe
22060	Insulin 0.5 0.5ml Syringe
47588	Insulin 1ml click count
38093	Insulin 1ml pre-set syringe
16959	Hypodermic U100 insulin syringe sterile single use / single patient use 1ml with 12mm needle 0.3mm/30gauge
2808	INSULIN LENTARD INJ
9363	U100 Insulin syringe 0.5ml
30861	INSULIN ZINC HUMAN SUSPENSION
24485	INSULIN ZINC ANIMAL SUSPENSION
45045	NovoTwist hypodermic insulin needles for pre-filled / reusable pen injectors screw on 5mm/32gauge (Novo Nordisk Ltd)
56656	Kendall Magellan hypodermic U100 insulin syringe sterile single use / single patient use 0.3ml with 8mm safety needle 0.3mm/30gauge (Covidien (UK) Commercial Ltd)
54629	Insupen hypodermic insulin needles for pre-filled / reusable pen injectors screw on 8mm/31gauge (Spirit Healthcare Ltd)
6554	Autopen 24 hypodermic insulin injection pen reusable for 3ml cartridge 1 unit dial up / range 1-21 units (Owen Mumford Ltd)
43568	ClikSTAR hypodermic insulin injection pen reusable for 3ml cartridge 1 unit dial up / range 1-80 units Silver (Sanofi)
15040	INSULIN MONOPHANE (ISOPHANE) 100 I/U INJ
35454	Comfort Point hypodermic insulin needles for pre-filled / reusable pen injectors screw on 8mm/31gauge (Disposable Medical Equipment Ltd)
48633	Comfort Point hypodermic insulin needles for pre-filled / reusable pen injectors screw on 4mm/31gauge (Disposable Medical Equipment Ltd)
54573	Hypodermic insulin injection pen reusable for 3ml cartridge 1 unit dial up / range 1-21 units
55687	Insulin degludec 100units/ml solution for injection 3ml pre-filled disposable devices
43833	ClikSTAR hypodermic insulin injection pen reusable for 3ml cartridge 1 unit dial up / range 1-80 units Blue (Sanofi)
8483	MONOJECT INSULIN NEEDLES
52232	Hypodermic insulin injection pen reusable for 3ml cartridge 0.5 unit dial up / range 1-30 units
33356	Hypodermic insulin needles for pre-filled / reusable pen injectors screw on 12mm/29gauge
49479	Hypodermic insulin needles for pre-filled / reusable pen injectors screw on 4mm/31gauge
12840	B-d u-100 0.5ml Insulin syringe (Becton, Dickinson UK Ltd)
56691	Insulin degludec 200units/ml solution for injection 3ml pre-filled disposable devices
55627	Insupen hypodermic insulin needles for pre-filled / reusable pen injectors screw on 5mm/31gauge (Spirit Healthcare Ltd)

12892	Clinipak u100 single use ins syr+12mm need28g 0.5ml [rand] 0.5ml Insulin syringe with 28gauge needle 12mm (Rand Rocket Ltd)
31267	INSULIN PUR-IN MIX 50/50 100 I/U INJ
24845	INSULIN PUR-IN ISOPHANE 100 I/U INJ
26795	SYRINGE INSULIN DISPOSABLE
11521	Hypodermic U100 insulin syringe sterile single use / single patient use 1ml with 12mm needle 0.4mm/27gauge
5967	Hypodermic U100 insulin syringe sterile single use / single patient use 0.5ml with not less than 12mm needle 0.33mm/29gauge
14504	INSULIN HYPURIN PROTAMINE ZINC 100 I/U INJ
14642	Bd m 0.3ml Insulin syringe with 29gauge needle 12mm (Becton, Dickinson UK Ltd)
40555	HumaPen Memoir hypodermic insulin injection pen reusable for 3ml cartridge 1 unit dial up / range 1-60 units (Eli Lilly and Company Ltd)
58745	GlucoRx FinePoint hypodermic insulin needles for pre-filled / reusable pen injectors screw on 4mm/31gauge (GlucoRx Ltd)
11271	B-d u-100 1ml Insulin syringe (Becton, Dickinson UK Ltd)
6233	Mylife Clickfine hypodermic insulin needles for pre-filled / reusable pen injectors snap on 10mm/29gauge (Ypsomed Ltd)
58579	Hypodermic insulin needles for pre-filled / reusable pen injectors screw on 4mm/32.5gauge
8354	INSULIN ISOPHANE 70%/NEUTRAL 30% 100 I/U INJ
56879	HumaPen Savvio hypodermic insulin injection pen reusable for 3ml cartridge 1 unit dial up / range 1-60 units Graphite (Eli Lilly and Company Ltd)
10566	INSULIN HUMULIN M CARTRIDGE 100 I/U
13550	INSULIN BP 100 I/U
5345	Monoject hypodermic U100 insulin syringe sterile single use / single patient use 0.5ml with 12mm needle 0.36mm/28gauge (Covidien (UK) Commercial Ltd)
5121	BD Micro-Fine + hypodermic insulin needles for pre-filled / reusable pen injectors screw on 12.7mm/29gauge (Becton, Dickinson UK Ltd)
7763	INSULIN NEUPHANE (ISOPHANE)(PURIFIED) 100 I/U INJ
8646	INSULIN ZINC CRYSTALLINE susp 100 I/U INJ
1591	U100 Insulin syringe 1ml
13036	Unifine hypodermic U100 insulin syringe sterile single use / single patient use 0.5ml with 12mm needle 0.33mm/29gauge (Owen Mumford Ltd)
24722	INSULIN ISOPHANE 50%/NEUTRAL 50% 100 I/U INJ
21459	SYRINGE INSULIN U100 S/U+8MM NEEDLE
27151	SYRINGE INSULIN U100 S/U+8MM NEEDLE
50798	Hypodermic insulin injection pen reusable for 3ml cartridge 0.5 unit dial up / range 1-35 units
48342	HumaPen Luxura hypodermic insulin injection pen reusable for 3ml cartridge 1 unit dial up / range 1-60 units Burgundy (Eli Lilly and Company Ltd)
61562	Hypodermic insulin needles for pre-filled / reusable pen injectors screw on 8mm/29gauge
16866	Unifine hypodermic U100 insulin syringe sterile single use / single patient use 0.3ml with 12mm needle 0.33mm/29gauge (Owen Mumford Ltd)
9834	Mylife Clickfine hypodermic insulin needles for pre-filled / reusable pen injectors snap on 12mm/29gauge (Ypsomed Ltd)

51650	Omnican Fine hypodermic insulin needles for pre-filled / reusable pen injectors screw on 6mm/31gauge (B.Braun Medical Ltd)
12300	SYRINGE INSULIN (BS1619/1) 2ML
37427	HumaPen Luxura HD hypodermic insulin injection pen reusable for 3ml cartridge 0.5 unit dial up / range 1-30 units (Eli Lilly and Company Ltd)
39150	Hypodermic U100 insulin syringe sterile single use / single patient use 1ml with 12mm safety needle 0.33mm/29gauge
5634	Hypodermic U100 insulin syringe sterile single use / single patient use 1ml with 12mm needle 0.36mm/28gauge
19707	INSULIN HUMULIN S (NEUTRAL SOLUBLE)
7412	U100 Insulin syringe 0.5ml
18446	Unifine hypodermic U100 insulin syringe sterile single use / single patient use 1ml with 12mm needle 0.33mm/29gauge (Owen Mumford Ltd)
7959	INSULIN MIXTARD 30/70 40 I/U INJ
18149	Monoject u100 insulin syringe 12mm(29g)0.5ml 12mm29G 0.5ml Insulin syringe (Covidien (UK) Commercial Ltd)
10133	U100 Insulin syringe 1ml
22823	INSULIN ISOPHANE (PURIFIED) 100 I/U INJ
60028	Hypodermic insulin needles for pre-filled / reusable pen injectors snap on 4mm/32gauge
11346	B-d u-100 0.3ml Insulin syringe (Becton, Dickinson UK Ltd)
14506	INSULIN BOVINE PROTAMINE ZINC 100 I/U INJ
13009	Hypodermic U100 insulin syringe sterile single use / single patient use 0.5ml with 12mm needle 0.36mm/28gauge
56352	HumaPen Savvio hypodermic insulin injection pen reusable for 3ml cartridge 1 unit dial up / range 1-60 units Green (Eli Lilly and Company Ltd)
44810	Hypodermic insulin needles for pre-filled / reusable pen injectors screw on 5mm/32gauge
19029	SYRINGE PRE-SET INSULIN FOR BLIND 2ML
38774	NovoPen 4 hypodermic insulin injection pen reusable for 3ml cartridge 1 unit dial up / range 1-60 units Blue (Novo Nordisk Ltd)
10258	Hypodermic insulin needles for pre-filled / reusable pen injectors screw on 8mm/31gauge
21945	INSULIN PORK INSULATARD
56808	Omnican Fine hypodermic insulin needles for pre-filled / reusable pen injectors screw on 12mm/29gauge (B.Braun Medical Ltd)
43670	Hypodermic insulin needles for pre-filled / reusable pen injectors screw on 4mm/32gauge
6991	BD Micro-Fine + hypodermic U100 insulin syringe sterile single use / single patient use 0.5ml with 12.7mm needle 0.33mm/29gauge (Becton, Dickinson UK Ltd)
5649	Autopen hypodermic insulin injection pen reusable for 1.5ml cartridge 1 unit dial up / range 1-16 units (Owen Mumford Ltd)
33101	Hypodermic insulin needles for pre-filled / reusable pen injectors screw on 12.7mm/29gauge
14887	Hypodermic insulin needles for pre-filled / reusable pen injectors screw on 5mm/31gauge
1751	Hypodermic U100 insulin syringe glass reusable 1ml
8839	INSULIN SEMITARD 100 I/U INJ

28978	INSULIN PUR-IN MIX 15/85 100 I/U INJ
19977	Omnican 50 hypodermic U100 insulin syringe sterile single use / single patient use 0.5ml with 12mm needle 0.3mm/30gauge (B.Braun Medical Ltd)
51612	Hypodermic insulin needles for pre-filled / reusable pen injectors screw on 5mm/29gauge
35218	Optipen pro 1 yellow insulin pen 3ml/1-60 units 3ml/1-60 units Insulin pen (Aventis Pharma)
60609	Insupen hypodermic insulin needles for pre-filled / reusable pen injectors screw on 6mm/32gauge (Spirit Healthcare Ltd)
58801	GlucoRx FinePoint hypodermic insulin needles for pre-filled / reusable pen injectors screw on 10mm/29gauge (GlucoRx Ltd)
20195	INSULIN BOVINE PROTAMINE ZINC 40 I/U INJ
6009	Myjector hypodermic U100 insulin syringe sterile single use / single patient use 0.5ml with 12mm needle 0.4mm/27gauge (Terumo UK Ltd)
6091	NovoPen 3 Fun hypodermic insulin injection pen reusable for 3ml cartridge 1 unit dial up / range 2-70 units Red (Novo Nordisk Ltd)
35057	OptiClik hypodermic insulin injection pen reusable for 3ml cartridge 1 unit dial up / range 1-80 units Blue (Sanofi)
5059	NovoPen 3 Classic hypodermic insulin injection pen reusable for 3ml cartridge 1 unit dial up / range 2-70 units (Novo Nordisk Ltd)
51881	Omnican Fine hypodermic insulin needles for pre-filled / reusable pen injectors screw on 8mm/31gauge (B.Braun Medical Ltd)
10145	Humapen luxura insulin pen 3ml/1-60 units Insulin pen 3ml/1-60 units (Eli Lilly and Company Ltd)
28723	INSULIN ZINC BOVINE SUSPENSION
6470	Autopen 24 hypodermic insulin injection pen reusable for 3ml cartridge 2 unit dial up / range 2-42 units (Owen Mumford Ltd)
13474	U100 Insulin syringe Sp.36 [2A] 1ml
60967	Nanopass hypodermic insulin needles for pre-filled / reusable pen injectors screw on 8mm/32.5gauge (Terumo UK Ltd)
48576	BD AutoShield Duo hypodermic insulin needles for pre-filled / reusable pen injectors screw on 5mm/30gauge (Becton, Dickinson UK Ltd)
23003	INSULIN ISOPHANE (NPH) 40 I/U
14646	Hypodermic U100 insulin syringe sterile single use / single patient use 0.3ml with 12mm needle 0.33mm/29gauge
19829	INSULIN NOVO MONOTARD MC
48501	HumaPen Luxura hypodermic insulin injection pen reusable for 3ml cartridge 1 unit dial up / range 1-60 units Champagne (Eli Lilly and Company Ltd)
12060	INSULIN QUICKSOL (SOLUBLE NEUTRAL) 100 I/U INJ
7861	INSULIN HUMULIN S (NEUTRAL) CARTRIDGE 100 I/U
5267	BD Micro-Fine + hypodermic U100 insulin syringe sterile single use / single patient use 0.5ml with 8mm needle 0.3mm/30gauge (Becton, Dickinson UK Ltd)
5022	NovoFine hypodermic insulin needles for pre-filled / reusable pen injectors screw on 12mm/28gauge (Novo Nordisk Ltd)
62180	Insulin aspart 100units/ml solution for injection 1.6ml cartridges
57387	Kendall Magellan hypodermic U100 insulin syringe sterile single use / single patient use 0.3ml with 12mm safety needle 0.33mm/29gauge (Covidien (UK) Commercial Ltd)

10242	Hypodermic insulin needles for pre-filled / reusable pen injectors screw on 6mm/31gauge
27911	INSULIN HUMAN ACTRAPID PENFILL
25006	INSULIN HUMAN ACTRAPID (NEUTRAL)
58817	GlucoRx FinePoint hypodermic insulin needles for pre-filled / reusable pen injectors screw on 12mm/29gauge (GlucoRx Ltd)
6753	Novopen Junior hypodermic insulin injection pen reusable for 3ml cartridge 0.5 unit dial up / range 1-35 units Green (Novo Nordisk Ltd)
21223	U100 Insulin syringe 0.3ml
35017	Optipen pro 1 white insulin pen 3ml/1-60 units 3ml/1-60 units Insulin pen (Aventis Pharma)
22946	Omnican 100 hypodermic U100 insulin syringe sterile single use / single patient use 1ml with 12mm needle 0.3mm/30gauge (B.Braun Medical Ltd)
49451	Mylife Clickfine AutoProtect hypodermic insulin needles for pre-filled / reusable pen injectors screw on 8mm/29gauge (Ypsomed Ltd)
57451	Microdot Droplet hypodermic insulin needles for pre-filled / reusable pen injectors screw on 8mm/31gauge (Cambridge Sensors Ltd)
59846	Mylife Clickfine hypodermic insulin needles for pre-filled / reusable pen injectors snap on 4mm/32gauge (Ypsomed Ltd)
48811	Unifine Pentips Plus hypodermic insulin needles for pre-filled / reusable pen injectors screw on 6mm/31gauge (Owen Mumford Ltd)
7757	INSULIN NEULENTE (ZINC SUSP)(PURIFIED) 100 I/U INJ
7062	Myjector hypodermic U100 insulin syringe sterile single use / single patient use 1ml with 12mm needle 0.4mm/27gauge (Terumo UK Ltd)
5850	Mylife Clickfine hypodermic insulin needles for pre-filled / reusable pen injectors snap on 6mm/31gauge (Ypsomed Ltd)
55521	Insupen hypodermic insulin needles for pre-filled / reusable pen injectors screw on 4mm/32gauge (Spirit Healthcare Ltd)
16209	INSULIN HYPURIN SOLUBLE 100 I/U INJ
8838	INSULIN SEMITARD 40 I/U INJ
5789	Hypodermic U100 insulin syringe sterile single use / single patient use 0.5ml with 8mm needle 0.3mm/30gauge
11245	B-d u-100 0.3ml Insulin syringe (Becton, Dickinson UK Ltd)
7783	INSULIN ISOPHANE (HUMAN) 100 I/U INJ
60951	Insulin human 100units/ml solution for injection 10ml vials
5164	NovoPen 3 Demi hypodermic insulin injection pen reusable for 3ml cartridge 0.5 unit dial up / range 1-35 units (Novo Nordisk Ltd)
321	INSULIN HUMAN ACTRAPID (NEUTRAL) 40 I/U INJ
57243	Omnican Fine hypodermic insulin needles for pre-filled / reusable pen injectors screw on 10mm/30gauge (B.Braun Medical Ltd)
6060	Unifine Pentips hypodermic insulin needles for pre-filled / reusable pen injectors screw on 6mm/30gauge (Owen Mumford Ltd)
4896	Monoject hypodermic U100 insulin syringe sterile single use / single patient use 1ml with 12mm needle 0.36mm/28gauge (Covidien (UK) Commercial Ltd)
22161	INSULIN HUMULIN M1 VIAL
5769	Hypodermic U100 insulin syringe sterile single use / single patient use 0.5ml with 12mm needle 0.4mm/27gauge

54028	Unifine Pentips Plus hypodermic insulin needles for pre-filled / reusable pen injectors screw on 12mm/29gauge (Owen Mumford Ltd)
59005	Mylife Penfine Classic hypodermic insulin needles for pre-filled / reusable pen injectors screw on 8mm/31gauge (Ypsomed Ltd)
8376	INSULIN ISOPHANE 100 I/U
58961	Mylife Penfine Classic hypodermic insulin needles for pre-filled / reusable pen injectors screw on 4mm/32gauge (Ypsomed Ltd)
23437	Hypodermic insulin needles for pre-filled / reusable pen injectors screw on 12mm/28gauge
18208	Hypodermic insulin needles for pre-filled / reusable pen injectors snap on 10mm/29gauge
22987	Hypodermic insulin needles for pre-filled / reusable pen injectors snap on 12mm/29gauge
59004	Mylife Penfine Classic hypodermic insulin needles for pre-filled / reusable pen injectors screw on 6mm/32gauge (Ypsomed Ltd)
60750	Hypodermic insulin needles for pre-filled / reusable pen injectors screw on 4mm/33gauge
11878	Hypodermic U100 insulin syringe sterile single use / single patient use 1ml with 8mm needle 0.3mm/30gauge
35081	OptiClik hypodermic insulin injection pen reusable for 3ml cartridge 1 unit dial up / range 1-80 units Grey (Sanofi)
49052	Unifine Pentips Plus hypodermic insulin needles for pre-filled / reusable pen injectors screw on 8mm/31gauge (Owen Mumford Ltd)
48765	Unifine Pentips hypodermic insulin needles for pre-filled / reusable pen injectors screw on 5mm/31gauge (Owen Mumford Ltd)
54885	Hypodermic insulin needles for pre-filled / reusable pen injectors screw on 8mm/32gauge
18195	Hypodermic insulin needles for pre-filled / reusable pen injectors snap on 6mm/31gauge
20672	INSULIN HUM/ACTRAPID
51107	Hypodermic insulin injection pen reusable for 3ml cartridge 2 unit dial up / range 2-42 units
47751	TS-A Safety hypodermic U100 insulin syringe sterile single use / single patient use 1ml with 12mm safety needle 0.33mm/29gauge (Minsmed)
48435	BD AutoShield hypodermic insulin needles for pre-filled / reusable pen injectors screw on 5mm/29gauge (Becton, Dickinson UK Ltd)
5421	NovoFine hypodermic insulin needles for pre-filled / reusable pen injectors screw on 6mm/31gauge (Novo Nordisk Ltd)
11408	Autopen Classic hypodermic insulin injection pen reusable for 3ml cartridge 1 unit dial up / range 1-21 units (Owen Mumford Ltd)
59133	BD SafetyGlide hypodermic U100 insulin syringe sterile single use / single patient use 0.5ml with 8mm safety needle 0.3mm/30gauge (Becton, Dickinson UK Ltd)
58798	GlucoRx FinePoint hypodermic insulin needles for pre-filled / reusable pen injectors screw on 6mm/31gauge (GlucoRx Ltd)
6724	U100 Insulin syringe 0.3ml
9578	Unifine Pentips hypodermic insulin needles for pre-filled / reusable pen injectors screw on 12mm/29gauge (Owen Mumford Ltd)
26784	INSULIN ZINC SEMILENTE SUSP BP 100 I/U INJ

5557	NovoPen 3 Fun hypodermic insulin injection pen reusable for 3ml cartridge 1 unit dial up / range 2-70 units Blue (Novo Nordisk Ltd)
11086	B-d u-100 0.5ml Insulin syringe (Becton, Dickinson UK Ltd)
25422	Hypoguard u100 click/count 1ml Insulin syringe (Hypoguard Ltd)
14191	Autopen Junior hypodermic insulin injection pen reusable for 3ml cartridge 2 unit dial up / range 2-42 units (Owen Mumford Ltd)
30918	ABCare hypodermic U100 insulin syringe glass reusable 0.5ml (Rand Rocket Ltd)
35143	NovoFine Autocover hypodermic insulin needles for pre-filled / reusable pen injectors screw on 8mm/30gauge (Novo Nordisk Ltd)
24866	INSULIN INSULATARD (LEO RETARD) 40 I/U INJ
6138	HumaPen Ergo hypodermic insulin injection pen reusable for 3ml cartridge 1 unit dial up / range 1-60 units Burgundy (Eli Lilly and Company Ltd)
7764	INSULIN NEUSULIN (NEUTRAL)(PURIFIED) 100 I/U INJ
15624	INSULIN ISOPHANE (HIGHLY PURIFIED) 100 I/U INJ
49172	NovoPen Echo hypodermic insulin injection pen reusable for 3ml cartridge 0.5 unit dial up / range 0.5-30 units Red (Novo Nordisk Ltd)
1645	INSULIN NOVO ACTRAPID MC 100 I/U INJ
11345	Bd Ultra Pen 3ml Insulin pen (Becton, Dickinson UK Ltd)
58754	BD SafetyGlide hypodermic U100 insulin syringe sterile single use / single patient use 1ml with 12mm safety needle 0.33mm/29gauge (Becton, Dickinson UK Ltd)
31699	ABCare hypodermic U100 insulin syringe glass reusable 1ml (Rand Rocket Ltd)
56639	HumaPen Savvio hypodermic insulin injection pen reusable for 3ml cartridge 1 unit dial up / range 1-60 units Blue (Eli Lilly and Company Ltd)
5142	BD Micro-Fine + hypodermic insulin needles for pre-filled / reusable pen injectors screw on 8mm/31gauge (Becton, Dickinson UK Ltd)
48771	Unifine Pentips Plus hypodermic insulin needles for pre-filled / reusable pen injectors screw on 5mm/31gauge (Owen Mumford Ltd)
4248	INSULIN NOVO ULTRATARD MC 100 I/U INJ
6981	Novopen Junior hypodermic insulin injection pen reusable for 3ml cartridge 0.5 unit dial up / range 1-35 units Yellow (Novo Nordisk Ltd)
58878	Hypodermic insulin needles for pre-filled / reusable pen injectors screw on 10mm/29gauge
6781	Autopen Classic hypodermic insulin injection pen reusable for 3ml cartridge 2 unit dial up / range 2-42 units (Owen Mumford Ltd)
7164	Unifine Pentips hypodermic insulin needles for pre-filled / reusable pen injectors screw on 6mm/31gauge (Owen Mumford Ltd)
58449	Unifine Pentips hypodermic insulin needles for pre-filled / reusable pen injectors screw on 4mm/32gauge (Owen Mumford Ltd)
56642	Microdot Droplet hypodermic insulin needles for pre-filled / reusable pen injectors screw on 6mm/31gauge (Cambridge Sensors Ltd)
38808	NovoPen 4 hypodermic insulin injection pen reusable for 3ml cartridge 1 unit dial up / range 1-60 units Silver (Novo Nordisk Ltd)
22328	Unifine single use Insulin syringe with 30gauge needle 8mm 0.5ml (Owen Mumford Ltd)
15294	Innovo hypodermic insulin injection pen reusable for 3ml cartridge 1 unit dial up / range 1-70 units Green (Novo Nordisk Ltd)
25786	Comfort Point hypodermic insulin needles for pre-filled / reusable pen injectors screw on 6mm/31gauge (Disposable Medical Equipment Ltd)
1839	INSULIN HUMULIN I (ISOPHANE) 100 I/U INJ

18301	INSULIN SOLUBLE INJ I/U^2
13108	Autopen Special Edition hypodermic insulin injection pen reusable for 3ml cartridge 1 unit dial up / range 1-21 units (Owen Mumford Ltd)
58578	Kendall Magellan hypodermic U100 insulin syringe sterile single use / single patient use 0.5ml with 12mm safety needle 0.33mm/29gauge (Covidien (UK) Commercial Ltd)
32053	INSULIN HUMALOG MIX 25
13969	U100 Insulin syringe 0.3ml
1643	INSULIN NOVO MONOTARD MC 100 I/U INJ
57388	Kendall Magellan hypodermic U100 insulin syringe sterile single use / single patient use 1ml with 8mm safety needle 0.3mm/30gauge (Covidien (UK) Commercial Ltd)
56983	Insupen hypodermic insulin needles for pre-filled / reusable pen injectors screw on 6mm/31gauge (Spirit Healthcare Ltd)
31466	Exubera insulin release unit (Pfizer Ltd)
55746	Insupen hypodermic insulin needles for pre-filled / reusable pen injectors screw on 8mm/32gauge (Spirit Healthcare Ltd)
7203	Unifine Pentips hypodermic insulin needles for pre-filled / reusable pen injectors screw on 8mm/31gauge (Owen Mumford Ltd)
9702	BD Micro-Fine + hypodermic U100 insulin syringe sterile single use / single patient use 1ml with 12.7mm needle 0.33mm/29gauge (Becton, Dickinson UK Ltd)
22496	INSULIN ZINC LENTE PURIFIED SUSPENSION
16389	Myjector hypodermic U100 insulin syringe sterile single use / single patient use 1ml with 12mm needle 0.33mm/29gauge (Terumo UK Ltd)
22974	Hypodermic insulin needles for pre-filled / reusable pen injectors snap on 8mm/31gauge
3076	NovoFine hypodermic insulin needles for pre-filled / reusable pen injectors screw on 6mm/30gauge (Novo Nordisk Ltd)
20671	INSULIN HUM/ACTRAPHANE
58995	BD SafetyGlide hypodermic U100 insulin syringe sterile single use / single patient use 0.5ml with 12mm safety needle 0.33mm/29gauge (Becton, Dickinson UK Ltd)
59243	BD SafetyGlide hypodermic U100 insulin syringe sterile single use / single patient use 0.3ml with 8mm safety needle 0.25mm/31gauge (Becton, Dickinson UK Ltd)
30305	Hypodermic U100 insulin syringe sterile single use / single patient use 0.5ml with 12mm needle 0.3mm/30gauge
53437	Hypodermic insulin needles for pre-filled / reusable pen injectors screw on 5mm/30gauge
60621	Hypodermic insulin injection pen reusable for 3ml cartridge 0.5 unit dial up / range 0.5-30 units
58884	GlucoRx FinePoint hypodermic insulin needles for pre-filled / reusable pen injectors screw on 8mm/31gauge (GlucoRx Ltd)
52319	Hypodermic insulin injection pen reusable for 3ml cartridge 1 unit dial up / range 1-80 units
57153	Kendall Magellan hypodermic U100 insulin syringe sterile single use / single patient use 0.5ml with 8mm safety needle 0.3mm/30gauge (Covidien (UK) Commercial Ltd)
18645	INSULIN NEUTRAL (PURIFIED) 100 I/U INJ
54027	Hypodermic insulin needles for pre-filled / reusable pen injectors snap on 4.5mm/31gauge
22806	INSULIN PORK ACTRAPID
22094	INSULIN HUMULIN M2 VIAL

56785	HumaPen Savvio hypodermic insulin injection pen reusable for 3ml cartridge 1 unit dial up / range 1-60 units Red (Eli Lilly and Company Ltd)
62098	Hypodermic insulin needles for pre-filled / reusable pen injectors screw on 8mm/32.5gauge
5015	NovoFine hypodermic insulin needles for pre-filled / reusable pen injectors screw on 8mm/30gauge (Novo Nordisk Ltd)
6730	Hypodermic U100 insulin syringe sterile single use / single patient use 1ml with not less than 12mm needle 0.33mm/29gauge
59311	Kendall Magellan hypodermic U100 insulin syringe sterile single use / single patient use 1ml with 12mm safety needle 0.33mm/29gauge (Covidien (UK) Commercial Ltd)
57493	HumaPen Savvio hypodermic insulin injection pen reusable for 3ml cartridge 1 unit dial up / range 1-60 units Silver (Eli Lilly and Company Ltd)
15951	Autopen Junior hypodermic insulin injection pen reusable for 3ml cartridge 1 unit dial up / range 1-21 units (Owen Mumford Ltd)
37055	Comfort Point hypodermic U100 insulin syringe sterile single use / single patient use 0.5ml with 12mm needle 0.33mm/29gauge (Disposable Medical Equipment Ltd)
57744	Omnican Fine hypodermic insulin needles for pre-filled / reusable pen injectors screw on 4mm/31gauge (B.Braun Medical Ltd)
7127	BD Micro-Fine + hypodermic U100 insulin syringe sterile single use / single patient use 1ml with 8mm needle 0.3mm/30gauge (Becton, Dickinson UK Ltd)
61780	Insupen hypodermic insulin needles for pre-filled / reusable pen injectors screw on 12mm/29gauge (Spirit Healthcare Ltd)
35078	Optipen pro 1 blue insulin pen 3ml/1-60 units 3ml/1-60 units Insulin pen (Aventis Pharma)
17643	Autopen Special Edition hypodermic insulin injection pen reusable for 3ml cartridge 2 unit dial up / range 2-42 units (Owen Mumford Ltd)
15895	Innovo hypodermic insulin injection pen reusable for 3ml cartridge 1 unit dial up / range 1-70 units Orange (Novo Nordisk Ltd)
48829	NovoPen Echo hypodermic insulin injection pen reusable for 3ml cartridge 0.5 unit dial up / range 0.5-30 units Blue (Novo Nordisk Ltd)
36959	Comfort Point hypodermic insulin needles for pre-filled / reusable pen injectors screw on 12mm/29gauge (Disposable Medical Equipment Ltd)
53148	Hypodermic insulin injection pen reusable for 3ml cartridge 1 unit dial up / range 1-60 units
1589	U100 Insulin syringe 0.5ml
10545	INSULIN HUMULIN M4 CARTRIDGE 100 I/U
45639	IME-FINE hypodermic insulin needles for pre-filled / reusable pen injectors screw on 6mm/31gauge (Arctic Medical Ltd)
5742	Unifine Pentips hypodermic insulin needles for pre-filled / reusable pen injectors screw on 8mm/30gauge (Owen Mumford Ltd)
49307	Mylife Clickfine hypodermic insulin needles for pre-filled / reusable pen injectors snap on 4.5mm/31gauge (Ypsomed Ltd)
62562	Insupen hypodermic insulin needles for pre-filled / reusable pen injectors screw on 4mm/33gauge (Spirit Healthcare Ltd)
10546	INSULIN HUMULIN M4 100 I/U INJ
54886	Hypodermic insulin needles for pre-filled / reusable pen injectors screw on 6mm/32gauge

56939	HumaPen Savvio hypodermic insulin injection pen reusable for 3ml cartridge 1 unit dial up / range 1-60 units Pink (Eli Lilly and Company Ltd)
6228	BD Micro-Fine + hypodermic U100 insulin syringe sterile single use / single patient use 0.3ml with 8mm needle 0.3mm/30gauge (Becton, Dickinson UK Ltd)
12244	INSULIN ZINC BOVINE susp 100 I/U INJ
13274	Clinipak u100 single use ins syr+12mm need28g 1ml [rand] 1ml Insulin syringe with 28gauge needle 12mm (Rand Rocket Ltd)
56624	Kendall Magellan hypodermic U100 insulin syringe sterile single use / single patient use 0.5ml with 12mm safety needle 0.3mm/30gauge (Covidien (UK) Commercial Ltd)
9619	Bd Ultra Pen 1.5ml Insulin pen (Becton, Dickinson UK Ltd)
5966	Mylife Clickfine hypodermic insulin needles for pre-filled / reusable pen injectors snap on 8mm/31gauge (Ypsomed Ltd)
2373	INSULIN HUMAN VELOSULIN 100 I/U INJ
43489	BD Micro-Fine Ultra hypodermic insulin needles for pre-filled / reusable pen injectors screw on 4mm/32gauge (Becton, Dickinson UK Ltd)
59475	Nanopass hypodermic insulin needles for pre-filled / reusable pen injectors screw on 4mm/32.5gauge (Terumo UK Ltd)
59793	Microdot Droplet hypodermic insulin needles for pre-filled / reusable pen injectors screw on 4mm/32gauge (Cambridge Sensors Ltd)
17076	Omnican 100 hypodermic U100 insulin syringe sterile single use / single patient use 1ml with 8mm needle 0.3mm/30gauge (B.Braun Medical Ltd)
9079	INSULIN SOLUBLE 100 I/U INJ
5620	U100 Insulin syringe 0.3ml
38236	Comfort Point hypodermic U100 insulin syringe sterile single use / single patient use 1ml with 12mm needle 0.33mm/29gauge (Disposable Medical Equipment Ltd)
20196	INSULIN SOLUBLE 40 I/U INJ
7765	INSULIN NEUTRAL (HUMAN) 100 I/U INJ
58581	GlucoRx FinePoint hypodermic insulin needles for pre-filled / reusable pen injectors screw on 5mm/31gauge (GlucoRx Ltd)
10691	INSULIN ISOPHANE (NPH) 100 I/U INJ
5873	BD Micro-Fine + hypodermic insulin needles for pre-filled / reusable pen injectors screw on 5mm/31gauge (Becton, Dickinson UK Ltd)
24554	Unifine single use Insulin syringe with 30gauge needle 8mm 0.3ml (Owen Mumford Ltd)
28666	Omnican 50 hypodermic U100 insulin syringe sterile single use / single patient use 0.5ml with 8mm needle 0.3mm/30gauge (B.Braun Medical Ltd)
19271	Hypodermic insulin needles for pre-filled / reusable pen injectors screw on 8mm/30gauge
6831	HumaPen Ergo hypodermic insulin injection pen reusable for 3ml cartridge 1 unit dial up / range 1-60 units Teal (Eli Lilly and Company Ltd)
6378	Hypodermic U100 insulin syringe sterile single use / single patient use 0.3ml with 8mm needle 0.3mm/30gauge
13096	Autopen hypodermic insulin injection pen reusable for 1.5ml cartridge 2 unit dial up / range 2-32 units (Owen Mumford Ltd)
7075	Optipen Pro 1 hypodermic insulin injection pen reusable for 3ml cartridge 1 unit dial up / range 1-60 units (Sanofi)
42797	Injex ampoule pack 60501 (Ocon Chemicals Ltd)
60626	InsuJet starter set 012007GBSP Grey (Spirit Healthcare Ltd)
42305	Injex 10ml vial adaptor pack 60502 (Ocon Chemicals Ltd)

51836	InsuJet 3ml cartridge adaptor pack 012071GB1503 (Spirit Healthcare Ltd)
26338	Injex starter set 60500 (Ocon Chemicals Ltd)
61845	NovoRapid PumpCart 100units/ml solution for injection 1.6ml cartridges (Novo Nordisk Ltd)
51182	InsuJet starter set 012003GBSP Lime (Spirit Healthcare Ltd)
44601	Injex 4 monthly refill pack 60504 (Ocon Chemicals Ltd)
49509	InsuJet starter set 012005GBSP Blue (Spirit Healthcare Ltd)
49508	InsuJet 10ml vial adaptor pack 01207GB1510 (Spirit Healthcare Ltd)
49507	InsuJet nozzle pack 012070GB15 (Spirit Healthcare Ltd)

Product code list: insulin treatments

Product Code	Product Name
4163	Rapitard MC 100unit/ml Injection (Novo Nordisk Ltd)
13416	Insulin biphasic 100 units/ml Injection
16142	Insulin aspart 100units/ml solution for injection 3ml cartridges
53251	NovoRapid Penfill 100units/ml solution for injection 3ml cartridges (DE Pharmaceuticals)
10067	Insulin biphasic aspart human pyr 30:70; 100 units/ml Injection
5892	NovoRapid FlexPen 100units/ml solution for injection 3ml pre-filled pen (Novo Nordisk Ltd)
11337	NovoRapid Novolet 100units/ml solution for injection (Novo Nordisk Ltd)
6061	Novomix 30 30/70 100units/ml Injection (Novo Nordisk Ltd)
53118	NovoRapid FlexPen 100units/ml solution for injection 3ml pre-filled pen (Mawdsley-Brooks & Company Ltd)
29567	Insulin aspart 100units/ml solution for injection 10ml vials
6209	NovoRapid 100units/ml solution for injection 10ml vials (Novo Nordisk Ltd)
6447	Insulin aspart human pyr 100 iu/ml Injection
59533	NovoRapid FlexPen 100units/ml solution for injection 3ml pre-filled pen (Sigma Pharmaceuticals Plc)
51743	NovoRapid Penfill 100units/ml solution for injection 3ml cartridges (Sigma Pharmaceuticals Plc)
49108	NovoRapid Penfill 100units/ml solution for injection 3ml cartridges (Necessity Supplies Ltd)
46666	NovoRapid FlexTouch 100units/ml solution for injection 3ml pre-filled pen (Novo Nordisk Ltd)
19877	Insulin aspart 100units/ml solution for injection 3ml pre-filled disposable devices
5021	NovoRapid Penfill 100units/ml solution for injection 3ml cartridges (Novo Nordisk Ltd)
56489	NovoMix 30 Penfill 100units/ml suspension for injection 3ml cartridges (Waymade Healthcare Plc)
7267	NovoMix 30 Penfill 100units/ml suspension for injection 3ml cartridges (Novo Nordisk Ltd)
23099	Insulin aspart biphasic 30/70 100units/ml suspension for injection 3ml pre-filled disposable devices
7228	NovoMix 30 FlexPen 100units/ml suspension for injection 3ml pre-filled pen (Novo Nordisk Ltd)
24795	Insulin aspart biphasic 30/70 100units/ml suspension for injection 3ml cartridges
55462	Tresiba FlexTouch 100units/ml solution for injection 3ml pre-filled pen (Novo Nordisk Ltd)
55910	Tresiba Penfill 100units/ml solution for injection 3ml cartridges (Novo Nordisk Ltd)
55234	Tresiba FlexTouch 200units/ml solution for injection 3ml pre-filled pen (Novo Nordisk Ltd)
55907	Insulin degludec 100units/ml solution for injection 3ml cartridges
6958	Levemir FlexPen 100units/ml solution for injection 3ml pre-filled pen (Novo Nordisk Ltd)
55618	Levemir FlexPen 100units/ml solution for injection 3ml pre-filled pen (Waymade Healthcare Plc)
35260	Levemir InnoLet 100units/ml solution for injection 3ml pre-filled pen (Novo Nordisk Ltd)
6965	Levemir Penfill 100units/ml solution for injection 3ml cartridges (Novo Nordisk Ltd)
14301	Insulin detemir 100units/ml solution for injection 3ml cartridges

14330	Insulin detemir 100units/ml solution for injection 3ml pre-filled disposable devices
10184	Insulin detemir 100 iu/ml Solution for injection
7400	Insulin glargine 100units/ml solution for injection 3ml pre-filled disposable devices
5953	Insulin glargine 100iu/ml Injection
10225	Lantus 100units/ml solution for injection 3ml OptiClik cartridges (Sanofi)
36853	Lantus 100units/ml solution for injection 3ml pre-filled SoloStar pen (Sanofi)
7266	Lantus 100units/ml solution for injection 3ml cartridges (Sanofi)
10259	Insulin glargine 100units/ml solution for injection 10ml vials
7393	Insulin glargine 100units/ml solution for injection 3ml cartridges
50633	Lantus 100units/ml solution for injection 3ml cartridges (Necessity Supplies Ltd)
6057	Lantus 100iu/ml Injection (Aventis Pharma)
56495	Lantus 100units/ml solution for injection 3ml pre-filled OptiSet pen (Waymade Healthcare Plc)
7237	Lantus 100units/ml solution for injection 3ml pre-filled OptiSet pen (Sanofi)
7402	Lantus 100units/ml solution for injection 10ml vials (Sanofi)
49831	Lantus 100units/ml solution for injection 3ml pre-filled SoloStar pen (Necessity Supplies Ltd)
29953	Apidra 100units/ml solution for injection 3ml OptiClik cartridges (Sanofi)
21583	Apidra 100units/ml solution for injection 3ml pre-filled OptiSet pen (Sanofi)
28101	Insulin glulisine 100units/ml solution for injection 10ml vials
28442	Insulin glulisine 100unit/ml Solution for injection
14345	Apidra 100units/ml solution for injection 3ml cartridges (Sanofi)
21590	Insulin glulisine 100units/ml solution for injection 3ml pre-filled disposable devices
36920	Apidra 100units/ml solution for injection 3ml pre-filled SoloStar pen (Sanofi)
19491	Apidra 100units/ml solution for injection 10ml vials (Sanofi)
14299	Insulin glulisine 100units/ml solution for injection 3ml cartridges
53710	Insulin human 500units/ml solution for injection 20ml vials
36356	Insulin human 3mg inhalation powder blisters
31465	Exubera 1mg inhalation powder blisters (Pfizer Ltd)
62276	Humulin R 500units/ml solution for injection 20ml vials (Imported (United States))
36355	Insulin human 1mg inhalation powder blisters
31467	Exubera 3mg inhalation powder blisters (Pfizer Ltd)
28588	Hypurin Bovine Isophane 100units/ml suspension for injection 3ml cartridges (Wockhardt UK Ltd)
30236	Isophane insulin 100iu/ml Injection
36066	Insulin isophane bovine 100units/ml suspension for injection 3ml cartridges
15484	Insulin isophane bovine 100units/ml suspension for injection 1.5ml cartridges
18590	Insulin isophane bovine 100units/ml suspension for injection 10ml vials
47856	Neuphane 100unit/ml Injection (Wellcome Medical Division)
14340	Hypurin Bovine Isophane 100units/ml suspension for injection 10ml vials (Wockhardt UK Ltd)
13516	Hypurin bovine isophane 100unit/ml Injection (C P Pharmaceuticals Ltd)
38422	Isophane 100iu/ml Injection (Celltech Pharma Europe Ltd)
43950	Humulin I KwikPen 100units/ml suspension for injection 3ml pre-filled pen (Eli Lilly and Company Ltd)
10207	Insulin isophane human 100units/ml suspension for injection 3ml cartridges
14928	Insulatard 100units/ml suspension for injection 10ml vials (Novo Nordisk Ltd)
8118	Humaject i 100iu/ml Pen (Eli Lilly and Company Ltd)

35468	Insuman Basal 100units/ml suspension for injection 5ml vials (Sanofi)
9737	Insulatard innolet 100iu/ml Injection (Novo Nordisk Ltd)
27461	Insuman Basal 100units/ml suspension for injection 3ml cartridges (Sanofi)
33966	Insulatard 100unit/ml Injection (Novo Nordisk Ltd)
4760	Humulin i 100unit/ml Injection (Eli Lilly and Company Ltd)
14918	Humulin I 100units/ml suspension for injection 10ml vials (Eli Lilly and Company Ltd)
13729	Insulin isophane human emp 100unit/ml Injection
1593	Insulatard penfill 100 100iu/ml Penfill (Novo Nordisk Ltd)
23992	Insuman Basal 100units/ml suspension for injection 3ml pre-filled OptiSet pen (Sanofi)
14925	Insulin isophane human vial 100unit/ml Sterile suspension injection
55517	Insulin isophane human 100units/ml suspension for injection 10ml vials
7771	Human protaphane penfill 100 100unit/ml Penfill (Novo Nordisk Ltd)
52748	Insulatard Penfill 100units/ml suspension for injection 3ml cartridges (Waymade Healthcare Plc)
11080	Insulin isophane human prb 100iu/ml Injection
5501	Insuman basal 100iu/ml Injection (Aventis Pharma)
10175	Insulin isophane human 100units/ml suspension for injection 1.5ml cartridges
59500	Insulin isophane human 100units/ml suspension for injection 5ml vials
7772	Human protaphane 100unit/ml Injection (Novo Nordisk Ltd)
1595	Insulatard NovoLet 100units/ml suspension for injection (Novo Nordisk Ltd)
1886	Insulatard 100iu/ml GE injection (Novo Nordisk Ltd)
46001	Insuman Basal 100units/ml suspension for injection 3ml pre-filled SoloStar pen (Sanofi)
5891	Insulatard FlexPen 100units/ml suspension for injection (Novo Nordisk Ltd)
14357	Humulin I 100units/ml suspension for injection 3ml cartridges (Eli Lilly and Company Ltd)
10208	Insulatard InnoLet 100units/ml suspension for injection 3ml pre-filled pen (Novo Nordisk Ltd)
15961	Insulin isophane human crb 100iu/ml Injection
14290	Insulatard Penfill 100units/ml suspension for injection 3ml cartridges (Novo Nordisk Ltd)
25812	Insulin isophane human 100units/ml suspension for injection 3ml pre-filled disposable devices
10229	Humulin I Pen 100units/ml suspension for injection 3ml pre-filled pen (Eli Lilly and Company Ltd)
20422	Insuman comb 15 100iu/ml Injection (Aventis Pharma)
1843	Pork Insulatard 100units/ml suspension for injection 10ml vials (Novo Nordisk Ltd)
7350	Insulin isophane porcine 100units/ml suspension for injection 10ml vials
13819	Hypurin Porcine Isophane 100units/ml suspension for injection 1.5ml cartridges (C P Pharmaceuticals Ltd)
28183	Hypurin Porcine Isophane 100units/ml suspension for injection 10ml vials (Wockhardt UK Ltd)
30686	Insulin isophane porcine 100units/ml suspension for injection 3ml cartridges
8895	Initard 50/50 100unit/ml Injection (Novo Nordisk Ltd)
4247	Insulin isophane porcine 100units/ml suspension for injection 1.5ml cartridges
14933	Hypurin Porcine Isophane 100units/ml suspension for injection 3ml cartridges (Wockhardt UK Ltd)
18224	Humalog 100units/ml solution for injection 10ml vials (Eli Lilly and Company Ltd)
57564	Humalog KwikPen 100units/ml solution for injection 3ml pre-filled pen (Waymade Healthcare Plc)

7318	Humalog 100units/ml solution for injection 3ml cartridges (Eli Lilly and Company Ltd)
57529	Humalog 100units/ml solution for injection 10ml vials (Dowelhurst Ltd)
26060	Insulin lispro 100units/ml solution for injection 10ml vials
5214	Insulin lispro 100units/ml solution for injection 1.5ml cartridges
5250	Insulin biphasic lispro human prb 25:75; 100 units/ml Injection
14313	Insulin lispro 100units/ml solution for injection 3ml cartridges
322	Humalog 100units/ml solution for injection 1.5ml cartridges (Eli Lilly and Company Ltd)
14362	Insulin lispro 100units/ml solution for injection 3ml pre-filled disposable devices
38986	Humalog KwikPen 100units/ml solution for injection 3ml pre-filled pen (Eli Lilly and Company Ltd)
4715	Humalog mix 25 25/75 100units/ml Injection (Eli Lilly and Company Ltd)
27177	Insulin biphasic lispro human prb 50:50; 100 units/ml Injection
10264	Humalog Pen 100units/ml solution for injection 3ml pre-filled pen (Eli Lilly and Company Ltd)
55603	Humalog KwikPen 100units/ml solution for injection 3ml pre-filled pen (DE Pharmaceuticals)
18593	Humalog Mix50 100units/ml suspension for injection 3ml cartridges (Eli Lilly and Company Ltd)
43953	Insulin lispro biphasic 25/75 100units/ml suspension for injection 10ml vials
42395	Humalog Mix25 100units/ml suspension for injection 10ml vials (Eli Lilly and Company Ltd)
36146	Insulin lispro biphasic 50/50 100units/ml suspension for injection 3ml cartridges
57622	Humalog Mix50 KwikPen 100units/ml suspension for injection 3ml pre-filled pen (Waymade Healthcare Plc)
52522	Humalog Mix50 KwikPen 100units/ml suspension for injection 3ml pre-filled pen (DE Pharmaceuticals)
35701	Insulin lispro biphasic 50/50 100units/ml suspension for injection 3ml pre-filled disposable devices
10001	Humalog Mix50 Pen 100units/ml suspension for injection 3ml pre-filled pen (Eli Lilly and Company Ltd)
39086	Humalog Mix50 KwikPen 100units/ml suspension for injection 3ml pre-filled pen (Eli Lilly and Company Ltd)
31258	Insulin lispro biphasic 25/75 100units/ml suspension for injection 3ml pre-filled disposable devices
10243	Humalog Mix25 100units/ml suspension for injection 3ml cartridges (Eli Lilly and Company Ltd)
28185	Insulin lispro biphasic 25/75 100units/ml suspension for injection 3ml cartridges
14270	Humalog Mix25 Pen 100units/ml suspension for injection 3ml pre-filled pen (Eli Lilly and Company Ltd)
39006	Humalog Mix25 KwikPen 100units/ml suspension for injection 3ml pre-filled pen (Eli Lilly and Company Ltd)
14505	Insulin protamine zinc bovine 100units/ml suspension for injection 10ml vials
9503	Hypurin Bovine Protamine Zinc 100units/ml suspension for injection 10ml vials (Wockhardt UK Ltd)
18592	Insulin soluble bovine 100units/ml solution for injection 10ml vials
14938	Insulin soluble bovine cartridge 100unit/ml Solution for injection
14339	Hypurin Bovine Neutral 100units/ml solution for injection 10ml vials (Wockhardt UK Ltd)

23231	Hypurin Bovine Neutral 100units/ml solution for injection 3ml cartridges (Wockhardt UK Ltd)
10572	Insulin soluble bovine 100unit/ml Injection
24593	Neutral insulin bovine 100unit/ml Injection
12297	Hypurin bovine neutral 100unit/ml Injection (C P Pharmaceuticals Ltd)
47360	Neutral insulin 100unit/ml Injection (Celltech Pharma Europe Ltd)
1592	Actrapid penfill 100 100iu/ml Penfill (Novo Nordisk Ltd)
56115	Human Actrapid Penfill 100units/ml solution for injection 1.5ml cartridges (Novo Nordisk Ltd)
22945	Insuman rapid 100iu/ml Injection (Aventis Pharma)
10910	Humaject m2 100iu/ml M2 pen (Eli Lilly and Company Ltd)
1588	Actrapid 100iu/ml Injection (Novo Nordisk Ltd)
4199	Humulin m1 100unit/ml M1 injection (Eli Lilly and Company Ltd)
10915	Humaject m1 100iu/ml M1 pen (Eli Lilly and Company Ltd)
11107	Humulin m4 100unit/ml M4 injection (Eli Lilly and Company Ltd)
10887	Penmix 40/60 100iu/ml Penfill (Novo Nordisk Ltd)
3551	Mixtard 20 penfill 100 100iu/ml Penfill (Novo Nordisk Ltd)
22155	Humaject m5 100iu/ml M5 pen (Eli Lilly and Company Ltd)
11056	Insulin biphasic isophane human pyr 30:70; 100 units/ml Injection
7349	Actrapid 100units/ml solution for injection 10ml vials (Novo Nordisk Ltd)
1649	Human actraphane 100iu/ml Injection (Novo Nordisk Ltd)
22058	Pur-in mix 15/85 Injection (C P Pharmaceuticals Ltd)
14649	Insulin biphasic isophane human pyr 10:90; 100 units/ml Injection
2929	Mixtard 30 100iu/ml GE injection (Novo Nordisk Ltd)
2454	Mixtard 30 penfill 100 100iu/ml Penfill (Novo Nordisk Ltd)
1806	Penmix 30/70 100iu/ml Penfill (Novo Nordisk Ltd)
26403	Pur-in mix 25/75 Injection (C P Pharmaceuticals Ltd)
10484	Penmix 20/80 Penfill (Novo Nordisk Ltd)
3550	Mixtard 40 penfill 100 100iu/ml Penfill (Novo Nordisk Ltd)
54462	Insulin biphasic isophane human emp 25:75; 100 units/ml Injection
4198	Humulin m3 100unit/ml M3 injection (Eli Lilly and Company Ltd)
17809	Humaject m4 100iu/ml M4 pen (Eli Lilly and Company Ltd)
41959	Penject 100unit/ml Injection device (Hypoguard Ltd)
14644	Insulin biphasic isophane human prb 20:80; 100 units/ml Injection
21110	Insulin biphasic isophane human prb 50:50; 100 units/ml Injection
24846	Pur-in neutral 100unit/ml Injection (C P Pharmaceuticals Ltd)
34097	Human initard 50/50 100unit/ml Injection (Novo Nordisk Ltd)
12654	Insulin soluble human prb 100unit/ml Injection
17336	Novopen 100unit/ml Injection device (Novo Nordisk Ltd)
9341	Insulin biphasic isophane human prb 30:70; 100 units/ml Injection
16129	Insulin soluble human 100units/ml solution for injection 3ml cartridges
33167	Insulin biphasic isophane human crb 25:75; 100 units/ml Injection
3439	Penmix 10/90 Pen (Novo Nordisk Ltd)
11055	Insulin biphasic isophane human pyr 20:80; 100 units/ml Injection
1840	Humulin s 100unit/ml Injection (Eli Lilly and Company Ltd)
21347	Penmix 40/60 100iu/ml Injection (Novo Nordisk Ltd)
13837	Insulin biphasic isophane human prb 10:90; 100 units/ml Injection
5255	Mixtard 10 penfill 100 100iu/ml Penfill (Novo Nordisk Ltd)

15199	Insuman comb 25 100iu/ml Injection (Aventis Pharma)
15710	Insulin soluble human emp 100unit/ml Injection
21395	Insulin biphasic isophane human pyr 40:60; 100 units/ml Injection
21374	Insulin biphasic isophane human prb 40:60; 100 units/ml Injection
1594	Actrapid NovoLet 100units/ml solution for injection (Novo Nordisk Ltd)
22983	Insuman Rapid 100units/ml solution for injection 3ml cartridges (Sanofi)
26621	Insulin soluble human crb 100iu/ml Injection
12638	Insulin soluble human pyr 100unit/ml Injection
23993	Insuman Rapid 100units/ml solution for injection 3ml pre-filled OptiSet pen (Sanofi)
4706	Velosulin 100units/ml solution for injection 10ml vials (Novo Nordisk Ltd)
14944	Humulin S 100units/ml solution for injection 3ml cartridges (Eli Lilly and Company Ltd)
27614	Penmix 30/70 100iu/ml Injection (Novo Nordisk Ltd)
27402	Insulin soluble human 100units/ml solution for injection 10ml vials
36430	Insulin soluble human 100units/ml solution for injection 3ml pre-filled disposable devices
56502	Actrapid Penfill 100units/ml solution for injection 3ml cartridges (Novo Nordisk Ltd)
9565	HumaJect S Pen 100units/ml solution for injection (Eli Lilly and Company Ltd)
3396	Penmix 10/90 Penfill (Novo Nordisk Ltd)
21235	Humulin S 100units/ml solution for injection 10ml vials (Eli Lilly and Company Ltd)
2220	Penmix 20/80 Pen (Novo Nordisk Ltd)
29837	Insulin biphasic isophane human prb 25:75; 100 units/ml Injection
1805	Mixtard 30/70 100unit/ml Injection (Novo Nordisk Ltd)
41120	Insulin isophane biphasic human 50/50 100units/ml suspension for injection 3ml pre-filled disposable devices
10277	Humulin M3 100units/ml suspension for injection 3ml cartridges (Eli Lilly and Company Ltd)
2221	Mixtard 30 NovoLet 100units/ml suspension for injection (Novo Nordisk Ltd)
8203	Penmix 50/50 100iu/ml Penfill (Novo Nordisk Ltd)
31205	Insuman Comb 50 100units/ml suspension for injection 3ml pre-filled OptiSet pen (Sanofi)
22697	Insulin isophane biphasic human 50/50 100units/ml suspension for injection 1.5ml cartridges
24002	Insuman Comb 25 100units/ml suspension for injection 5ml vials (Sanofi)
21554	Insuman comb 50 100iu/ml Injection (Aventis Pharma)
60938	Mixtard 30 100units/ml suspension for injection 10ml vials (Waymade Healthcare Plc)
5933	Mixtard 50 NovoLet 100units/ml suspension for injection (Novo Nordisk Ltd)
60933	Humulin M3 100units/ml suspension for injection 10ml vials (Sigma Pharmaceuticals Plc)
50691	Human Mixtard 20 Penfill 100units/ml suspension for injection 1.5ml cartridges (Novo Nordisk Ltd)
16160	Humulin M3 Pen 100units/ml suspension for injection 3ml pre-filled pen (Eli Lilly and Company Ltd)
56857	Insulin isophane biphasic human 15/85 100units/ml suspension for injection 3ml cartridges
17731	Penmix 50/50 100iu/ml Injection (Novo Nordisk Ltd)
2812	Mixtard 40 NovoLet 100units/ml suspension for injection (Novo Nordisk Ltd)
25736	Insulin isophane biphasic human 10/90 100units/ml suspension for injection 3ml cartridges

16152	Insulin isophane biphasic human 30/70 100units/ml suspension for injection 3ml cartridges
52722	Human Mixtard 30 Penfill 100units/ml suspension for injection 1.5ml cartridges (Novo Nordisk Ltd)
12818	Human Mixtard 50 100units/ml suspension for injection 10ml vials (Novo Nordisk Ltd)
21422	Insulin isophane biphasic human 40/60 100units/ml suspension for injection 3ml cartridges
35253	Insuman Comb 50 100units/ml suspension for injection 3ml cartridges (Sanofi)
10245	Mixtard 10 Penfill 100units/ml suspension for injection 3ml cartridges (Novo Nordisk Ltd)
43991	Humulin M3 KwikPen 100units/ml suspension for injection 3ml pre-filled pen (Eli Lilly and Company Ltd)
36194	Insulin isophane biphasic human 25/75 100units/ml suspension for injection 3ml cartridges
21232	Insulin isophane biphasic human 30/70 100units/ml suspension for injection 10ml vials
13277	Mixtard 50 Penfill 100units/ml suspension for injection 3ml cartridges (Novo Nordisk Ltd)
7319	Mixtard 20 Penfill 100units/ml suspension for injection 3ml cartridges (Novo Nordisk Ltd)
25735	Insulin isophane biphasic human 20/80 100units/ml suspension for injection 3ml cartridges
24993	Insuman Comb 25 100units/ml suspension for injection 3ml cartridges (Sanofi)
28096	Insulin isophane biphasic human 50/50 100units/ml suspension for injection 3ml cartridges
44480	Insuman Comb 25 100units/ml suspension for injection 3ml pre-filled SoloStar pen (Sanofi)
7231	Mixtard 30 Penfill 100units/ml suspension for injection 3ml cartridges (Novo Nordisk Ltd)
33232	Insulin isophane biphasic human 50/50 100units/ml suspension for injection 5ml vials
30819	Insuman Comb 15 100units/ml suspension for injection 3ml pre-filled OptiSet pen (Sanofi)
10244	Mixtard 40 Penfill 100units/ml suspension for injection 3ml cartridges (Novo Nordisk Ltd)
45158	Insuman Comb 15 100units/ml suspension for injection 3ml cartridges (Sanofi)
7300	Mixtard 30 100units/ml suspension for injection 10ml vials (Novo Nordisk Ltd)
7793	HumaJect M3 Pen 100units/ml suspension for injection (Eli Lilly and Company Ltd)
8841	Humulin M5 100units/ml suspension for injection 10ml vials (Eli Lilly and Company Ltd)
4093	Humulin M2 100units/ml suspension for injection 3ml cartridges (Eli Lilly and Company Ltd)
25133	Insuman Comb 25 100units/ml suspension for injection 3ml pre-filled OptiSet pen (Sanofi)
5845	Mixtard 30 InnoLet 100units/ml suspension for injection 3ml pre-filled pen (Novo Nordisk Ltd)
2456	Mixtard 10 NovoLet 100units/ml suspension for injection (Novo Nordisk Ltd)
44378	Insulin isophane biphasic human 25/75 100units/ml suspension for injection 3ml pre-filled disposable devices
42954	Insulin isophane biphasic human 25/75 100units/ml suspension for injection 5ml vials

57620	Humulin M3 100units/ml suspension for injection 10ml vials (Mawdsley-Brooks & Company Ltd)
19513	Humulin M3 100units/ml suspension for injection 10ml vials (Eli Lilly and Company Ltd)
2455	Mixtard 20 NovoLet 100units/ml suspension for injection (Novo Nordisk Ltd)
4790	Mixtard 50 penfill 100 100iu/ml Penfill (Novo Nordisk Ltd)
19878	Insulin isophane biphasic human 30/70 100units/ml suspension for injection 3ml pre-filled disposable devices
13622	Hypurin porcine neutral 100unit/ml Injection (C P Pharmaceuticals Ltd)
27396	Insulin soluble porcine 100units/ml solution for injection 10ml vials
26098	Hypurin Porcine Neutral 100units/ml solution for injection 10ml vials (Wockhardt UK Ltd)
14930	Hypurin Porcine Neutral 100units/ml solution for injection 3ml cartridges (Wockhardt UK Ltd)
25479	Insulin soluble porcine 100units/ml solution for injection 3ml cartridges
1842	Pork velosulin 100unit/ml Injection (Novo Nordisk Ltd)
36513	Velosulin cartridge 100unit/ml Injection (Novo Nordisk Ltd)
4129	Insulin soluble porcine 100units/ml solution for injection 1.5ml cartridges
30209	Actrapid mc 100unit/ml Injection (Arun Products Ltd)
9521	Pork Actrapid 100units/ml solution for injection 10ml vials (Novo Nordisk Ltd)
36031	Insulin isophane biphasic porcine 30/70 100units/ml suspension for injection 3ml cartridges
14619	Insulin isophane biphasic porcine 30/70 100units/ml suspension for injection 1.5ml cartridges
20995	Hypurin Porcine 30/70 Mix 100units/ml suspension for injection 3ml cartridges (Wockhardt UK Ltd)
2459	Pork Mixtard 30 100units/ml suspension for injection 10ml vials (Novo Nordisk Ltd)
24800	Hypurin Porcine 30/70 Mix 100units/ml suspension for injection 10ml vials (Wockhardt UK Ltd)
27280	Insulin isophane biphasic porcine 30/70 100units/ml suspension for injection 10ml vials
9618	Hypurin Porcine 30/70 Mix 100units/ml suspension for injection 1.5ml cartridges (C P Pharmaceuticals Ltd)
9376	Insulin zinc suspension crystalline human pyr 100unit/ml long acting Injection
18931	Insulin zinc crystalline human 100units/ml suspension for injection 10ml vials
8322	Insulin zinc suspension mixed human pyr 100unit/ml Injection
7537	Humulin Zn 100units/ml suspension for injection 10ml vials (Eli Lilly and Company Ltd)
1844	Ultratard 100units/ml suspension for injection 10ml vials (Novo Nordisk Ltd)
16682	Tempulin 100unit/ml Injection (Knoll Ltd)
16700	Insulin zinc mixed bovine vial 100unit/ml Sterile suspension injection
12035	Insulin zinc mixed bovine 100units/ml suspension for injection 10ml vials
41834	Insulin zinc suspension lente 100iu/ml Injection (Celltech Pharma Europe Ltd)
17712	Hypurin Bovine Lente 100units/ml suspension for injection 10ml vials (Wockhardt UK Ltd)
10547	Humulin Lente 100units/ml suspension for injection 10ml vials (Eli Lilly and Company Ltd)
1587	Monotard 100units/ml suspension for injection 10ml vials (Novo Nordisk Ltd)
18461	Insulin zinc mixed human 100units/ml suspension for injection 10ml vials
44251	Insulin zinc suspension mixed porcine 100unit/ml Injection
34031	Monotard mc 100unit/ml Injection (Novo Nordisk Ltd)

26498	Insulin zinc suspension mixed bovine and porcine 100unit/ml Injection
4784	Lentard mc 100unit/ml Injection (Novo Nordisk Ltd)
12299	Semitard mc 100unit/ml Injection (Novo Nordisk Ltd)
34713	Insulin 1 ml syringe
2321	Insulin 0.5ml disposable syringe
23636	Insulin 1ml disposable syringe
40085	Insulin 2ml syringe
22060	Insulin 0.5 0.5ml Syringe
47588	Insulin 1ml click count
38093	Insulin 1ml pre-set syringe
16959	Hypodermic U100 insulin syringe sterile single use / single patient use 1ml with 12mm needle 0.3mm/30gauge
2808	INSULIN LENTARD INJ
9363	U100 Insulin syringe 0.5ml
30861	INSULIN ZINC HUMAN SUSPENSION
24485	INSULIN ZINC ANIMAL SUSPENSION
45045	NovoTwist hypodermic insulin needles for pre-filled / reusable pen injectors screw on 5mm/32gauge (Novo Nordisk Ltd)
56656	Kendall Magellan hypodermic U100 insulin syringe sterile single use / single patient use 0.3ml with 8mm safety needle 0.3mm/30gauge (Covidien (UK) Commercial Ltd)
54629	Insupen hypodermic insulin needles for pre-filled / reusable pen injectors screw on 8mm/31gauge (Spirit Healthcare Ltd)
6554	Autopen 24 hypodermic insulin injection pen reusable for 3ml cartridge 1 unit dial up / range 1-21 units (Owen Mumford Ltd)
43568	ClikSTAR hypodermic insulin injection pen reusable for 3ml cartridge 1 unit dial up / range 1-80 units Silver (Sanofi)
15040	INSULIN MONOPHANE (ISOPHANE) 100 I/U INJ
35454	Comfort Point hypodermic insulin needles for pre-filled / reusable pen injectors screw on 8mm/31gauge (Disposable Medical Equipment Ltd)
48633	Comfort Point hypodermic insulin needles for pre-filled / reusable pen injectors screw on 4mm/31gauge (Disposable Medical Equipment Ltd)
54573	Hypodermic insulin injection pen reusable for 3ml cartridge 1 unit dial up / range 1-21 units
55687	Insulin degludec 100units/ml solution for injection 3ml pre-filled disposable devices
43833	ClikSTAR hypodermic insulin injection pen reusable for 3ml cartridge 1 unit dial up / range 1-80 units Blue (Sanofi)
8483	MONOJECT INSULIN NEEDLES
52232	Hypodermic insulin injection pen reusable for 3ml cartridge 0.5 unit dial up / range 1-30 units
33356	Hypodermic insulin needles for pre-filled / reusable pen injectors screw on 12mm/29gauge
49479	Hypodermic insulin needles for pre-filled / reusable pen injectors screw on 4mm/31gauge
12840	B-d u-100 0.5ml Insulin syringe (Becton, Dickinson UK Ltd)
56691	Insulin degludec 200units/ml solution for injection 3ml pre-filled disposable devices
55627	Insupen hypodermic insulin needles for pre-filled / reusable pen injectors screw on 5mm/31gauge (Spirit Healthcare Ltd)

12892	Clinipak u100 single use ins syr+12mm need28g 0.5ml [rand] 0.5ml Insulin syringe with 28gauge needle 12mm (Rand Rocket Ltd)
31267	INSULIN PUR-IN MIX 50/50 100 I/U INJ
24845	INSULIN PUR-IN ISOPHANE 100 I/U INJ
26795	SYRINGE INSULIN DISPOSABLE
11521	Hypodermic U100 insulin syringe sterile single use / single patient use 1ml with 12mm needle 0.4mm/27gauge
5967	Hypodermic U100 insulin syringe sterile single use / single patient use 0.5ml with not less than 12mm needle 0.33mm/29gauge
14504	INSULIN HYPURIN PROTAMINE ZINC 100 I/U INJ
14642	Bd m 0.3ml Insulin syringe with 29gauge needle 12mm (Becton, Dickinson UK Ltd)
40555	HumaPen Memoir hypodermic insulin injection pen reusable for 3ml cartridge 1 unit dial up / range 1-60 units (Eli Lilly and Company Ltd)
58745	GlucoRx FinePoint hypodermic insulin needles for pre-filled / reusable pen injectors screw on 4mm/31gauge (GlucoRx Ltd)
11271	B-d u-100 1ml Insulin syringe (Becton, Dickinson UK Ltd)
6233	Mylife Clickfine hypodermic insulin needles for pre-filled / reusable pen injectors snap on 10mm/29gauge (Ypsomed Ltd)
58579	Hypodermic insulin needles for pre-filled / reusable pen injectors screw on 4mm/32.5gauge
8354	INSULIN ISOPHANE 70%/NEUTRAL 30% 100 I/U INJ
56879	HumaPen Savvio hypodermic insulin injection pen reusable for 3ml cartridge 1 unit dial up / range 1-60 units Graphite (Eli Lilly and Company Ltd)
10566	INSULIN HUMULIN M CARTRIDGE 100 I/U
13550	INSULIN BP 100 I/U
5345	Monoject hypodermic U100 insulin syringe sterile single use / single patient use 0.5ml with 12mm needle 0.36mm/28gauge (Covidien (UK) Commercial Ltd)
5121	BD Micro-Fine + hypodermic insulin needles for pre-filled / reusable pen injectors screw on 12.7mm/29gauge (Becton, Dickinson UK Ltd)
7763	INSULIN NEUPHANE (ISOPHANE)(PURIFIED) 100 I/U INJ
8646	INSULIN ZINC CRYSTALLINE susp 100 I/U INJ
1591	U100 Insulin syringe 1ml
13036	Unifine hypodermic U100 insulin syringe sterile single use / single patient use 0.5ml with 12mm needle 0.33mm/29gauge (Owen Mumford Ltd)
24722	INSULIN ISOPHANE 50%/NEUTRAL 50% 100 I/U INJ
21459	SYRINGE INSULIN U100 S/U+8MM NEEDLE
27151	SYRINGE INSULIN U100 S/U+8MM NEEDLE
50798	Hypodermic insulin injection pen reusable for 3ml cartridge 0.5 unit dial up / range 1-35 units
48342	HumaPen Luxura hypodermic insulin injection pen reusable for 3ml cartridge 1 unit dial up / range 1-60 units Burgundy (Eli Lilly and Company Ltd)
61562	Hypodermic insulin needles for pre-filled / reusable pen injectors screw on 8mm/29gauge
16866	Unifine hypodermic U100 insulin syringe sterile single use / single patient use 0.3ml with 12mm needle 0.33mm/29gauge (Owen Mumford Ltd)
9834	Mylife Clickfine hypodermic insulin needles for pre-filled / reusable pen injectors snap on 12mm/29gauge (Ypsomed Ltd)

51650	Omnican Fine hypodermic insulin needles for pre-filled / reusable pen injectors screw on 6mm/31gauge (B.Braun Medical Ltd)
12300	SYRINGE INSULIN (BS1619/1) 2ML
37427	HumaPen Luxura HD hypodermic insulin injection pen reusable for 3ml cartridge 0.5 unit dial up / range 1-30 units (Eli Lilly and Company Ltd)
39150	Hypodermic U100 insulin syringe sterile single use / single patient use 1ml with 12mm safety needle 0.33mm/29gauge
5634	Hypodermic U100 insulin syringe sterile single use / single patient use 1ml with 12mm needle 0.36mm/28gauge
19707	INSULIN HUMULIN S (NEUTRAL SOLUBLE)
7412	U100 Insulin syringe 0.5ml
18446	Unifine hypodermic U100 insulin syringe sterile single use / single patient use 1ml with 12mm needle 0.33mm/29gauge (Owen Mumford Ltd)
7959	INSULIN MIXTARD 30/70 40 I/U INJ
18149	Monoject u100 insulin syringe 12mm(29g)0.5ml 12mm29G 0.5ml Insulin syringe (Covidien (UK) Commercial Ltd)
10133	U100 Insulin syringe 1ml
22823	INSULIN ISOPHANE (PURIFIED) 100 I/U INJ
60028	Hypodermic insulin needles for pre-filled / reusable pen injectors snap on 4mm/32gauge
11346	B-d u-100 0.3ml Insulin syringe (Becton, Dickinson UK Ltd)
14506	INSULIN BOVINE PROTAMINE ZINC 100 I/U INJ
13009	Hypodermic U100 insulin syringe sterile single use / single patient use 0.5ml with 12mm needle 0.36mm/28gauge
56352	HumaPen Savvio hypodermic insulin injection pen reusable for 3ml cartridge 1 unit dial up / range 1-60 units Green (Eli Lilly and Company Ltd)
44810	Hypodermic insulin needles for pre-filled / reusable pen injectors screw on 5mm/32gauge
19029	SYRINGE PRE-SET INSULIN FOR BLIND 2ML
38774	NovoPen 4 hypodermic insulin injection pen reusable for 3ml cartridge 1 unit dial up / range 1-60 units Blue (Novo Nordisk Ltd)
10258	Hypodermic insulin needles for pre-filled / reusable pen injectors screw on 8mm/31gauge
21945	INSULIN PORK INSULATARD
56808	Omnican Fine hypodermic insulin needles for pre-filled / reusable pen injectors screw on 12mm/29gauge (B.Braun Medical Ltd)
43670	Hypodermic insulin needles for pre-filled / reusable pen injectors screw on 4mm/32gauge
6991	BD Micro-Fine + hypodermic U100 insulin syringe sterile single use / single patient use 0.5ml with 12.7mm needle 0.33mm/29gauge (Becton, Dickinson UK Ltd)
5649	Autopen hypodermic insulin injection pen reusable for 1.5ml cartridge 1 unit dial up / range 1-16 units (Owen Mumford Ltd)
33101	Hypodermic insulin needles for pre-filled / reusable pen injectors screw on 12.7mm/29gauge
14887	Hypodermic insulin needles for pre-filled / reusable pen injectors screw on 5mm/31gauge
1751	Hypodermic U100 insulin syringe glass reusable 1ml
8839	INSULIN SEMITARD 100 I/U INJ

28978	INSULIN PUR-IN MIX 15/85 100 I/U INJ
19977	Omnican 50 hypodermic U100 insulin syringe sterile single use / single patient use 0.5ml with 12mm needle 0.3mm/30gauge (B.Braun Medical Ltd)
51612	Hypodermic insulin needles for pre-filled / reusable pen injectors screw on 5mm/29gauge
35218	Optipen pro 1 yellow insulin pen 3ml/1-60 units 3ml/1-60 units Insulin pen (Aventis Pharma)
60609	Insupen hypodermic insulin needles for pre-filled / reusable pen injectors screw on 6mm/32gauge (Spirit Healthcare Ltd)
58801	GlucoRx FinePoint hypodermic insulin needles for pre-filled / reusable pen injectors screw on 10mm/29gauge (GlucoRx Ltd)
20195	INSULIN BOVINE PROTAMINE ZINC 40 I/U INJ
6009	Myjector hypodermic U100 insulin syringe sterile single use / single patient use 0.5ml with 12mm needle 0.4mm/27gauge (Terumo UK Ltd)
6091	NovoPen 3 Fun hypodermic insulin injection pen reusable for 3ml cartridge 1 unit dial up / range 2-70 units Red (Novo Nordisk Ltd)
35057	OptiClik hypodermic insulin injection pen reusable for 3ml cartridge 1 unit dial up / range 1-80 units Blue (Sanofi)
5059	NovoPen 3 Classic hypodermic insulin injection pen reusable for 3ml cartridge 1 unit dial up / range 2-70 units (Novo Nordisk Ltd)
51881	Omnican Fine hypodermic insulin needles for pre-filled / reusable pen injectors screw on 8mm/31gauge (B.Braun Medical Ltd)
10145	Humapen luxura insulin pen 3ml/1-60 units Insulin pen 3ml/1-60 units (Eli Lilly and Company Ltd)
28723	INSULIN ZINC BOVINE SUSPENSION
6470	Autopen 24 hypodermic insulin injection pen reusable for 3ml cartridge 2 unit dial up / range 2-42 units (Owen Mumford Ltd)
13474	U100 Insulin syringe Sp.36 [2A] 1ml
60967	Nanopass hypodermic insulin needles for pre-filled / reusable pen injectors screw on 8mm/32.5gauge (Terumo UK Ltd)
48576	BD AutoShield Duo hypodermic insulin needles for pre-filled / reusable pen injectors screw on 5mm/30gauge (Becton, Dickinson UK Ltd)
23003	INSULIN ISOPHANE (NPH) 40 I/U
14646	Hypodermic U100 insulin syringe sterile single use / single patient use 0.3ml with 12mm needle 0.33mm/29gauge
19829	INSULIN NOVO MONOTARD MC
48501	HumaPen Luxura hypodermic insulin injection pen reusable for 3ml cartridge 1 unit dial up / range 1-60 units Champagne (Eli Lilly and Company Ltd)
12060	INSULIN QUICKSOL (SOLUBLE NEUTRAL) 100 I/U INJ
7861	INSULIN HUMULIN S (NEUTRAL) CARTRIDGE 100 I/U
5267	BD Micro-Fine + hypodermic U100 insulin syringe sterile single use / single patient use 0.5ml with 8mm needle 0.3mm/30gauge (Becton, Dickinson UK Ltd)
5022	NovoFine hypodermic insulin needles for pre-filled / reusable pen injectors screw on 12mm/28gauge (Novo Nordisk Ltd)
62180	Insulin aspart 100units/ml solution for injection 1.6ml cartridges
57387	Kendall Magellan hypodermic U100 insulin syringe sterile single use / single patient use 0.3ml with 12mm safety needle 0.33mm/29gauge (Covidien (UK) Commercial Ltd)

10242	Hypodermic insulin needles for pre-filled / reusable pen injectors screw on 6mm/31gauge
27911	INSULIN HUMAN ACTRAPID PENFILL
25006	INSULIN HUMAN ACTRAPID (NEUTRAL)
58817	GlucoRx FinePoint hypodermic insulin needles for pre-filled / reusable pen injectors screw on 12mm/29gauge (GlucoRx Ltd)
6753	Novopen Junior hypodermic insulin injection pen reusable for 3ml cartridge 0.5 unit dial up / range 1-35 units Green (Novo Nordisk Ltd)
21223	U100 Insulin syringe 0.3ml
35017	Optipen pro 1 white insulin pen 3ml/1-60 units 3ml/1-60 units Insulin pen (Aventis Pharma)
22946	Omnican 100 hypodermic U100 insulin syringe sterile single use / single patient use 1ml with 12mm needle 0.3mm/30gauge (B.Braun Medical Ltd)
49451	Mylife Clickfine AutoProtect hypodermic insulin needles for pre-filled / reusable pen injectors screw on 8mm/29gauge (Ypsomed Ltd)
57451	Microdot Droplet hypodermic insulin needles for pre-filled / reusable pen injectors screw on 8mm/31gauge (Cambridge Sensors Ltd)
59846	Mylife Clickfine hypodermic insulin needles for pre-filled / reusable pen injectors snap on 4mm/32gauge (Ypsomed Ltd)
48811	Unifine Pentips Plus hypodermic insulin needles for pre-filled / reusable pen injectors screw on 6mm/31gauge (Owen Mumford Ltd)
7757	INSULIN NEULENTE (ZINC SUSP)(PURIFIED) 100 I/U INJ
7062	Myjector hypodermic U100 insulin syringe sterile single use / single patient use 1ml with 12mm needle 0.4mm/27gauge (Terumo UK Ltd)
5850	Mylife Clickfine hypodermic insulin needles for pre-filled / reusable pen injectors snap on 6mm/31gauge (Ypsomed Ltd)
55521	Insupen hypodermic insulin needles for pre-filled / reusable pen injectors screw on 4mm/32gauge (Spirit Healthcare Ltd)
16209	INSULIN HYPURIN SOLUBLE 100 I/U INJ
8838	INSULIN SEMITARD 40 I/U INJ
5789	Hypodermic U100 insulin syringe sterile single use / single patient use 0.5ml with 8mm needle 0.3mm/30gauge
11245	B-d u-100 0.3ml Insulin syringe (Becton, Dickinson UK Ltd)
7783	INSULIN ISOPHANE (HUMAN) 100 I/U INJ
60951	Insulin human 100units/ml solution for injection 10ml vials
5164	NovoPen 3 Demi hypodermic insulin injection pen reusable for 3ml cartridge 0.5 unit dial up / range 1-35 units (Novo Nordisk Ltd)
321	INSULIN HUMAN ACTRAPID (NEUTRAL) 40 I/U INJ
57243	Omnican Fine hypodermic insulin needles for pre-filled / reusable pen injectors screw on 10mm/30gauge (B.Braun Medical Ltd)
6060	Unifine Pentips hypodermic insulin needles for pre-filled / reusable pen injectors screw on 6mm/30gauge (Owen Mumford Ltd)
4896	Monoject hypodermic U100 insulin syringe sterile single use / single patient use 1ml with 12mm needle 0.36mm/28gauge (Covidien (UK) Commercial Ltd)
22161	INSULIN HUMULIN M1 VIAL
5769	Hypodermic U100 insulin syringe sterile single use / single patient use 0.5ml with 12mm needle 0.4mm/27gauge

54028	Unifine Pentips Plus hypodermic insulin needles for pre-filled / reusable pen injectors screw on 12mm/29gauge (Owen Mumford Ltd)
59005	Mylife Penfine Classic hypodermic insulin needles for pre-filled / reusable pen injectors screw on 8mm/31gauge (Ypsomed Ltd)
8376	INSULIN ISOPHANE 100 I/U
58961	Mylife Penfine Classic hypodermic insulin needles for pre-filled / reusable pen injectors screw on 4mm/32gauge (Ypsomed Ltd)
23437	Hypodermic insulin needles for pre-filled / reusable pen injectors screw on 12mm/28gauge
18208	Hypodermic insulin needles for pre-filled / reusable pen injectors snap on 10mm/29gauge
22987	Hypodermic insulin needles for pre-filled / reusable pen injectors snap on 12mm/29gauge
59004	Mylife Penfine Classic hypodermic insulin needles for pre-filled / reusable pen injectors screw on 6mm/32gauge (Ypsomed Ltd)
60750	Hypodermic insulin needles for pre-filled / reusable pen injectors screw on 4mm/33gauge
11878	Hypodermic U100 insulin syringe sterile single use / single patient use 1ml with 8mm needle 0.3mm/30gauge
35081	OptiClik hypodermic insulin injection pen reusable for 3ml cartridge 1 unit dial up / range 1-80 units Grey (Sanofi)
49052	Unifine Pentips Plus hypodermic insulin needles for pre-filled / reusable pen injectors screw on 8mm/31gauge (Owen Mumford Ltd)
48765	Unifine Pentips hypodermic insulin needles for pre-filled / reusable pen injectors screw on 5mm/31gauge (Owen Mumford Ltd)
54885	Hypodermic insulin needles for pre-filled / reusable pen injectors screw on 8mm/32gauge
18195	Hypodermic insulin needles for pre-filled / reusable pen injectors snap on 6mm/31gauge
20672	INSULIN HUM/ACTRAPID
51107	Hypodermic insulin injection pen reusable for 3ml cartridge 2 unit dial up / range 2-42 units
47751	TS-A Safety hypodermic U100 insulin syringe sterile single use / single patient use 1ml with 12mm safety needle 0.33mm/29gauge (Minsmed)
48435	BD AutoShield hypodermic insulin needles for pre-filled / reusable pen injectors screw on 5mm/29gauge (Becton, Dickinson UK Ltd)
5421	NovoFine hypodermic insulin needles for pre-filled / reusable pen injectors screw on 6mm/31gauge (Novo Nordisk Ltd)
11408	Autopen Classic hypodermic insulin injection pen reusable for 3ml cartridge 1 unit dial up / range 1-21 units (Owen Mumford Ltd)
59133	BD SafetyGlide hypodermic U100 insulin syringe sterile single use / single patient use 0.5ml with 8mm safety needle 0.3mm/30gauge (Becton, Dickinson UK Ltd)
58798	GlucoRx FinePoint hypodermic insulin needles for pre-filled / reusable pen injectors screw on 6mm/31gauge (GlucoRx Ltd)
6724	U100 Insulin syringe 0.3ml
9578	Unifine Pentips hypodermic insulin needles for pre-filled / reusable pen injectors screw on 12mm/29gauge (Owen Mumford Ltd)
26784	INSULIN ZINC SEMILENTE SUSP BP 100 I/U INJ

5557	NovoPen 3 Fun hypodermic insulin injection pen reusable for 3ml cartridge 1 unit dial up / range 2-70 units Blue (Novo Nordisk Ltd)
11086	B-d u-100 0.5ml Insulin syringe (Becton, Dickinson UK Ltd)
25422	Hypoguard u100 click/count 1ml Insulin syringe (Hypoguard Ltd)
14191	Autopen Junior hypodermic insulin injection pen reusable for 3ml cartridge 2 unit dial up / range 2-42 units (Owen Mumford Ltd)
30918	ABCare hypodermic U100 insulin syringe glass reusable 0.5ml (Rand Rocket Ltd)
35143	NovoFine Autocover hypodermic insulin needles for pre-filled / reusable pen injectors screw on 8mm/30gauge (Novo Nordisk Ltd)
24866	INSULIN INSULATARD (LEO RETARD) 40 I/U INJ
6138	HumaPen Ergo hypodermic insulin injection pen reusable for 3ml cartridge 1 unit dial up / range 1-60 units Burgundy (Eli Lilly and Company Ltd)
7764	INSULIN NEUSULIN (NEUTRAL)(PURIFIED) 100 I/U INJ
15624	INSULIN ISOPHANE (HIGHLY PURIFIED) 100 I/U INJ
49172	NovoPen Echo hypodermic insulin injection pen reusable for 3ml cartridge 0.5 unit dial up / range 0.5-30 units Red (Novo Nordisk Ltd)
1645	INSULIN NOVO ACTRAPID MC 100 I/U INJ
11345	Bd Ultra Pen 3ml Insulin pen (Becton, Dickinson UK Ltd)
58754	BD SafetyGlide hypodermic U100 insulin syringe sterile single use / single patient use 1ml with 12mm safety needle 0.33mm/29gauge (Becton, Dickinson UK Ltd)
31699	ABCare hypodermic U100 insulin syringe glass reusable 1ml (Rand Rocket Ltd)
56639	HumaPen Savvio hypodermic insulin injection pen reusable for 3ml cartridge 1 unit dial up / range 1-60 units Blue (Eli Lilly and Company Ltd)
5142	BD Micro-Fine + hypodermic insulin needles for pre-filled / reusable pen injectors screw on 8mm/31gauge (Becton, Dickinson UK Ltd)
48771	Unifine Pentips Plus hypodermic insulin needles for pre-filled / reusable pen injectors screw on 5mm/31gauge (Owen Mumford Ltd)
4248	INSULIN NOVO ULTRATARD MC 100 I/U INJ
6981	Novopen Junior hypodermic insulin injection pen reusable for 3ml cartridge 0.5 unit dial up / range 1-35 units Yellow (Novo Nordisk Ltd)
58878	Hypodermic insulin needles for pre-filled / reusable pen injectors screw on 10mm/29gauge
6781	Autopen Classic hypodermic insulin injection pen reusable for 3ml cartridge 2 unit dial up / range 2-42 units (Owen Mumford Ltd)
7164	Unifine Pentips hypodermic insulin needles for pre-filled / reusable pen injectors screw on 6mm/31gauge (Owen Mumford Ltd)
58449	Unifine Pentips hypodermic insulin needles for pre-filled / reusable pen injectors screw on 4mm/32gauge (Owen Mumford Ltd)
56642	Microdot Droplet hypodermic insulin needles for pre-filled / reusable pen injectors screw on 6mm/31gauge (Cambridge Sensors Ltd)
38808	NovoPen 4 hypodermic insulin injection pen reusable for 3ml cartridge 1 unit dial up / range 1-60 units Silver (Novo Nordisk Ltd)
22328	Unifine single use Insulin syringe with 30gauge needle 8mm 0.5ml (Owen Mumford Ltd)
15294	Innovo hypodermic insulin injection pen reusable for 3ml cartridge 1 unit dial up / range 1-70 units Green (Novo Nordisk Ltd)
25786	Comfort Point hypodermic insulin needles for pre-filled / reusable pen injectors screw on 6mm/31gauge (Disposable Medical Equipment Ltd)
1839	INSULIN HUMULIN I (ISOPHANE) 100 I/U INJ

18301	INSULIN SOLUBLE INJ I/U^2
13108	Autopen Special Edition hypodermic insulin injection pen reusable for 3ml cartridge 1 unit dial up / range 1-21 units (Owen Mumford Ltd)
58578	Kendall Magellan hypodermic U100 insulin syringe sterile single use / single patient use 0.5ml with 12mm safety needle 0.33mm/29gauge (Covidien (UK) Commercial Ltd)
32053	INSULIN HUMALOG MIX 25
13969	U100 Insulin syringe 0.3ml
1643	INSULIN NOVO MONOTARD MC 100 I/U INJ
57388	Kendall Magellan hypodermic U100 insulin syringe sterile single use / single patient use 1ml with 8mm safety needle 0.3mm/30gauge (Covidien (UK) Commercial Ltd)
56983	Insupen hypodermic insulin needles for pre-filled / reusable pen injectors screw on 6mm/31gauge (Spirit Healthcare Ltd)
31466	Exubera insulin release unit (Pfizer Ltd)
55746	Insupen hypodermic insulin needles for pre-filled / reusable pen injectors screw on 8mm/32gauge (Spirit Healthcare Ltd)
7203	Unifine Pentips hypodermic insulin needles for pre-filled / reusable pen injectors screw on 8mm/31gauge (Owen Mumford Ltd)
9702	BD Micro-Fine + hypodermic U100 insulin syringe sterile single use / single patient use 1ml with 12.7mm needle 0.33mm/29gauge (Becton, Dickinson UK Ltd)
22496	INSULIN ZINC LENTE PURIFIED SUSPENSION
16389	Myjector hypodermic U100 insulin syringe sterile single use / single patient use 1ml with 12mm needle 0.33mm/29gauge (Terumo UK Ltd)
22974	Hypodermic insulin needles for pre-filled / reusable pen injectors snap on 8mm/31gauge
3076	NovoFine hypodermic insulin needles for pre-filled / reusable pen injectors screw on 6mm/30gauge (Novo Nordisk Ltd)
20671	INSULIN HUM/ACTRAPHANE
58995	BD SafetyGlide hypodermic U100 insulin syringe sterile single use / single patient use 0.5ml with 12mm safety needle 0.33mm/29gauge (Becton, Dickinson UK Ltd)
59243	BD SafetyGlide hypodermic U100 insulin syringe sterile single use / single patient use 0.3ml with 8mm safety needle 0.25mm/31gauge (Becton, Dickinson UK Ltd)
30305	Hypodermic U100 insulin syringe sterile single use / single patient use 0.5ml with 12mm needle 0.3mm/30gauge
53437	Hypodermic insulin needles for pre-filled / reusable pen injectors screw on 5mm/30gauge
60621	Hypodermic insulin injection pen reusable for 3ml cartridge 0.5 unit dial up / range 0.5-30 units
58884	GlucoRx FinePoint hypodermic insulin needles for pre-filled / reusable pen injectors screw on 8mm/31gauge (GlucoRx Ltd)
52319	Hypodermic insulin injection pen reusable for 3ml cartridge 1 unit dial up / range 1-80 units
57153	Kendall Magellan hypodermic U100 insulin syringe sterile single use / single patient use 0.5ml with 8mm safety needle 0.3mm/30gauge (Covidien (UK) Commercial Ltd)
18645	INSULIN NEUTRAL (PURIFIED) 100 I/U INJ
54027	Hypodermic insulin needles for pre-filled / reusable pen injectors snap on 4.5mm/31gauge
22806	INSULIN PORK ACTRAPID
22094	INSULIN HUMULIN M2 VIAL

56785	HumaPen Savvio hypodermic insulin injection pen reusable for 3ml cartridge 1 unit dial up / range 1-60 units Red (Eli Lilly and Company Ltd)
62098	Hypodermic insulin needles for pre-filled / reusable pen injectors screw on 8mm/32.5gauge
5015	NovoFine hypodermic insulin needles for pre-filled / reusable pen injectors screw on 8mm/30gauge (Novo Nordisk Ltd)
6730	Hypodermic U100 insulin syringe sterile single use / single patient use 1ml with not less than 12mm needle 0.33mm/29gauge
59311	Kendall Magellan hypodermic U100 insulin syringe sterile single use / single patient use 1ml with 12mm safety needle 0.33mm/29gauge (Covidien (UK) Commercial Ltd)
57493	HumaPen Savvio hypodermic insulin injection pen reusable for 3ml cartridge 1 unit dial up / range 1-60 units Silver (Eli Lilly and Company Ltd)
15951	Autopen Junior hypodermic insulin injection pen reusable for 3ml cartridge 1 unit dial up / range 1-21 units (Owen Mumford Ltd)
37055	Comfort Point hypodermic U100 insulin syringe sterile single use / single patient use 0.5ml with 12mm needle 0.33mm/29gauge (Disposable Medical Equipment Ltd)
57744	Omnican Fine hypodermic insulin needles for pre-filled / reusable pen injectors screw on 4mm/31gauge (B.Braun Medical Ltd)
7127	BD Micro-Fine + hypodermic U100 insulin syringe sterile single use / single patient use 1ml with 8mm needle 0.3mm/30gauge (Becton, Dickinson UK Ltd)
61780	Insupen hypodermic insulin needles for pre-filled / reusable pen injectors screw on 12mm/29gauge (Spirit Healthcare Ltd)
35078	Optipen pro 1 blue insulin pen 3ml/1-60 units 3ml/1-60 units Insulin pen (Aventis Pharma)
17643	Autopen Special Edition hypodermic insulin injection pen reusable for 3ml cartridge 2 unit dial up / range 2-42 units (Owen Mumford Ltd)
15895	Innovo hypodermic insulin injection pen reusable for 3ml cartridge 1 unit dial up / range 1-70 units Orange (Novo Nordisk Ltd)
48829	NovoPen Echo hypodermic insulin injection pen reusable for 3ml cartridge 0.5 unit dial up / range 0.5-30 units Blue (Novo Nordisk Ltd)
36959	Comfort Point hypodermic insulin needles for pre-filled / reusable pen injectors screw on 12mm/29gauge (Disposable Medical Equipment Ltd)
53148	Hypodermic insulin injection pen reusable for 3ml cartridge 1 unit dial up / range 1-60 units
1589	U100 Insulin syringe 0.5ml
10545	INSULIN HUMULIN M4 CARTRIDGE 100 I/U
45639	IME-FINE hypodermic insulin needles for pre-filled / reusable pen injectors screw on 6mm/31gauge (Arctic Medical Ltd)
5742	Unifine Pentips hypodermic insulin needles for pre-filled / reusable pen injectors screw on 8mm/30gauge (Owen Mumford Ltd)
49307	Mylife Clickfine hypodermic insulin needles for pre-filled / reusable pen injectors snap on 4.5mm/31gauge (Ypsomed Ltd)
62562	Insupen hypodermic insulin needles for pre-filled / reusable pen injectors screw on 4mm/33gauge (Spirit Healthcare Ltd)
10546	INSULIN HUMULIN M4 100 I/U INJ
54886	Hypodermic insulin needles for pre-filled / reusable pen injectors screw on 6mm/32gauge

56939	HumaPen Savvio hypodermic insulin injection pen reusable for 3ml cartridge 1 unit dial up / range 1-60 units Pink (Eli Lilly and Company Ltd)
6228	BD Micro-Fine + hypodermic U100 insulin syringe sterile single use / single patient use 0.3ml with 8mm needle 0.3mm/30gauge (Becton, Dickinson UK Ltd)
12244	INSULIN ZINC BOVINE susp 100 I/U INJ
13274	Clinipak u100 single use ins syr+12mm need28g 1ml [rand] 1ml Insulin syringe with 28gauge needle 12mm (Rand Rocket Ltd)
56624	Kendall Magellan hypodermic U100 insulin syringe sterile single use / single patient use 0.5ml with 12mm safety needle 0.3mm/30gauge (Covidien (UK) Commercial Ltd)
9619	Bd Ultra Pen 1.5ml Insulin pen (Becton, Dickinson UK Ltd)
5966	Mylife Clickfine hypodermic insulin needles for pre-filled / reusable pen injectors snap on 8mm/31gauge (Ypsomed Ltd)
2373	INSULIN HUMAN VELOSULIN 100 I/U INJ
43489	BD Micro-Fine Ultra hypodermic insulin needles for pre-filled / reusable pen injectors screw on 4mm/32gauge (Becton, Dickinson UK Ltd)
59475	Nanopass hypodermic insulin needles for pre-filled / reusable pen injectors screw on 4mm/32.5gauge (Terumo UK Ltd)
59793	Microdot Droplet hypodermic insulin needles for pre-filled / reusable pen injectors screw on 4mm/32gauge (Cambridge Sensors Ltd)
17076	Omnican 100 hypodermic U100 insulin syringe sterile single use / single patient use 1ml with 8mm needle 0.3mm/30gauge (B.Braun Medical Ltd)
9079	INSULIN SOLUBLE 100 I/U INJ
5620	U100 Insulin syringe 0.3ml
38236	Comfort Point hypodermic U100 insulin syringe sterile single use / single patient use 1ml with 12mm needle 0.33mm/29gauge (Disposable Medical Equipment Ltd)
20196	INSULIN SOLUBLE 40 I/U INJ
7765	INSULIN NEUTRAL (HUMAN) 100 I/U INJ
58581	GlucoRx FinePoint hypodermic insulin needles for pre-filled / reusable pen injectors screw on 5mm/31gauge (GlucoRx Ltd)
10691	INSULIN ISOPHANE (NPH) 100 I/U INJ
5873	BD Micro-Fine + hypodermic insulin needles for pre-filled / reusable pen injectors screw on 5mm/31gauge (Becton, Dickinson UK Ltd)
24554	Unifine single use Insulin syringe with 30gauge needle 8mm 0.3ml (Owen Mumford Ltd)
28666	Omnican 50 hypodermic U100 insulin syringe sterile single use / single patient use 0.5ml with 8mm needle 0.3mm/30gauge (B.Braun Medical Ltd)
19271	Hypodermic insulin needles for pre-filled / reusable pen injectors screw on 8mm/30gauge
6831	HumaPen Ergo hypodermic insulin injection pen reusable for 3ml cartridge 1 unit dial up / range 1-60 units Teal (Eli Lilly and Company Ltd)
6378	Hypodermic U100 insulin syringe sterile single use / single patient use 0.3ml with 8mm needle 0.3mm/30gauge
13096	Autopen hypodermic insulin injection pen reusable for 1.5ml cartridge 2 unit dial up / range 2-32 units (Owen Mumford Ltd)
7075	Optipen Pro 1 hypodermic insulin injection pen reusable for 3ml cartridge 1 unit dial up / range 1-60 units (Sanofi)
42797	Injex ampoule pack 60501 (Ocon Chemicals Ltd)
60626	InsuJet starter set 012007GBSP Grey (Spirit Healthcare Ltd)
42305	Injex 10ml vial adaptor pack 60502 (Ocon Chemicals Ltd)

51836	InsuJet 3ml cartridge adaptor pack 012071GB1503 (Spirit Healthcare Ltd)
26338	Injex starter set 60500 (Ocon Chemicals Ltd)
61845	NovoRapid PumpCart 100units/ml solution for injection 1.6ml cartridges (Novo Nordisk Ltd)
51182	InsuJet starter set 012003GBSP Lime (Spirit Healthcare Ltd)
44601	Injex 4 monthly refill pack 60504 (Ocon Chemicals Ltd)
49509	InsuJet starter set 012005GBSP Blue (Spirit Healthcare Ltd)
49508	InsuJet 10ml vial adaptor pack 01207GB1510 (Spirit Healthcare Ltd)
49507	InsuJet nozzle pack 012070GB15 (Spirit Healthcare Ltd)
