# Supplementary material for: Can commonly prescribed drugs be repurposed for the prevention or treatment of Alzheimer's and other neurodegenerative diseases? Protocol for an observational cohort study in the UK Clinical Practice Research Datalink
Source: BMJ Open. 2016 Dec 12;6(12):e012044. doi: 10.1136/bmjopen-2016-012044 (PMC5168636; doi:10.1136/bmjopen-2016-012044)
Supplement: supplementary file [file bmjopen-2016-012044supp4.pdf]

Medical code list: possible Alzheimer's disease

Read Code	Read Term
E00..00	Senile and presenile organic psychotic conditions
E00..11	Senile dementia
E00..12	Senile/presenile dementia
E000.00	Uncomplicated senile dementia
E001.00	Presenile dementia
E001000	Uncomplicated presenile dementia
E001100	Presenile dementia with delirium
E001200	Presenile dementia with paranoia
E001300	Presenile dementia with depression
E001z00	Presenile dementia NOS
E002.00	Senile dementia with depressive or paranoid features
E002000	Senile dementia with paranoia
E002100	Senile dementia with depression
E002z00	Senile dementia with depressive or paranoid features NOS
E003.00	Senile dementia with delirium
E00z.00	Senile or presenile psychoses NOS
Eu02z11	[X] Presenile dementia NOS
Eu02z12	[X] Presenile psychosis NOS
Eu02z14	[X] Senile dementia NOS
Eu02z15	[X] Senile psychosis NOS
Eu02z16	[X] Senile dementia, depressed or paranoid type
Eu05700	[X]Mild cognitive disorder
F11z.11	Cerebral atrophy

Medical code list: probable Alzheimer's disease

Read Code	Read Term
Eu00.00	[X]Dementia in Alzheimer's disease
Eu00000	[X]Dementia in Alzheimer's disease with early onset
Eu00011	[X]Presenile dementia,Alzheimer's type
Eu00012	[X]Primary degen dementia, Alzheimer's type, presenile onset
Eu00013	[X]Alzheimer's disease type 2
Eu00100	[X]Dementia in Alzheimer's disease with late onset
Eu00111	[X]Alzheimer's disease type 1
Eu00112	[X]Senile dementia,Alzheimer's type
Eu00113	[X]Primary degen dementia of Alzheimer's type, senile onset
Eu00200	[X]Dementia in Alzheimer's dis, atypical or mixed type
Eu00z00	[X]Dementia in Alzheimer's disease, unspecified
Eu00z11	[X]Alzheimer's dementia unspec
F110.00	Alzheimer's disease
F110000	Alzheimer's disease with early onset
F110100	Alzheimer's disease with late onset
Fyu3000	[X]Other Alzheimer's disease

Medical code list: non-specific dementia (non Alzheimer's disease dementias)

Read Code	Read Term
6AB..00	Dementia annual review
9hD0.00	Excepted from dementia quality indicators: Patient unsuitabl
9hD1.00	Excepted from dementia quality indicators: Informed dissent
E00y.00	Other senile and presenile organic psychoses
E041.00	Dementia in conditions EC
Eu02.00	[X]Dementia in other diseases classified elsewhere
Eu02y00	[X]Dementia in other specified diseases classif elsewhere
Eu02z00	[X] Unspecified dementia
Eu02z13	[X] Primary degenerative dementia NOS
F112.00	Senile degeneration of brain

Medical code list: other dementia (non Alzheimer's disease dementias)

Read Code	Read Term
8BPa.00	Antipsychotic drug therapy for dementia
E02y100	Drug-induced dementia
Eu01111	[X]Predominantly cortical dementia
Eu02000	[X]Dementia in Pick's disease
Eu02100	[X]Dementia in Creutzfeldt-Jakob disease
Eu02200	[X]Dementia in Huntington's disease
Eu02300	[X]Dementia in Parkinson's disease
Eu02400	[X]Dementia in human immunodef virus [HIV] disease
Eu02500	[X]Lewy body dementia
Eu04100	[X]Delirium superimposed on dementia
F111.00	Pick's disease
F116.00	Lewy body disease
F11x200	Cerebral degeneration due to cerebrovascular disease

Medical code list: vascular dementia (non Alzheimer's disease dementias)

Read Code	Read Term
E004.00	Arteriosclerotic dementia
E004.11	Multi infarct dementia
E004000	Uncomplicated arteriosclerotic dementia
E004100	Arteriosclerotic dementia with delirium
E004200	Arteriosclerotic dementia with paranoia
E004300	Arteriosclerotic dementia with depression
E004z00	Arteriosclerotic dementia NOS
Eu01.00	[X]Vascular dementia
Eu01.11	[X]Arteriosclerotic dementia
Eu01000	[X]Vascular dementia of acute onset
Eu01100	[X]Multi-infarct dementia
Eu01200	[X]Subcortical vascular dementia
Eu01300	[X]Mixed cortical and subcortical vascular dementia
Eu01y00	[X]Other vascular dementia
Eu01z00	[X]Vascular dementia, unspecified

Medical code list: amyotrophic lateral sclerosis

Read Code	Read Term
7Q04100	Amyotrophic lateral sclerosis drugs Band 1
F152.00	Motor neurone disease
F152000	Amyotrophic lateral sclerosis
F152z00	Motor neurone disease NOS

Medical code list: Parkinson's disease

Read Code	Read Term
F12..00	Parkinson's disease
F12z.00	Parkinson's disease NOS
