# Supplementary material for: Can commonly prescribed drugs be repurposed for the prevention or treatment of Alzheimer's and other neurodegenerative diseases? Protocol for an observational cohort study in the UK Clinical Practice Research Datalink
Source: BMJ Open. 2016 Dec 12;6(12):e012044. doi: 10.1136/bmjopen-2016-012044 (PMC5168636; doi:10.1136/bmjopen-2016-012044)
Supplement: supplementary file [file bmjopen-2016-012044supp5.pdf]

Product code list: treatments for Alzheimer's disease

Product Code	Product Name
2930	Donepezil 5mg tablets
2931	Donepezil 10mg tablets
4597	Rivastigmine 1.5mg capsules
5247	Aricept 10mg tablets (Eisai Ltd)
5334	Reminyl 12mg tablets (Shire Pharmaceuticals Ltd)
5400	Aricept 5mg tablets (Eisai Ltd)
5616	Exelon 6mg capsules (Novartis Pharmaceuticals UK Ltd)
6225	Memantine 10mg tablets
7329	Galantamine 20mg/5ml oral solution sugar free
7361	Galantamine 24mg modified-release capsules
9786	Rivastigmine 6mg capsules
9854	Reminyl 4mg tablets (Shire Pharmaceuticals Ltd)
9966	Ebixa 5mg/pump actuation oral solution (Lundbeck Ltd)
10187	Galantamine 4mg tablets
10255	Galantamine 8mg modified-release capsules
11546	Exelon 1.5mg capsules (Novartis Pharmaceuticals UK Ltd)
11635	Galantamine 12mg tablets
11654	Galantamine 8mg tablets
11716	Exelon 3mg capsules (Novartis Pharmaceuticals UK Ltd)
11751	Rivastigmine 3mg capsules
11752	Rivastigmine 4.5mg capsules
11827	Rivastigmine 2mg/ml oral solution sugar free
11837	Memantine 10mg/ml oral solution sugar free
14309	Galantamine 16mg modified-release capsules
18062	Reminyl 8mg tablets (Shire Pharmaceuticals Ltd)
18556	Exelon 2mg/ml oral solution (Novartis Pharmaceuticals UK Ltd)
18587	Reminyl XL 8mg capsules (Shire Pharmaceuticals Ltd)
18800	Ebixa 10mg tablets (Lundbeck Ltd)
20140	Reminyl XL 16mg capsules (Shire Pharmaceuticals Ltd)
20404	Exelon 4.5mg capsules (Novartis Pharmaceuticals UK Ltd)
24088	Reminyl XL 24mg capsules (Shire Pharmaceuticals Ltd)
29288	Reminyl 4mg/ml oral solution (Shire Pharmaceuticals Ltd)
35088	Donepezil 10mg orodispersible tablets sugar free
35179	Donepezil 5mg orodispersible tablets sugar free
36848	Aricept Evess 5mg orodispersible tablets (Eisai Ltd)
36976	Rivastigmine 4.6mg/24hours transdermal patches
37132	Rivastigmine 9.5mg/24hours transdermal patches
37188	Aricept Evess 10mg orodispersible tablets (Eisai Ltd)
37444	Exelon 4.6mg/24hours transdermal patches (Novartis Pharmaceuticals UK Ltd)
37957	Exelon 9.5mg/24hours transdermal patches (Novartis Pharmaceuticals UK Ltd)
38976	Memantine 5mg+10mg+15mg+20mg Tablet
39240	Memantine 20mg tablets
39362	Ebixa tablets treatment initiation pack (Lundbeck Ltd)
39363	Ebixa 20mg tablets (Lundbeck Ltd)
48015	Galsya XL 24mg capsules (Consilient Health Ltd)
48482	Galsya XL 8mg capsules (Consilient Health Ltd)

53842	Aricept 5mg tablets (Waymade Healthcare Plc)
53882	Rivastigmine 2mg/ml oral solution
55720	Gatalin XL 24mg capsules (Aspire Pharma Ltd)
55928	Exelon 4.5mg capsules (Waymade Healthcare Plc)
56600	Donepezil 5mg tablets (Zentiva)
56709	Gatalin XL 16mg capsules (Aspire Pharma Ltd)
56771	Rivastigmine 3mg capsules (Dr Reddy's Laboratories (UK) Ltd)
57139	Ebixa 10mg tablets (DE Pharmaceuticals)
57171	Erastig 9.5mg/24hours transdermal patches (Teva UK Ltd)
57627	Erastig 4.6mg/24hours transdermal patches (Teva UK Ltd)
58709	Donepezil 10mg tablets (A A H Pharmaceuticals Ltd)
58780	Voleze 9.5mg/24hours transdermal patches (Focus Pharmaceuticals Ltd)
58947	Donepezil 10mg tablets (Accord Healthcare Ltd)
59871	Donepezil 10mg/5ml oral suspension
60107	Donepezil 5mg tablets (Alliance Healthcare (Distribution) Ltd)
60493	Galantex XL 24mg capsules (Creo Pharma Ltd)
60723	Rivastigmine 6mg capsules (Waymade Healthcare Plc)
61385	Nemdatine 10mg tablets (Actavis UK Ltd)
61476	Acumor XL 24mg capsules (Generics (UK) Ltd)
61618	Nemdatine 20mg tablets (Actavis UK Ltd)
61921	Luventa XL 24mg capsules (Fontus Health Ltd)
62164	Alzest 9.5mg/24hours transdermal patches (Dr Reddy's Laboratories (UK) Ltd)

Product code list: treatments for amyotrophic lateral sclerosis

Product Code	Product Name
3975	Riluzole 50mg tablets
18838	Rilutek 50mg tablets (Sanofi)

Product code list: treatments for Parkinson's disease

Product Code	Product Name
514	Cabergoline 500microgram tablets
546	Sinemet 110 Tablet (Bristol-Myers Squibb Pharmaceuticals Ltd)
758	Entacapone 200mg tablets
772	Ropinirole 1mg tablets
811	Pramipexole 88microgram tablets
1853	Half sinemet cr 25mg+100mg Tablet (Bristol-Myers Squibb Pharmaceuticals Ltd)
2074	Sinemet 275 Tablet (Bristol-Myers Squibb Pharmaceuticals Ltd)
2279	Bromocriptine 2.5mg tablets
2464	Pergolide 250microgram tablets
2465	Madopar CR capsules (Roche Products Ltd)
2466	Madopar 100mg/25mg capsules (Roche Products Ltd)
2467	Selegiline 5mg tablets
2534	Sinemet plus Tablet (Bristol-Myers Squibb Pharmaceuticals Ltd)
2739	Sinemet 62.5 Tablet (Bristol-Myers Squibb Pharmaceuticals Ltd)
2829	Sinemet CR 50mg+200mg Tablet (Bristol-Myers Squibb Pharmaceuticals Ltd)
3562	Madopar 50mg/12.5mg capsules (Roche Products Ltd)
3640	Selegiline 10mg tablets
3641	Levodopa with benserazide 100mg + 25mg Capsule
3642	Co-beneldopa 25mg/100mg capsules
3910	Madopar 50mg/12.5mg dispersible tablets (Roche Products Ltd)
4146	Pergolide 50microgram tablets
4300	Bromocriptine 5mg capsules
4581	Dostinex 500microgram tablets (Pfizer Ltd)
4866	Co-careldopa 12.5mg/50mg tablets
4941	Pergolide 1mg tablets
4975	Bromocriptine 1mg tablets
5233	Selegiline 10mg/5ml oral solution
5248	Co-beneldopa 25mg/100mg modified-release capsules
5310	Co-careldopa 25mg/250mg tablets
5339	Amantadine 100mg capsules
5389	Cabergoline 1mg tablets
5406	Cabergoline 2mg tablets
5464	Co-beneldopa 12.5mg/50mg capsules
5487	Apomorphine 2mg sublingual tablets sugar free
5535	Apomorphine 3mg sublingual tablets sugar free
5575	Uprima 2mg sublingual tablets (Abbott Laboratories Ltd)
5665	Uprima 3mg sublingual tablets (Abbott Laboratories Ltd)
5673	Co-beneldopa 25mg/100mg dispersible tablets sugar free
5674	Ropinirole 250microgram tablets
5675	Co-careldopa 25mg/100mg modified-release tablets
5766	Pramipexole 180microgram tablets
5869	Madopar 100mg/25mg dispersible tablets (Roche Products Ltd)
5909	Pramipexole 700microgram tablets
6012	Ropinirole 250micrograms with 500micrograms with 1mg tablet
6016	Cabergoline 4mg tablets
6035	Amantadine 50mg/5ml oral solution sugar free

6122	Ropinirole 5mg tablets
6143	Ropinirole 500micrograms with 1mg with 2mg tablet
6156	Co-careldopa 25mg/100mg tablets
7040	Rasagiline 1mg tablets
7051	Selegiline 1.25mg oral lyophilisates sugar free
7246	Co-careldopa 50mg/200mg modified-release tablets
7256	Madopar 200mg/50mg capsules (Roche Products Ltd)
7339	Mirapexin 0.18mg tablets (Boehringer Ingelheim Ltd)
7386	Stalevo 150mg/37.5mg/200mg tablets (Orion Pharma (UK) Ltd)
7386	Stalevo 150mg/37.5mg/200mg tablets (Orion Pharma (UK) Ltd)
7428	Symmetrel 100mg capsules (Alliance Pharmaceuticals Ltd)
7879	Levodopa with benserazide 200mg + 50mg Capsule
8407	Levodopa with benserazide 50mg + 12.5mg Capsule
8408	Levodopa with benserazide 50mg + 12.5mg Dispersible tablet
8426	Eldepryl 5mg tablets (Orion Pharma (UK) Ltd)
9283	Co-careldopa 10mg/100mg tablets
9327	Tolcapone 100mg tablets
9333	Parlodel 2.5mg Tablet (Novartis Pharmaceuticals UK Ltd)
9512	Co-beneldopa 50mg/200mg capsules
9701	Apomorphine 10mg/ml injection
9799	Ropinirole 2mg tablets
10142	Stalevo 100mg/25mg/200mg tablets (Orion Pharma (UK) Ltd)
10142	Stalevo 100mg/25mg/200mg tablets (Orion Pharma (UK) Ltd)
11235	Co-beneldopa 12.5mg/50mg dispersible tablets sugar free
11277	Pergolide Starter Pack (Pergolide 50 micrograms tablet with Pergolide 250 micrograms tablet) 81 tablets
11541	Cabaser 1mg tablets (Pfizer Ltd)
11586	Zelapar 1.25mg oral lyophilisates (Teva UK Ltd)
12057	Bromocriptine 10mg capsules
12481	Levodopa 125mg Capsule
12762	ReQuip 250microgram tablets (GlaxoSmithKline UK Ltd)
13403	Parlodel 1mg Tablet (Novartis Pharmaceuticals UK Ltd)
13515	Parlodel 5mg Capsule (Novartis Pharmaceuticals UK Ltd)
13736	Celance 250microgram tablets (Eli Lilly and Company Ltd)
13793	Celance 1mg tablets (Eli Lilly and Company Ltd)
14140	Cabaser 4mg tablets (Pfizer Ltd)
14342	Ropinirole 500microgram tablets
14914	Rotigotine 2mg/24hours transdermal patches
14915	Rotigotine 6mg/24hours transdermal patches
14916	Stalevo 50mg/12.5mg/200mg tablets (Orion Pharma (UK) Ltd)
14916	Stalevo 50mg/12.5mg/200mg tablets (Orion Pharma (UK) Ltd)
14934	ReQuip 1mg tablets (GlaxoSmithKline UK Ltd)
14935	Apomorphine 50mg/10ml solution for infusion pre-filled syringes
16224	Eldepryl 10mg tablets (Orion Pharma (UK) Ltd)
16617	Mirapexin 0.088mg tablets (Boehringer Ingelheim Ltd)
16780	Levodopa 500mg tablets
16817	Benserazide 50mg with Levodopa 200mg capsules
16861	Levodopa with benserazide 100mg + 25mg Dispersible tablet
16933	Mirapexin 0.7mg tablets (Boehringer Ingelheim Ltd)

16970	Levodopa with benserazide 100mg + 25mg Modified-release capsule
17053	Rotigotine 4mg/24hours transdermal patches
17058	Adartrel 250microgram tablets (GlaxoSmithKline UK Ltd)
17066	Adartrel 500microgram tablets (GlaxoSmithKline UK Ltd)
17443	Cabaser 2mg tablets (Pfizer Ltd)
17619	ReQuip 2mg tablets (GlaxoSmithKline UK Ltd)
17622	ReQuip 5mg tablets (GlaxoSmithKline UK Ltd)
18346	Levodopa 250mg Capsule
18566	APO-go PFS 50mg/10ml solution for infusion pre-filled syringes (Britannia Pharmaceuticals Ltd)
18715	Brocadopa 125mg Capsule (Yamanouchi Pharma Ltd)
18891	Adartrel 2mg tablets (GlaxoSmithKline UK Ltd)
19267	Apo-go 10mg/ml Injection (Britannia Pharmaceuticals Ltd)
19492	Neupro 2mg/24hours transdermal patches (UCB Pharma Ltd)
19498	Neupro 4mg/24hours transdermal patches (UCB Pharma Ltd)
19505	Neupro 6mg/24hours transdermal patches (UCB Pharma Ltd)
19592	Larodopa 500mg Tablet (Cambridge Laboratories Ltd)
20023	Celance 50microgram tablets (Eli Lilly and Company Ltd)
20211	Britaject 10mg/ml Subcutaneous injection (Britannia Pharmaceuticals Ltd)
20651	Comtess 200mg tablets (Orion Pharma (UK) Ltd)
21118	Tasmar fc 100mg Tablet (Roche Products Ltd)
21361	Parlodel 10mg Capsule (Novartis Pharmaceuticals UK Ltd)
21745	Symmetrel 50mg/5ml syrup (Alliance Pharmaceuticals Ltd)
21793	Azilect 1mg tablets (Teva UK Ltd)
22602	Rotigotine 2mg/24hr with 4mg/24hr with 6mg/24hr with 8mg/24hr patch
22604	Rotigotine 8mg/24hours transdermal patches
22675	Tolcapone fc 200mg Tablet
23293	Neupro 8mg/24hours transdermal patches (UCB Pharma Ltd)
24528	Brocadopa 500mg Capsule (Yamanouchi Pharma Ltd)
25288	Co-careldopa 10mg/100mg tablets (Teva UK Ltd)
25298	Eldepryl 10mg/5ml syrup (Orion Pharma (UK) Ltd)
25844	Levodopa 500mg Capsule
25890	Lysovir 100mg capsules (Alliance Pharmaceuticals Ltd)
26041	Tasmar 100mg tablets (Meda Pharmaceuticals Ltd)
26257	Centrapryl 5mg Tablet (Opus Pharmaceuticals Ltd)
27302	Brocadopa 250mg Capsule (Yamanouchi Pharma Ltd)
27649	Benserazide 12.5mg with levodopa 50mg capsules
27738	Stilline 10mg Tablet (Berk Pharmaceuticals Ltd)
28590	Vivapryl 5mg Tablet (Viatris Pharmaceuticals Ltd)
29846	Vivapryl 10mg Tablet (Viatris Pharmaceuticals Ltd)
31268	Co-careldopa 25mg/250mg tablets (Teva UK Ltd)
31356	Tasmar fc 200mg Tablet (Roche Products Ltd)
31682	Benserazide 12.5mg with Levodopa 50mg dispersible tablets
32003	Benserazide 25mg with Levodopa 100mg dispersible tablet
32067	Bromocriptine 2.5mg Tablet (Generics (UK) Ltd)
33133	Apomorphine 50mg/5ml solution for injection ampoules
33541	Co-careldopa 5mg/20mg/1ml intestinal gel 100ml cassette
33781	Apomorphine 20mg/2ml solution for injection ampoules
34123	Selegiline 5mg tablets (IVAX Pharmaceuticals UK Ltd)

34132	Bromocriptine 2.5mg tablets (A A H Pharmaceuticals Ltd)
34219	Bromocriptine 2.5mg Tablet (Berk Pharmaceuticals Ltd)
34250	Selegiline 10mg tablets (IVAX Pharmaceuticals UK Ltd)
35255	Apomorphine 30mg/3ml solution for injection pre-filled disposable devices
35407	Benserazide 25mg with levodopa 100mg capsules
35939	APO-go 50mg/5ml solution for injection ampoules (Britannia Pharmaceuticals Ltd)
36038	APO-go PEN 30mg/3ml solution for injection (Britannia Pharmaceuticals Ltd)
37489	Benserazide 25mg with Levodopa 100mg modified-release capsules
37635	Pramipexole 350microgram tablets
37984	Mirapexin 0.35mg tablets (Boehringer Ingelheim Ltd)
38151	Ropinirole 8mg modified-release tablets
38199	APO-go 20mg/2ml solution for injection ampoules (Britannia Pharmaceuticals Ltd)
38245	Ropinirole 2mg modified-release tablets
38249	Ropinirole 4mg modified-release tablets
38250	ReQuip XL 4mg tablets (GlaxoSmithKline UK Ltd)
38251	ReQuip XL 2mg tablets (GlaxoSmithKline UK Ltd)
38256	ReQuip XL 8mg tablets (GlaxoSmithKline UK Ltd)
38357	Stalevo 200mg/50mg/200mg tablets (Orion Pharma (UK) Ltd)
38357	Stalevo 200mg/50mg/200mg tablets (Orion Pharma (UK) Ltd)
39348	Caramet 50mg/200mg CR tablets (Teva UK Ltd)
40039	Parlodel 2.5mg tablets (Meda Pharmaceuticals Ltd)
40235	Selegiline 5mg tablets (Niche Generics Ltd)
40306	Stalevo 75mg/18.75mg/200mg tablets (Orion Pharma (UK) Ltd)
40306	Stalevo 75mg/18.75mg/200mg tablets (Orion Pharma (UK) Ltd)
40418	Stilline 5mg Tablet (Berk Pharmaceuticals Ltd)
40452	Caramet 25mg/100mg CR tablets (Teva UK Ltd)
40568	Stalevo 125mg/31.25mg/200mg tablets (Orion Pharma (UK) Ltd)
40568	Stalevo 125mg/31.25mg/200mg tablets (Orion Pharma (UK) Ltd)
40866	Rotigotine 1mg/24hours transdermal patches
40947	Parlodel 5mg capsules (Meda Pharmaceuticals Ltd)
41091	Co-careldopa 25mg/100mg/5ml oral suspension
41147	Neupro 1mg/24hours transdermal patches (UCB Pharma Ltd)
41219	Pramipexole 260microgram modified-release tablets
41242	Pramipexole 3.15mg modified-release tablets
41243	Pramipexole 1.05mg modified-release tablets
41265	Pramipexole 520microgram modified-release tablets
41272	Pramipexole 2.1mg modified-release tablets
41309	Rotigotine 3mg/24hours transdermal patches
41350	Mirapexin 2.1mg modified-release tablets (Boehringer Ingelheim Ltd)
41358	Mirapexin 0.52mg modified-release tablets (Boehringer Ingelheim Ltd)
41359	Mirapexin 0.26mg modified-release tablets (Boehringer Ingelheim Ltd)
41360	Mirapexin 1.05mg modified-release tablets (Boehringer Ingelheim Ltd)
41370	Co-careldopa 25mg/100mg/5ml oral solution
41464	Ropinirole 250microgram tablets (Zentiva)
41484	Mirapexin 3.15mg modified-release tablets (Boehringer Ingelheim Ltd)
41707	Selegiline 10mg tablets (Teva UK Ltd)
41804	Parlodel 1mg tablets (Meda Pharmaceuticals Ltd)
42041	Sinemet 12.5mg/50mg tablets (Merck Sharp & Dohme Ltd)

42147	Sinemet Plus 25mg/100mg tablets (Merck Sharp & Dohme Ltd)
42172	Half Sinemet CR 25mg/100mg tablets (Merck Sharp & Dohme Ltd)
42215	Co-careldopa 25mg/100mg tablets (Teva UK Ltd)
42255	Sinemet CR 50mg/200mg tablets (Merck Sharp & Dohme Ltd)
42262	Sinemet 25mg/250mg tablets (Merck Sharp & Dohme Ltd)
42293	Sinemet 10mg/100mg tablets (Merck Sharp & Dohme Ltd)
42649	Neupro 3mg/24hours transdermal patches (UCB Pharma Ltd)
42808	Selegiline 10mg tablets (Niche Generics Ltd)
43033	Tilolec 200mg/50mg modified-release tablets (Tillomed Laboratories Ltd)
43631	Co-careldopa 50mg/200mg modified-release tablets (A A H Pharmaceuticals Ltd)
43718	Lecado 25mg+100mg Modified-release tablet (Sandoz Ltd)
43855	Lecado 50mg+200mg Modified-release tablet (Sandoz Ltd)
44486	Pramipexole 1.57mg modified-release tablets
44566	Mirapexin 1.57mg modified-release tablets (Boehringer Ingelheim Ltd)
44649	Pramipexole 2.62mg modified-release tablets
44750	Mirapexin 2.62mg modified-release tablets (Boehringer Ingelheim Ltd)
45303	Ropinirole 2mg tablets (Generics (UK) Ltd)
45761	Duodopa intestinal gel 100ml cassette (AbbVie Ltd)
46660	Neliprax 0.35mg tablets (Aspire Pharma Ltd)
47248	Stalevo 175mg/43.75mg/200mg tablets (Orion Pharma (UK) Ltd)
47248	Stalevo 175mg/43.75mg/200mg tablets (Orion Pharma (UK) Ltd)
47730	Pramipexole 88microgram tablets (Actavis UK Ltd)
47762	Spiroco XL 2mg tablets (Teva UK Ltd)
47763	Spiroco XL 8mg tablets (Teva UK Ltd)
48075	Spiroco XL 4mg tablets (Teva UK Ltd)
49481	Sinemet Plus 25mg/100mg tablets (Waymade Healthcare Plc)
50289	Mirapexin 0.088mg tablets (Waymade Healthcare Plc)
51023	Sinemet 10mg/100mg tablets (Lexon (UK) Ltd)
52376	Ralnea XL 2mg tablets (Consilient Health Ltd)
52694	Sinemet Plus 25mg/100mg tablets (DE Pharmaceuticals)
53299	Sinemet 25mg/250mg tablets (Mawdsley-Brooks & Company Ltd)
53526	Half Sinemet CR 25mg/100mg tablets (DE Pharmaceuticals)
53837	Ropinirole 1mg tablets (A A H Pharmaceuticals Ltd)
54469	Pramipexole 88microgram tablets (Sigma Pharmaceuticals Plc)
55508	Co-careldopa 6.25mg/25mg/5ml oral suspension
55876	Pergolide 50microgram tablets (A A H Pharmaceuticals Ltd)
56261	Stalevo 100mg/25mg/200mg tablets (Lexon (UK) Ltd)
56261	Stalevo 100mg/25mg/200mg tablets (Lexon (UK) Ltd)
56442	Bromocriptine 2.5mg tablets (Kent Pharmaceuticals Ltd)
56507	Sinemet 10mg/100mg tablets (Sigma Pharmaceuticals Plc)
57082	Co-careldopa 10mg/100mg tablets (A A H Pharmaceuticals Ltd)
57511	ReQuip XL 2mg tablets (DE Pharmaceuticals)
57518	Neupro 8mg/24hours transdermal patches (Mawdsley-Brooks & Company Ltd)
57586	Sinemet 25mg/250mg tablets (Dowelhurst Ltd)
57635	Co-beneldopa 12.5mg/50mg capsules (Teva UK Ltd)
57652	Ropinirole 1mg tablets (Waymade Healthcare Plc)
58776	Pramipexole 88microgram tablets (A A H Pharmaceuticals Ltd)
58831	Raponer XL 8mg tablets (Actavis UK Ltd)

59192	Ropinirole 250microgram tablets (A A H Pharmaceuticals Ltd)
59833	Ralnea XL 8mg tablets (Consilient Health Ltd)
59980	Repinex XL 8mg tablets (Aspire Pharma Ltd)
59997	Repinex XL 2mg tablets (Aspire Pharma Ltd)
60818	Pramipexole 88microgram tablets (Generics (UK) Ltd)
61452	ReQuip 2mg tablets (DE Pharmaceuticals)
61511	Raponer XL 2mg tablets (Actavis UK Ltd)
61512	Raponer XL 4mg tablets (Actavis UK Ltd)
62274	Oprymea 0.52mg modified-release tablets (Consilient Health Ltd)
62435	Co-careldopa 12.5mg/50mg/5ml oral suspension
62482	Sastravi 100mg/25mg/200mg tablets (Actavis UK Ltd)
62482	Sastravi 100mg/25mg/200mg tablets (Actavis UK Ltd)
62543	Sastravi 150mg/37.5mg/200mg tablets (Actavis UK Ltd)
62543	Sastravi 150mg/37.5mg/200mg tablets (Actavis UK Ltd)
62652	Bromocriptine 2.5mg Tablet (Approved Prescription Services Ltd)
