# Supplementary material for: Can commonly prescribed drugs be repurposed for the prevention or treatment of Alzheimer's and other neurodegenerative diseases? Protocol for an observational cohort study in the UK Clinical Practice Research Datalink
Source: BMJ Open. 2016 Dec 12;6(12):e012044. doi: 10.1136/bmjopen-2016-012044 (PMC5168636; doi:10.1136/bmjopen-2016-012044)
Supplement: supplementary file [file bmjopen-2016-012044supp6.pdf]

Power calculations for cohort B where start of follow up is treatment for hypertension

Exposure	Exposed Group	Unexposed Group	Sample Size	Detectable Hazard Ratio
Beta-adrenoceptor blocking drugs	262081	Control	Control	Control
Angiotensin-converting enzyme inhibitors	187256	262081	449337	0.992
Thiazides and related diuretics	176791	262081	438872	0.991
Calcium channel blockers	140621	262081	402702	0.991
Loop diuretics	97675	262081	359756	0.990
Alpha-adrenoceptor blocking drugs	67517	262081	329598	0.988
Centrally acting antihypertensive drugs	25746	262081	287827	0.982
Angiotensin-ii receptor antagonists	13366	262081	275447	0.975
Vasodilator antihypertensive drugs	9679	262081	271760	0.971
Potassium-sparing diuretics	7727	262081	269808	0.968

Power calculations for cohort B where start of follow up is a combination of treatments for hypertension

Exposure	Exposed Group	Unexposed Group	Sample Size	Detectable Hazard Ratio
Beta-adrenoceptor blocking drugs	262081	Control	Control	Control
Loop diuretics & potassium-sparing diuretics	20446	262081	282527	0.980
Potassium-sparing diuretics & thiazides and related diuretics	6513	262081	268594	0.965
Beta-adrenoceptor blocking drugs & thiazides and related diuretics	1789	262081	263870	0.934
Angiotensin-converting enzyme inhibitors & thiazides and related diuretics	624	262081	262705	0.888
Angiotensin-ii receptor antagonists & thiazides and related diuretics	564	262081	262645	0.882
Beta-adrenoceptor blocking drugs & potassium-sparing diuretics & thiazides and related diuretics	46	262081	262127	0.587
Angiotensin-converting enzyme inhibitors & calcium channel blockers	35	262081	262116	0.526
Beta-adrenoceptor blocking drugs & loop diuretics & thiazides	22	262081	262103	0.403

Power calculations for cohort B where start of follow up is treatment for hypercholesterolaemia

Exposure	Exposed Group	Unexposed Group	Sample Size	Detectable Hazard Ratio
Statins	788158	Control	Control	Control
Fibrates	8988	788158	797146	0.970
Bile acid sequestrants	7360	788158	795518	0.967
Omega-3 fatty acid compounds	2344	788158	790502	0.942
Ezetimibe	1345	788158	789503	0.924
Nicotinic acid group	321	788158	788479	0.844

Power calculations for cohort B where start of follow up is a combination of treatments for hypercholesterolaemia

Exposure	Exposed Group	Unexposed Group	Sample Size	Detectable Hazard Ratio
Statins	788158	Control	Control	Control
Ezetimibe & statins	171	788158	788329	0.786

Power calculations for cohort B where start of follow up is treatment for type 2 diabetes

Exposure	Exposed Group	Unexposed Group	Sample Size	Detectable Hazard Ratio
Biguanides	156304	Control	Control	Control
Sulphonylureas	41812	156304	198116	0.985
Other antidiabetic drugs	2471	156304	158775	0.943

Power calculations for cohort B where start of follow up is a combination of treatments for type 2 diabetes

Exposure	Exposed Group	Unexposed Group	Sample Size	Detectable Hazard Ratio
Biguanides	156304	Control	Control	Control
Biguanides & other antidiabetic drugs	213	156304	156517	0.808
