# Supplementary material for: Can commonly prescribed drugs be repurposed for the prevention or treatment of Alzheimer's and other neurodegenerative diseases? Protocol for an observational cohort study in the UK Clinical Practice Research Datalink
Source: BMJ Open. 2016 Dec 12;6(12):e012044. doi: 10.1136/bmjopen-2016-012044 (PMC5168636; doi:10.1136/bmjopen-2016-012044)
Supplement: supplementary file [file bmjopen-2016-012044supp7.pdf]

Power calculations for cohort C where start of follow up is diagnosis of dementia

Exposure	Exposed Group	Unexposed Group	Sample Size	Detectable Hazard Ratio
Treatment for hypertension prior to diagnosis	28586	76885	105471	0.981
Treatment for hypercholesterolaemia prior to diagnosis	26686	78785	105471	0.980
Treatment for type 2 diabetes prior to diagnosis	5867	99604	105471	0.964
Treatment for hypertension post diagnosis	5548	99923	105471	0.963
Treatment for hypercholesterolaemia post diagnosis	5062	100409	105471	0.961
Treatment for type 2 diabetes post diagnosis	1470	104001	105471	0.931

Power calculations for cohort C where start of follow up is diagnosis of Parkinson's disease

Exposure	Exposed Group	Unexposed Group	Sample Size	Detectable Hazard Ratio
Treatment for hypertension prior to diagnosis	6225	14461	20686	0.959
Treatment for hypercholesterolaemia prior to diagnosis	4563	16123	20686	0.955
Treatment for hypertension post diagnosis	2051	18635	20686	0.938
Treatment for hypercholesterolaemia post diagnosis	1842	18844	20686	0.936
Treatment for type 2 diabetes prior to diagnosis	907	19779	20686	0.913
Treatment for type 2 diabetes post diagnosis	357	20329	20686	0.870

Power calculations for cohort C where start of follow up is diagnosis of amyotrophic lateral sclerosis

Exposure	Exposed Group	Unexposed Group	Sample Size	Detectable Hazard Ratio
Treatment for hypertension prior to diagnosis	670	1557	2227	0.881
Treatment for hypercholesterolaemia prior to diagnosis	530	1697	2227	0.874
Treatment for hypertension post diagnosis	152	2075	2227	0.807
Treatment for type 2 diabetes prior to diagnosis	86	2141	2227	0.762
Treatment for hypercholesterolaemia post diagnosis	66	2161	2227	0.739
Treatment for type 2 diabetes post diagnosis	18	2209	2227	0.600
