# Supplementary material for: Can commonly prescribed drugs be repurposed for the prevention or treatment of Alzheimer's and other neurodegenerative diseases? Protocol for an observational cohort study in the UK Clinical Practice Research Datalink
Source: BMJ Open. 2016 Dec 12;6(12):e012044. doi: 10.1136/bmjopen-2016-012044 (PMC5168636; doi:10.1136/bmjopen-2016-012044)
Supplement: supplementary file [file bmjopen-2016-012044supp8.pdf]

Feasibility numbers for the treatments for hypertension

Subclass	# Patients
Alpha-adrenoceptor Blocking Drugs	67517
Angiotensin-converting Enzyme Inhibitors	187256
Angiotensin-converting Enzyme Inhibitors AND Calcium Channel Blockers	35
Angiotensin-converting Enzyme Inhibitors AND Thiazides and Related Diuretics	624
Adrenergic Neurone Blocking Drugs	3
Angiotensin-ii Receptor Antagonists	13366
Angiotensin-ii Receptor Antagonists AND Calcium Channel Blockers	9
Angiotensin-ii Receptor Antagonists AND Calcium Channel Blockers AND Thiazides And Related Diuretics	0
Angiotensin-ii Receptor Antagonists AND Thiazides and Related Diuretics	564
Beta-adrenoceptor Blocking Drugs	262081
Beta-adrenoceptor Blocking Drugs AND Calcium Channel Blockers	3
Beta-adrenoceptor Blocking Drugs AND Loop Diuretics	0
Beta-adrenoceptor Blocking Drugs AND Loop Diuretics AND Thiazides and Related Diuretics	22
Beta-adrenoceptor Blocking Drugs AND Potassium-sparing Diuretics And Aldosterone Antagonists AND Thiazides and Related Diuretics	46
Beta-adrenoceptor Blocking Drugs AND Thiazides and Related Diuretics	1789
Centrally Acting Antihypertensive Drugs	25746
Calcium-channel Blockers	140621
Calcium Channel Blockers AND Thiazides And Related Diuretics	0
Loop Diuretics	97675
Loop Diuretics AND Potassium-sparing Diuretics and Aldosterone Antagonists	20446
Potassium-sparing Diuretics and Aldosterone Antagonists	7727
Potassium-sparing Diuretics and Aldosterone Antagonists AND Thiazides and Related Diuretics	6513
Renin Inhibitors	6
Thiazides and Related Diuretics	176791
Vasodilator Antihypertensive Drugs	9679
**Total**	**1018519**

Feasibility numbers for the treatments for hypercholesterolaemia

**Subclass**	**# Patients**
Bile Acid Sequestrants	7360
Ezetimibe	1345
Ezetimibe AND Statins	171
Fibrates	8988
Nicotinic Acid Group	321
Omega-3 Fatty Acid Compounds	2344
Statins	788158
**Total**	**808687**

Feasibility numbers for the treatments for type 2 diabetes

**Subclass**	**# Patients**
Biguanides	156304
Biguanides AND Other Antidiabetic Drugs	213
Other Antidiabetic Drugs	2471
Sulphonylureas	41812
**Total**	**200800**

Feasibility numbers for the neurodegenerative disease diagnoses

Diagnosis	# Patients
Dementia	105471
Amyotrophic lateral sclerosis	2227
Parkinson's disease	20686

Feasibility numbers for the treatment for dementia

Treatment	# Patients
Treatment for Dementia	33071

Feasibility numbers for the treatments of interest, restricted to patients with a neurodegenerative disease of interest

Diagnosis	Treatment	Treatment First	Diagnosis & Treatment Received Together	Diagnosis First	Total
**Dementia**	Hypertension	28586	132	5548	34266
	Hypercholesterolaemia	26686	208	5062	31956
	Type 2 Diabetes	5687	16	1470	7173
**Amyotrophic lateral sclerosis**	Hypertension	670	1	152	823
	Hypercholesterolaemia	530	0	66	596
	Type 2 Diabetes	86	0	18	104
**Parkinson's disease**	Hypertension	6225	41	2051	8317
	Hypercholesterolaemia	4563	21	1842	6426
	Type 2 Diabetes	907	4	357	1268

Average age of patients receiving treatments and diagnoses of interest

Event	Average Age (years)	Standard Deviation (years)
Hypertension treatment	61.91	12.91
Hypercholesterolaemia treatment	64.61	11.42
Type 2 diabetes treatment	63.66	12.11
Dementia treatment	79.98	7.98
Dementa diagnosis	81.43	8.5
Amyotrophic lateral sclerosis diagnosis	68.45	10.89
Parkinson's disease diagnosis	74.62	9.76
